# Supplementary material for: Alternative Analysis Approaches for the Assessment of Pilot Bioavailability/Bioequivalence Studies
Source: Pharmaceutics. 2023 May 7;15(5):1430. doi: 10.3390/pharmaceutics15051430 (PMC10222359; doi:10.3390/pharmaceutics15051430)
Supplement: Supplementary file 1 [file pharmaceutics-15-01430-s001.zip › pharmaceutics-2362186-supplementary.pdf]

ALTERNATIVE ANALYSIS APPROACHES FOR THE ASSESSMENT OF PILOT BIOAVAILABILITY/BIOEQUIVALENCE STUDIES

Sara Carolina Henriques [1,2], João Albuquerque [2,3], Paulo Paixão [1], Luís Almeida [2], Nuno Elvas Silva [1,2]

[1] Research Institute for Medicines (iMed.ULisboa), Faculty of Pharmacy, Universidade de Lisboa. Lisboa, Portugal;

[2] BlueClinical, Porto, Portugal

[3] Centro de Estatística e Aplicações, Faculdade de Ciências, Universidade de Lisboa, Lisboa, Portugal

Appendix SA. Descriptive Statistics of Simulated Pharmacokinetic Data

Table of Contents

Table of Contents 2

Appendix SA.1. Simulated Baseline Studies 3

Appendix SA.1.1. Simulated Concentrations 3

Appendix SA.1.2. Simulated Non-Compartmental Analysis Parameters 5

Appendix SA.1.3. Simulated Pilot Studies Results 10

Appendix SA.2. Simulated Studies with Variability in k_a 16

Appendix SA.2.1. Simulated Pharmacokinetic Parameters 16

Appendix SA.2.2. Simulated Concentrations 19

Appendix SA.2.3. Simulated Non-Compartmental Analysis Parameters 21

Appendix SA.2.4. Simulated Pilot Studies Results 28

Appendix SA.3. Simulated Studies with Variability in V 58

Appendix SA.3.1. Simulated Pharmacokinetic Parameters 58

Appendix SA.3.2. Simulated Concentrations 61

Appendix SA.3.3. Simulated Non-Compartmental Analysis Parameters 63

Appendix SA.3.4. Simulated Pilot Studies Results 70

Appendix SA.4. Simulated Studies with Variability in k_e 100

Appendix SA.4.1. Simulated Pharmacokinetic Parameters 100

Appendix SA.4.2. Simulated Concentrations 103

Appendix SA.4.3. Simulated Non-Compartmental Analysis Parameters 105

Appendix SA.4.4. Simulated Pilot Studies Results 112

Appendix SA.1. Simulated Baseline Studies

Appendix SA.1.1. Simulated Concentrations

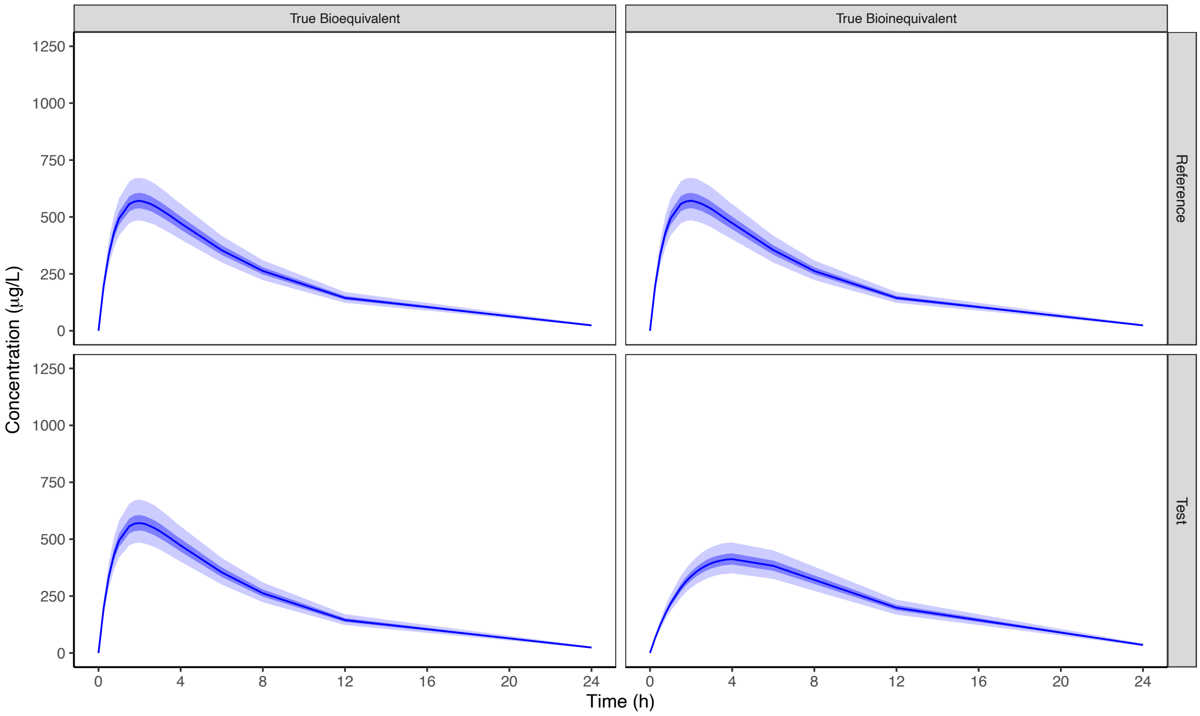

Figure S.1. 95% Confidence Interval of Simulated Concentration-Time Data for Baseline Studies – Linear Scale

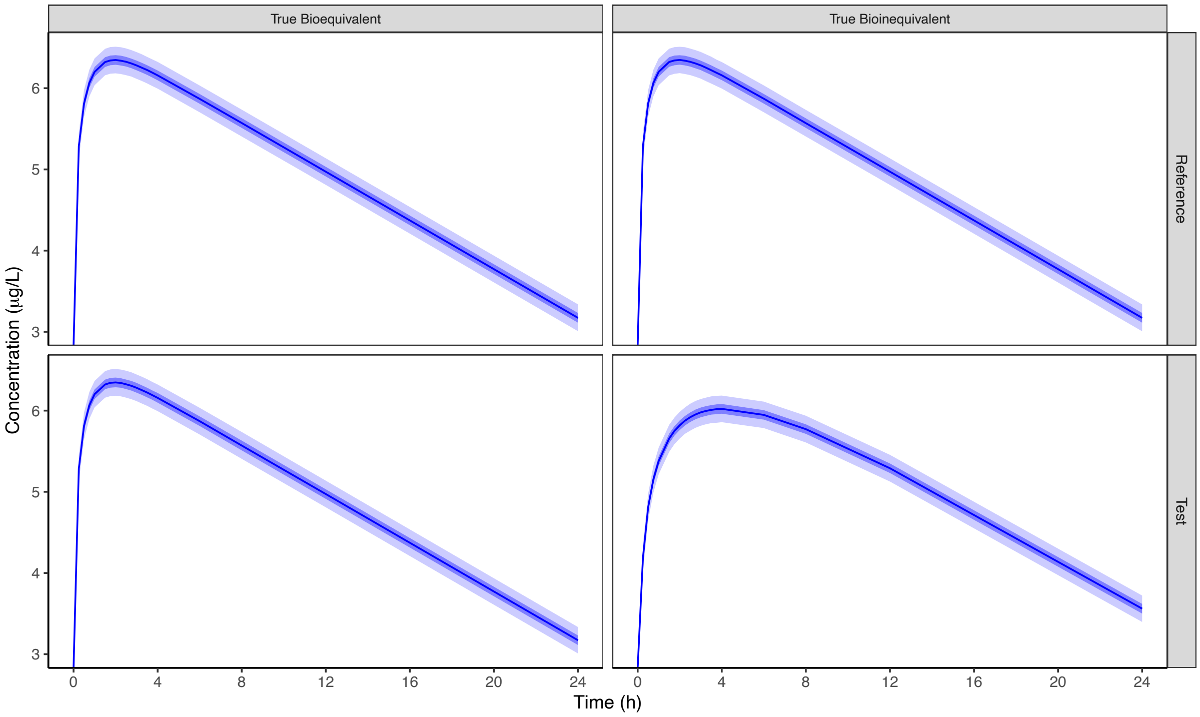

Figure S.2. 95% Confidence Interval of Simulated Concentration-Time Data for Baseline Studies – Semi-Logarithmic Scale

Appendix SA.1.2. Simulated Non-Compartmental Analysis Parameters

Table S.1. Descriptive Statistics of C_{max} (µg/L) Derived from Simulations for Baseline Studies

	True Bioequivalent		True Bioinequivalent	
	**Reference**	**Test**	**Reference**	**Test**
n	21000	21000	21000	21000
G_{Mean}	642.51	642.72	642.23	460.69
GSD	1.06	1.06	1.06	1.06
GCV%	6.1	6.2	6.1	6.2
G_{mean} 95% CI	641.98 – 643.05	642.18 – 643.26	641.70 – 642.76	460.30 – 461.08
A_{Mean}	643.73	643.96	643.44	461.59
SD	39.99	40.48	39.81	29.17
SE	0.28	0.28	0.27	0.20
CV%	6.2	6.3	6.2	6.3
Variance	1599.01	1638.56	1584.78	850.61
A_{mean} 95% CI	643.19 – 644.27	643.42 – 644.51	642.90 – 643.97	461.20 – 461.99
Minimum	528.65	519.55	531.42	375.95
Q1	616.03	615.31	615.19	440.91
Median	639.87	639.78	639.72	458.77
Q3	667.35	667.99	667.28	479.29
Maximum	871.83	879.17	853.28	608.76

n: Number of observations, G_{mean}: Geometric mean, GSD: Geometric standard deviation, GCV%: Geometric coefficient of variation, G_{mean} 95% CI: 95% confidence interval of the geometric mean, A_{mean}: Arithmetic mean, SD: Standard deviation, SD: Standard error, CV%: Coefficient of variation, A_{mean} 95% CI: 95% confidence interval of the arithmetic mean, Q1: 1st (25%) quartile, Q3: 3rd (75%) quartile

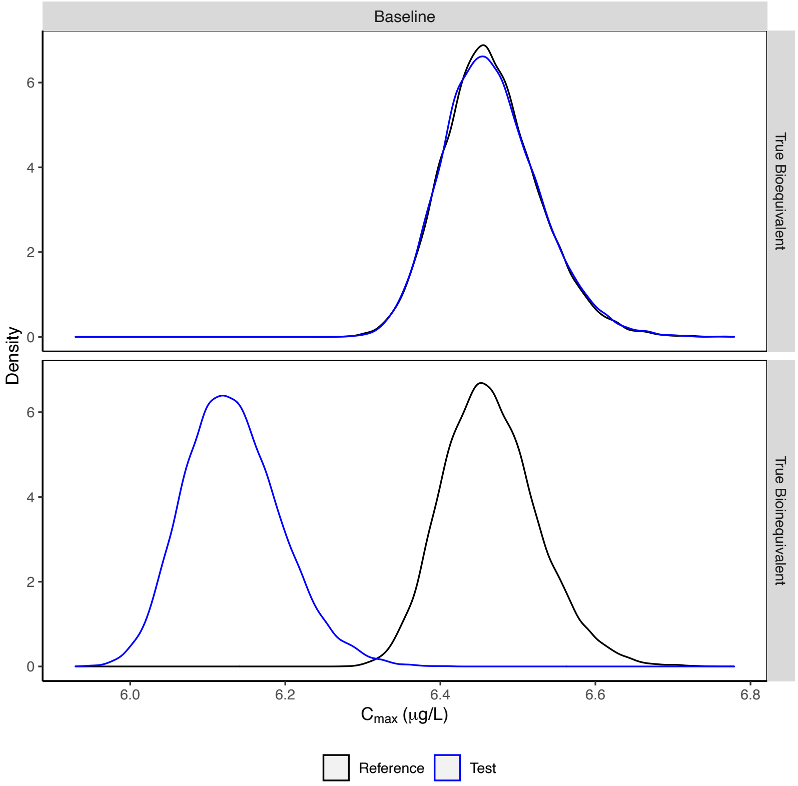

Figure S.3. Distribution of *Ln*-Transformed C_{max}, Derived from Simulations for Baseline Studies

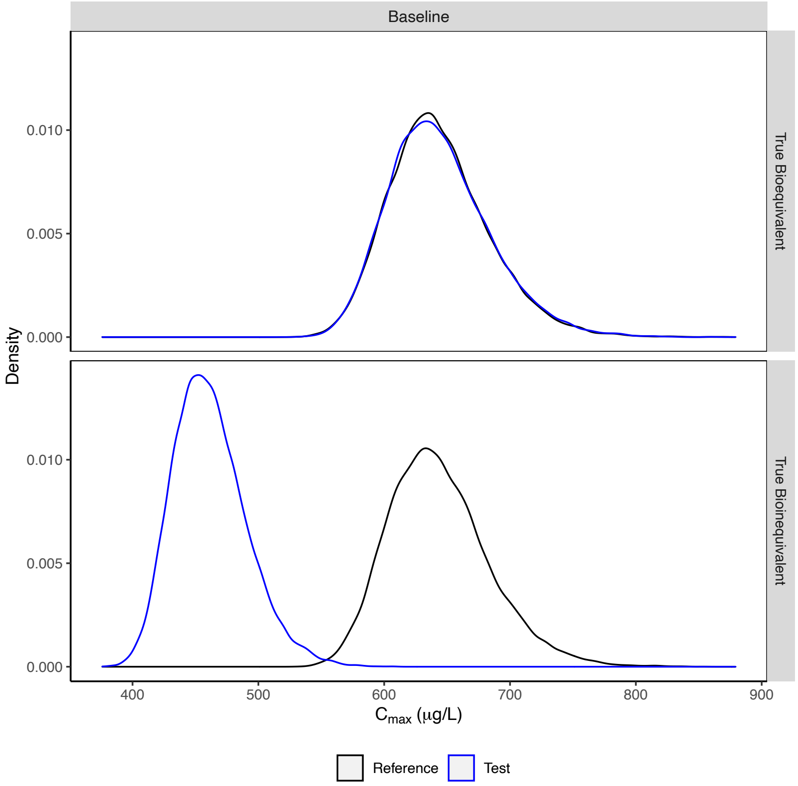

Figure S.4. Distribution of Untransformed C_{max}, Derived from Simulations for Baseline Studies

Table S.2. Descriptive Statistics of t_{max} (h) Derived from Simulations for Baseline Studies

	True Bioequivalent		True Bioinequivalent	
	**Reference**	**Test**	**Reference**	**Test**
n	21000	21000	21000	21000
Minimum	0.75	0.75	0.75	1.75
Q1	1.75	1.75	1.75	3.25
Median	2.25	2.25	2.25	3.50
Q3	2.50	2.50	2.50	4.00
Maximum	4.00	4.00	4.00	8.00

n: Number of observations, Q1: 1st (25%) quartile, Q3: 3rd (75%) quartile

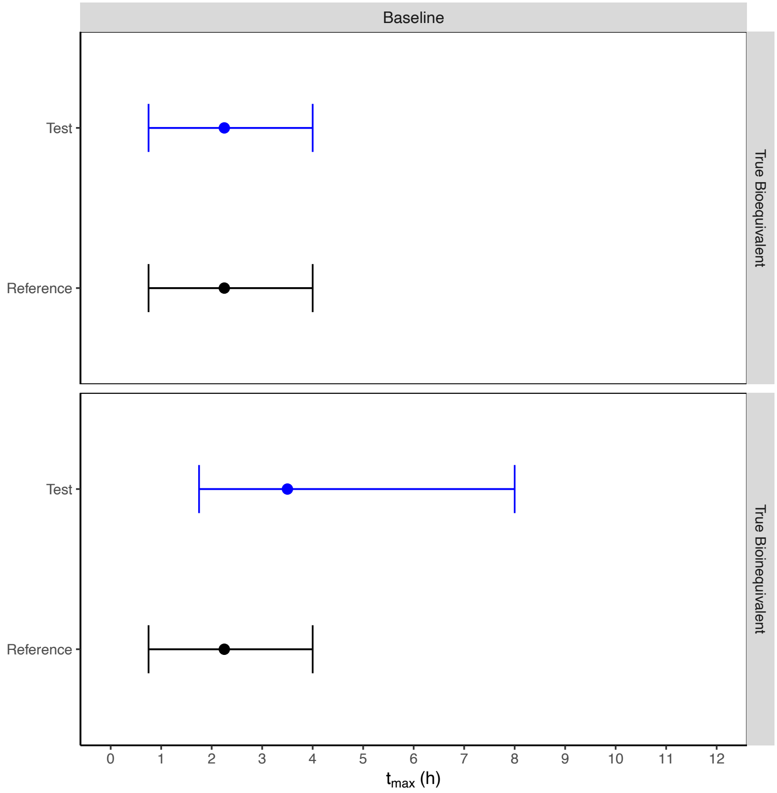

Figure S.5. Distribution of Untransformed t_{max}, Derived from Simulations for Baseline Studies

Table S.3. Descriptive Statistics of AUC_{0-t} (µg.h/L) Derived from Simulations for Baseline Studies

	True Bioequivalent		True Bioinequivalent	
	**Reference**	**Test**	**Reference**	**Test**
n	21000	21000	21000	21000
G_{Mean}	4950.44	4950.48	4951.08	4839.58
GSD	1.03	1.03	1.03	1.04
GCV%	3.1	3.1	3.1	3.8
G_{mean} 95% CI	4948.33 – 4952.55	4948.38 – 4952.58	4948.98 – 4953.18	4837.13 – 4842.04
A_{Mean}	4952.89	4952.92	4953.52	4843
SD	156.08	155.61	155.48	182.16
SE	1.08	1.07	1.07	1.26
CV%	3.2	3.1	3.1	3.8
Variance	24360.26	24214.54	24173.22	33181.9
A_{mean} 95% CI	4950.78 – 4955.00	4950.82 – 4955.03	4951.41 – 4955.62	4840.54 – 4845.47
Minimum	4289.91	4362.04	4325.83	4125.3
Q1	4845.38	4846.53	4846.47	4718.22
Median	4949.15	4948.27	4950.03	4838.51
Q3	5055.84	5055.47	5058.05	4961.98
Maximum	5628.86	5577.39	5612.09	5714.7

n: Number of observations, G_{mean}: Geometric mean, GSD: Geometric standard deviation, GCV%: Geometric coefficient of variation, G_{mean} 95% CI: 95% confidence interval of the geometric mean, A_{mean}: Arithmetic mean, SD: Standard deviation, SD: Standard error, CV%: Coefficient of variation, A_{mean} 95% CI: 95% confidence interval of the arithmetic mean, Q1: 1st (25%) quartile, Q3: 3rd (75%) quartile

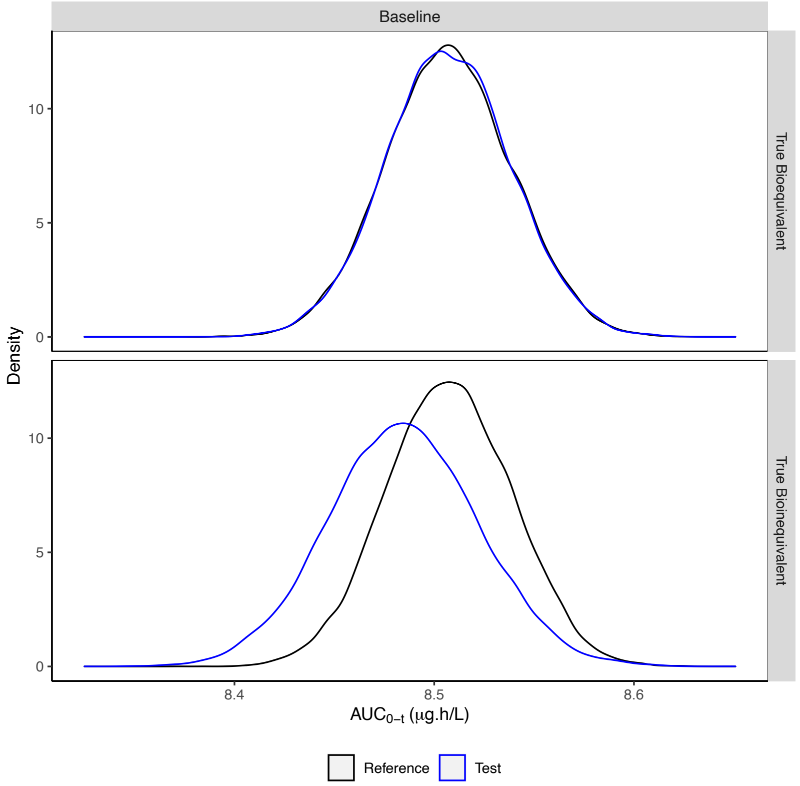

Figure S.6. Distribution of *Ln*-Transformed AUC_{0-t}, Derived from Simulations for Baseline Studies

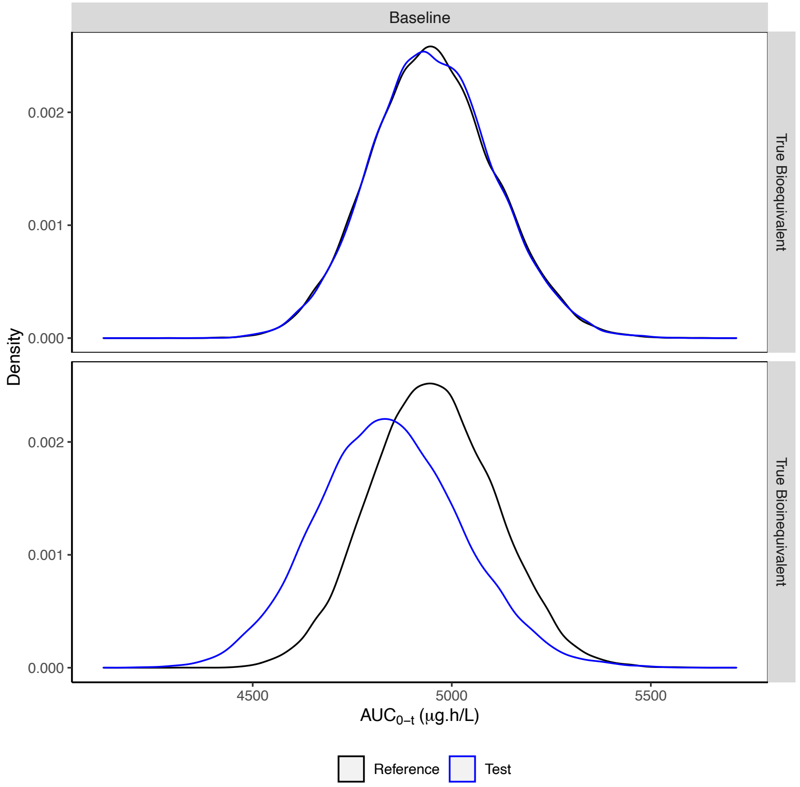

Figure S.7. Distribution of Untransformed AUC_{0-t}, Derived from Simulations for Baseline Studies

Appendix SA.1.3. Simulated Pilot Studies Results

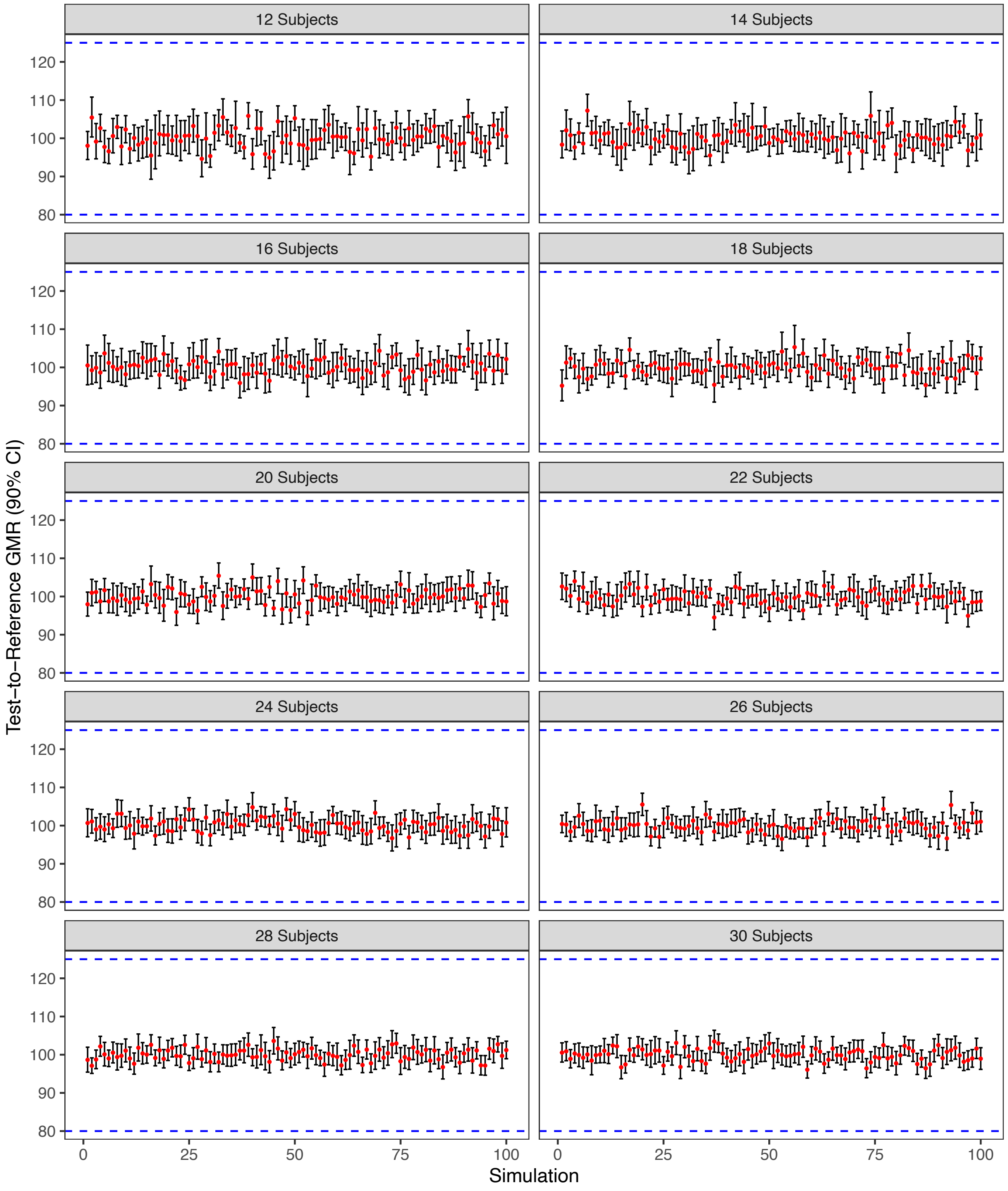

Figure S.8. Average Bioequivalence Analysis Results (Test-to-Reference GMR and Corresponding 90% CI) for C_{max} Derived from True Bioequivalent Baseline Simulations

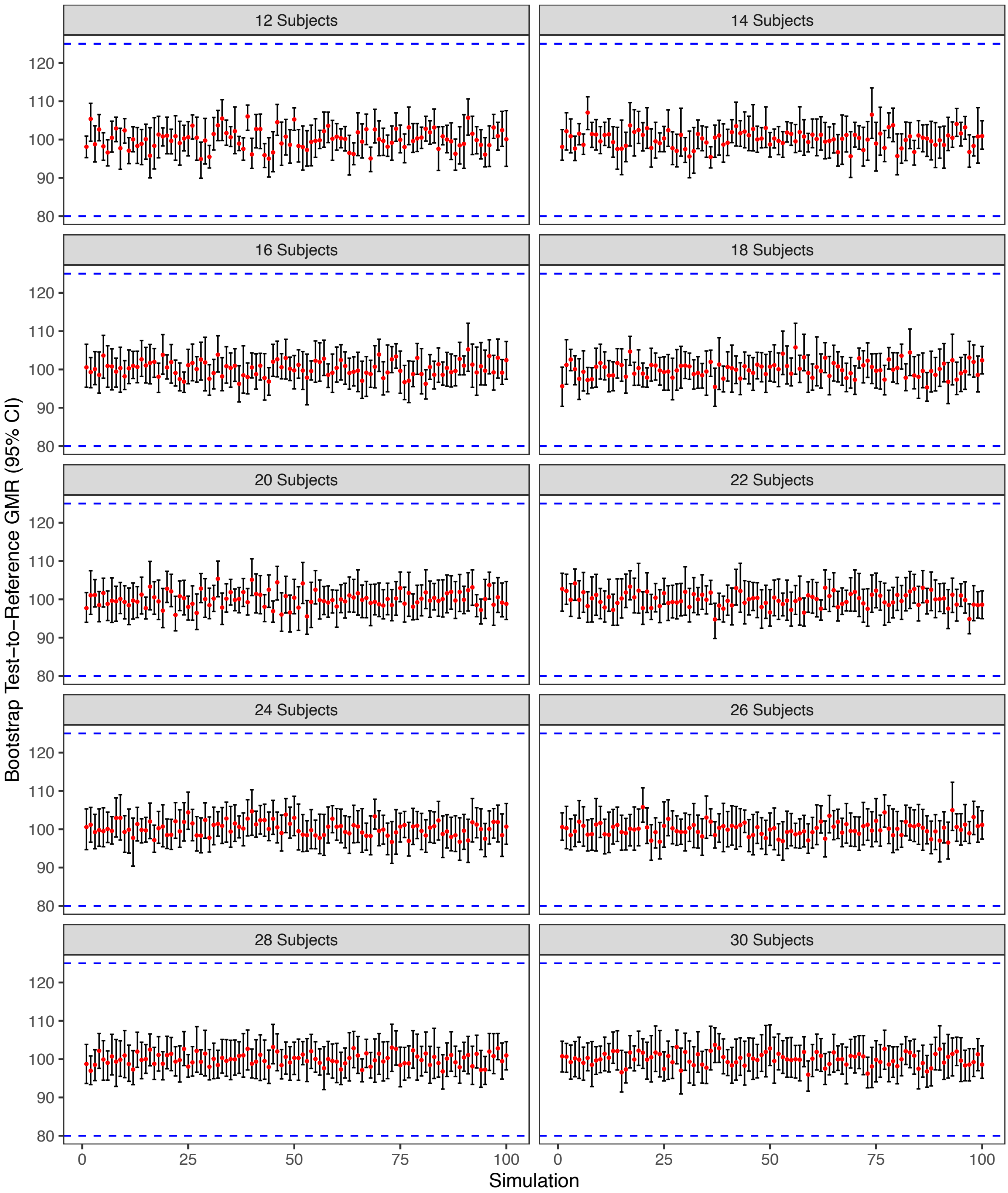

Figure S.9. Bootstrap Bioequivalence Analysis Results (Test-to-Reference GMR and Corresponding 95% CI) for C_{max} Derived from True Bioequivalent Baseline Simulations

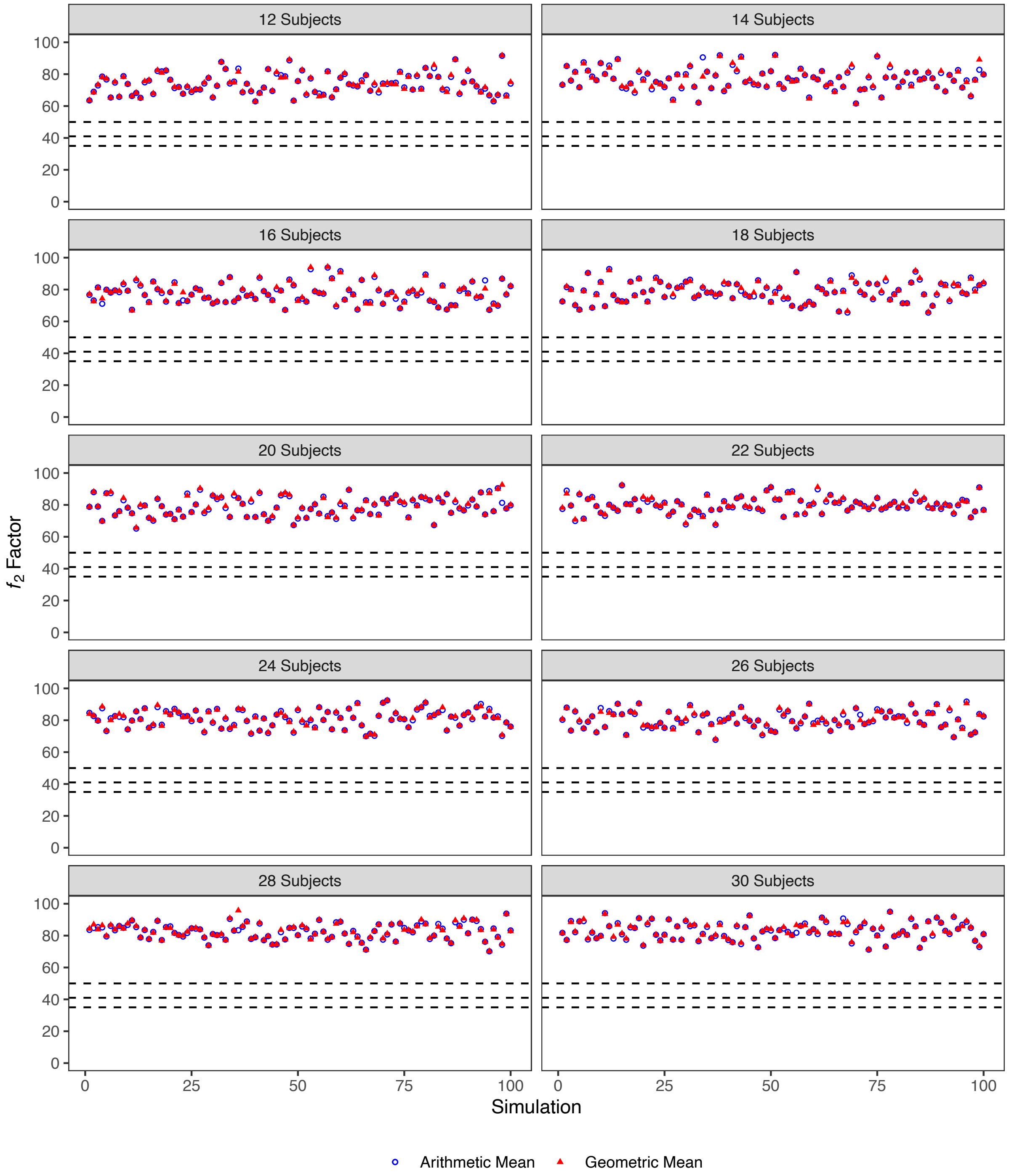

Figure S.10. Similarity f_2 Factor Results, Derived from Arithmetic and Geometric Mean Concentration-Time Profiles Until the Reference C_{max}, Derived from True Bioequivalent Baseline Simulations

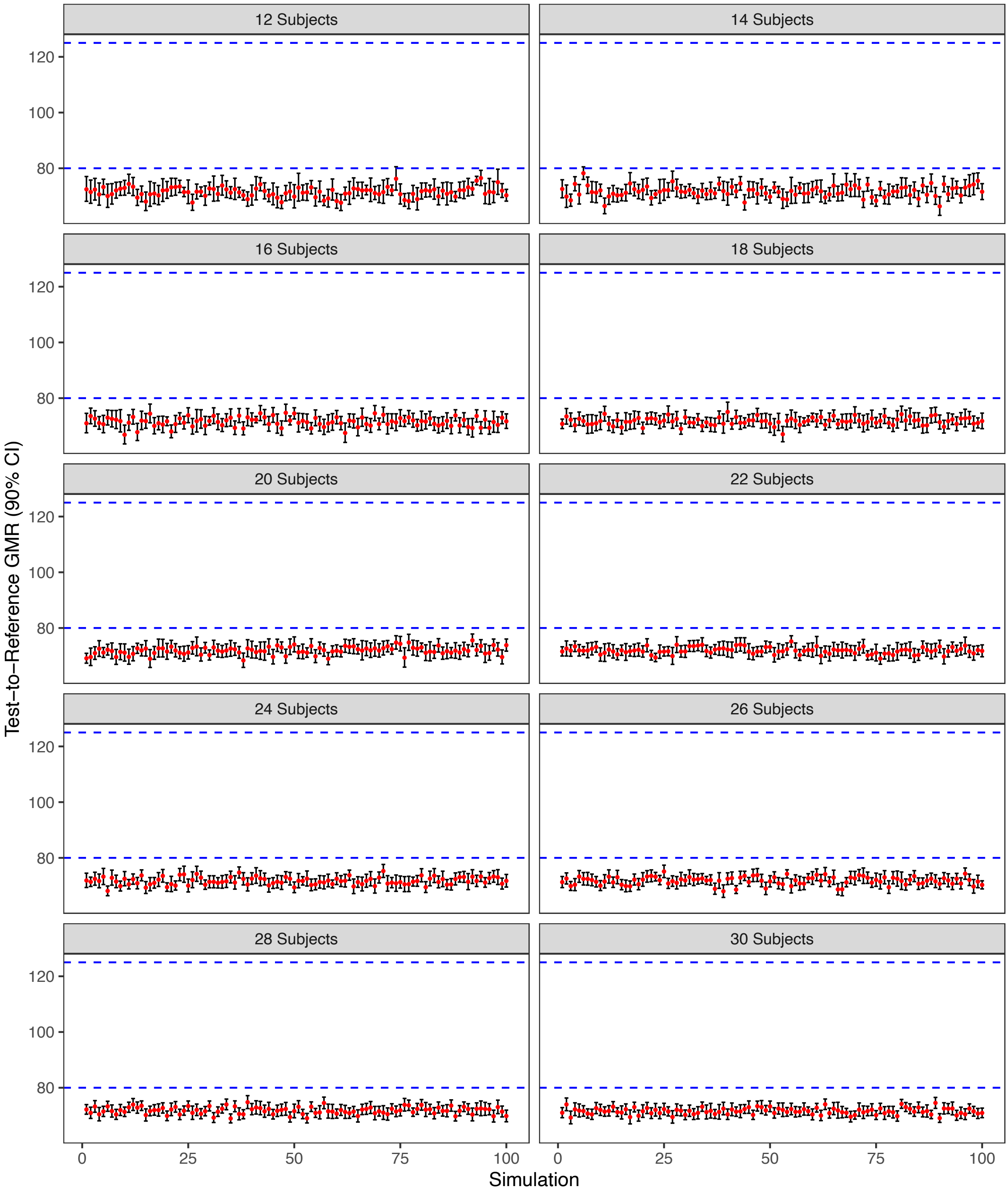

Figure S.11. Average Bioequivalence Analysis Results (Test-to-Reference GMR and Corresponding 90% CI) for C_{max} Derived from True Bioinequivalent Baseline Simulations

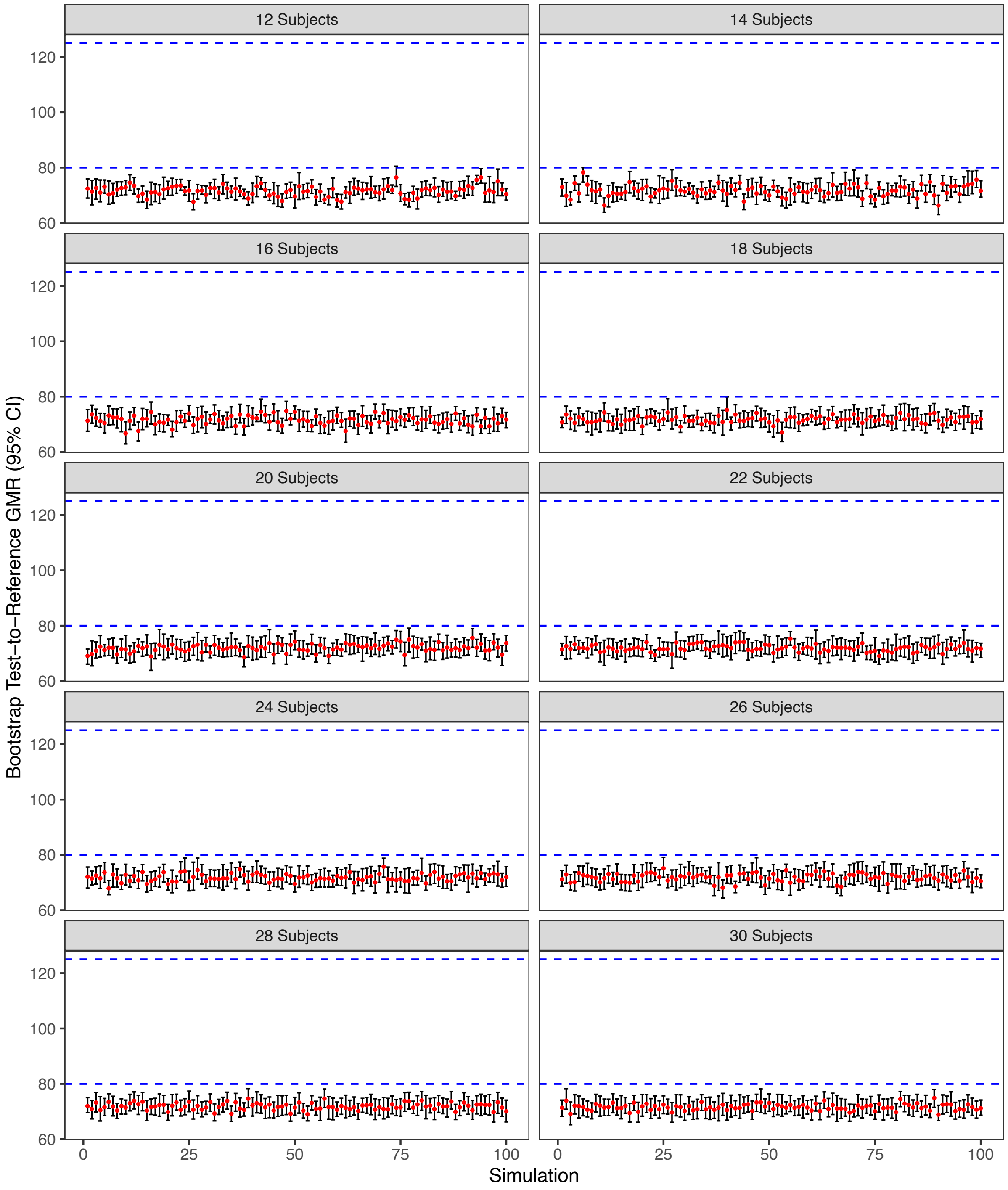

Figure S.12. Bootstrap Bioequivalence Analysis Results (Test-to-Reference GMR and Corresponding 95% CI) for C_{max} Derived from True Bioinequivalent Baseline Simulations

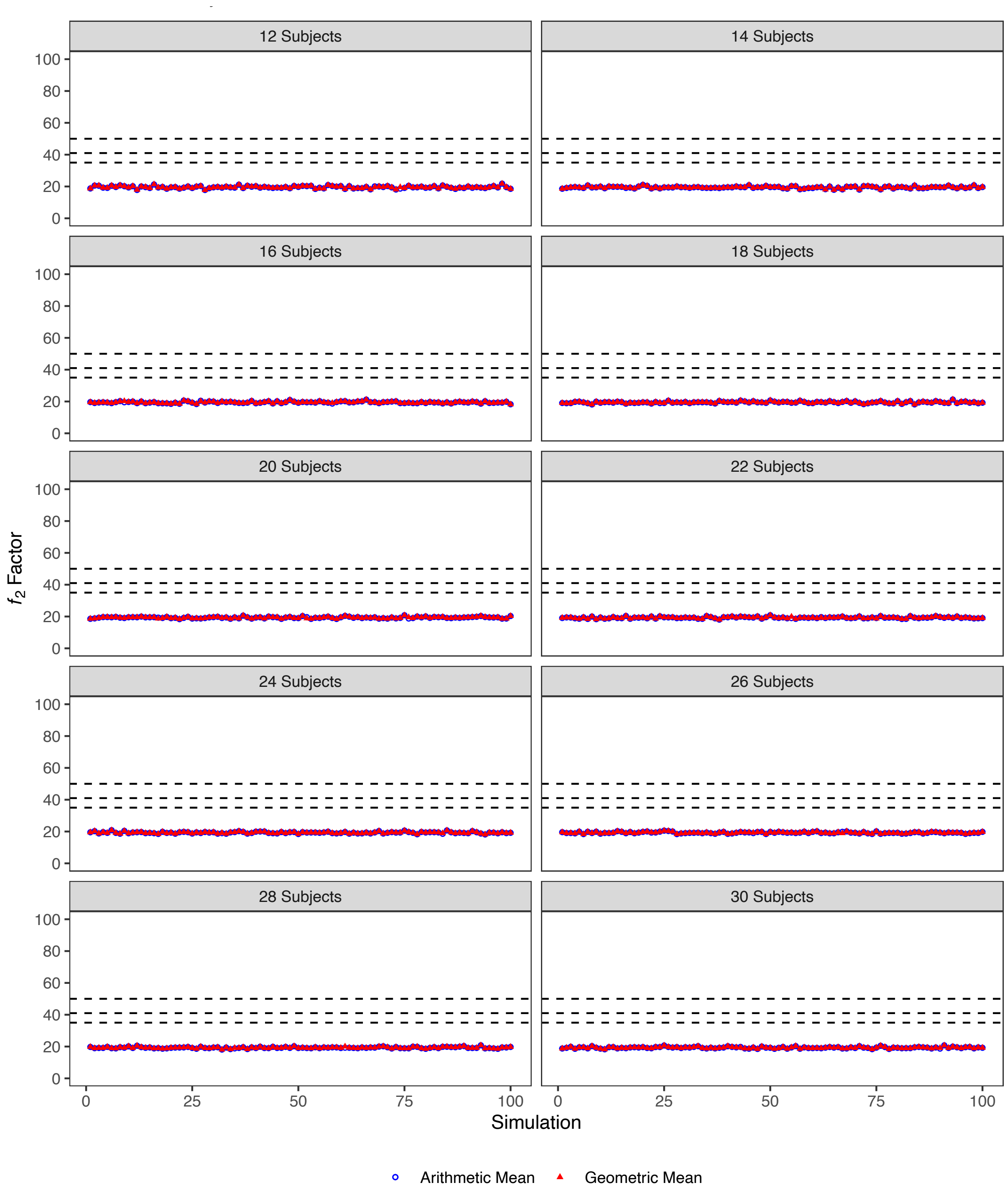

Figure S.13. Similarity f_2 Factor Results, Derived from Arithmetic and Geometric Mean Concentration-Time Profiles Until the Reference C_{max}, Derived from True Bioequivalent Baseline Simulations

Appendix SA.2. Simulated Studies with Variability in k_a

Appendix SA.2.1. Simulated Pharmacokinetic Parameters

Table S.4. Descriptive Statistics of Simulated k_a for True Bioequivalent Studies

	30% IIV 0% IOV		30% IIV 10% IOV		30% IIV 20% IOV		30% IIV 30% IOV		0% IIV 45% IOV	
	Reference	Test	Reference	Test	Reference	Test	Reference	Test	Reference	Test
n	21000	21000	21000	21000	21000	21000	21000	21000	21000	21000
G_{Mean}	1.22	1.22	1.22	1.23	1.22	1.22	1.22	1.22	1.22	1.22
GSD	1.35	1.35	1.37	1.37	1.44	1.43	1.52	1.53	1.57	1.58
GCV%	30.5	30.5	32.1	32.1	37.3	37.3	44.1	44.3	47.6	48.1
G_{mean} 95% CI	1.21 – 1.22	1.21 – 1.22	1.22 – 1.23	1.22 – 1.23	1.21 – 1.23	1.22 – 1.23	1.21 – 1.23	1.21 – 1.22	1.21 – 1.23	1.21 – 1.23
A_{Mean}	1.27	1.27	1.28	1.29	1.30	1.30	1.33	1.33	1.35	1.35
SD	0.39	0.39	0.41	0.41	0.48	0.48	0.59	0.59	0.64	0.65
SE	0.00	0.00	0.00	0.00	0.00	0.00	0.00	0.00	0.00	0.00
CV%	30.4	30.4	32.1	32.1	37.1	37.0	44.0	44.4	47.7	48.1
Variance	0.15	0.15	0.17	0.17	0.23	0.23	0.34	0.35	0.42	0.42
A_{mean} 95% CI	1.27 – 1.28	1.27 – 1.28	1.28 – 1.29	1.28 – 1.29	1.30 – 1.31	1.30 – 1.31	1.32 – 1.34	1.32 – 1.34	1.34 – 1.36	1.35 – 1.36
Minimum	0.31	0.31	0.34	0.39	0.29	0.29	0.22	0.23	0.20	0.17
Q1	1.00	1.00	0.99	0.99	0.96	0.96	0.91	0.91	0.90	0.90
Median	1.22	1.22	1.22	1.23	1.22	1.23	1.23	1.21	1.22	1.22
Q3	1.50	1.50	1.51	1.51	1.56	1.56	1.62	1.62	1.65	1.66
Maximum	4.01	4.01	3.87	3.74	4.93	4.53	6.37	7.83	8.17	9.57

n: Number of observations, G_{mean}: Geometric mean, GSD: Geometric standard deviation, GCV%: Geometric coefficient of variation, G_{mean} 95% CI: 95% confidence interval of the geometric mean, A_{mean}: Arithmetic mean, SD: Standard deviation, SD: Standard error, CV%: Coefficient of variation, A_{mean} 95% CI: 95% confidence interval of the arithmetic mean, Q1: 1st (25%) quartile, Q3: 3rd (75%) quartile

Table S.5. Descriptive Statistics of Simulated k_a for True Bioinequivalent Studies

	30% IIV 0% IOV		30% IIV 10% IOV		30% IIV 20% IOV		30% IIV 30% IOV		0% IIV 45% IOV	
	**Reference**	**Test**	**Reference**	**Test**	**Reference**	**Test**	**Reference**	**Test**	**Reference**	**Test**
n	21000	21000	21000	21000	21000	21000	21000	21000	21000	21000
G_{Mean}	1.22	0.37	1.22	0.37	1.22	0.37	1.22	0.36	1.22	0.37
GSD	1.35	1.35	1.37	1.37	1.43	1.43	1.53	1.53	1.57	1.56
GCV%	30.4	30.4	32.5	32.3	37.1	37.0	44.6	44.6	47.5	46.9
G_{mean} 95% CI	1.21 – 1.22	0.36 – 0.37	1.21 – 1.22	0.36 – 0.37	1.21 – 1.22	0.36 – 0.37	1.21 – 1.23	0.36 – 0.37	1.22 – 1.23	0.37 – 0.37
A_{Mean}	1.27	0.38	1.28	0.38	1.30	0.39	1.33	0.40	1.35	0.41
SD	0.39	0.12	0.42	0.12	0.48	0.14	0.60	0.18	0.64	0.19
SE	0.00	0.00	0.00	0.00	0.00	0.00	0.00	0.00	0.00	0.00
CV%	30.7	30.7	32.5	32.4	37.1	37.1	44.8	44.8	47.1	46.8
Variance	0.15	0.01	0.17	0.02	0.23	0.02	0.36	0.03	0.41	0.04
A_{mean} 95% CI	1.27 – 1.28	0.38 – 0.38	1.27 – 1.28	0.38 – 0.39	1.29 – 1.31	0.39 – 0.39	1.33 – 1.34	0.40 – 0.40	1.35 – 1.36	0.40 – 0.41
Minimum	0.42	0.13	0.39	0.11	0.27	0.07	0.22	0.06	0.17	0.05
Q1	0.99	0.30	0.98	0.29	0.95	0.29	0.91	0.27	0.91	0.27
Median	1.22	0.36	1.21	0.37	1.22	0.37	1.22	0.37	1.23	0.37
Q3	1.49	0.45	1.51	0.45	1.55	0.47	1.62	0.49	1.67	0.50
Maximum	4.54	1.36	4.71	1.59	4.66	1.38	5.89	2.18	6.68	2.01

n: Number of observations, G_{mean}: Geometric mean, GSD: Geometric standard deviation, GCV%: Geometric coefficient of variation, G_{mean} 95% CI: 95% confidence interval of the geometric mean, A_{mean}: Arithmetic mean, SD: Standard deviation, SD: Standard error, CV%: Coefficient of variation, A_{mean} 95% CI: 95% confidence interval of the arithmetic mean, Q1: 1st (25%) quartile, Q3: 3rd (75%) quartile

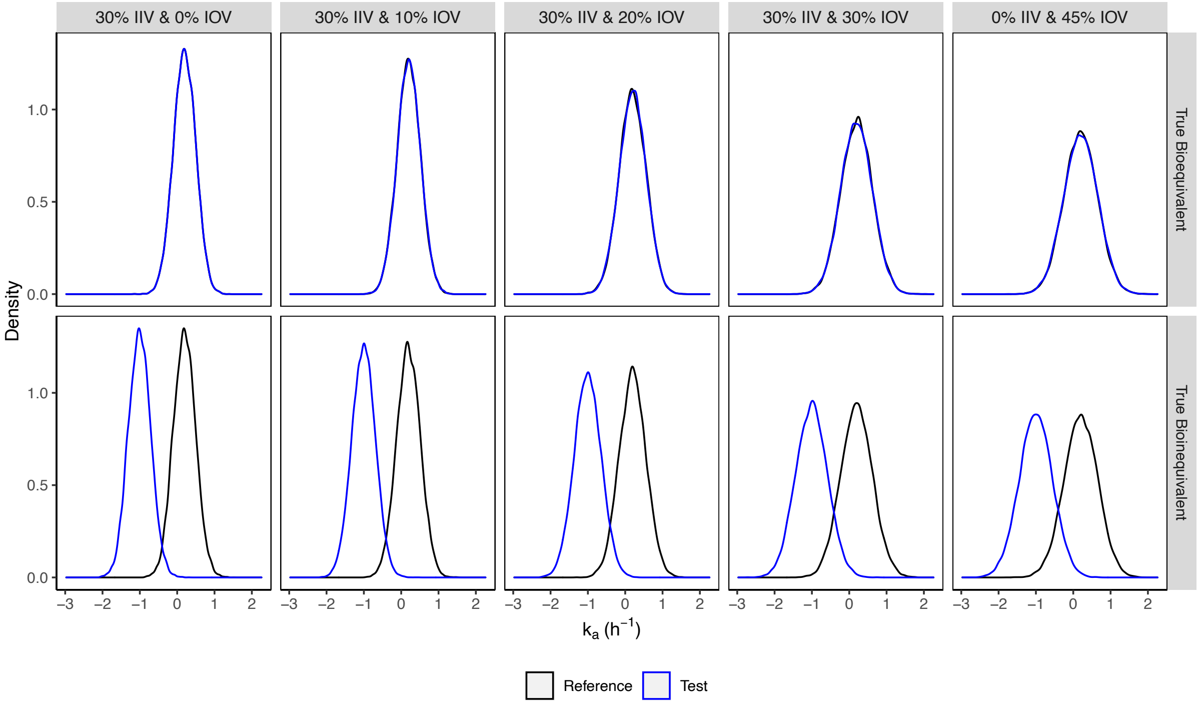

Figure S.14. Distribution of Simulated *Ln*-Transformed k_a

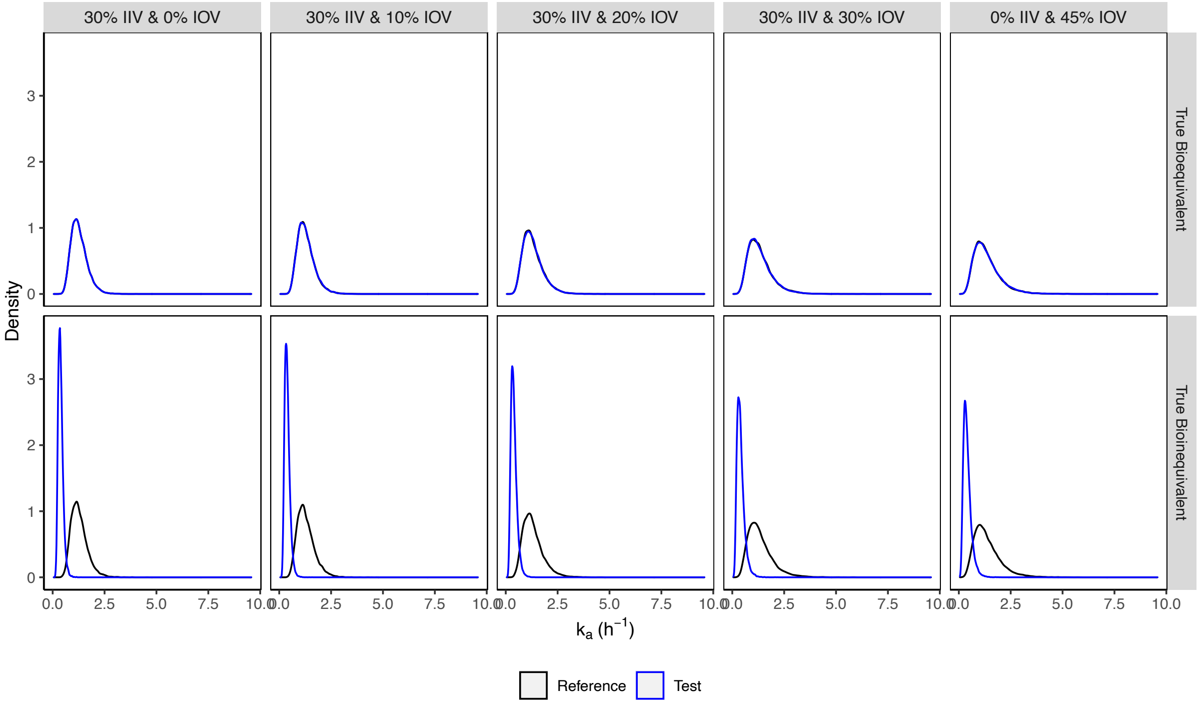

Figure S.15. Distribution of Simulated Untransformed k_a

Appendix SA.2.2. Simulated Concentrations

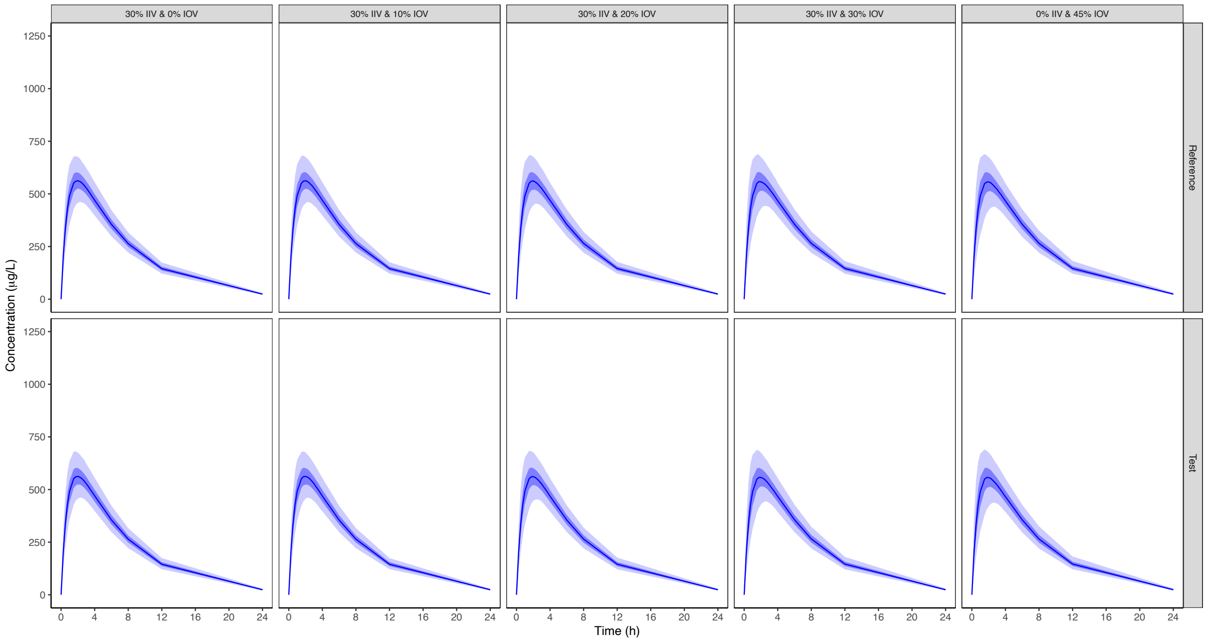

Figure S.16. 95% Confidence Interval of Simulated Concentration-Time Data, for True Bioequivalent Studies, with Variability in k_a – Linear Scale

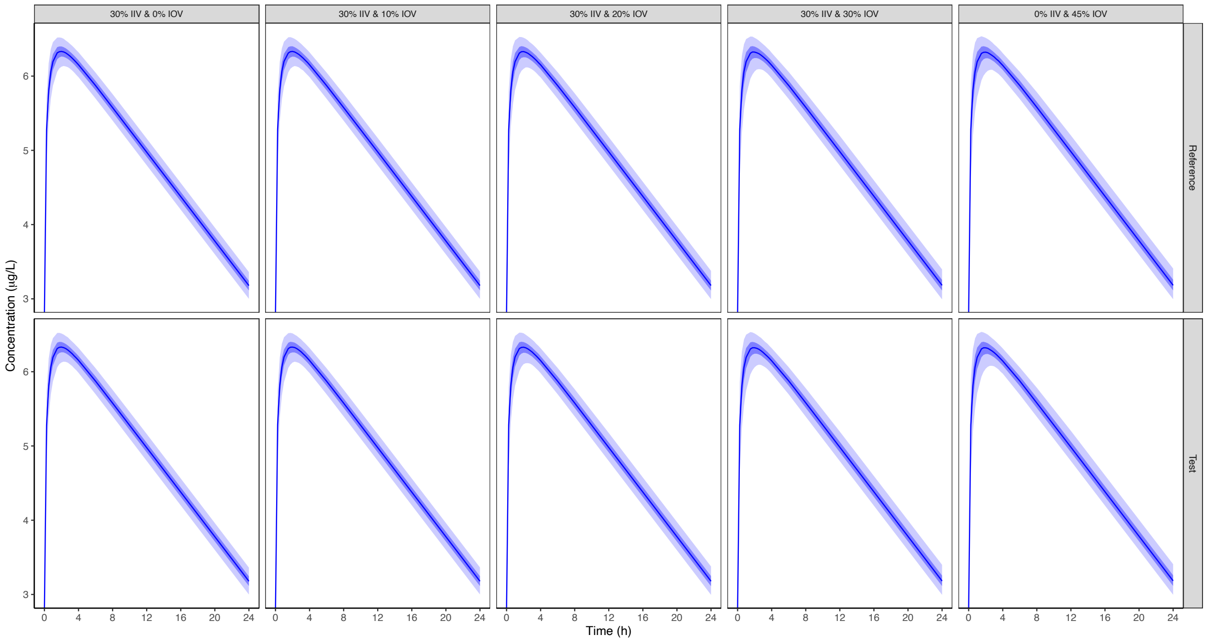

Figure S.17. 95% Confidence Interval of Simulated Concentration-Time Data, for True Bioequivalent Studies, with Variability in k_a – Semi-Logarithmic Scale

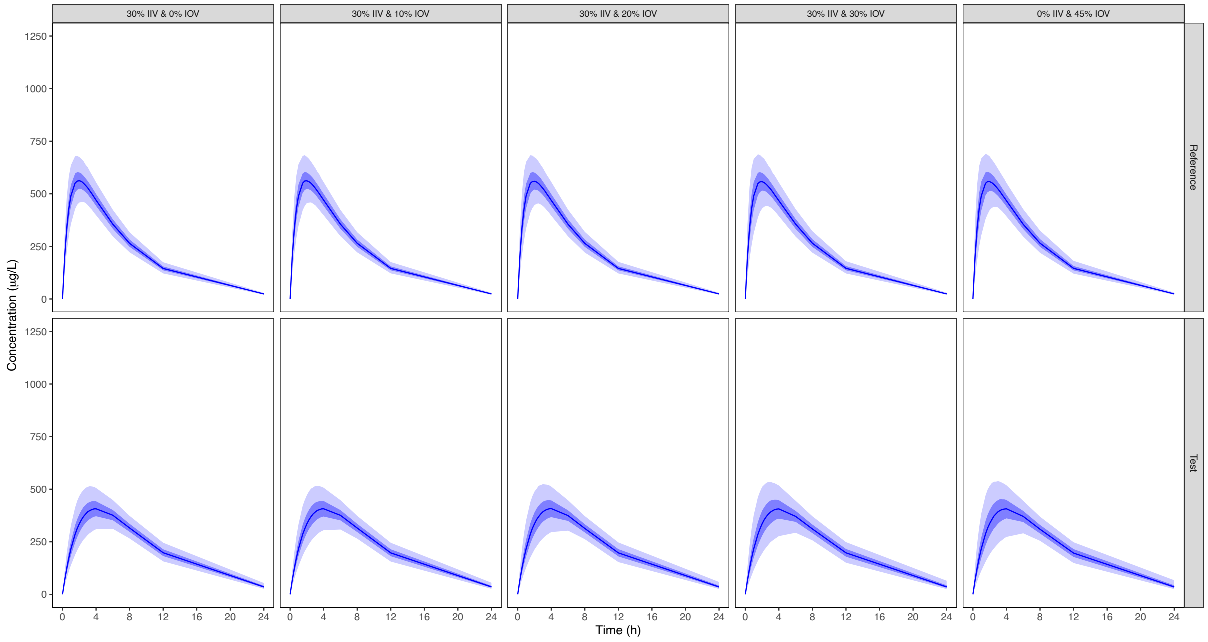

Figure S.18. 95% Confidence Interval of Simulated Concentration-Time Data, for True Bioinequivalent Studies, with Variability in k_a – Linear Scale

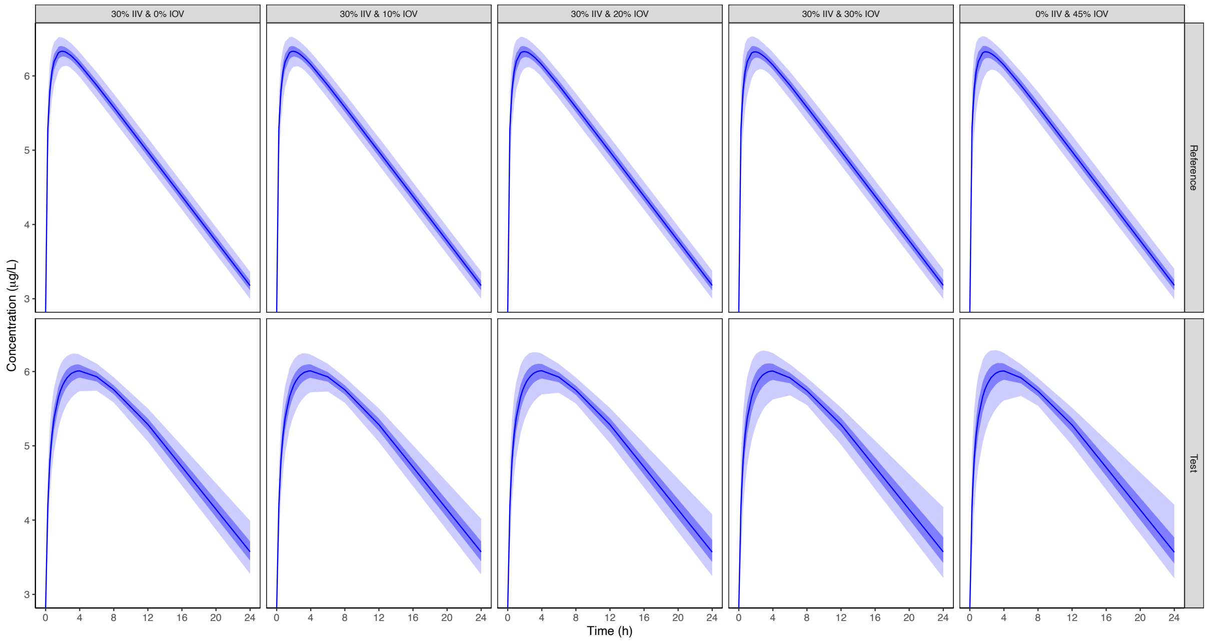

Figure S.19. 95% Confidence Interval of Simulated Concentration-Time Data, for True Bioinequivalent Studies, with Variability in k_a – Semi-Logarithmic Scale

Appendix SA.2.3. Simulated Non-Compartmental Analysis Parameters

Table S.6. Descriptive Statistics of C_{max} (µg/L) Derived from Simulated True Bioequivalent Studies, with Variability in k_a

	30% IIV 0% IOV		30% IIV 10% IOV		30% IIV 20% IOV		30% IIV 30% IOV		0% IIV 45% IOV	
	Reference	Test	Reference	Test	Reference	Test	Reference	Test	Reference	Test
n	21000	21000	21000	21000	21000	21000	21000	21000	21000	21000
G_{Mean}	638.07	638.16	638.10	638.09	636.60	636.33	634.66	633.73	633.13	632.84
GSD	1.08	1.08	1.08	1.09	1.09	1.09	1.10	1.10	1.11	1.11
GCV%	7.9	8.0	8.0	8.2	8.8	8.7	9.6	9.7	10.2	10.3
G_{mean} 95% CI	637.39 – 638.75	637.48 – 638.85	637.41 – 638.80	637.39 – 638.80	635.85 – 637.36	635.58 – 637.08	633.84 – 635.49	632.90 – 634.57	632.26 – 634.01	631.96 – 633.71
A_{Mean}	640.09	640.18	640.16	640.28	639.03	638.73	637.59	636.71	636.39	636.15
SD	51.52	51.21	51.56	56.25	55.96	55.56	60.98	61.68	64.19	65.88
SE	0.36	0.35	0.36	0.39	0.39	0.38	0.42	0.43	0.44	0.45
CV%	8.0	8.0	8.1	8.8	8.8	8.7	9.6	9.7	10.1	10.4
Variance	2654.44	2622.32	2658.28	3163.54	3131.14	3086.6	3718.11	3804.46	4120.20	4339.63
A_{mean} 95% CI	639.39 – 640.79	639.49 – 640.88	639.47 – 640.86	639.52 – 641.04	638.28 – 639.79	637.98 – 639.48	636.76 – 638.41	635.88 – 637.55	635.52 – 637.26	635.26 – 637.04
Minimum	436.96	299.99	444.46	418.16	370.73	383.58	372.26	363.94	349.28	314.16
Q1	606.00	606.01	605.73	605.78	602.56	602.69	599.36	597.63	596.83	596.34
Median	637.47	637.83	637.45	637.71	637.59	637.53	637.35	636.33	636.64	635.64
Q3	671.17	671.72	672.11	671.73	673.61	672.96	675.81	675.23	676.86	676.18
Maximum	1952.31	1578.70	917.68	3366.30	1822.76	1820.42	1913.78	2016.21	2106.63	2888.50

n: Number of observations, G_{mean}: Geometric mean, GSD: Geometric standard deviation, GCV%: Geometric coefficient of variation, G_{mean} 95% CI: 95% confidence interval of the geometric mean, A_{mean}: Arithmetic mean, SD: Standard deviation, SD: Standard error, CV%: Coefficient of variation, A_{mean} 95% CI: 95% confidence interval of the arithmetic mean, Q1: 1st (25%) quartile, Q3: 3rd (75%) quartile

Table S.7. Descriptive Statistics of C_{max} (μg/L) Derived from Simulated True Bioinequivalent Studies, with Variability in k_a

	30% IIV 0% IOV		30% IIV 10% IOV		30% IIV 20% IOV		30% IIV 30% IOV		0% IIV 45% IOV	
	Reference	Test	Reference	Test	Reference	Test	Reference	Test	Reference	Test
n	21000	21000	21000	21000	21000	21000	21000	21000	21000	21000
G_{Mean}	638.29	455.45	637.6	454.62	636.13	454.64	633.79	451.39	633.3	452.01
GSD	1.08	1.15	1.09	1.16	1.09	1.18	1.1	1.21	1.11	1.22
GCV%	7.9	14.1	8.3	14.8	8.7	16.3	9.7	18.9	10.2	19.7
G_{mean} 95% CI	637.61 – 638.97	454.59 – 456.31	636.89 – 638.31	453.72 – 455.53	635.39 – 636.87	453.65 – 455.64	632.96 – 634.62	450.25 – 452.54	632.44 – 634.17	450.82 – 453.21
A_{Mean}	640.28	459.9	639.8	459.63	638.52	460.57	636.71	459.14	636.51	460.63
SD	51.24	64.42	54.6	71.77	55.87	73.94	60.59	83.27	63.16	93.43
SE	0.35	0.44	0.38	0.50	0.39	0.51	0.42	0.57	0.44	0.64
CV%	8.0	14.0	8.5	15.6	8.7	16.1	9.5	18.1	9.9	20.3
Variance	2625.34	4149.59	2980.64	5151.53	3121.12	5467.43	3671.6	6934.46	3988.94	8728.87
A_{mean} 95% CI	639.59 – 640.97	459.03 – 460.77	639.06 – 640.54	458.66 – 460.60	637.76 – 639.27	459.57 – 461.57	635.89 – 637.53	458.01 – 460.26	635.65 – 637.36	459.37 – 461.89
Minimum	455.13	243.25	425.36	232.53	402.25	234.63	361.4	166.77	292.94	158.82
Q1	605.99	415.61	604.57	413.93	602.66	409.6	598.07	401.11	597.11	400.26
Median	637.77	458.71	636.98	459.17	636.79	459.96	636.68	459.16	636.96	460.17
Q3	672.12	502.55	672.01	503.28	672.48	510.44	675.38	516.6	676.61	520.3
Maximum	2081.16	1746.56	1972.37	3075.28	1936.51	1830.62	911.18	1704.53	1399.83	4299.59

n: Number of observations, G_{mean}: Geometric mean, GSD: Geometric standard deviation, GCV%: Geometric coefficient of variation, G_{mean} 95% CI: 95% confidence interval of the geometric mean, A_{mean}: Arithmetic mean, SD: Standard deviation, SD: Standard error, CV%: Coefficient of variation, A_{mean} 95% CI: 95% confidence interval of the arithmetic mean, Q1: 1st (25%) quartile, Q3: 3rd (75%) quartile

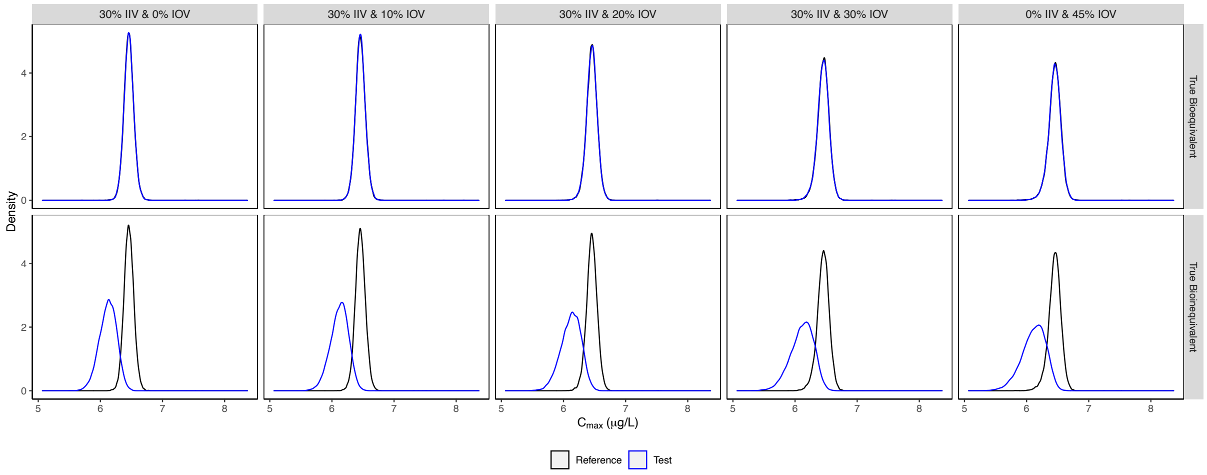

Figure S.20. Distribution of *Ln*-Transformed C_{max}, Derived from Simulations for Studies with Variability in k_a

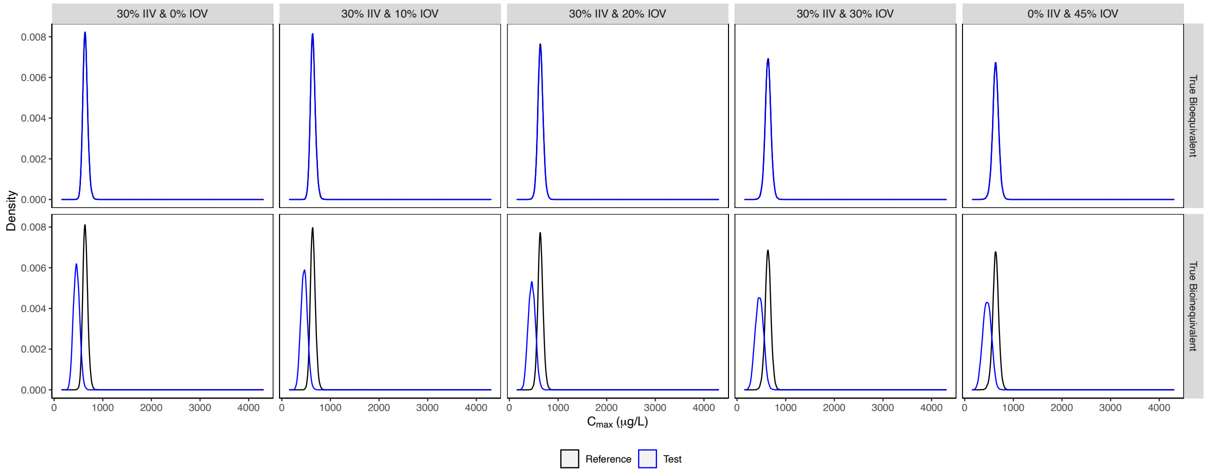

Figure S.21. Distribution of Untransformed C_{max}, Derived from Simulations for Studies with Variability in k_a

Table S.8. Descriptive Statistics of t_{max} (h) Derived from Simulated True Bioequivalent Studies, with Variability in k_a

	30% IIV 0% IOV		30% IIV 10% IOV		30% IIV 20% IOV		30% IIV 30% IOV		0% IIV 45% IOV	
	**Reference**	**Test**	**Reference**	**Test**	**Reference**	**Test**	**Reference**	**Test**	**Reference**	**Test**
n	21000	21000	21000	21000	21000	21000	21000	21000	21000	21000
Minimum	0.50	0.50	0.50	0.50	0.50	0.50	0.50	0.50	0.50	0.25
Q1	1.75	1.75	1.75	1.75	1.75	1.75	1.75	1.75	1.75	1.75
Median	2.25	2.25	2.25	2.25	2.25	2.25	2.25	2.25	2.25	2.25
Q3	2.75	2.75	2.75	2.75	2.75	2.75	2.75	2.75	2.75	2.75
Maximum	6.00	6.00	6.00	8.00	6.00	6.00	6.00	6.00	6.00	8.00

n: Number of observations, Q1: 1st (25%) quartile, Q3: 3rd (75%) quartile

Table S.9. Descriptive Statistics of t_{max} (h) Derived from Simulated True Bioinequivalent Studies, with Variability in k_a

	30% IIV 0% IOV		30% IIV 10% IOV		30% IIV 20% IOV		30% IIV 30% IOV		0% IIV 45% IOV	
	**Reference**	**Test**	**Reference**	**Test**	**Reference**	**Test**	**Reference**	**Test**	**Reference**	**Test**
n	21000	21000	21000	21000	21000	21000	21000	21000	21000	21000
Minimum	0.50	1.50	0.50	1.50	0.25	1.00	0.50	1.00	0.50	1.00
Q1	1.75	3.25	1.75	3.25	1.75	3.00	1.75	3.00	1.75	3.00
Median	2.25	3.50	2.25	3.50	2.25	3.50	2.25	3.50	2.25	3.50
Q3	2.75	4.00	2.75	4.00	2.75	4.00	2.75	4.00	2.75	4.00
Maximum	4.00	12.00	6.00	12.00	6.00	12.00	6.00	12.00	8.00	12.00

n: Number of observations, Q1: 1st (25%) quartile, Q3: 3rd (75%) quartile

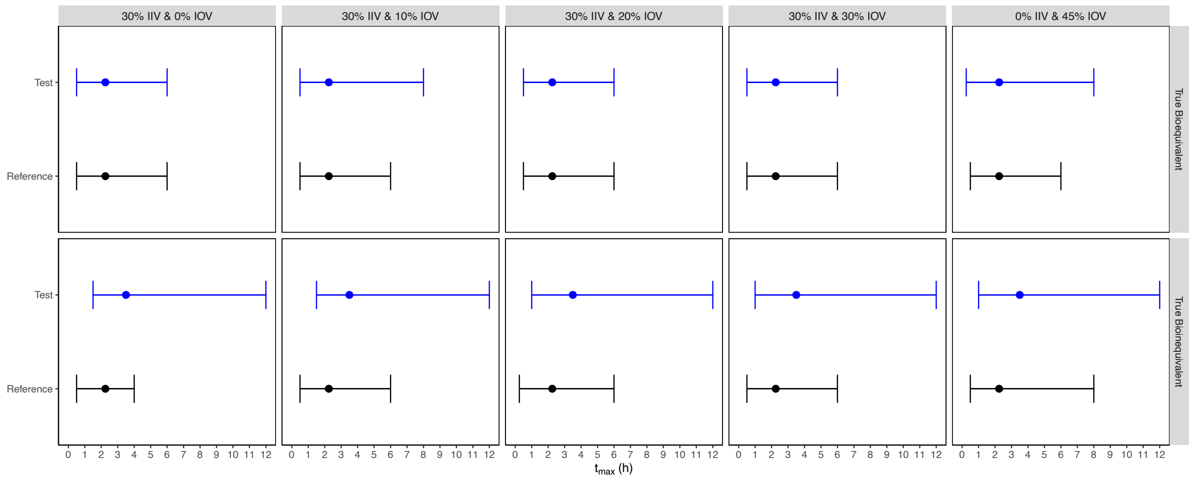

Figure S.22. Distribution of Untransformed t_{max}, Derived from Simulations for Studies with Variability in k_a

Table S.10. Descriptive Statistics of AUC_{0-t} (µg.h/L) Derived from Simulated True Bioequivalent Studies, with Variability in k_a

	30% IIV 0% IOV		30% IIV 10% IOV		30% IIV 20% IOV		30% IIV 30% IOV		0% IIV 45% IOV	
	Reference	Test	Reference	Test	Reference	Test	Reference	Test	Reference	Test
n	21000	21000	21000	21000	21000	21000	21000	21000	21000	21000
G_{Mean}	4945.47	4948.26	4947.90	4946.83	4948.63	4945.41	4946.10	4946.55	4944.13	4945.06
GSD	1.03	1.03	1.03	1.03	1.03	1.03	1.03	1.03	1.03	1.04
GCV%	3.2	3.3	3.2	3.4	3.2	3.3	3.2	3.3	3.3	3.6
G_{mean} 95% CI	4943.30 – 4947.64	4946.03 – 4950.49	4945.79 – 4950.02	4944.53 – 4949.13	4946.48 – 4950.77	4943.23 – 4947.60	4943.94 – 4948.26	4944.36 – 4948.74	4941.95 – 4946.31	4942.67 – 4947.45
A_{Mean}	4948.15	4951	4950.37	4950.14	4951.18	4948.12	4948.72	4949.23	4946.82	4948.41
SD	170.17	166.25	156.18	230.45	161.08	171.06	164.99	166.33	169.25	212.58
SE	1.17	1.15	1.08	1.59	1.11	1.18	1.14	1.15	1.17	1.47
CV%	3.4	3.4	3.2	4.7	3.3	3.5	3.3	3.4	3.4	4.3
Variance	28958.89	27639.68	24391.74	53107.75	25946.03	29261.7	27222.47	27664.8	28646.92	45190.39
A_{mean} 95% CI	4945.84 – 4950.45	4948.75 – 4953.25	4948.25 – 4952.48	4947.02 – 4953.25	4949.01 – 4953.36	4945.80 – 4950.43	4946.49 – 4950.95	4946.98 – 4951.48	4944.53 – 4949.11	4945.53 – 4951.28
Minimum	4349.7	1530.35	4351.33	4264.03	4011.44	4391.94	4374.88	4376.71	4367.09	1181.6
Q1	4841.35	4843.5	4845.58	4843.28	4844.6	4840.08	4842.96	4841.35	4837.92	4839.16
Median	4944.99	4947.21	4947.77	4947.25	4947.47	4944.36	4946.47	4946.77	4943.48	4945.95
Q3	5050.85	5052.54	5052.88	5052.03	5054.19	5052.23	5051.75	5052.83	5050.98	5051.61
Maximum	14677.41	11840.03	5587.45	27708.92	10499.32	14694.94	12416.26	11430.87	13925.36	23711.35

n: Number of observations, G_{mean}: Geometric mean, GSD: Geometric standard deviation, GCV%: Geometric coefficient of variation, G_{mean} 95% CI: 95% confidence interval of the geometric mean, A_{mean}: Arithmetic mean, SD: Standard deviation, SD: Standard error, CV%: Coefficient of variation, A_{mean} 95% CI: 95% confidence interval of the arithmetic mean, Q1: 1st (25%) quartile, Q3: 3rd (75%) quartile

Table S.11. Descriptive Statistics of AUC_{0-t} (µg.h/L) derived from Simulated True Bioinequivalent Studies, with Variability in k_a

	30% IIV 0% IOV		30% IIV 10% IOV		30% IIV 20% IOV		30% IIV 30% IOV		0% IIV 45% IOV	
	Reference	Test	Reference	Test	Reference	Test	Reference	Test	Reference	Test
n	21000	21000	21000	21000	21000	21000	21000	21000	21000	21000
G_{Mean}	4949.1	4821.22	4950.1	4817.11	4945.35	4814.03	4943.85	4797.67	4944.72	4793.35
GSD	1.03	1.04	1.03	1.05	1.03	1.05	1.03	1.05	1.03	1.06
GCV%	3.2	4.4	3.4	4.8	3.4	4.7	3.2	5.1	3.3	5.7
G_{mean} 95% CI	4946.96 – 4951.24	4818.35 – 4824.09	4947.82 – 4952.38	4813.99 – 4820.23	4943.11 – 4947.60	4810.98 – 4817.09	4941.73 – 4945.96	4794.39 – 4800.95	4942.53 – 4946.91	4789.65 – 4797.04
A_{Mean}	4951.63	4826.14	4953.1	4823.73	4948.3	4819.65	4946.32	4803.78	4947.4	4802.11
SD	159	238.68	184.9	349.74	188.44	257.89	156.52	247.16	165.66	398.9
SE	1.1	1.65	1.28	2.41	1.3	1.78	1.08	1.71	1.14	2.75
CV%	3.2	4.9	3.7	7.3	3.8	5.4	3.2	5.1	3.3	8.3
Variance	25280.5	56968.14	34186.94	122316.71	35509.68	66508.55	24496.97	61087.33	27442.23	159119.67
A_{mean} 95% CI	4949.48 – 4953.79	4822.91 – 4829.36	4950.60 – 4955.60	4819.00 – 4828.46	4945.75 – 4950.85	4816.16 – 4823.13	4944.21 – 4948.44	4800.44 – 4807.12	4945.16 – 4949.64	4796.72 – 4807.51
Minimum	4386.37	3892.08	4388.87	3959.48	4365.77	3388.41	4353.3	1220.16	3633.33	653.17
Q1	4844.52	4694.67	4845.31	4686.99	4840.81	4684.77	4840.6	4669.68	4839.28	4666.44
Median	4949.44	4823.64	4948.48	4823.95	4944.77	4822.61	4942.84	4817.02	4944.03	4814.92
Q3	5055.42	4958.62	5054.97	4954.73	5052.6	4956.57	5049.76	4954.1	5053.14	4952.75
Maximum	7929.43	17109.25	12928.25	28676.93	18745.79	18253.05	5606.99	13142.26	11608.71	36515.48

n: Number of observations, G_{mean}: Geometric mean, GSD: Geometric standard deviation, GCV%: Geometric coefficient of variation, G_{mean} 95% CI: 95% confidence interval of the geometric mean, A_{mean}: Arithmetic mean, SD: Standard deviation, SD: Standard error, CV%: Coefficient of variation, A_{mean} 95% CI: 95% confidence interval of the arithmetic mean, Q1: 1st (25%) quartile, Q3: 3rd (75%) quartile

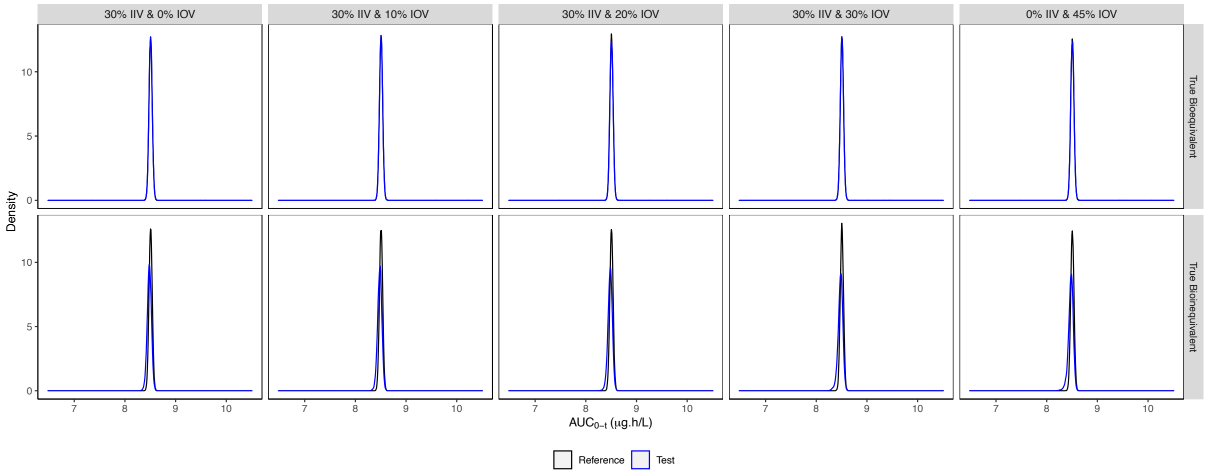

Figure S.23. Distribution of *Ln*-Transformed $AUC_{0\text{-}t}$, Simulated for Studies with Variability in k_a

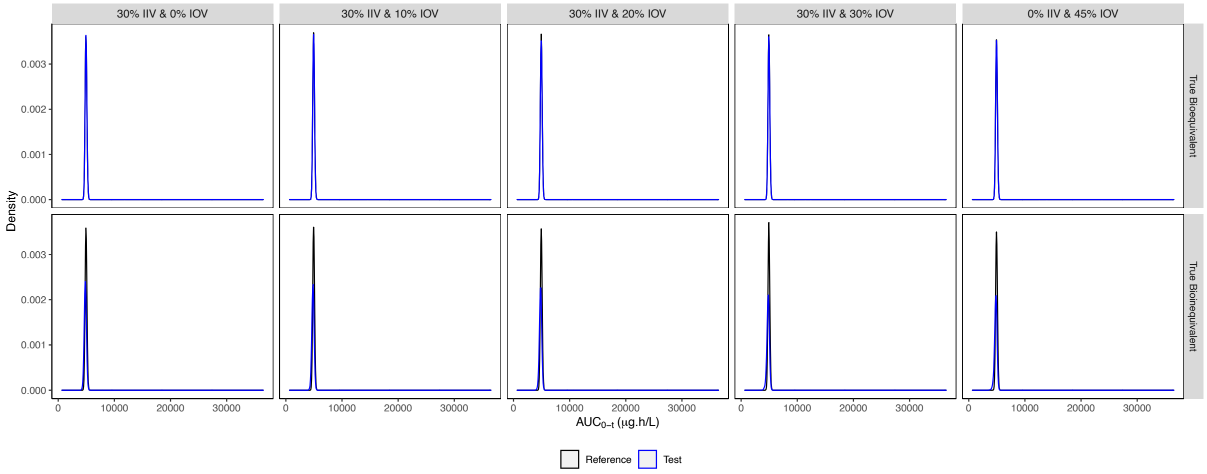

Figure S.24. Distribution of Untransformed $AUC_{0\text{-}t}$, Derived from Simulations for Studies with Variability in k_a

Appendix SA.2.4. Simulated Pilot Studies Results

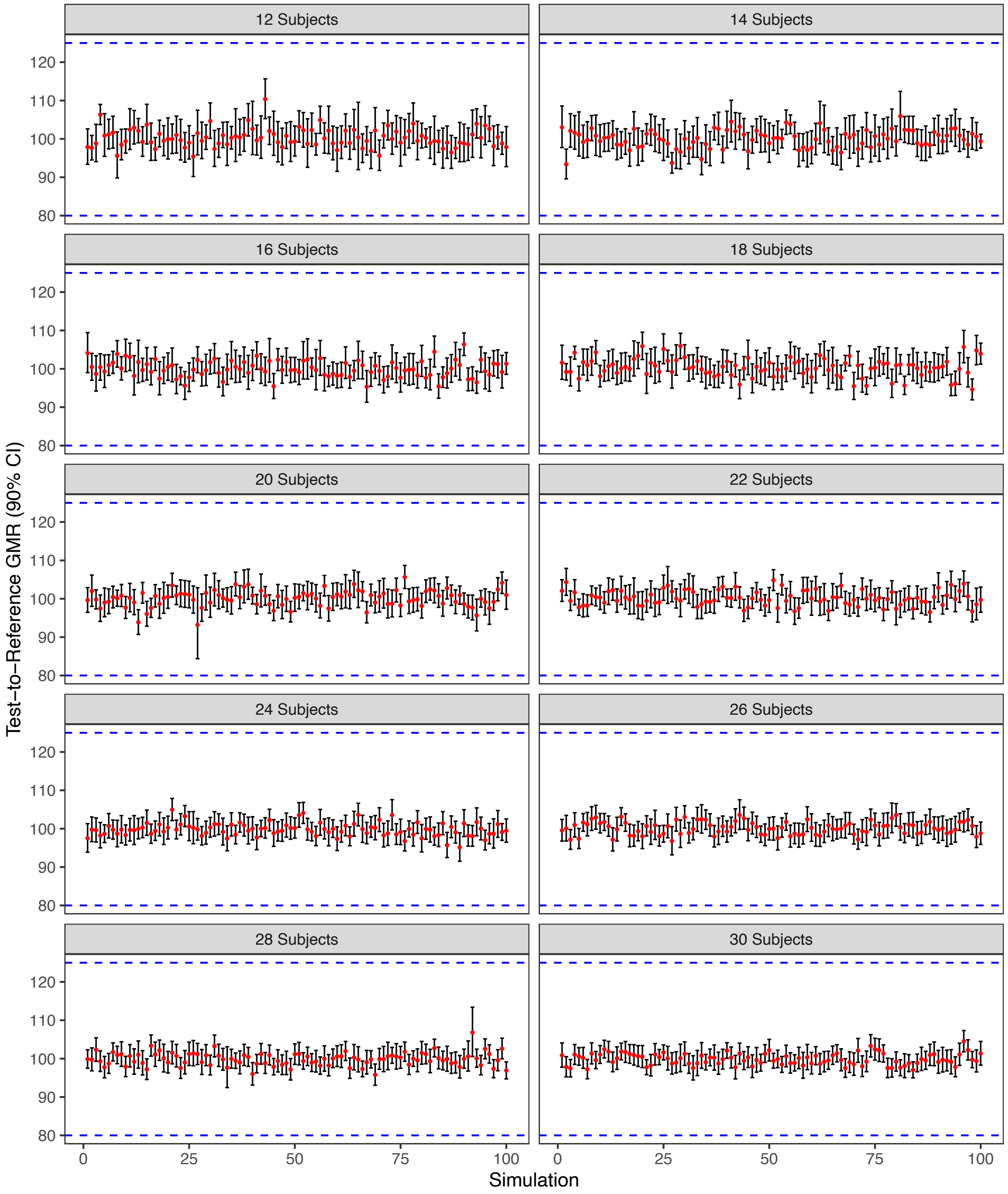

Figure S.25. Average Bioequivalence Analysis Results (Test-to-Reference GMR and Corresponding 90% CI) for C_{max} Derived from True Bioequivalent Simulations with 30% IIV & 0% IOV in k_a

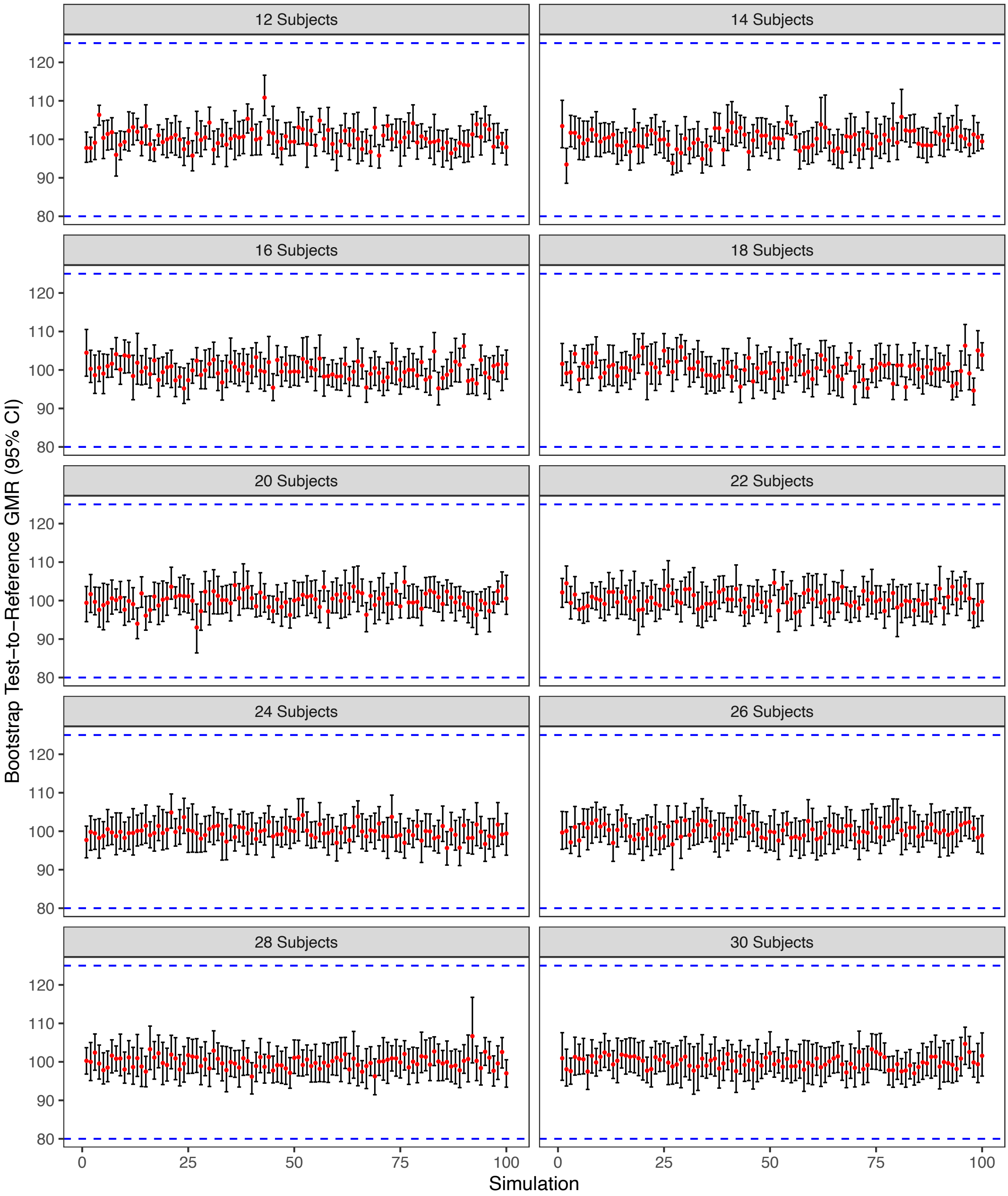

Figure S.26. Bootstrap Bioequivalence Analysis Results (Test-to-Reference GMR and Corresponding 95% CI) for C_{max} Derived from True Bioequivalent Simulations with 30% IIV & 0% IOV in k_a

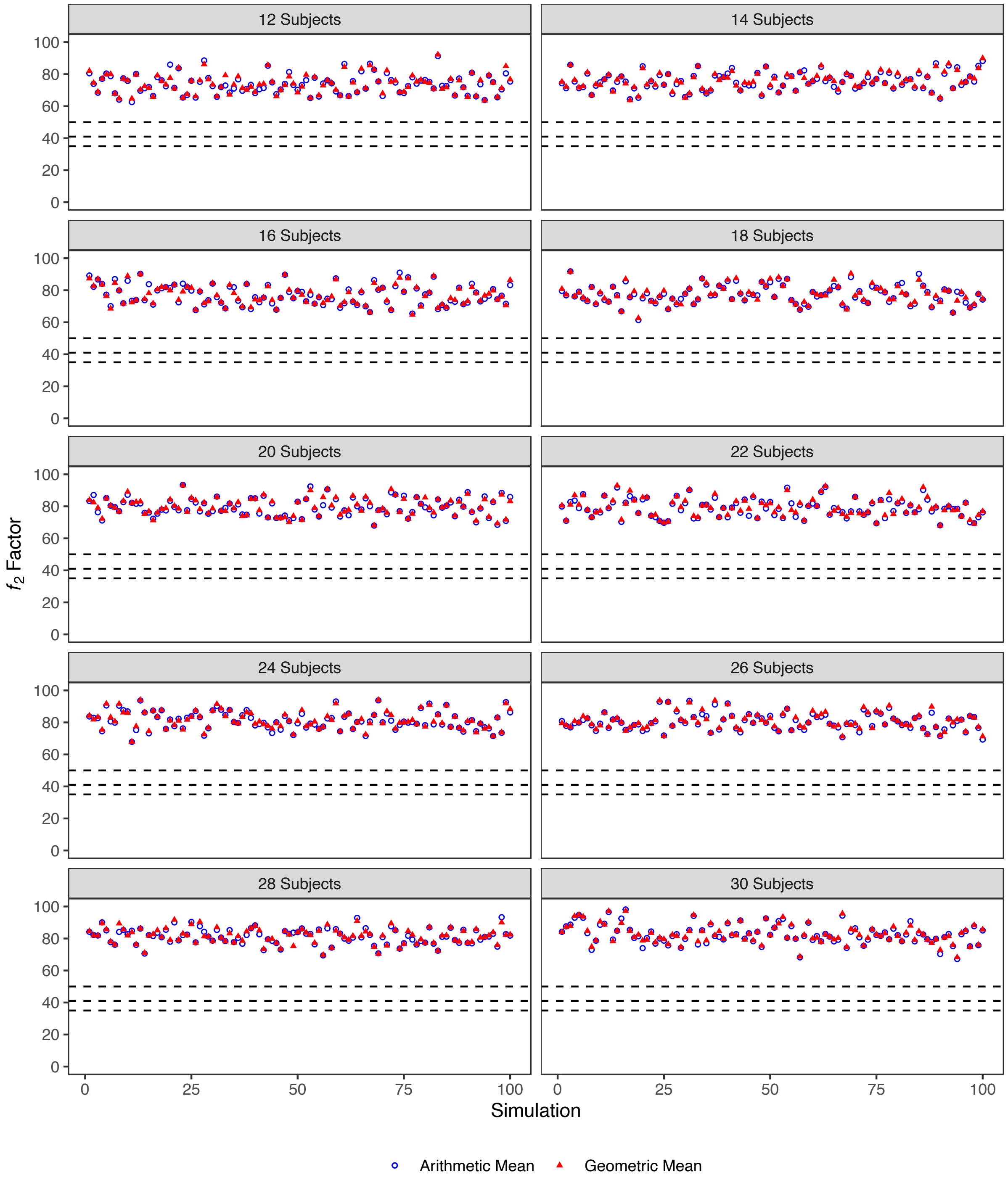

Figure S.27. Similarity f_2 Factor Results, Derived from Arithmetic and Geometric Mean Concentration-Time Profiles Until the Reference C_{max}, Derived from True Bioequivalent Simulations with 30% IIV & 0% IOV in k_a

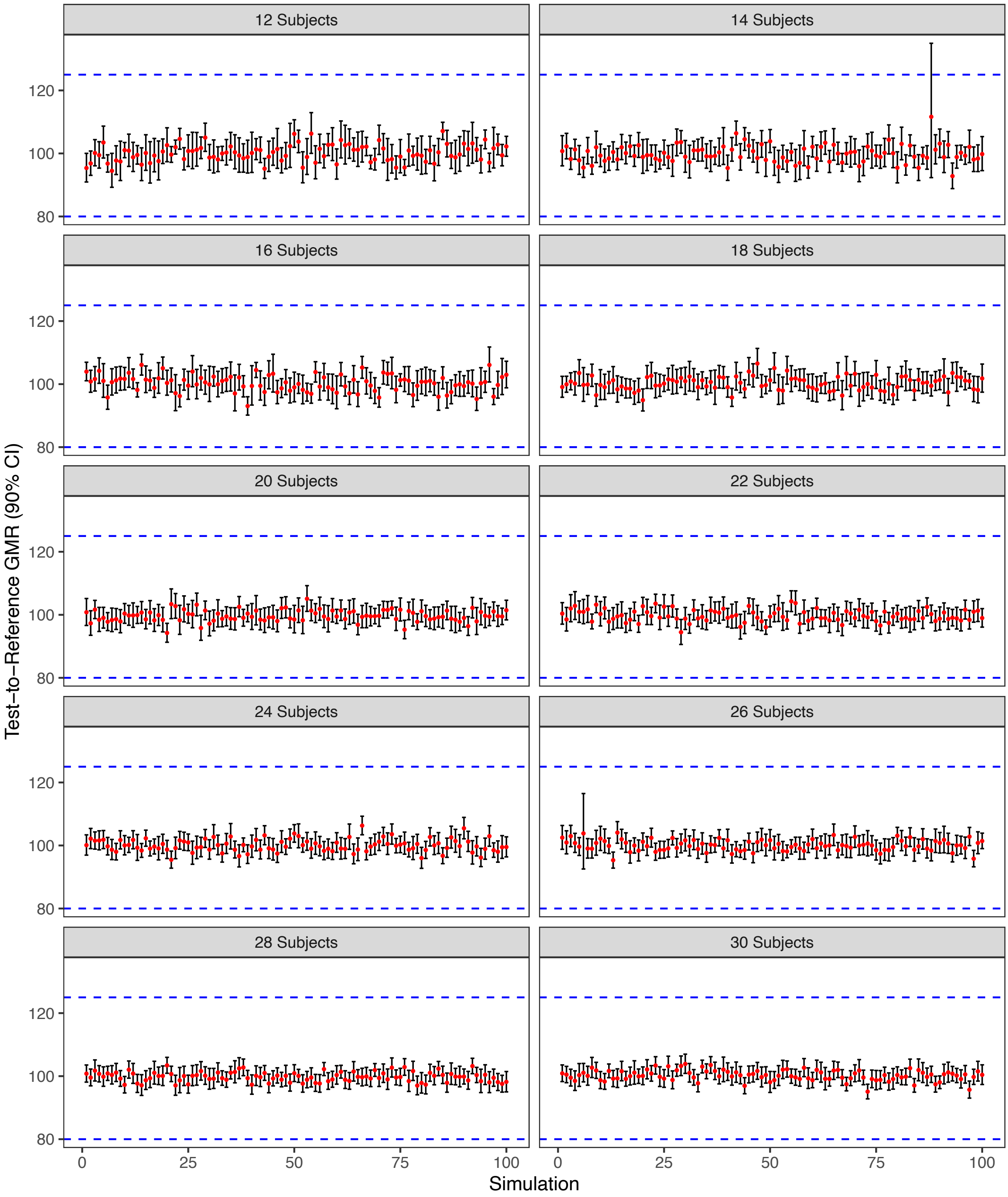

Figure S.28. Average Bioequivalence Analysis Results (Test-to-Reference GMR and Corresponding 90% CI) for C_{max} Derived from True Bioequivalent Simulations with 30% IIV & 10% IOV in k_a

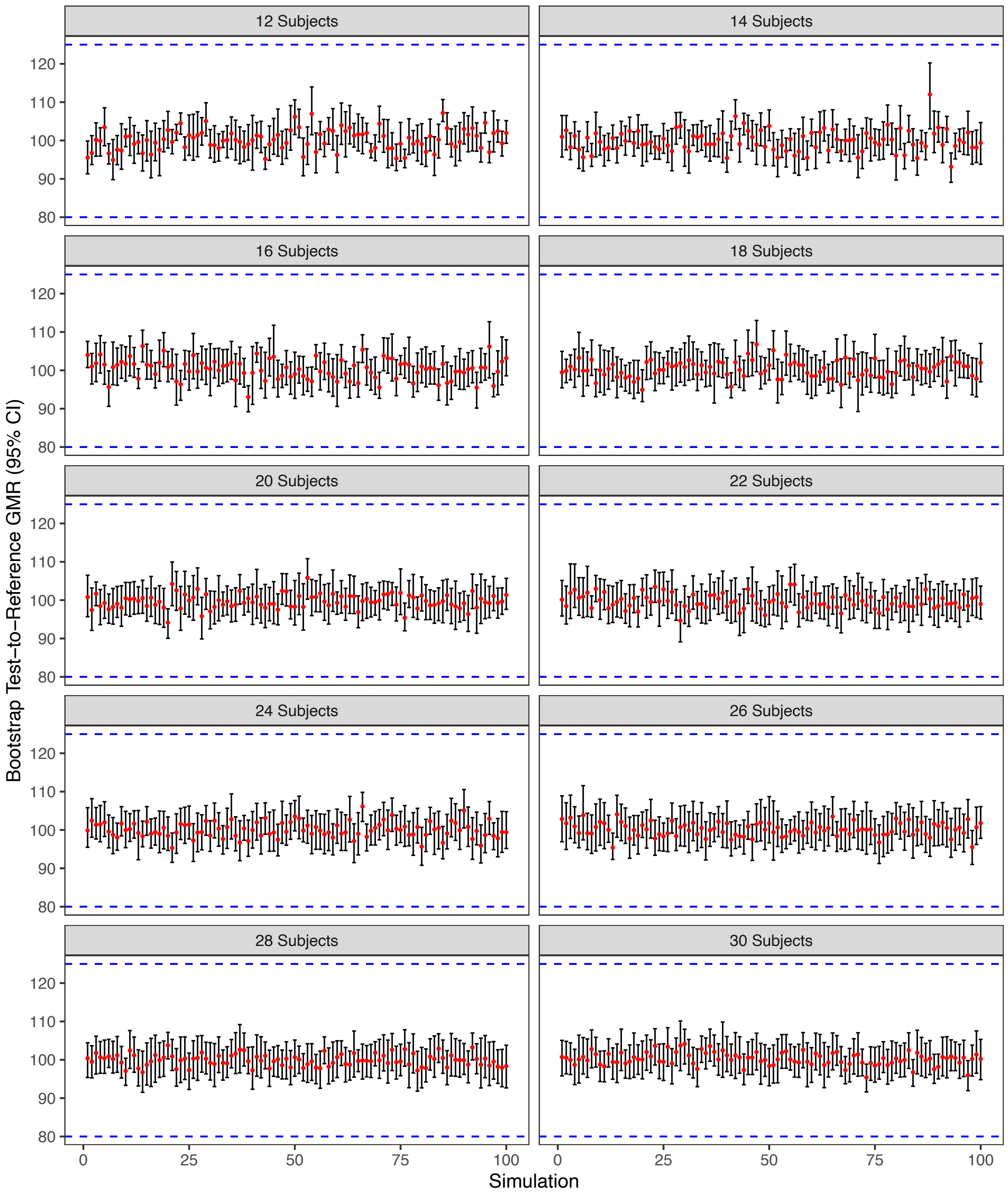

Figure S.29. Bootstrap Bioequivalence Analysis Results (Test-to-Reference GMR and Corresponding 95% CI) for C_{max} Derived from True Bioequivalent Simulations with 30% IIV & 10% IOV in k_a

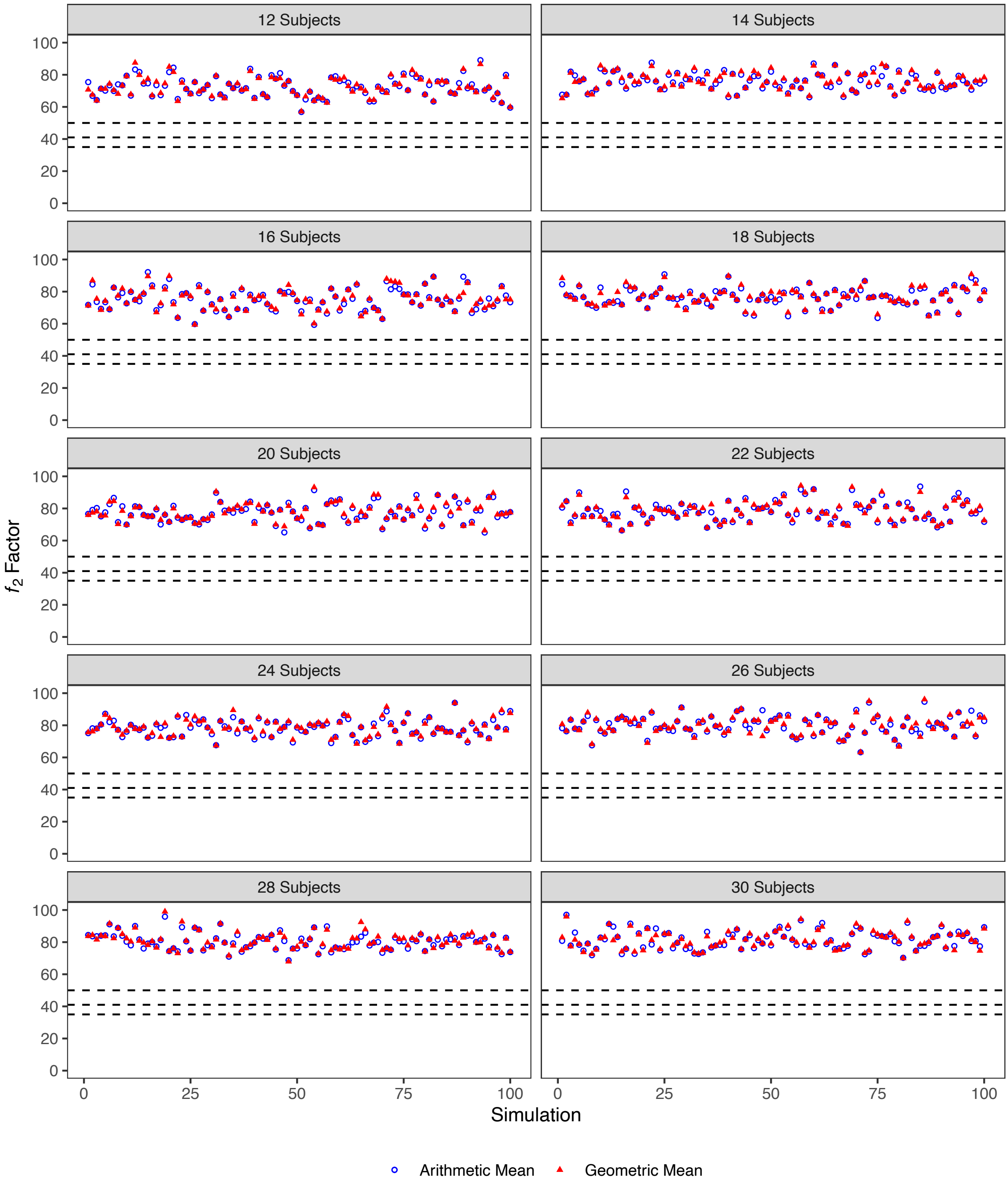

Figure S.30. Similarity f_2 Factor Results, Derived from Arithmetic and Geometric Mean Concentration-Time Profiles Until the Reference C_{max}, Derived from True Bioequivalent Simulations with 30% IIV & 10% IOV in k_a

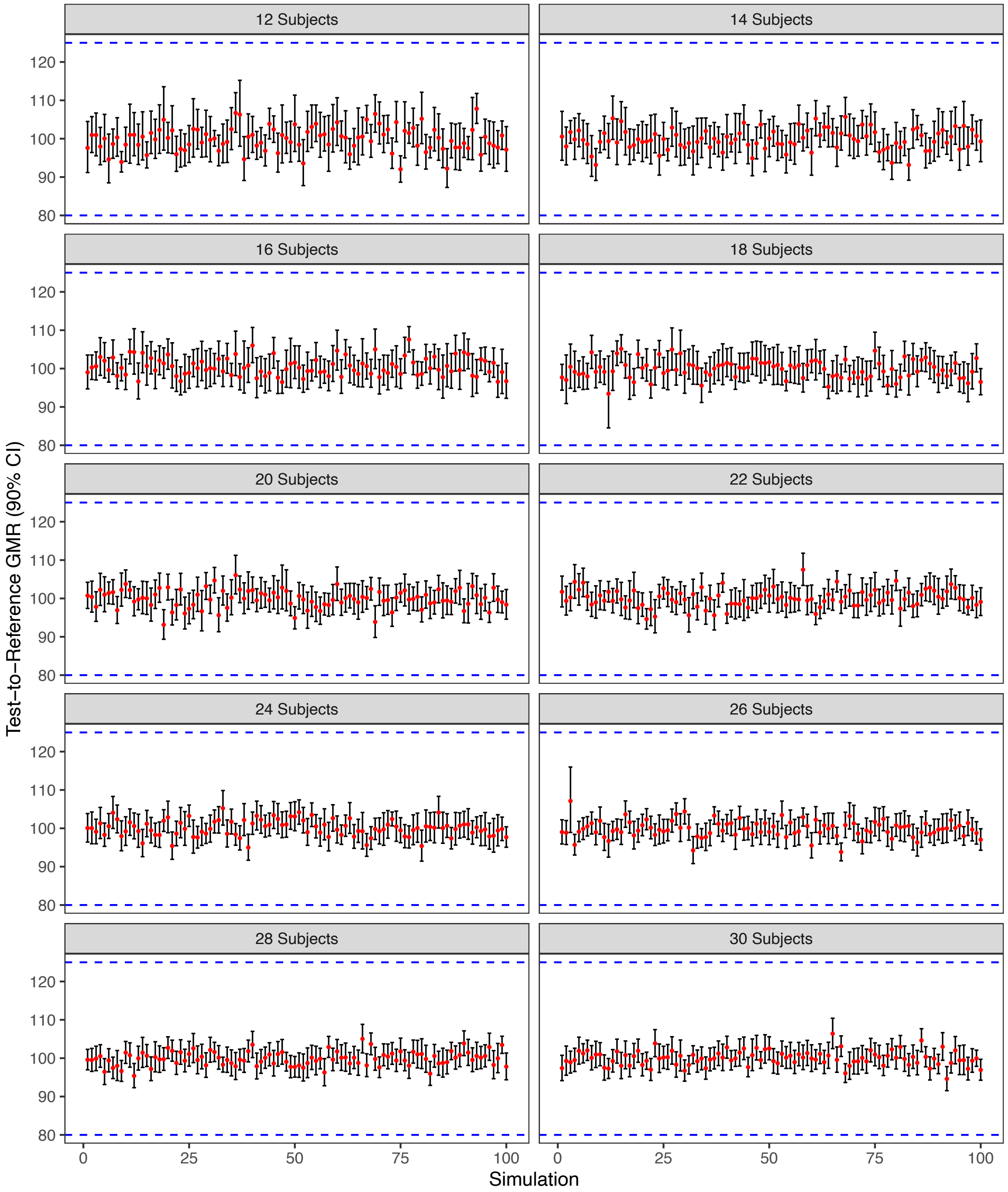

Figure S.31. Average Bioequivalence Analysis Results (Test-to-Reference GMR and Corresponding 90% CI) for C_{max} Derived from True Bioequivalent Simulations with 30% IIV & 20% IOV in k_a

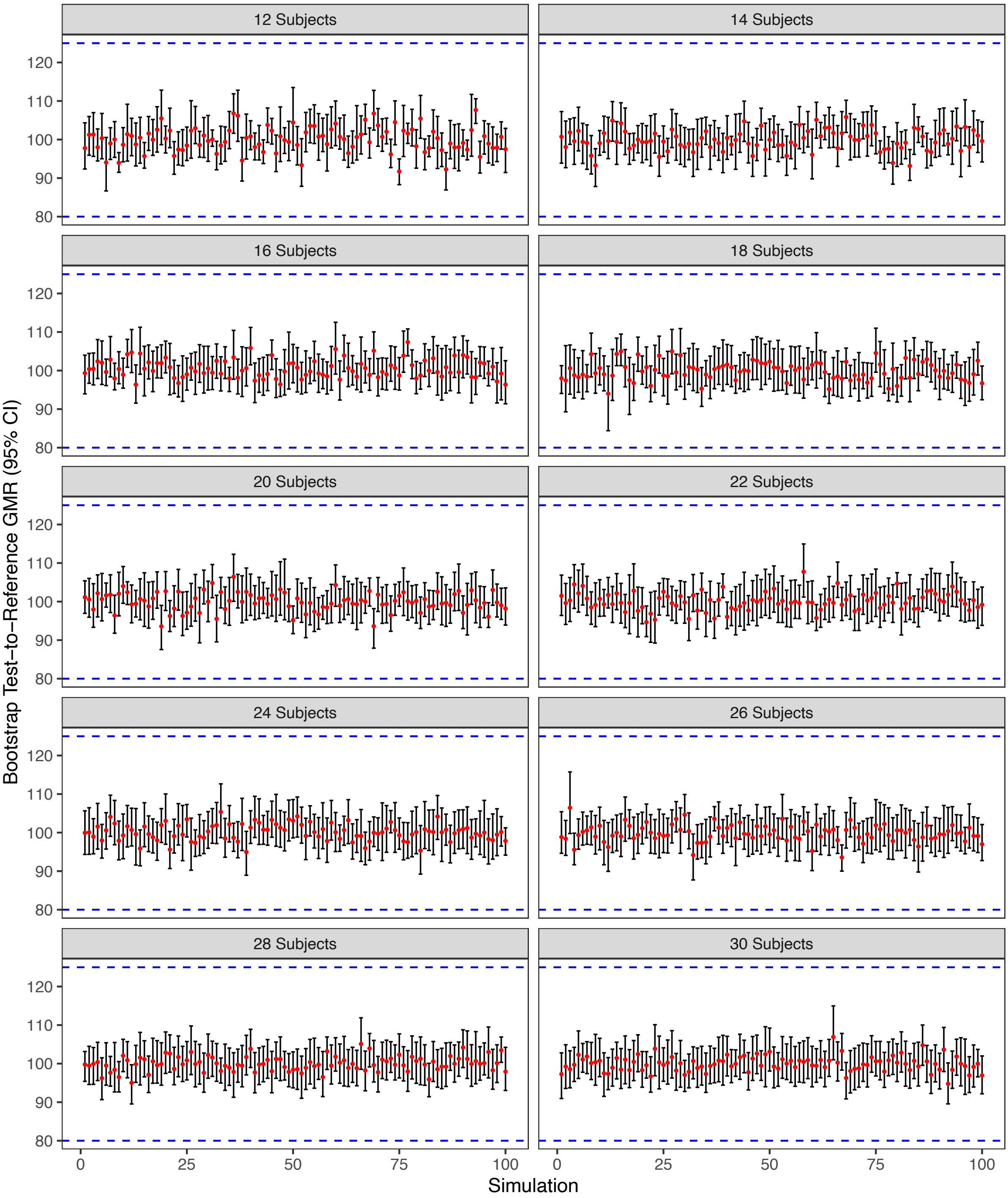

Figure S.32. Bootstrap Bioequivalence Analysis Results (Test-to-Reference GMR and Corresponding 95% CI) for C_{max} Derived from True Bioequivalent Simulations with 30% IIV & 20% IOV in k_a

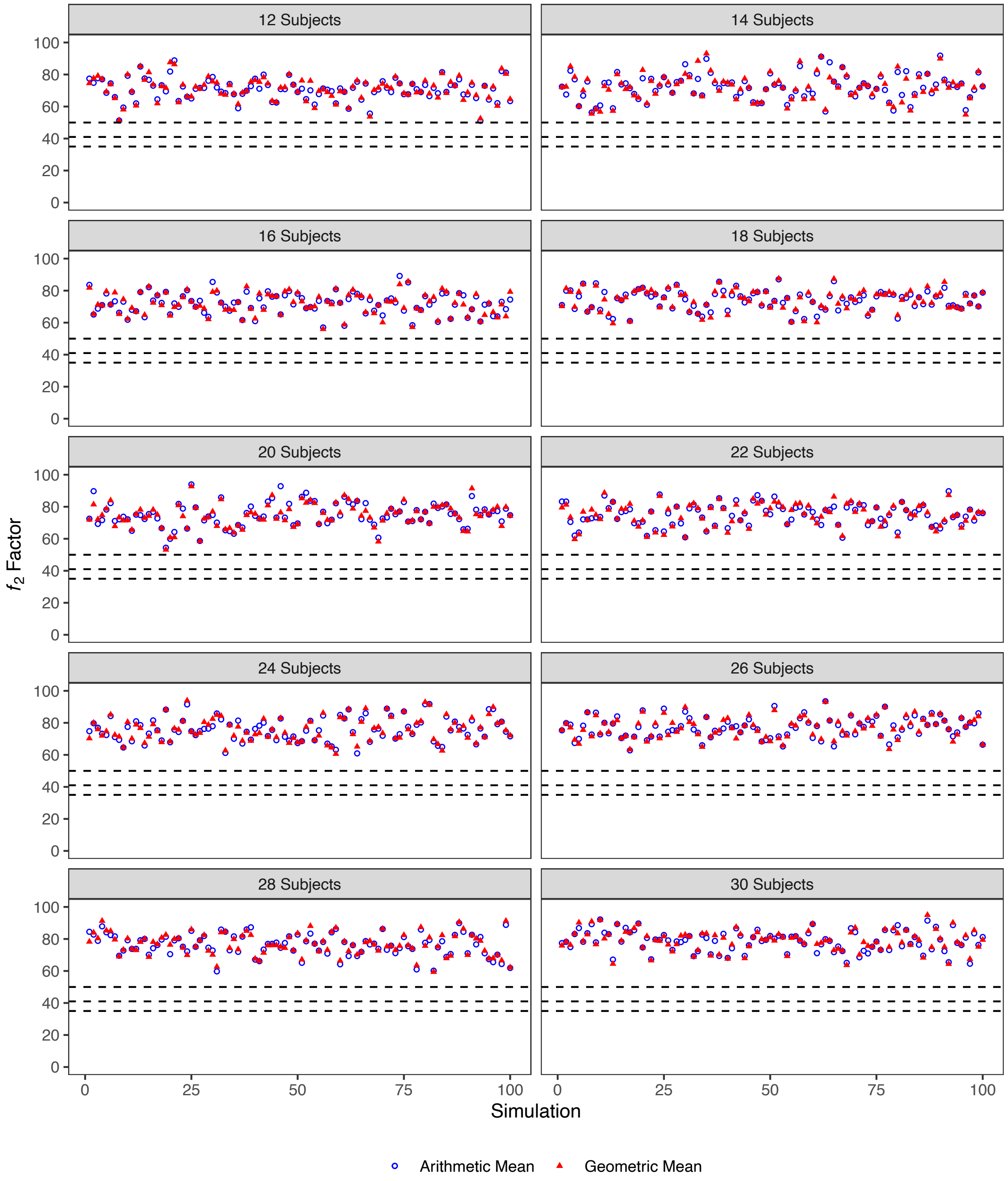

Figure S.33. Similarity f_2 Factor Results, Derived from Arithmetic and Geometric Mean Concentration-Time Profiles Until the Reference C_{max}, Derived from True Bioequivalent Simulations with 30% IIV & 20% IOV in k_a

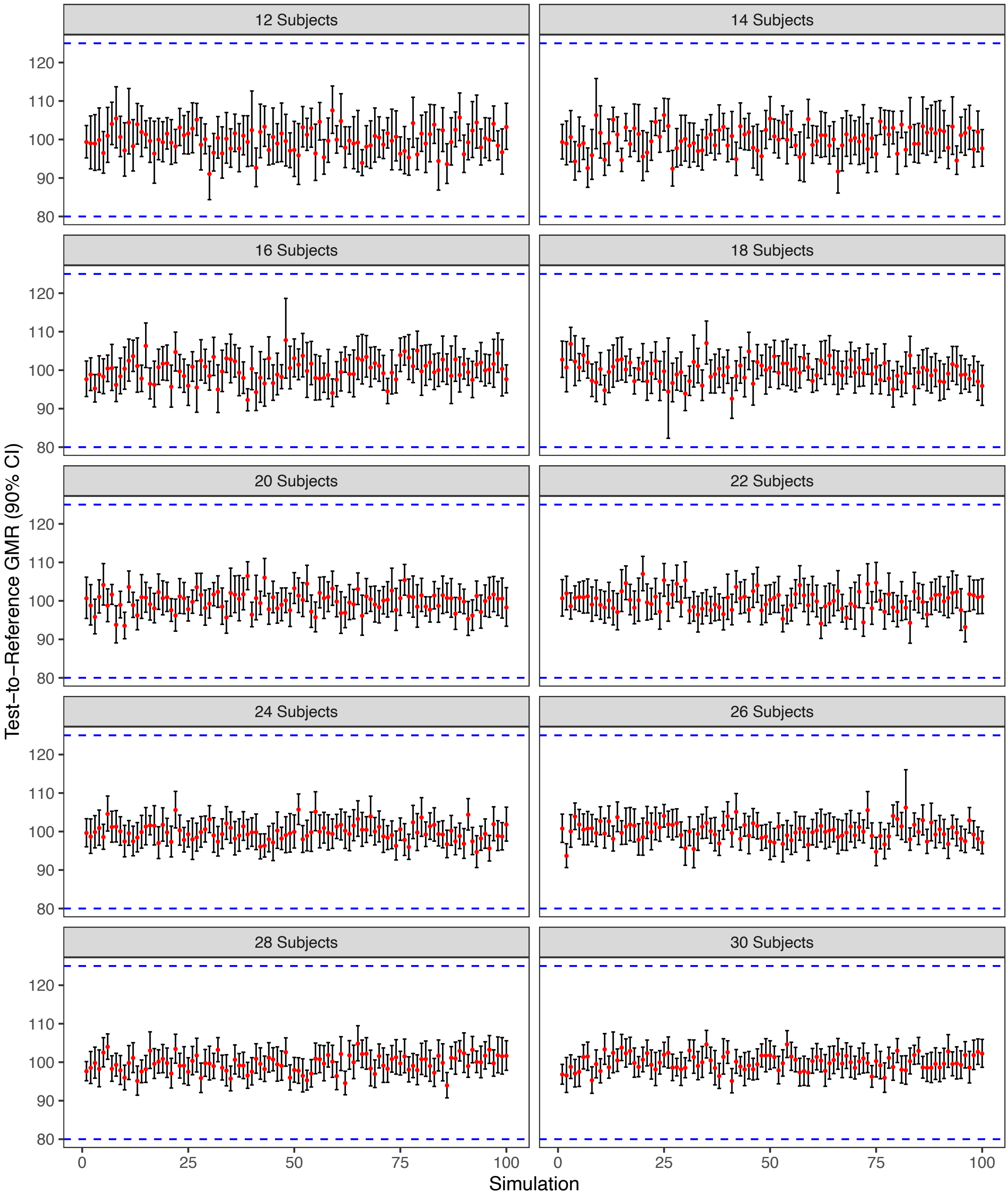

Figure S.34. Average Bioequivalence Analysis Results (Test-to-Reference GMR and Corresponding 90% CI) for C_{max} Derived from True Bioequivalent Simulations with 30% IIV & 30% IOV in k_a

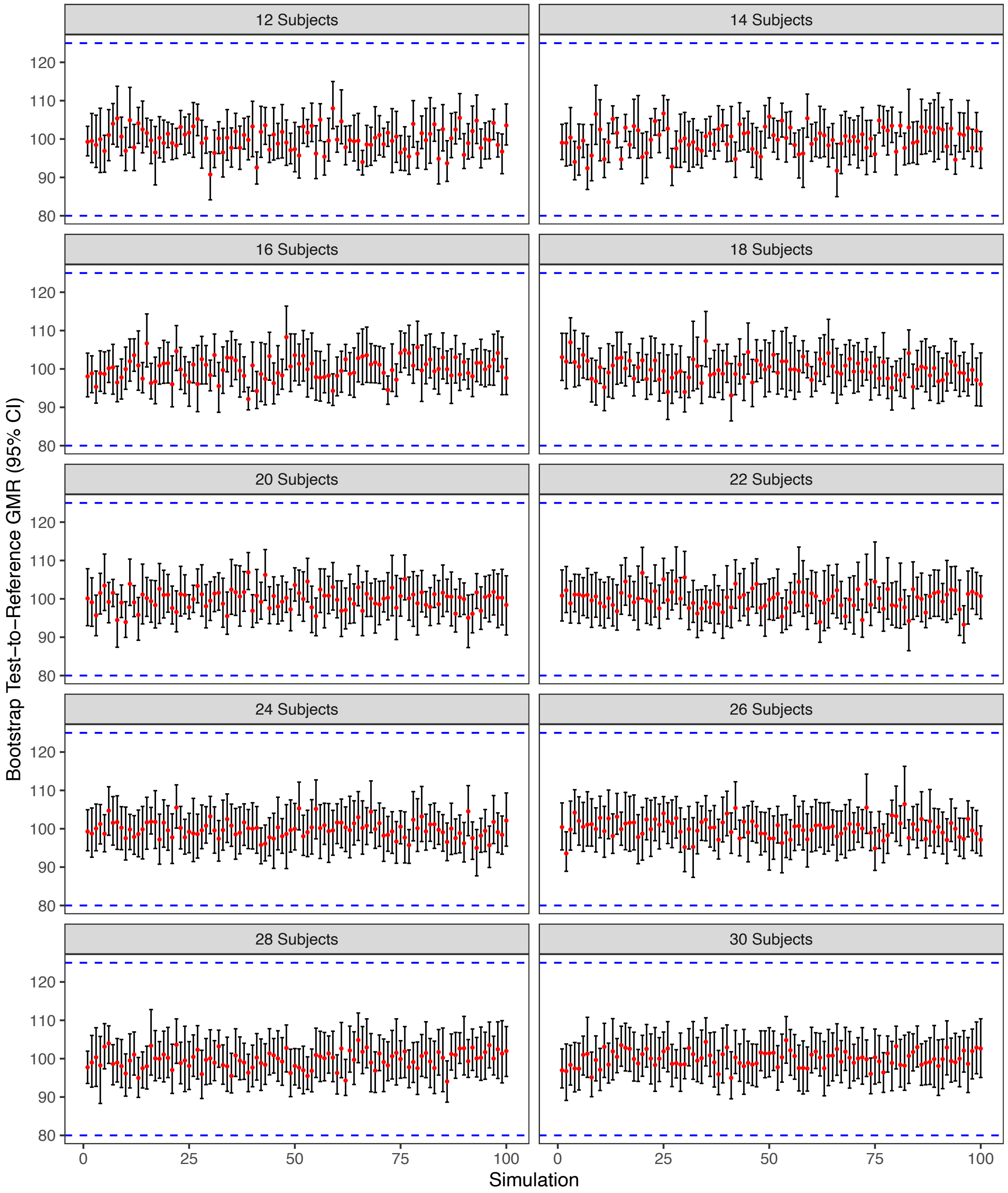

Figure S.35. Bootstrap Bioequivalence Analysis Results (Test-to-Reference GMR and Corresponding 95% CI) for C_{max} Derived from True Bioequivalent Simulations with 30% IIV & 30% IOV in k_a

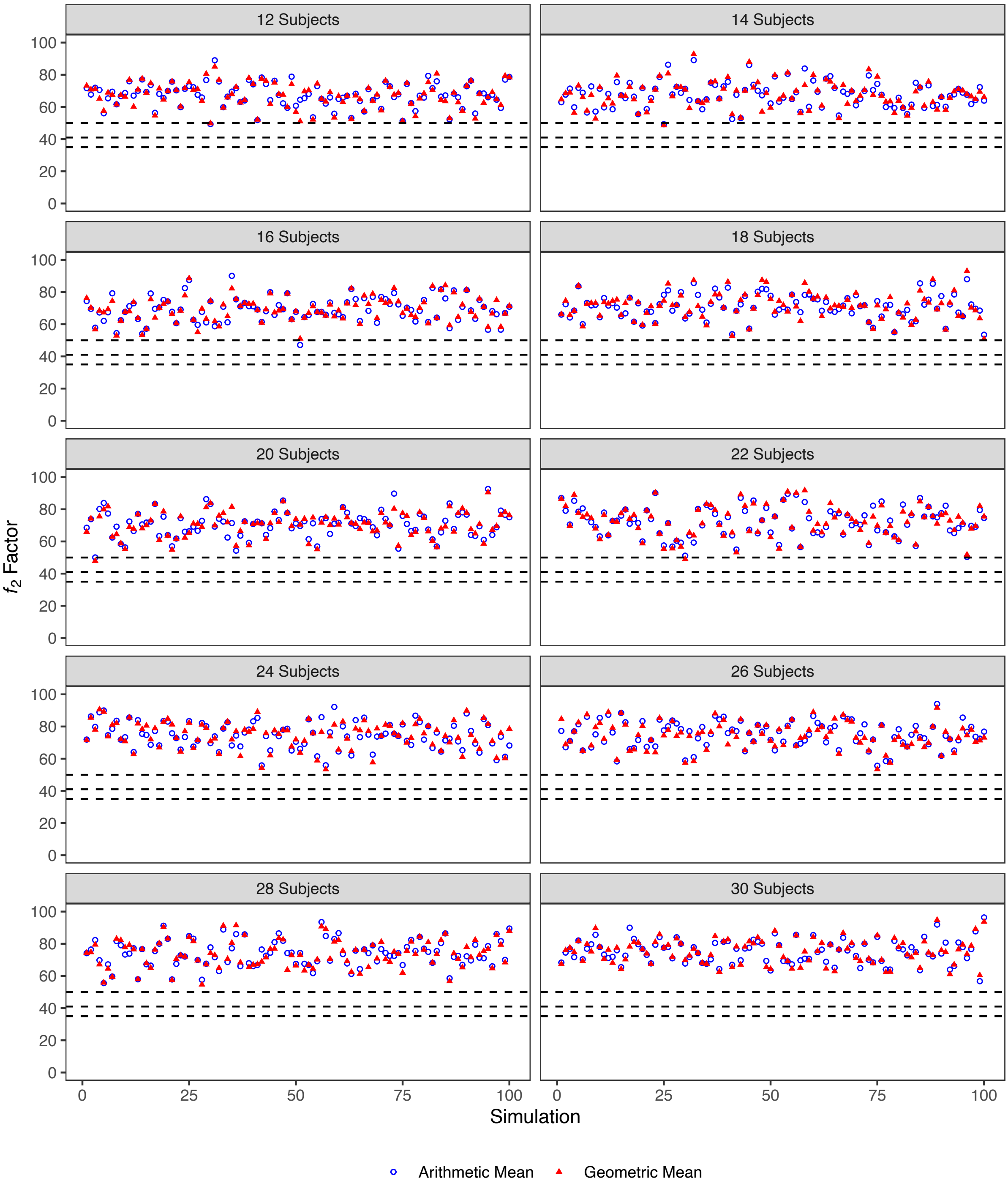

Figure S.36. Similarity f_2 Factor Results, Derived from Arithmetic and Geometric Mean Concentration-Time Profiles Until the Reference C_{max}, Derived from True Bioequivalent Simulations with 30% IIV & 30% IOV in k_a

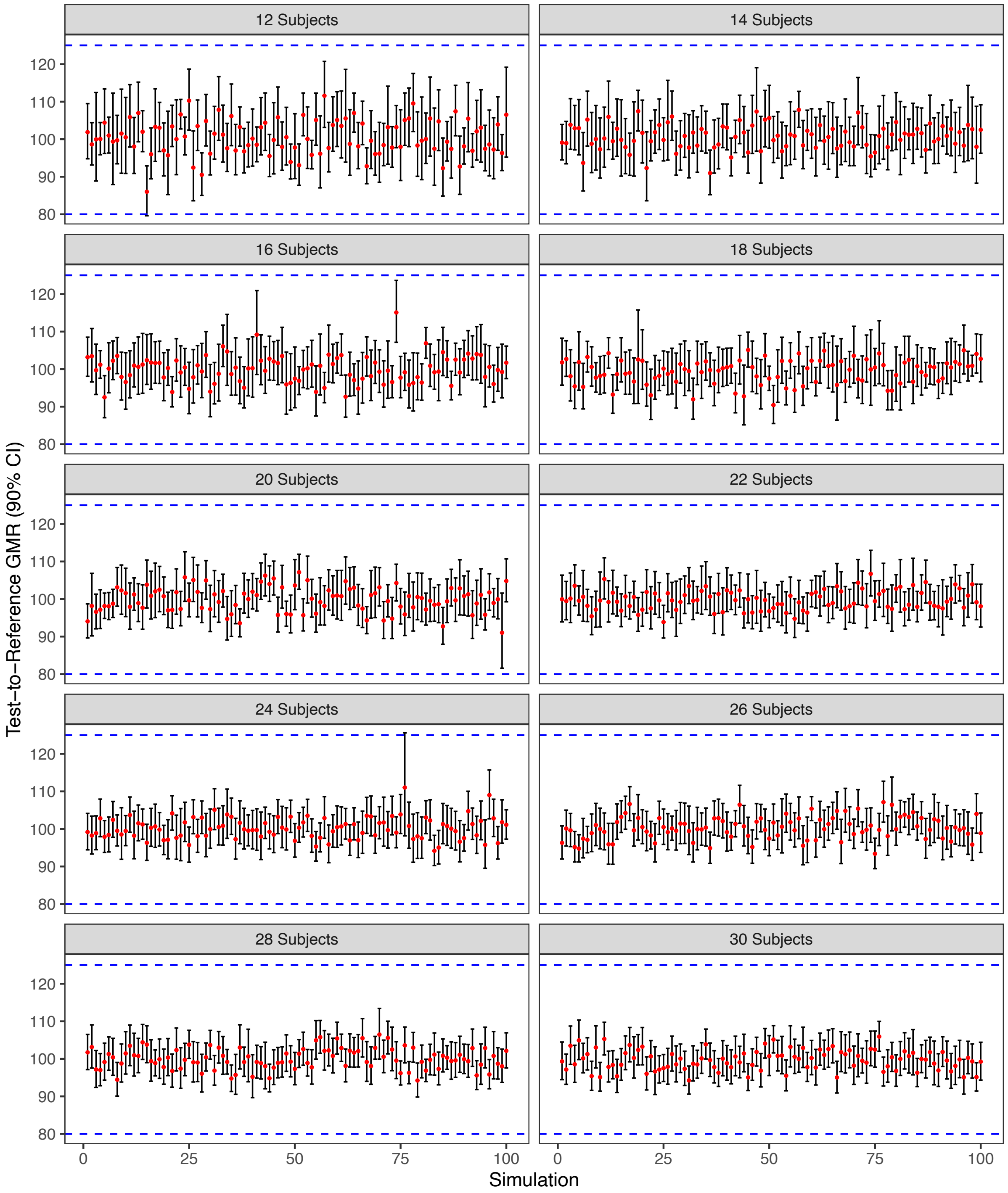

Figure S.37. Average Bioequivalence Analysis Results (Test-to-Reference GMR and Corresponding 90% CI) for C_{max} Derived from True Bioequivalent Simulations with 0% IIV & 45% IOV in k_a

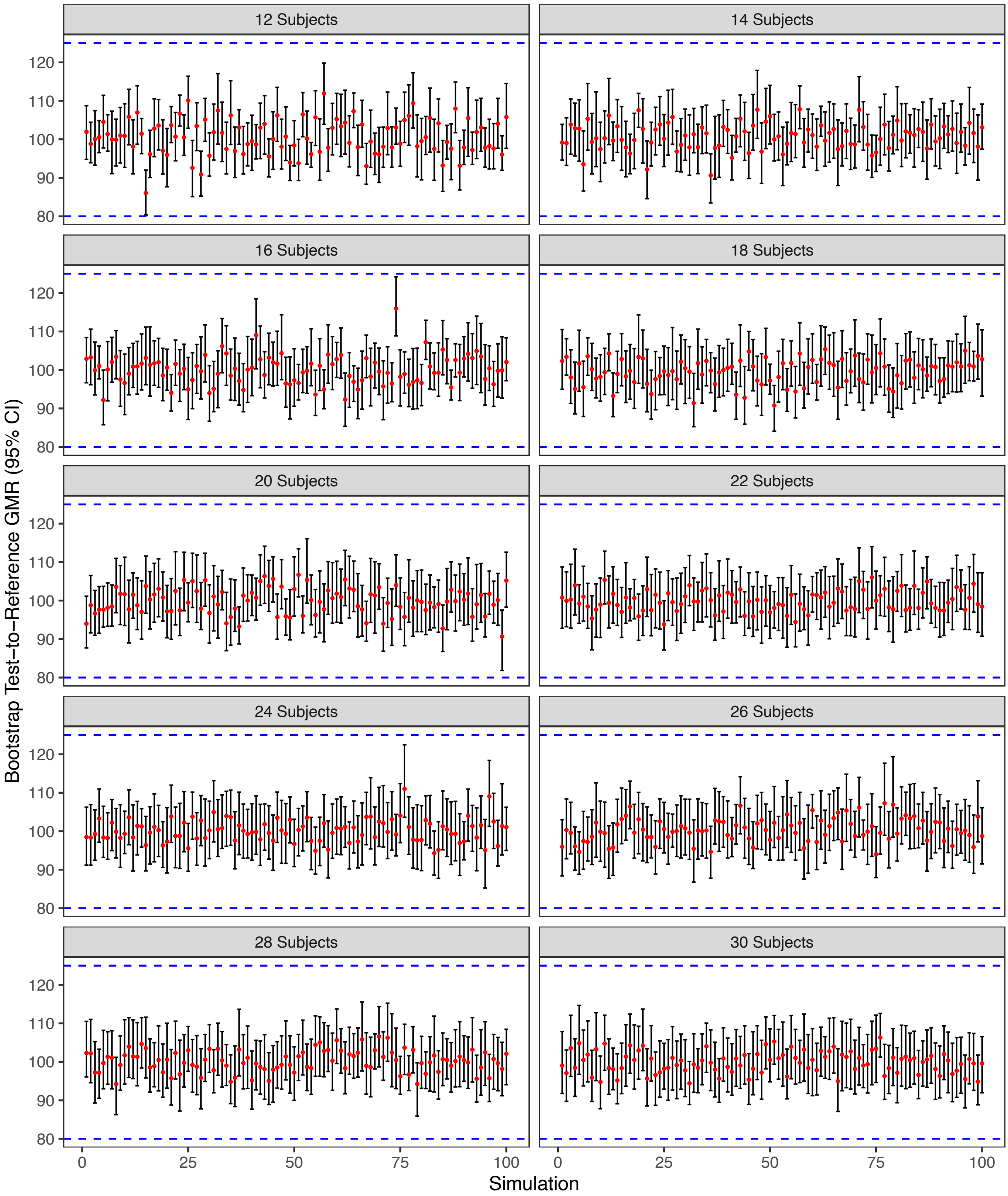

Figure S.38. Bootstrap Bioequivalence Analysis Results (Test-to-Reference GMR and Corresponding 95% CI) for C_{max} Derived from True Bioequivalent Simulations with 0% IIV & 45% IOV in k_a

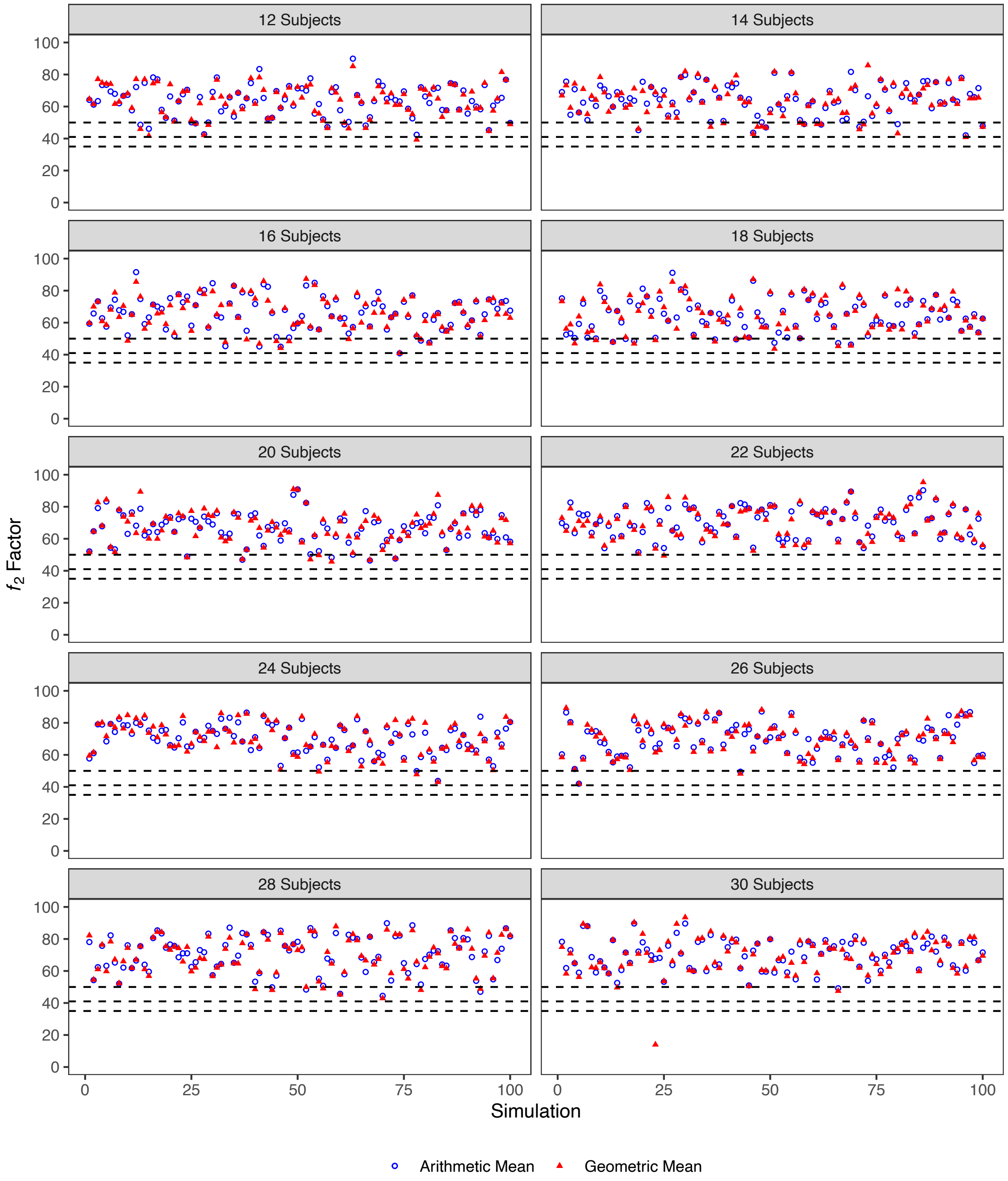

Figure S.39. Similarity f_2 Factor Results, Derived from Arithmetic and Geometric Mean Concentration-Time Profiles Until the Reference C_{max}, Derived from True Bioequivalent Simulations with 0% IIV & 45% IOV in k_a

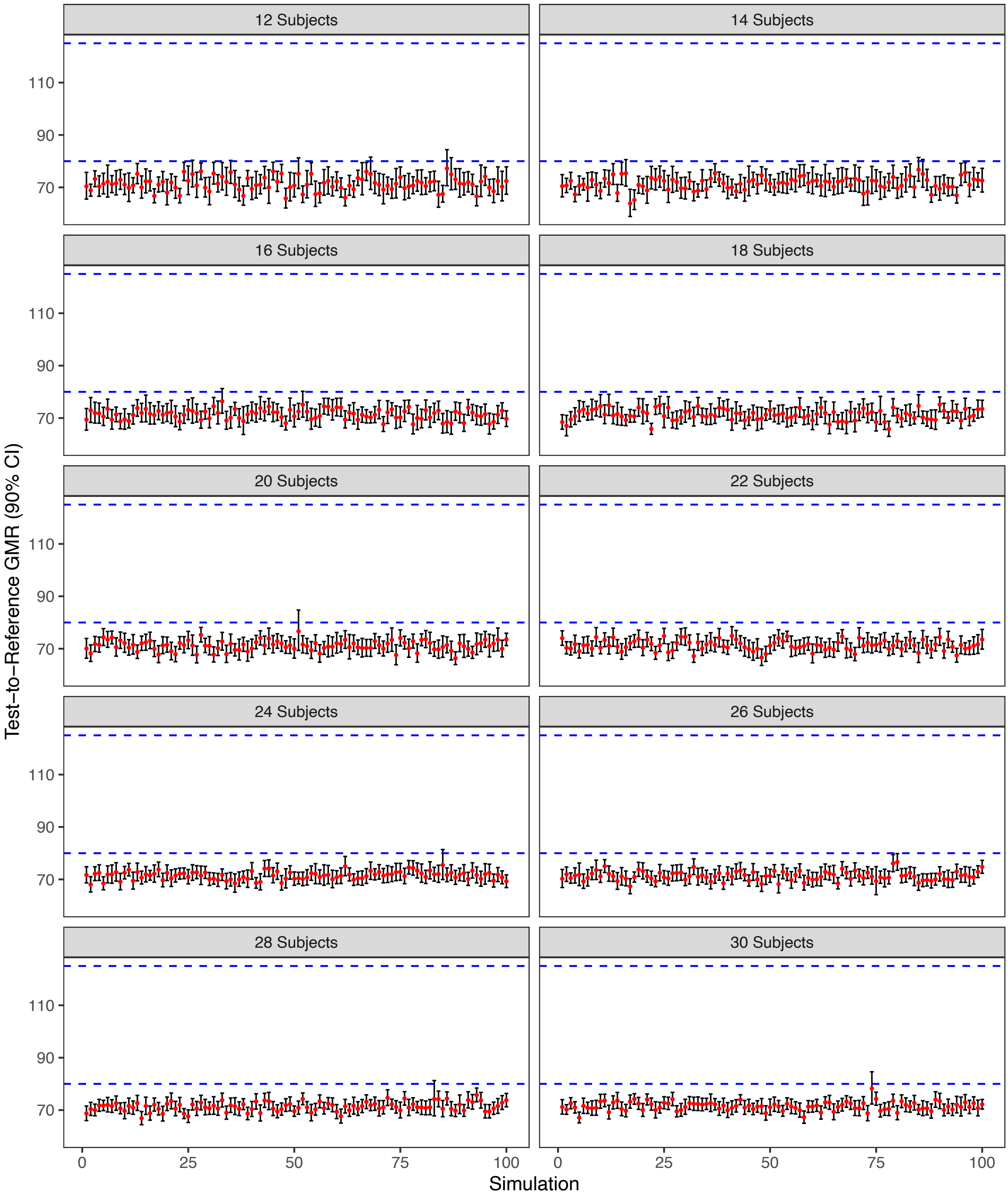

Figure S.40. Average Bioequivalence Analysis Results (Test-to-Reference GMR and Corresponding 90% CI) for C_{max} Derived from True Bioinequivalent Simulations with 30% IIV & 0% IOV in k_a

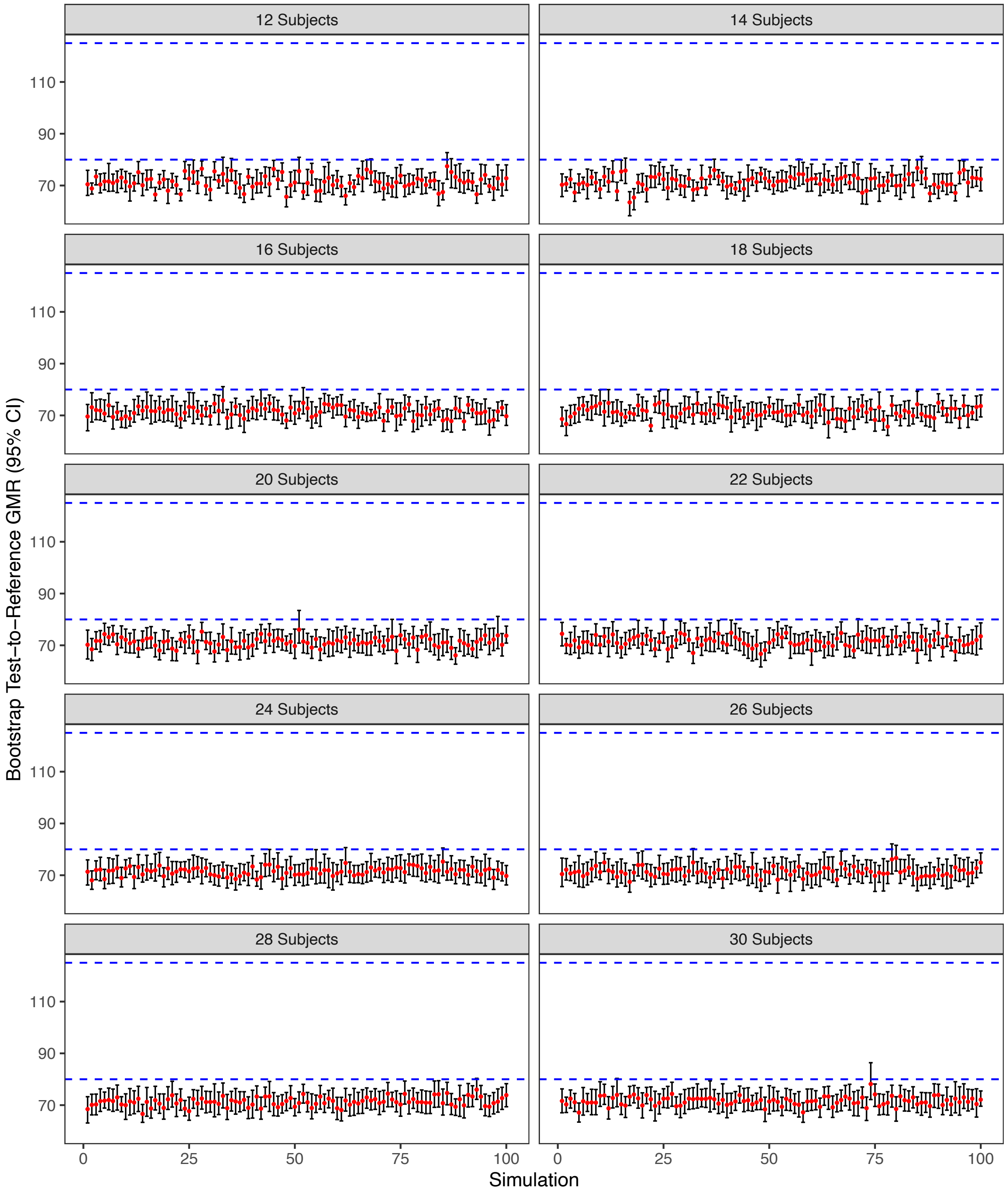

Figure S.41. Bootstrap Bioequivalence Analysis Results (Test-to-Reference GMR and Corresponding 95% CI) for C_{max} Derived from True Bioinequivalent Simulations with 30% IIV & 0% IOV in k_a

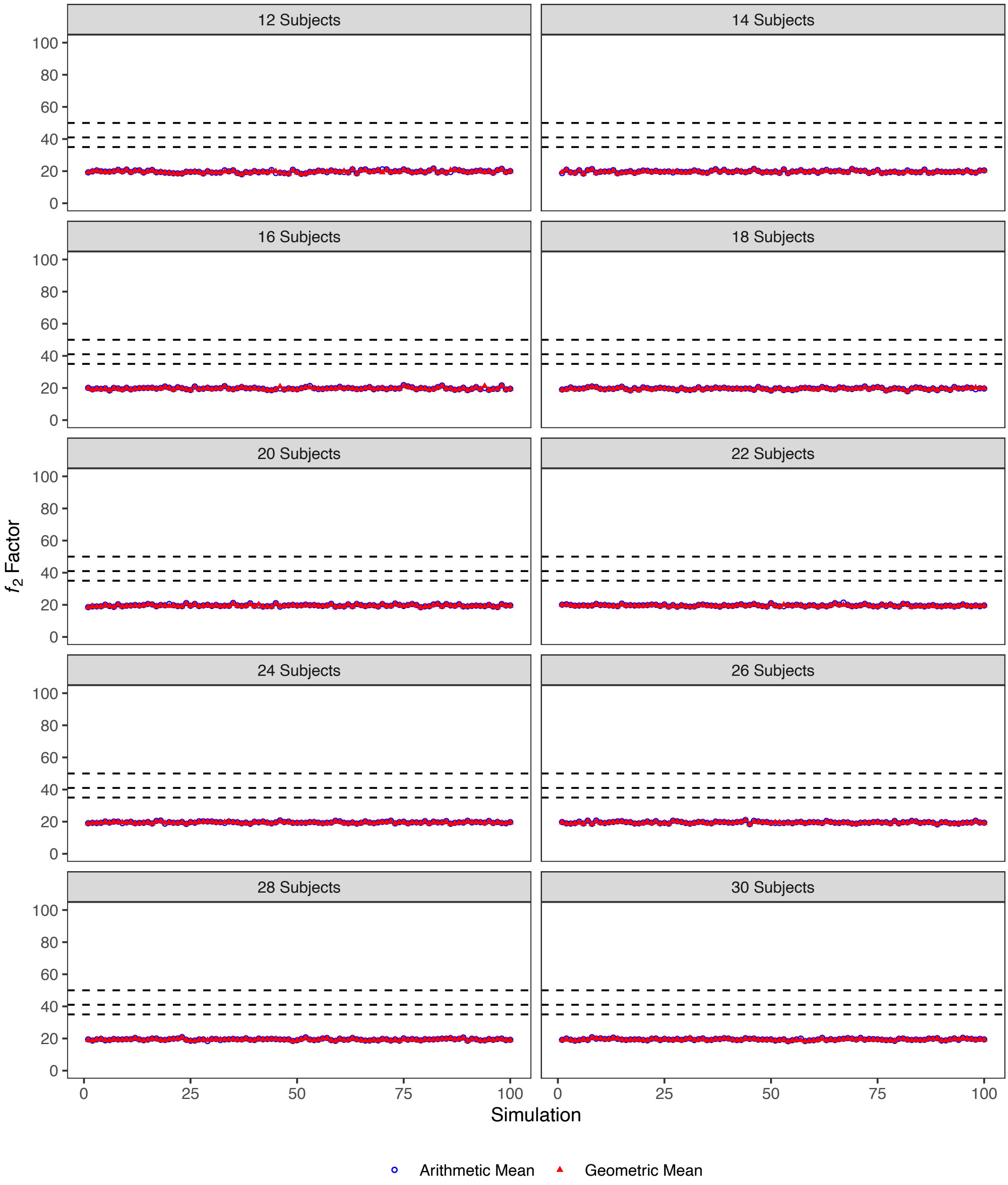

Figure S.42. Similarity f_2 Factor Results, Derived from Arithmetic and Geometric Mean Concentration-Time Profiles Until the Reference C_{max}, Derived from True Bioinequivalent Simulations with 30% IIV & 0% IOV in k_a

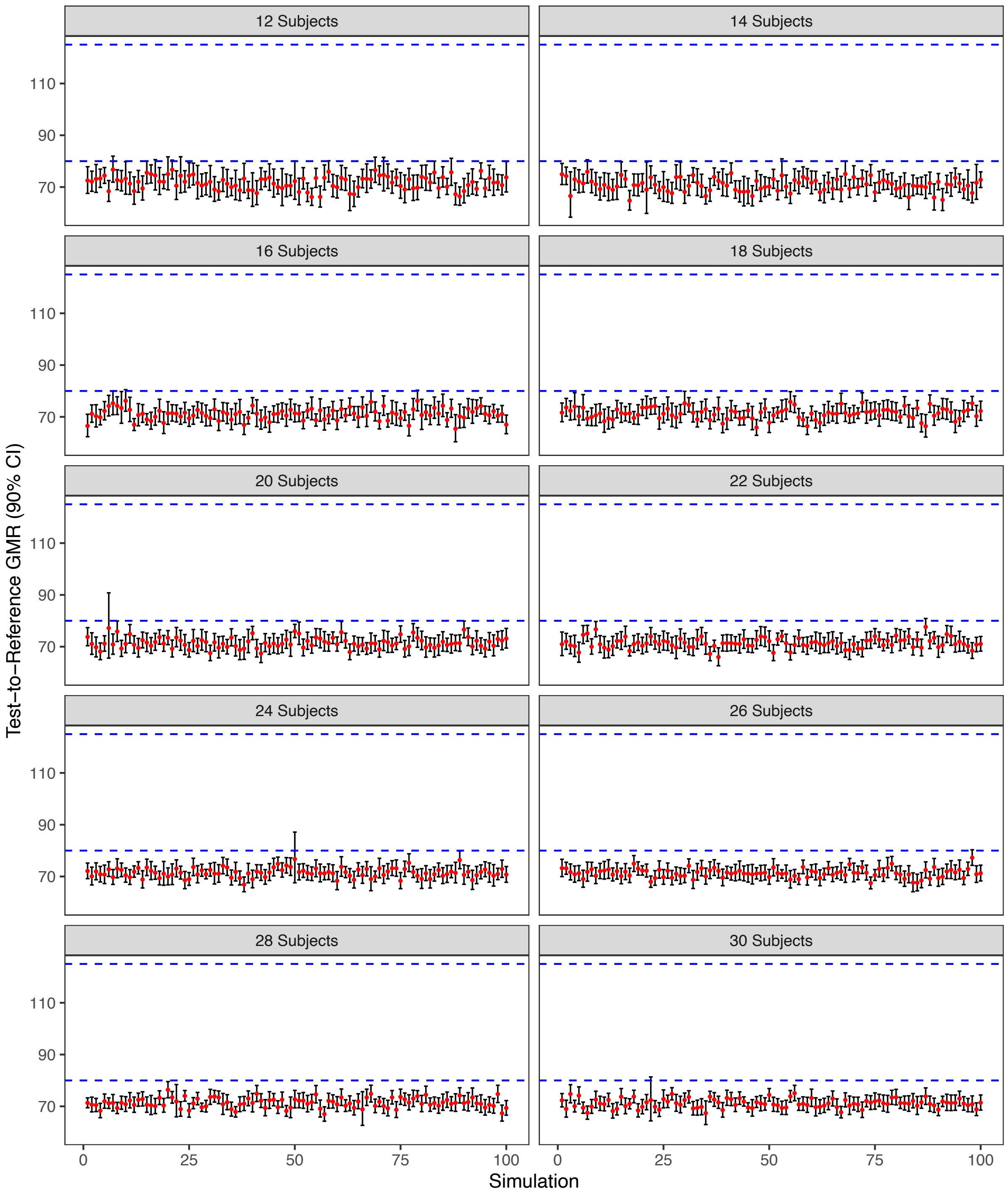

Figure S.43. Average Bioequivalence Analysis Results (Test-to-Reference GMR and Corresponding 90% CI) for C_{max} Derived from True Bioinequivalent Simulations with 30% IIV & 10% IOV in k_a

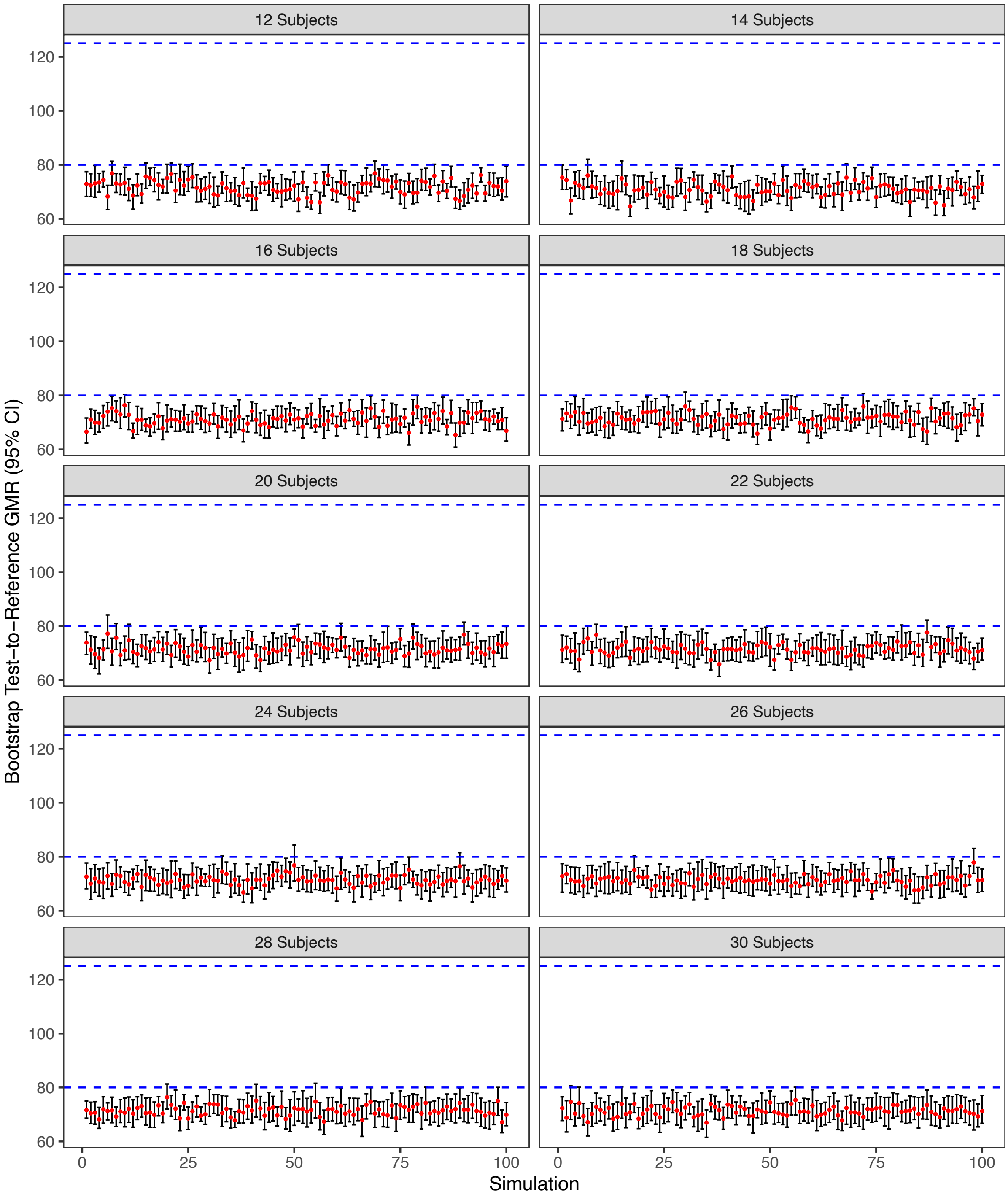

Figure S.44. Bootstrap Bioequivalence Analysis Results (Test-to-Reference GMR and Corresponding 95% CI) for C_{max} Derived from True Bioinequivalent Simulations with 30% IIV & 10% IOV in k_a

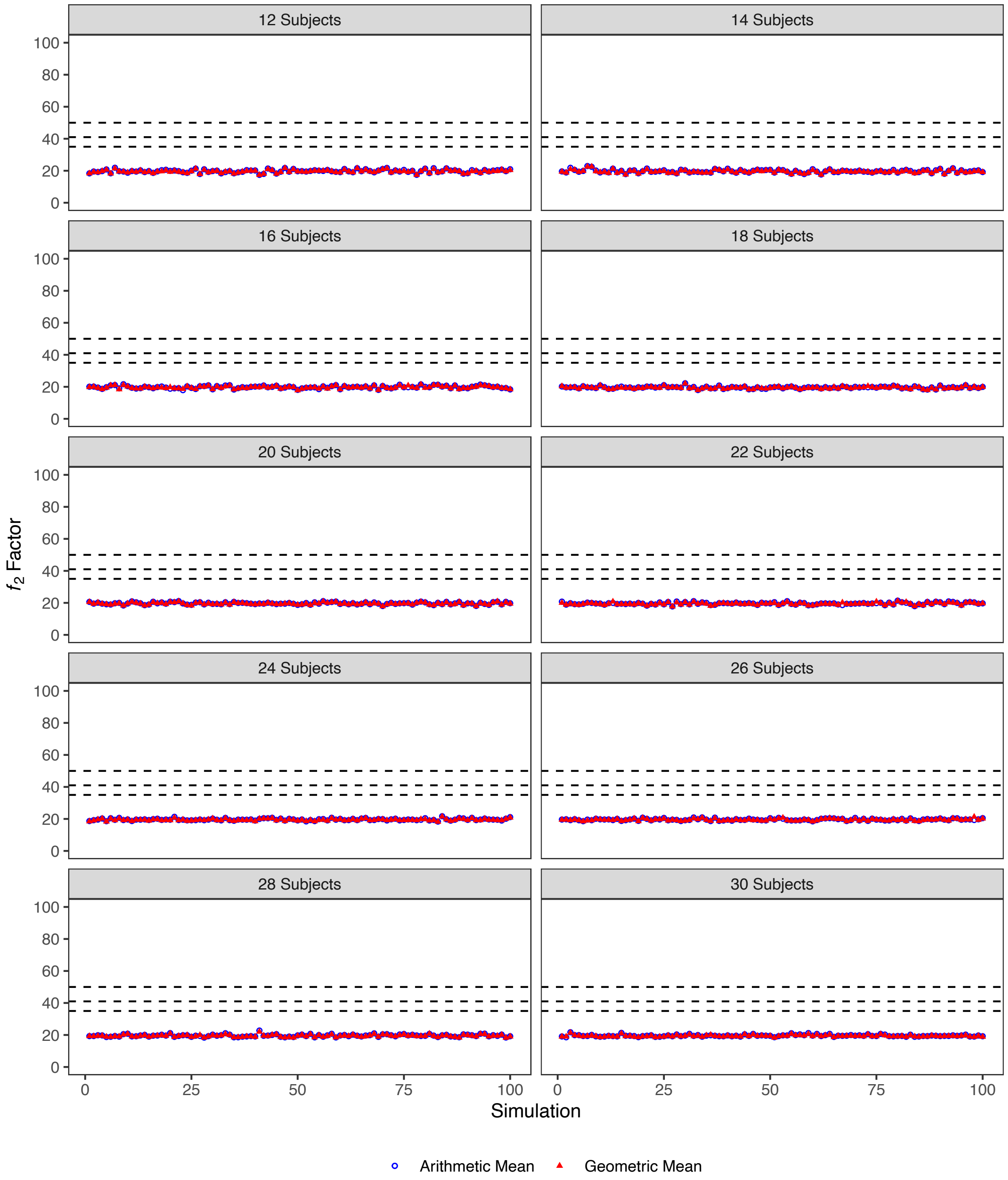

Figure S.45. Similarity f_2 Factor Results, Derived from Arithmetic and Geometric Mean Concentration-Time Profiles Until the Reference C_{max}, Derived from True Bioinequivalent Simulations with 30% IIV & 10% IOV in k_a

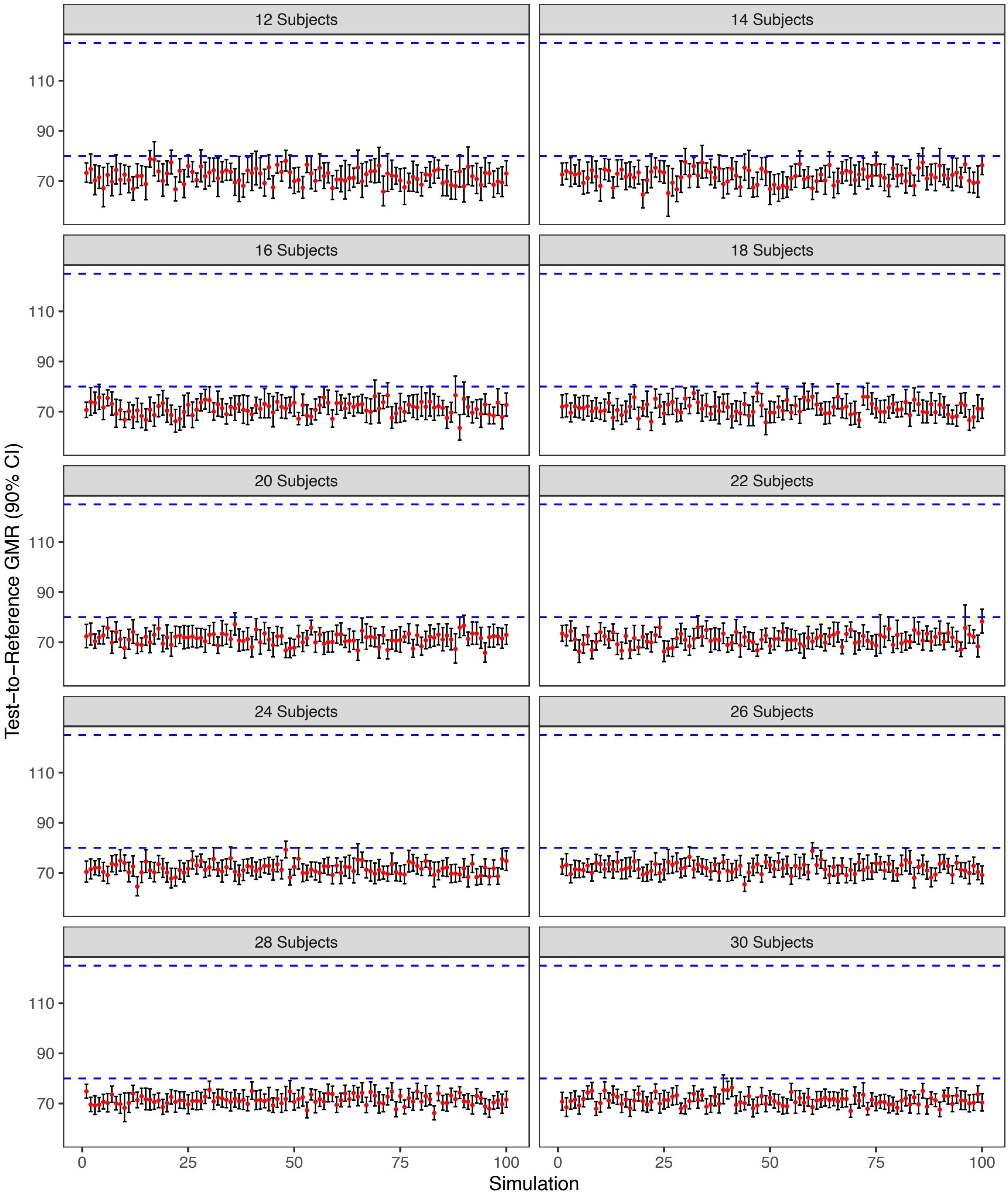

Figure S.46. Average Bioequivalence Analysis Results (Test-to-Reference GMR and Corresponding 90% CI) for C_{max} Derived from True Bioinequivalent Simulations with 30% IIV & 20% IOV in k_a

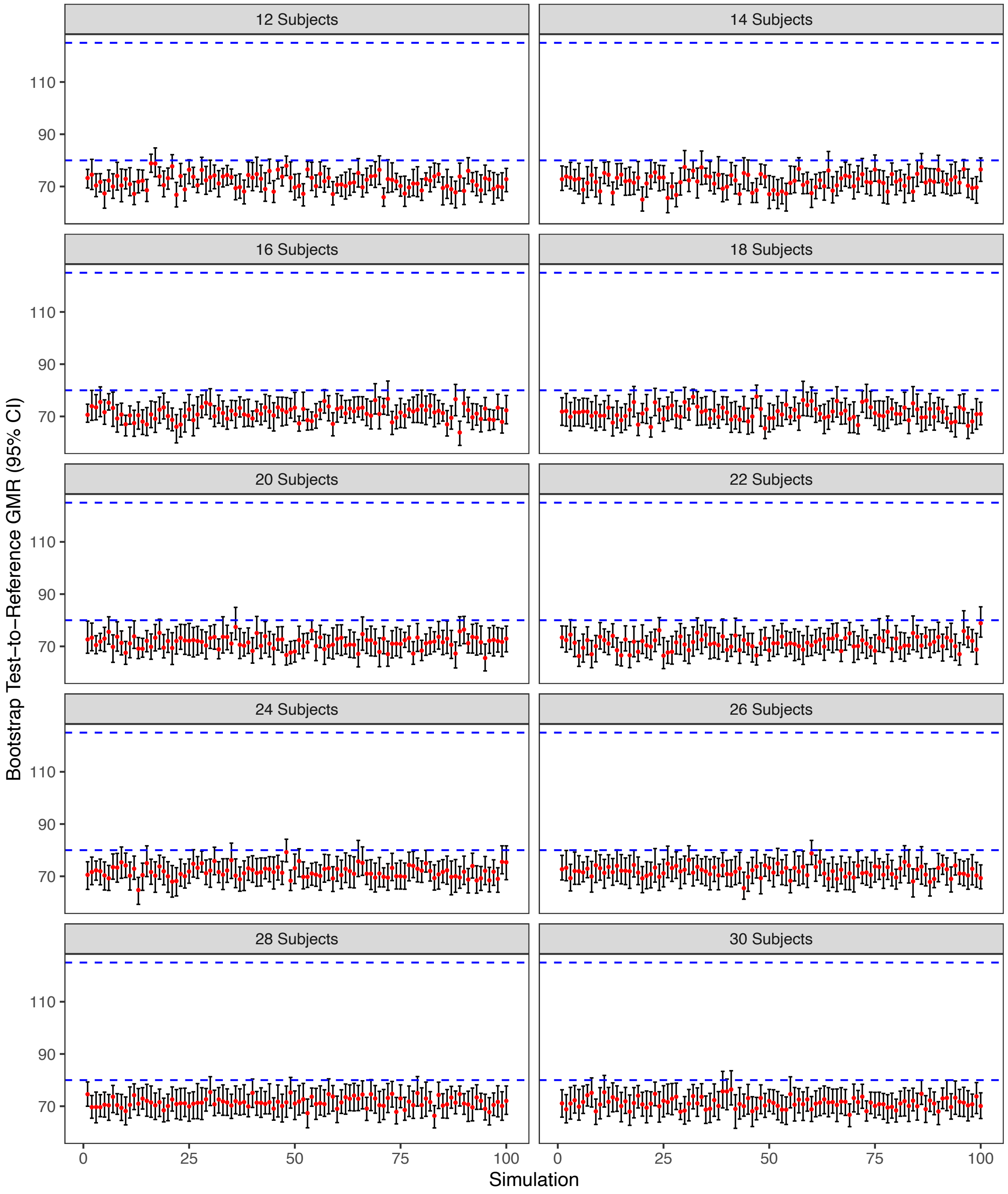

Figure S.47. Bootstrap Bioequivalence Analysis Results (Test-to-Reference GMR and Corresponding 95% CI) for C_{max} Derived from True Bioinequivalent Simulations with 30% IIV & 20% IOV in k_a

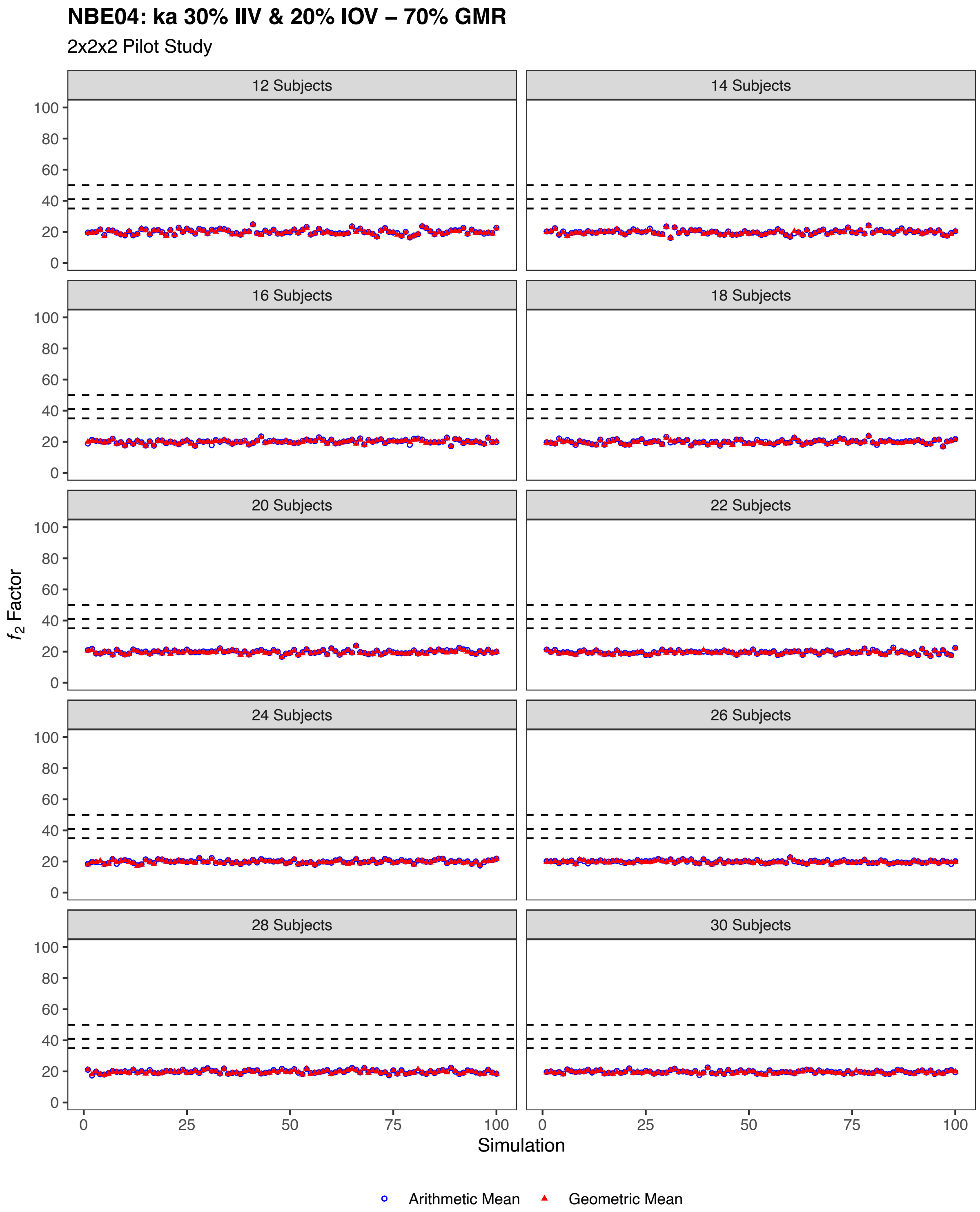

Figure S.48. Similarity f_2 Factor Results, Derived from Arithmetic and Geometric Mean Concentration-Time Profiles Until the Reference C_{max}, Derived from True Bioinequivalent Simulations with 30% IIV & 20% IOV in k_a

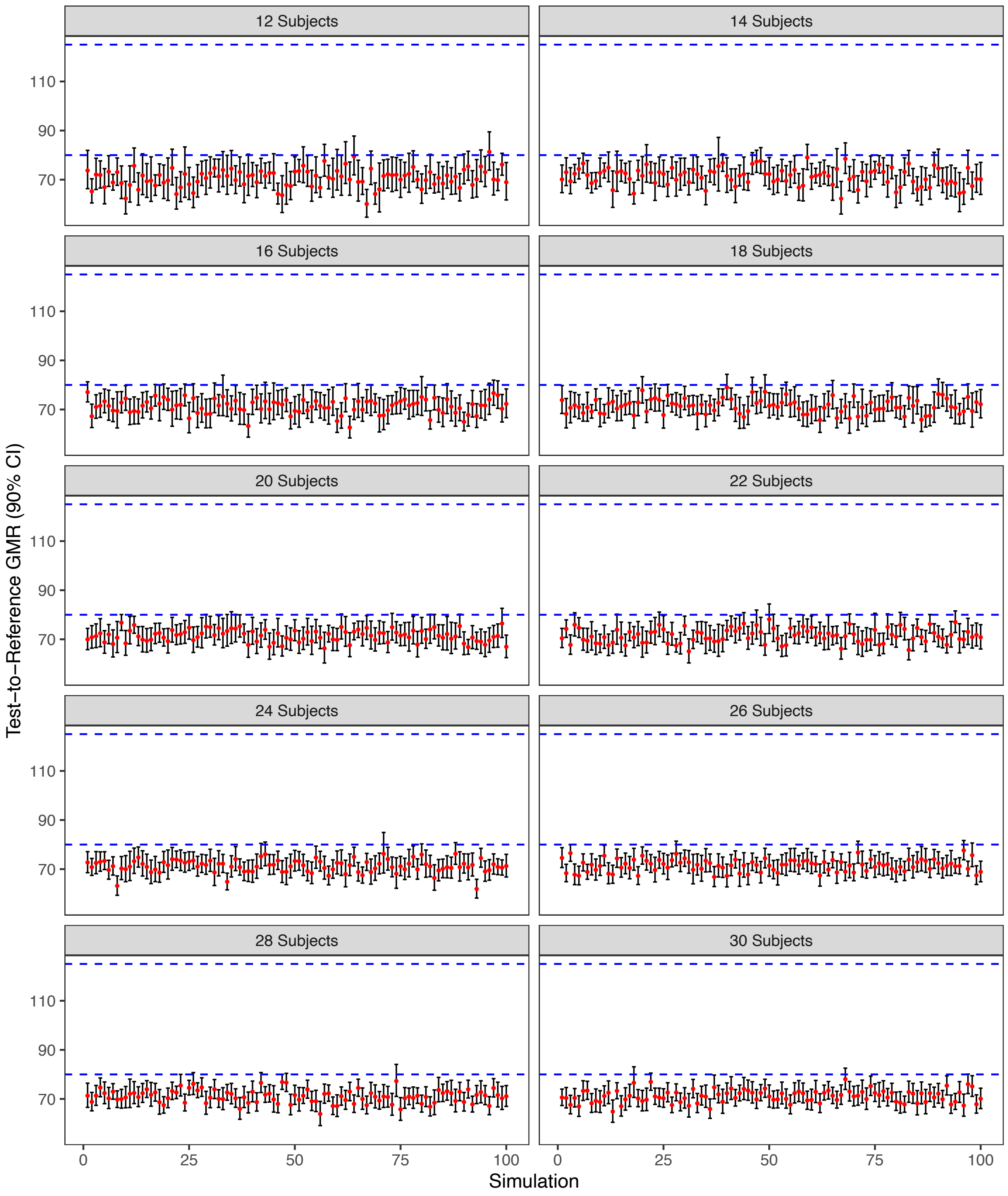

Figure S.49. Average Bioequivalence Analysis Results (Test-to-Reference GMR and Corresponding 90% CI) for C_{max} Derived from True Bioinequivalent Simulations with 30% IIV & 30% IOV in k_a

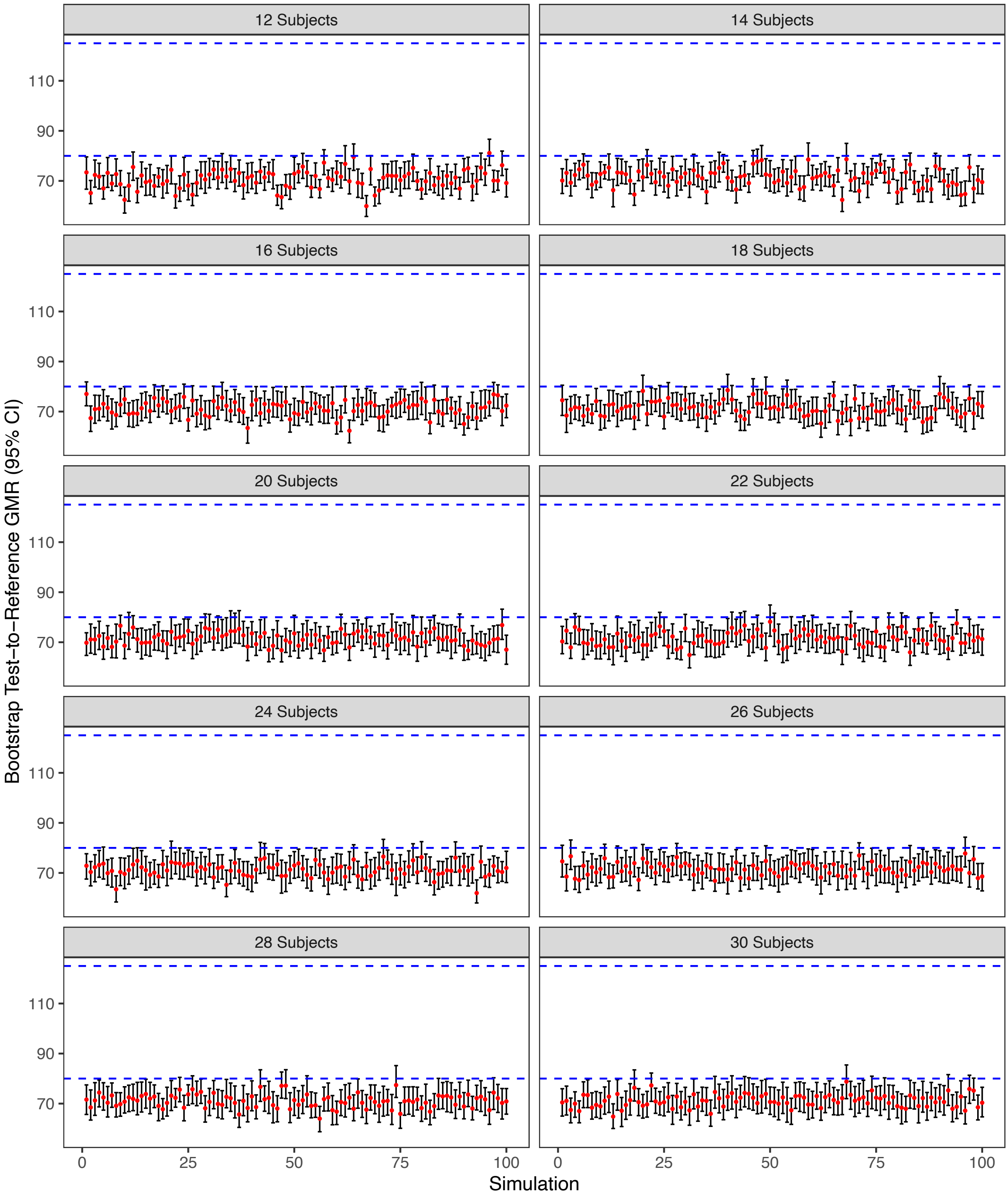

Figure S.50. Bootstrap Bioequivalence Analysis Results (Test-to-Reference GMR and Corresponding 95% CI) for C_{max} Derived from True Bioinequivalent Simulations with 30% IIV & 30% IOV in k_a

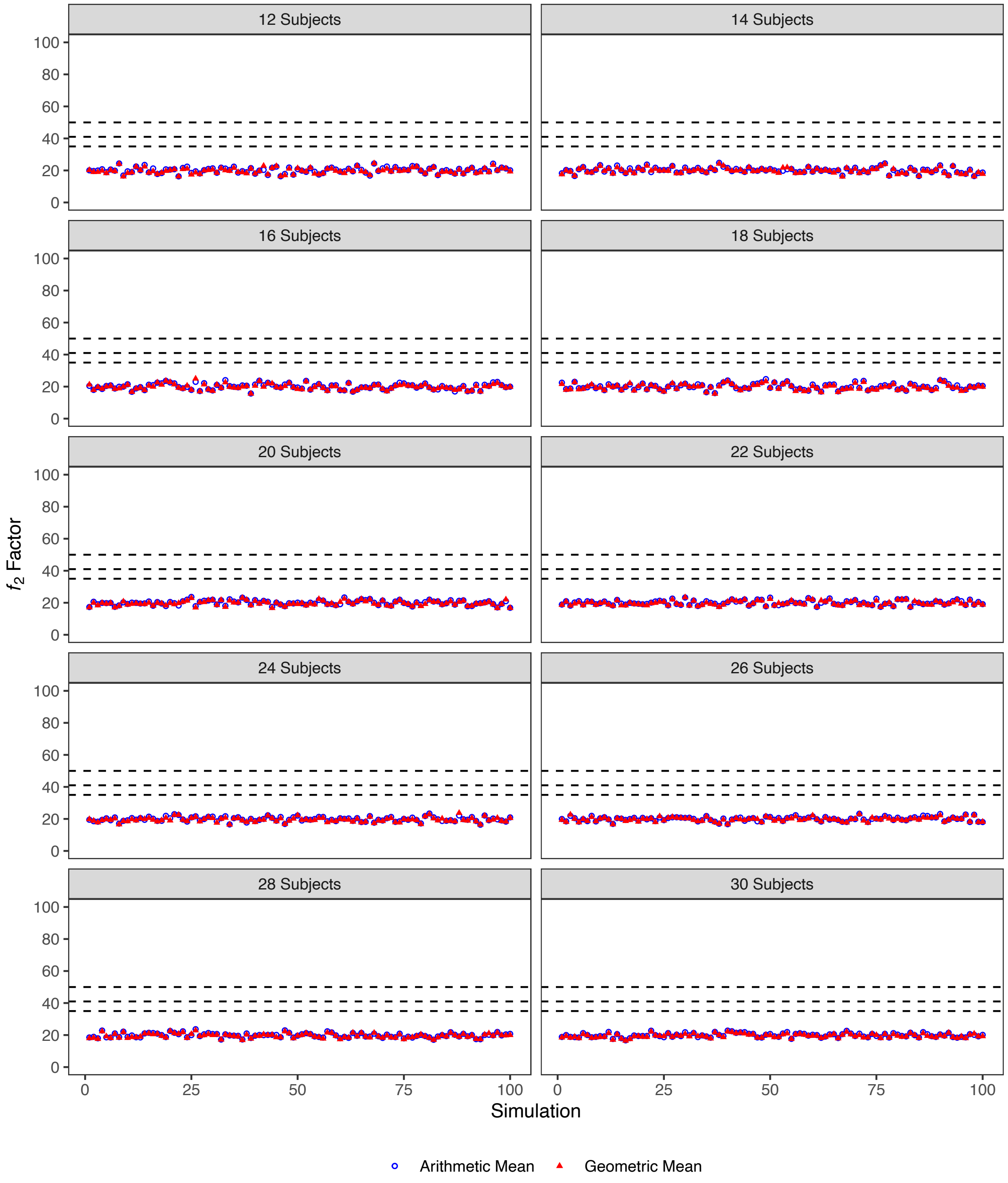

Figure S.51. Similarity f_2 Factor Results, Derived from Arithmetic and Geometric Mean Concentration-Time Profiles Until the Reference C_{max}, Derived from True Bioinequivalent Simulations with 30% IIV & 30% IOV in k_a

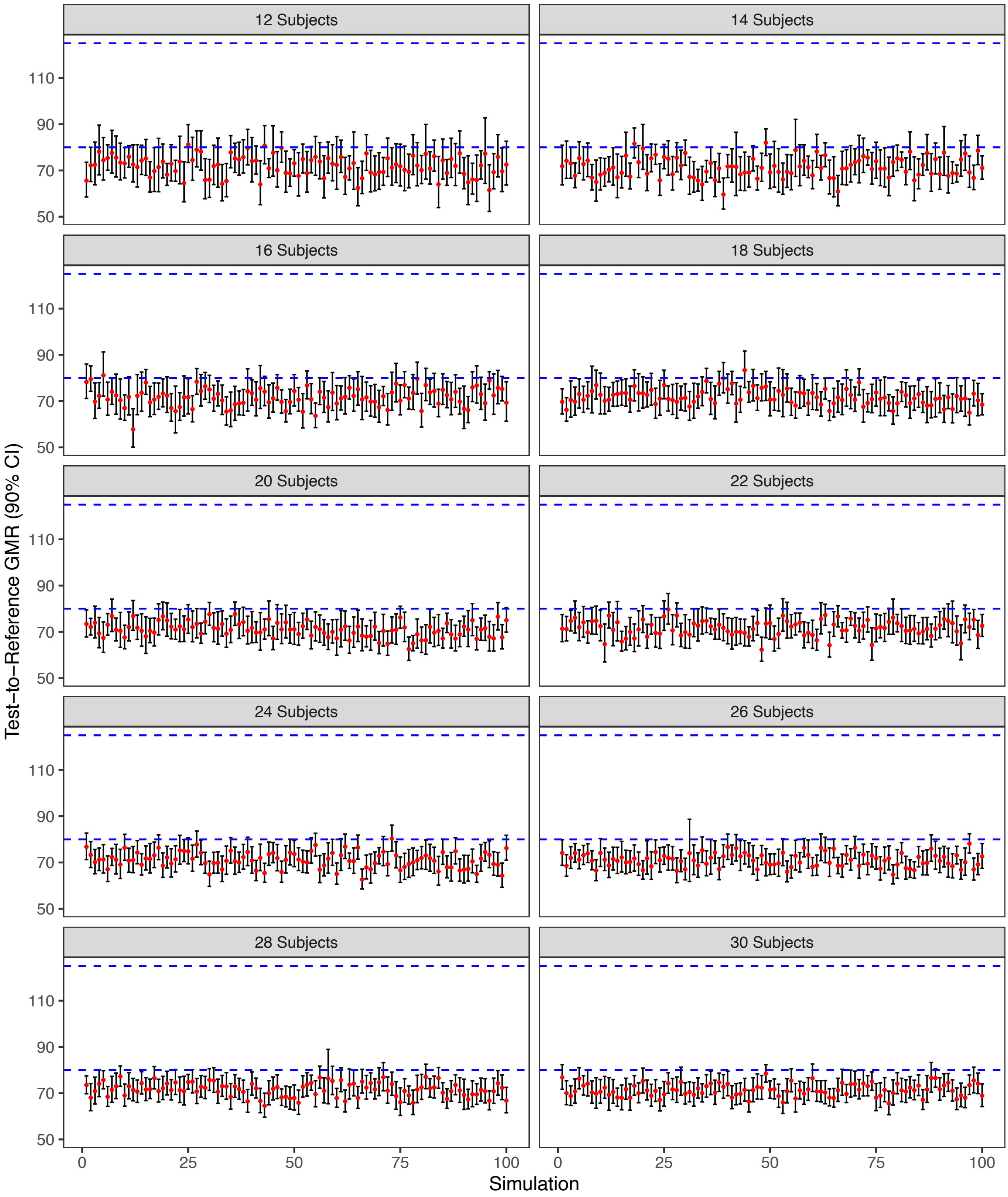

Figure S.52. Average Bioequivalence Analysis Results (Test-to-Reference GMR and Corresponding 90% CI) for C_{max} Derived from True Bioinequivalent Simulations with 0% IIV & 45% IOV in k_a

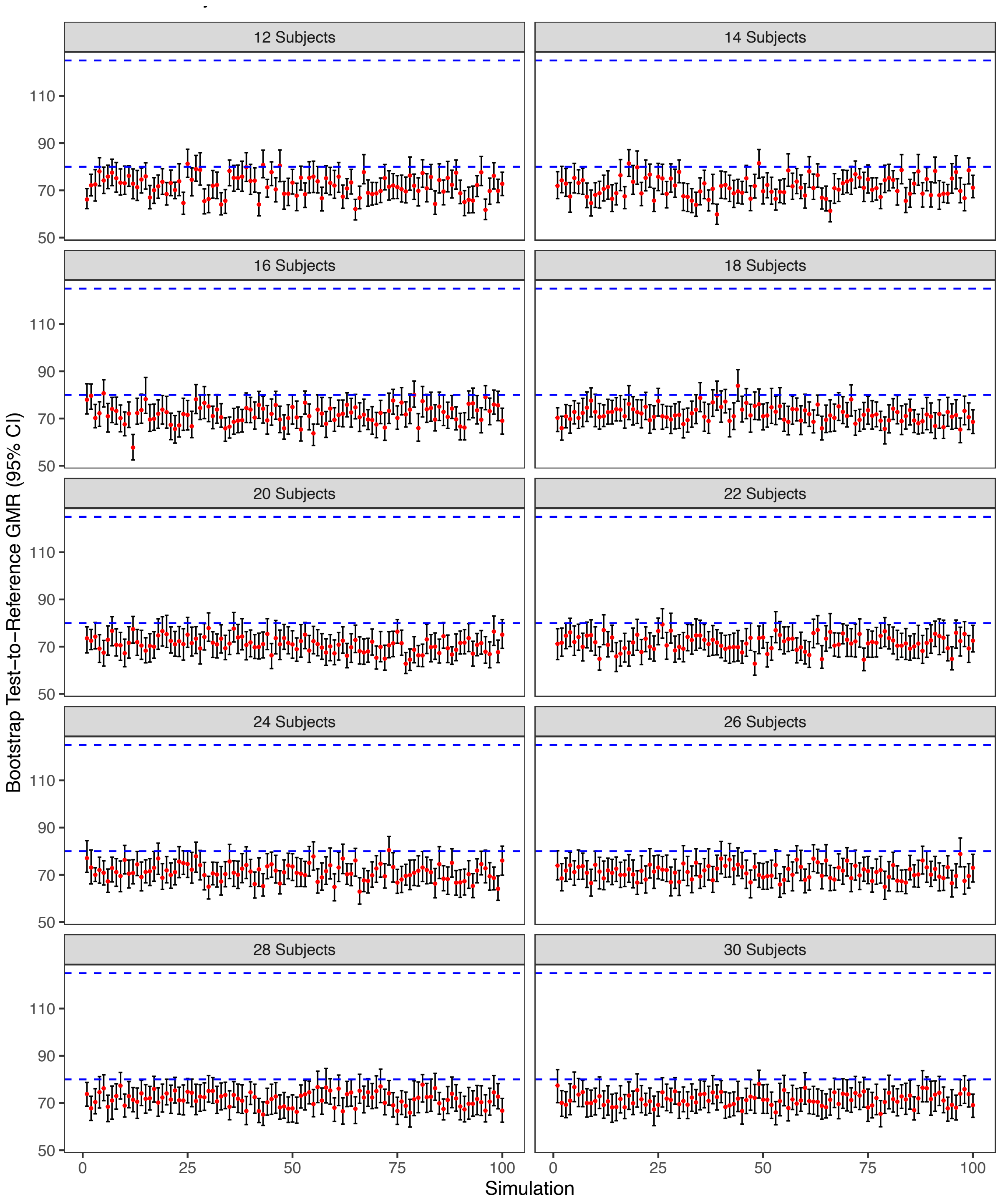

Figure S.53. Bootstrap Bioequivalence Analysis Results (Test-to-Reference GMR and Corresponding 95% CI) for C_{max} Derived from True Bioinequivalent Simulations with 0% IIV & 45% IOV in k_a

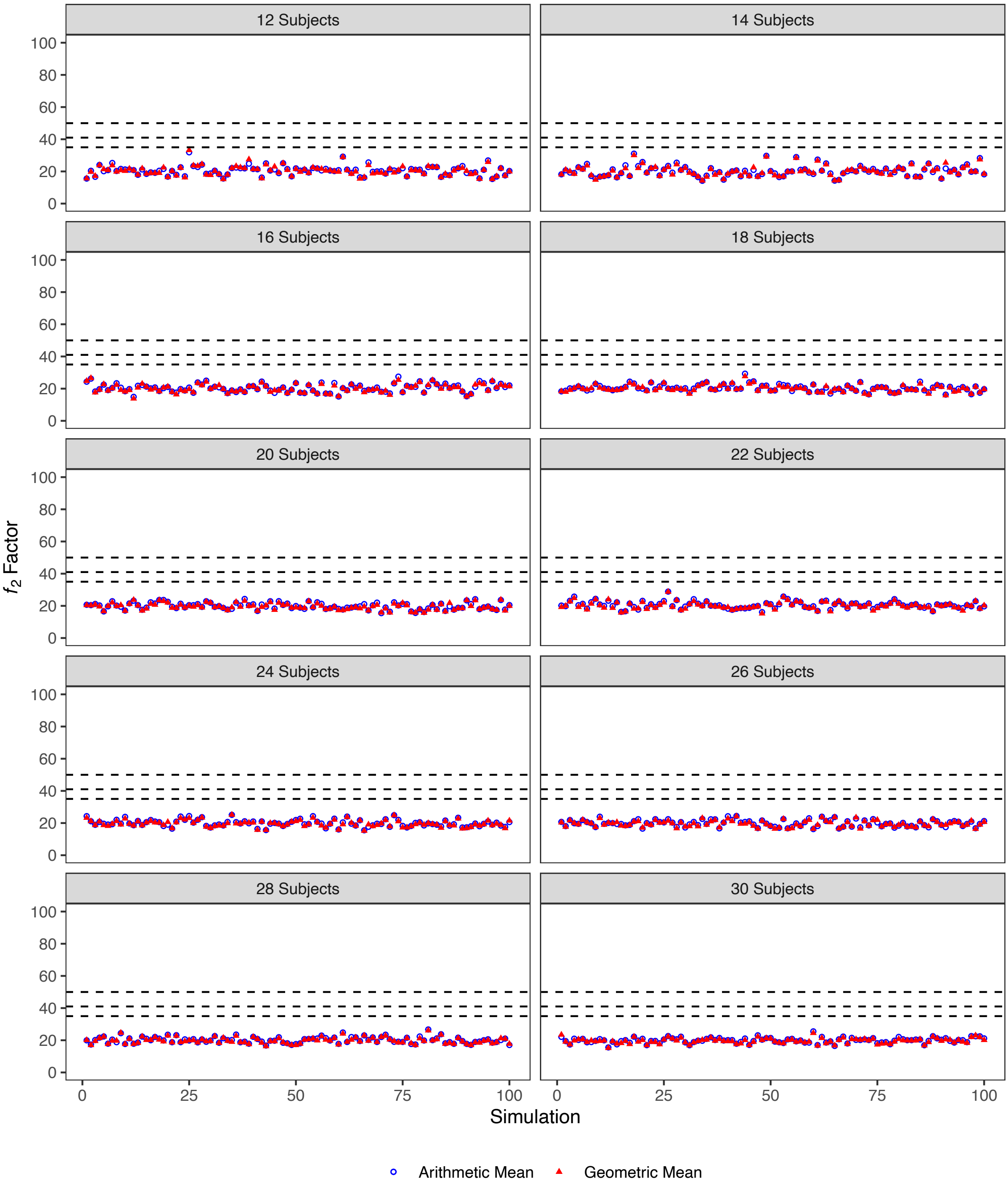

Figure S.54. Similarity f_2 Factor Results, Derived from Arithmetic and Geometric Mean Concentration-Time Profiles Until the Reference C_{max}, Derived from True Bioinequivalent Simulations with 0% IIV & 45% IOV in k_a

Appendix SA.3. Simulated Studies with Variability in V

Appendix SA.3.1. Simulated Pharmacokinetic Parameters

Table S.12. Descriptive Statistics of Simulated V, for True Bioequivalent Studies

	30% IIV 0% IOV		30% IIV 10% IOV		30% IIV 20% IOV		30% IIV 30% IOV		0% IIV 45% IOV	
	Reference	Test	Reference	Test	Reference	Test	Reference	Test	Reference	Test
n	21000	21000	21000	21000	21000	21000	21000	21000	21000	21000
G_{Mean}	58.89	58.89	58.67	58.70	59.23	59.08	58.65	58.77	58.58	58.99
GSD	1.35	1.35	1.37	1.38	1.43	1.43	1.53	1.53	1.57	1.57
GCV%	30.7	30.7	32.6	32.7	36.9	37.0	44.3	44.6	47.6	47.4
G_{mean} 95% CI	58.66 – 59.13	58.66 – 59.13	58.41 – 58.92	58.44 – 58.95	58.94 – 59.52	58.80 – 59.37	58.31 – 58.98	58.43 – 59.11	58.22 – 58.93	58.63 – 59.35
A_{Mean}	61.62	61.62	61.71	61.76	63.14	63.01	64.13	64.35	64.87	65.29
SD	18.95	18.95	20.15	20.23	23.35	23.39	28.30	28.60	30.80	31.02
SE	0.13	0.13	0.14	0.14	0.16	0.16	0.20	0.20	0.21	0.21
CV%	30.8	30.8	32.7	32.8	37.0	37.1	44.1	44.4	47.5	47.5
Variance	359.28	359.28	406.06	409.21	545.44	547.19	800.99	817.86	948.54	962.29
A_{mean} 95% CI	61.36 – 61.87	61.36 – 61.87	61.44 – 61.98	61.49 – 62.04	62.83 – 63.46	62.69 – 63.33	63.74 – 64.51	63.96 – 64.74	64.45 – 65.28	64.87 – 65.71
Minimum	17.13	17.13	18.53	16.31	13.18	16.04	11.19	7.13	10.25	10.35
Q1	48.16	48.16	47.25	47.28	46.50	46.38	44.25	44.22	42.91	43.56
Median	58.76	58.76	58.64	58.81	59.22	59.02	58.59	58.75	58.52	59.07
Q3	72.09	72.09	72.77	72.80	75.41	75.33	77.96	78.38	79.47	79.80
Maximum	202.63	202.63	215.21	253.60	233.41	261.50	309.23	332.10	383.36	384.64

n: Number of observations, G_{mean}: Geometric mean, GSD: Geometric standard deviation, GCV%: Geometric coefficient of variation, G_{mean} 95% CI: 95% confidence interval of the geometric mean, A_{mean}: Arithmetic mean, SD: Standard deviation, SD: Standard error, CV%: Coefficient of variation, A_{mean} 95% CI: 95% confidence interval of the arithmetic mean, Q1: 1st (25%) quartile, Q3: 3rd (75%) quartile

Table S.13. Descriptive Statistics of Simulated V, for True Bioinequivalent Studies

	30% IIV 0% IOV		30% IIV 10% IOV		30% IIV 20% IOV		30% IIV 30% IOV		0% IIV 45% IOV	
	**Reference**	**Test**	**Reference**	**Test**	**Reference**	**Test**	**Reference**	**Test**	**Reference**	**Test**
n	21000	21000	21000	21000	21000	21000	21000	21000	21000	21000
G_{Mean}	58.70	58.70	58.80	58.73	58.83	58.74	59.09	58.98	58.93	58.96
GSD	1.35	1.35	1.38	1.38	1.44	1.43	1.53	1.53	1.57	1.57
GCV%	30.7	30.7	32.8	32.7	37.3	37.2	44.3	44.8	47.6	47.5
G_{mean} 95% CI	58.46 – 58.94	58.46 – 58.94	58.55 – 59.06	58.48 – 58.98	58.54 – 59.12	58.46 – 59.03	58.75 – 59.43	58.64 – 59.32	58.57 – 59.29	58.61 – 59.33
A_{Mean}	61.39	61.39	61.88	61.79	62.80	62.69	64.66	64.63	65.28	65.31
SD	18.74	18.74	20.35	20.19	23.42	23.42	28.84	29.01	31.25	31.28
SE	0.13	0.13	0.14	0.14	0.16	0.16	0.20	0.20	0.22	0.22
CV%	30.5	30.5	32.9	32.7	37.3	37.4	44.6	44.9	47.9	47.9
Variance	351.34	351.34	414.23	407.61	548.58	548.54	832.00	841.80	976.70	978.51
A_{mean} 95% CI	61.14 – 61.64	61.14 – 61.64	61.61 – 62.16	61.51 – 62.06	62.48 – 63.11	62.38 – 63.01	64.27 – 65.05	64.24 – 65.02	64.86 – 65.70	64.89 – 65.74
Minimum	18.85	18.85	17.91	16.51	13.92	16.62	12.70	9.48	10.37	10.37
Q1	47.94	47.94	47.39	47.35	46.12	46.07	44.21	44.05	43.55	43.61
Median	58.80	58.80	58.87	58.86	58.90	58.57	58.96	59.29	58.73	59.00
Q3	71.94	71.94	72.84	72.70	75.18	74.85	78.54	78.68	79.62	79.75
Maximum	184.65	184.65	211.43	196.78	274.17	293.65	300.95	321.78	405.13	339.83

n: Number of observations, G_{mean}: Geometric mean, GSD: Geometric standard deviation, GCV%: Geometric coefficient of variation, G_{mean} 90% CI: 90% confidence interval of the geometric mean, A_{mean}: Arithmetic mean, SD: Standard deviation, SD: Standard error, CV%: Coefficient of variation, A_{mean} 90% CI: 90% confidence interval of the arithmetic mean, Q1: 1st (25%) quartile, Q3: 3rd (75%) quartile

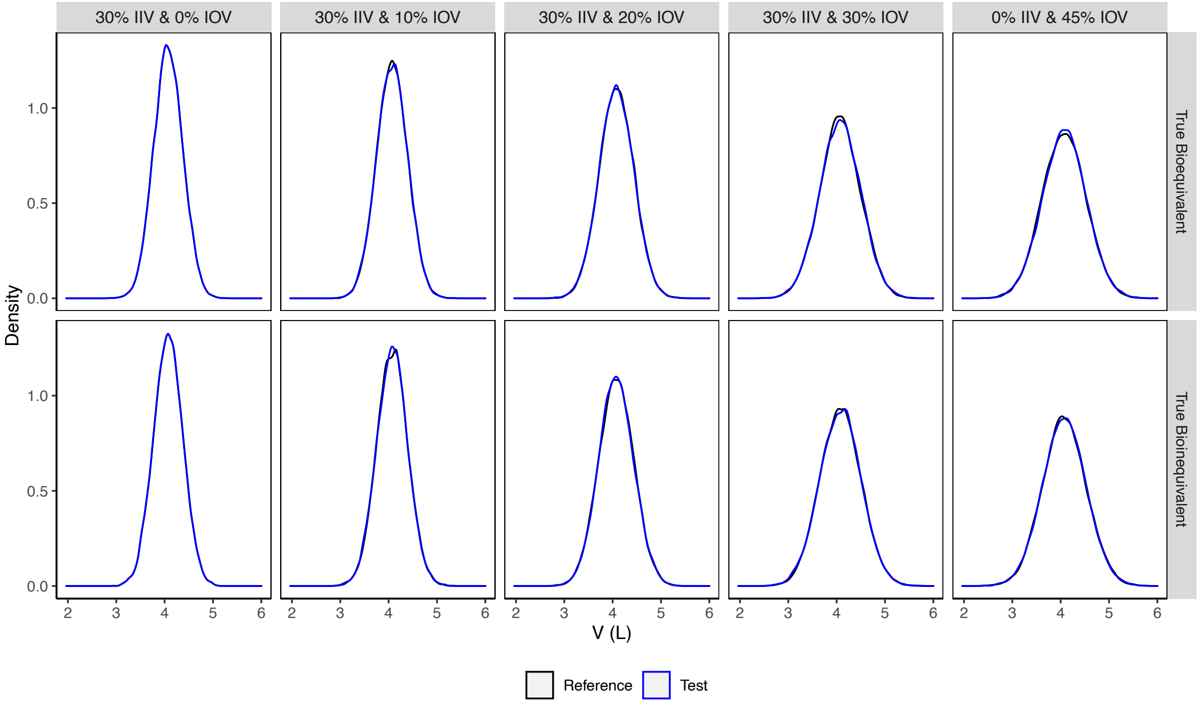

Figure S.55. Distribution of Simulated *Ln*-Transformed V

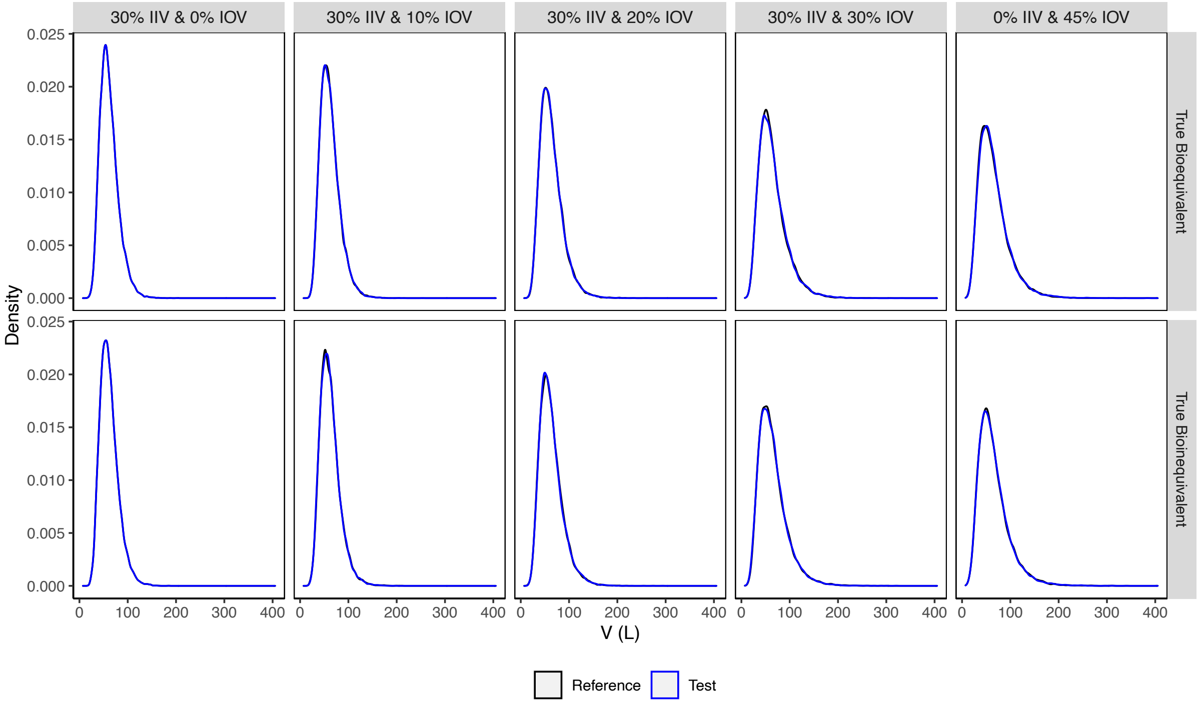

Figure S.56. Distribution of Simulated Untransformed V

Appendix SA.3.2. Simulated Concentrations

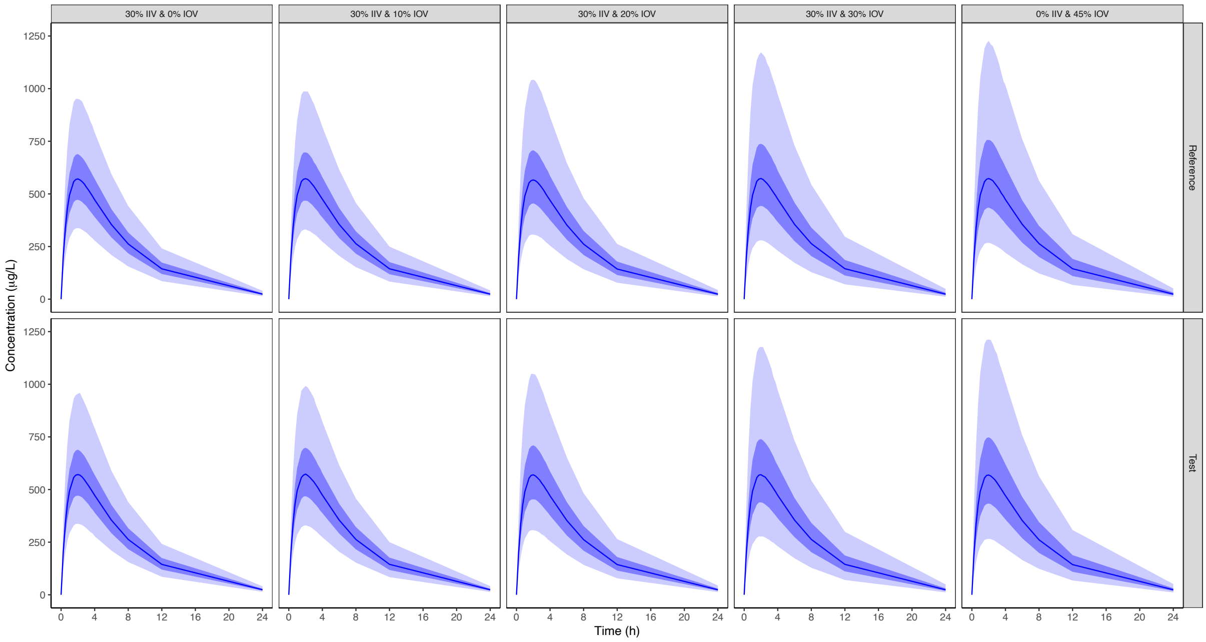

Figure S.57. 95% Confidence Interval of Simulated Concentration-Time Data, for True Bioequivalent Studies, with Variability in V – Linear Scale

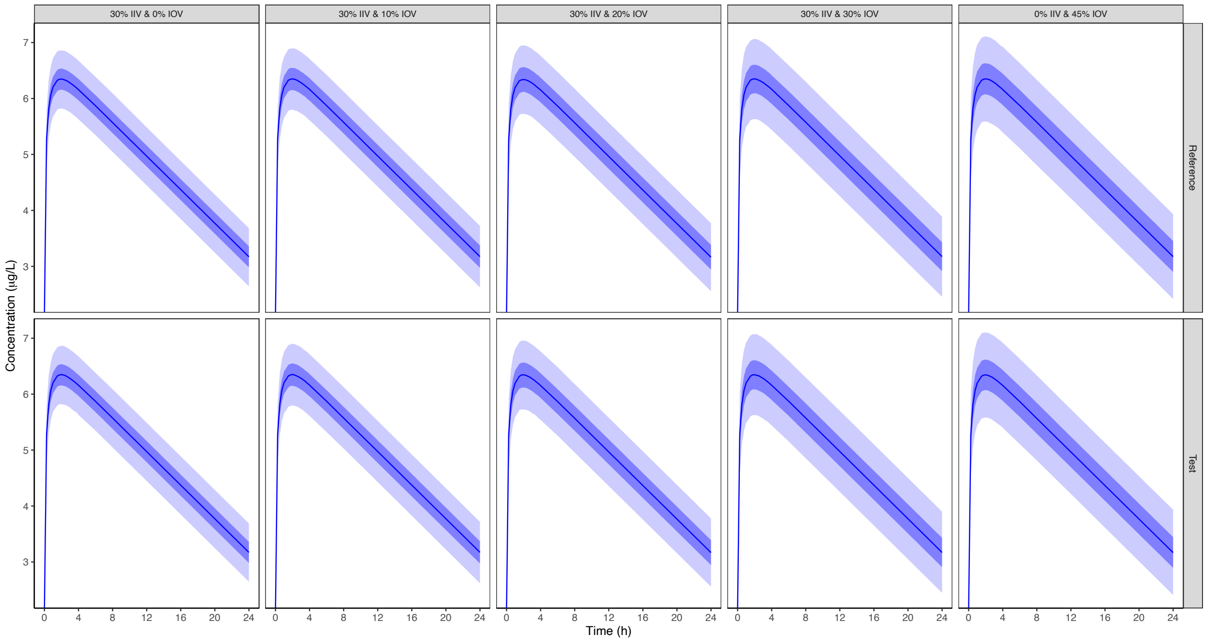

Figure S.58. 95% Confidence Interval of Simulated Concentration-Time Data, for True Bioequivalent Studies, with Variability in V – Semi-Logarithmic Scale

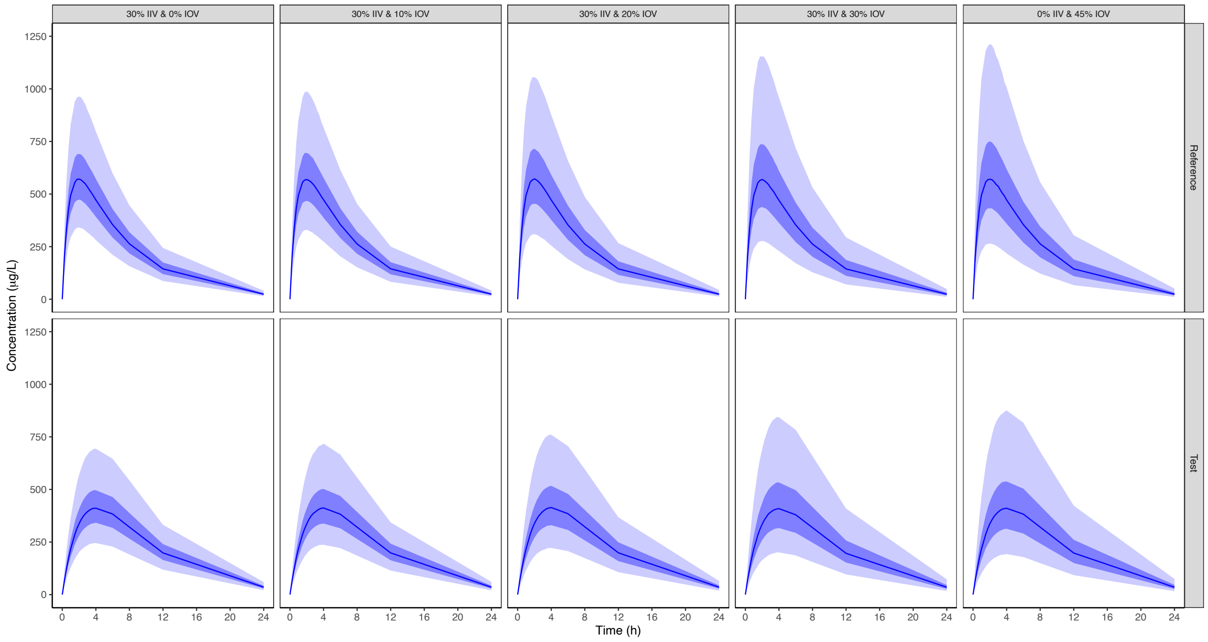

Figure S.59. 95% Confidence Interval of Simulated Concentration-Time Data, for True Bioinequivalent Studies, with Variability in V – Linear Scale

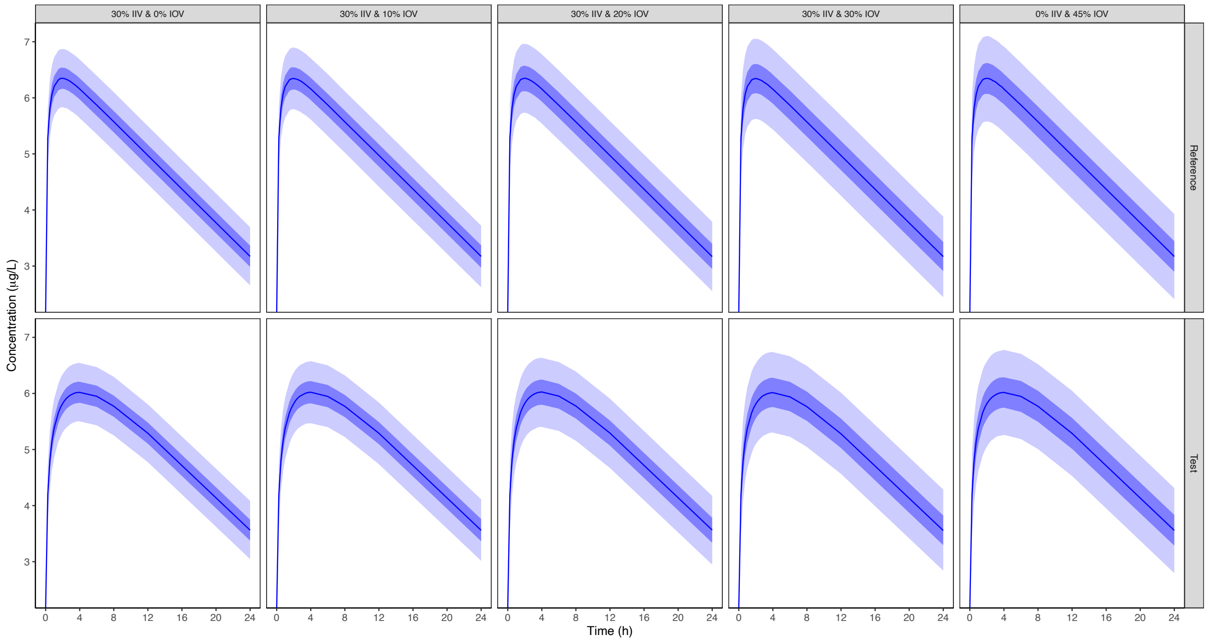

Figure S.60. 95% Confidence Interval of Simulated Concentration-Time Data, for True Bioinequivalent Studies, with Variability in V – Semi-Logarithmic Scale

Appendix SA.3.3. Simulated Non-Compartmental Analysis Parameters

Table S.14. Descriptive Statistics of C_{max} (µg/L) Derived from Simulated True Bioequivalent Studies, with Variability in V

	30% IIV 0% IOV		30% IIV 10% IOV		30% IIV 20% IOV		30% IIV 30% IOV		0% IIV 45% IOV	
	Reference	Test	Reference	Test	Reference	Test	Reference	Test	Reference	Test
n	21000	21000	21000	21000	21000	21000	21000	21000	21000	21000
G_{Mean}	641.22	641.77	644.2	643.42	637.95	639.7	643.81	642.2	645.13	640.63
GSD	1.36	1.36	1.38	1.38	1.44	1.44	1.53	1.54	1.58	1.58
GCV%	31.4	31.4	33.3	33.3	37.5	37.6	44.7	45.2	48.1	47.9
G_{mean} 95% CI	638.56 – 643.88	639.12 – 644.44	641.38 – 647.03	640.60 – 646.25	634.83 – 641.09	636.57 – 642.86	640.11 – 647.54	638.46 – 645.96	641.16 – 649.13	636.71 – 644.58
A_{Mean}	672.08	672.69	678.94	678.21	681.33	683.4	705.26	705	715.86	710.19
SD	210.77	211.11	225.41	225.37	255.37	257.23	316.32	320.27	343.37	339.08
SE	1.45	1.46	1.56	1.56	1.76	1.78	2.18	2.21	2.37	2.34
CV%	31.4	31.4	33.2	33.2	37.5	37.6	44.9	45.4	48	47.7
Variance	44425.1	44568.77	50810.09	50793.47	65215.33	66166.7	100055.9	102572.53	117906.04	114977.69
A_{mean} 95% CI	669.23 – 674.93	669.84 – 675.55	675.89 – 681.99	675.16 – 681.26	677.87 – 684.78	679.92 – 686.88	700.98 – 709.54	700.67 – 709.33	711.22 – 720.51	705.61 – 714.78
Minimum	175.5	191.23	195.58	135.8	168.6	143.48	131.46	117.92	96.28	98.06
Q1	521.59	521.58	517.25	516.18	498.45	499.52	484.5	479.56	474.24	473.16
Median	641.59	643.57	644.77	643.4	639.85	641.04	644.19	640.89	644.12	638.05
Q3	790.22	789.61	802.29	802.12	815.5	817.9	856.71	859.28	881.11	870.41
Maximum	2174.99	2381.99	2103.38	2234.27	3268.62	2616.26	3954.37	5064.56	3720.72	3366.91

n: Number of observations, G_{mean}: Geometric mean, GSD: Geometric standard deviation, GCV%: Geometric coefficient of variation, G_{mean} 95% CI: 95% confidence interval of the geometric mean, A_{mean}: Arithmetic mean, SD: Standard deviation, SD: Standard error, CV%: Coefficient of variation, A_{mean} 95% CI: 95% confidence interval of the arithmetic mean, Q1: 1st (25%) quartile, Q3: 3rd (75%) quartile

Table S.15. Descriptive Statistics of C_{max} (μg/L) Derived from Simulated True Bioinequivalent Studies, with Variability in V

	30% IIV 0% IOV		30% IIV 10% IOV		30% IIV 20% IOV		30% IIV 30% IOV		0% IIV 45% IOV	
	Reference	Test	Reference	Test	Reference	Test	Reference	Test	Reference	Test
n	21000	21000	21000	21000	21000	21000	21000	21000	21000	21000
G_{Mean}	643.51	461.52	642.04	460.93	641.81	460.91	638.7	458.88	641.19	459.33
GSD	1.36	1.36	1.38	1.38	1.44	1.44	1.54	1.54	1.58	1.58
GCV%	31.4	31.4	33.4	33.4	38.0	37.9	44.9	45.3	48.1	48.0
G_{mean} 95% CI	640.85 – 646.18	459.61 – 463.44	639.22 – 644.87	458.91 – 462.96	638.63 – 645.00	458.63 – 463.19	635.01 – 642.41	456.21 – 461.57	637.25 – 645.15	456.51 – 462.17
A_{Mean}	674.47	483.82	676.84	485.92	686.57	492.77	699.86	503.83	711.35	509.46
SD	212.11	152.42	226.03	162.03	260.62	185.74	311.55	228.86	342.2	244.36
SE	1.46	1.05	1.56	1.12	1.8	1.28	2.15	1.58	2.36	1.69
CV%	31.4	31.5	33.4	33.3	38.0	37.7	44.5	45.4	48.1	48.0
Variance	44991.05	23230.82	51087.56	26253.06	67921.24	34499.66	97064.66	52378.36	117099.37	59711.56
A_{mean} 95% CI	671.60 – 677.34	481.76 – 485.88	673.78 – 679.90	483.73 – 488.11	683.04 – 690.09	490.26 – 495.28	695.65 – 704.07	500.73 – 506.92	706.72 – 715.98	506.16 – 512.77
Minimum	216.57	132.92	185.99	140.56	121.37	88.85	122.87	87.46	92.25	76.3
Q1	523	374.78	516.39	370.93	499.79	360.4	476.63	344.11	472.14	339
Median	641.64	460.54	641.45	459.01	641.76	462.46	640.38	456.7	642.13	459.45
Q3	791.47	567.44	799.13	574.9	824.08	589.31	858.44	614.42	870.38	624.31
Maximum	2113.89	1562.95	2195.95	1691.72	2851.48	1721.36	3189.02	2852.31	3748.34	2755.17

n: Number of observations, G_{mean}: Geometric mean, GSD: Geometric standard deviation, GCV%: Geometric coefficient of variation, G_{mean} 95% CI: 95% confidence interval of the geometric mean, A_{mean}: Arithmetic mean, SD: Standard deviation, SD: Standard error, CV%: Coefficient of variation, A_{mean} 95% CI: 95% confidence interval of the arithmetic mean, Q1: 1st (25%) quartile, Q3: 3rd (75%) quartile

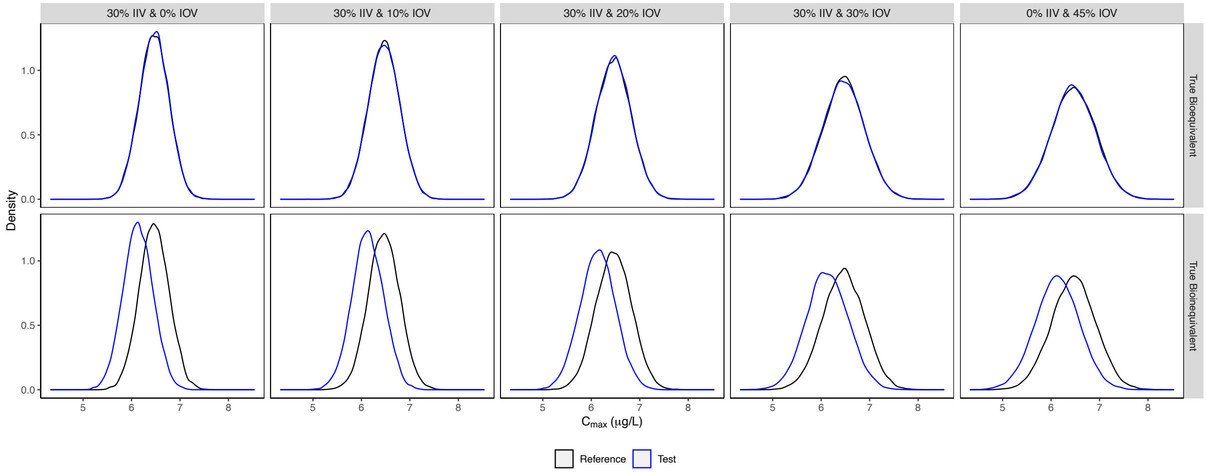

Figure S.61. Distribution of *Ln*-Transformed C_{max}, Derived from Simulations for Studies with Variability in V

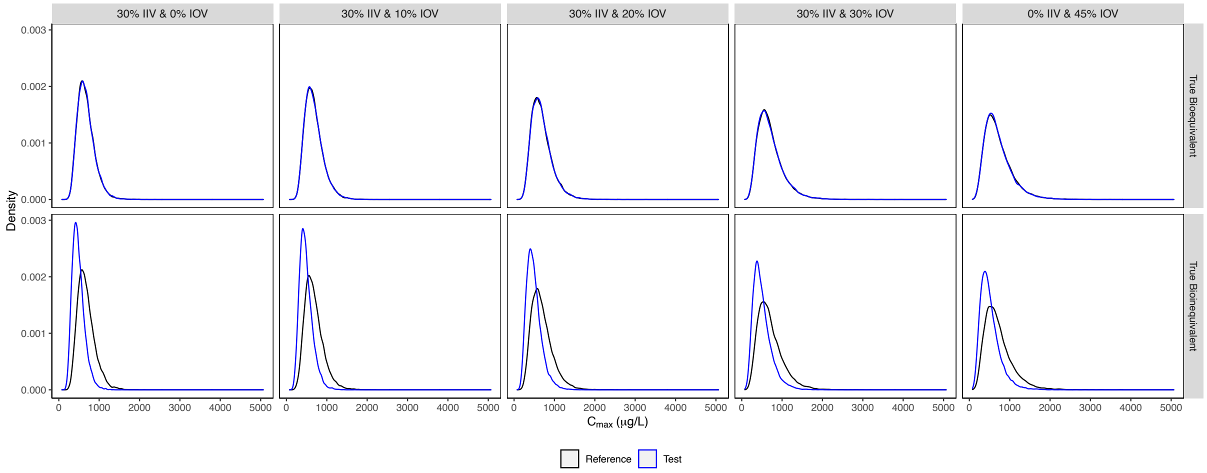

Figure S.62. Distribution of Untransformed C_{max}, Derived from Simulations for Studies with Variability in V

Table S.16. Descriptive Statistics of t_{max} (h) Derived from Simulated True Bioequivalent Studies, with Variability in V

	30% IIV 0% IOV		30% IIV 10% IOV		30% IIV 20% IOV		30% IIV 30% IOV		0% IIV 45% IOV	
	Reference	Test	Reference	Test	Reference	Test	Reference	Test	Reference	Test
n	21000	21000	21000	21000	21000	21000	21000	21000	21000	21000
Minimum	0.75	1.00	0.75	0.75	0.75	0.75	0.75	0.75	0.75	0.75
Q1	1.75	1.75	1.75	1.75	1.75	1.75	1.75	1.75	1.75	1.75
Median	2.25	2.25	2.25	2.25	2.25	2.25	2.25	2.25	2.25	2.25
Q3	2.50	2.50	2.50	2.50	2.50	2.50	2.50	2.50	2.50	2.50
Maximum	4.00	4.00	4.00	4.00	4.00	4.00	4.00	4.00	4.00	4.00

n: Number of observations, Q1: 1st (25%) quartile, Q3: 3rd (75%) quartile

Table S.17. Descriptive Statistics of t_{max} (h) Derived from Simulated True Bioinequivalent Studies, with Variability in V

	30% IIV 0% IOV		30% IIV 10% IOV		30% IIV 20% IOV		30% IIV 30% IOV		0% IIV 45% IOV	
	Reference	Test	Reference	Test	Reference	Test	Reference	Test	Reference	Test
n	21000	21000	21000	21000	21000	21000	21000	21000	21000	21000
Minimum	0.75	1.75	0.75	1.75	0.75	1.75	0.75	1.75	0.75	1.50
Q1	1.75	3.25	1.75	3.25	1.75	3.25	1.75	3.25	1.75	3.25
Median	2.25	3.50	2.25	3.50	2.25	3.50	2.25	3.50	2.25	3.50
Q3	2.50	4.00	2.50	4.00	2.50	4.00	2.50	4.00	2.50	4.00
Maximum	4.00	8.00	4.00	8.00	4.00	8.00	4.00	8.00	4.00	8.00

n: Number of observations, Q1: 1st (25%) quartile, Q3: 3rd (75%) quartile

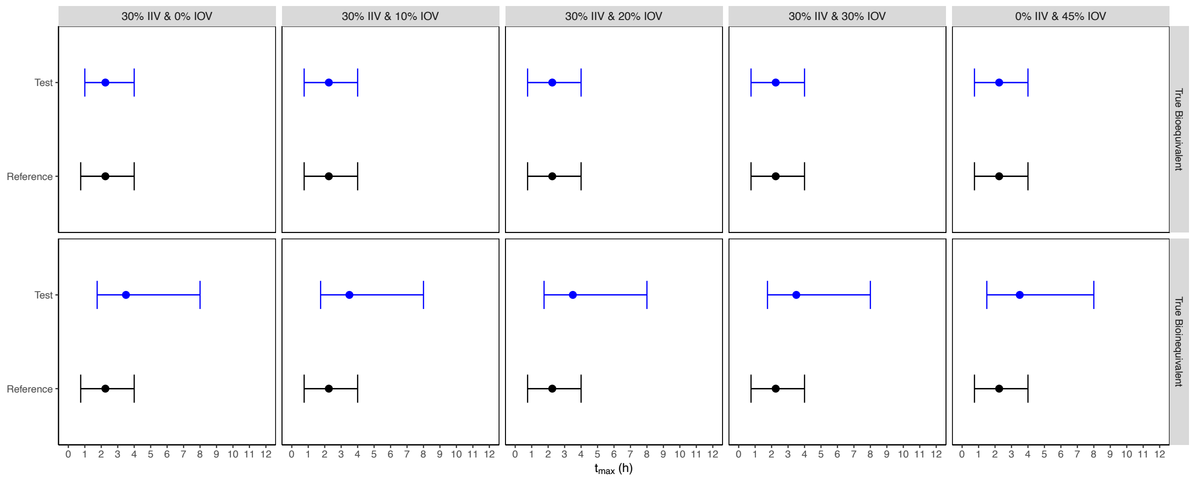

Figure S.63. Distribution of Untransformed t_{max}, Derived from Simulations for Studies with Variability in V

Table S.18. Descriptive Statistics of AUC_{0-t} (h) Derived from Simulated True Bioequivalent Studies, with Variability in V

	30% IIV 0% IOV		30% IIV 10% IOV		30% IIV 20% IOV		30% IIV 30% IOV		0% IIV 45% IOV	
	Reference	Test	Reference	Test	Reference	Test	Reference	Test	Reference	Test
n	21000	21000	21000	21000	21000	21000	21000	21000	21000	21000
G_{Mean}	4941.8	4942.68	4960.88	4958.66	4915.06	4926.42	4961.97	4952.85	4968.16	4935.19
GSD	1.35	1.35	1.38	1.38	1.43	1.43	1.53	1.53	1.57	1.57
GCV%	30.9	30.9	32.8	32.9	37.0	37.2	44.4	44.8	47.7	47.6
G_{mean} 95% CI	4921.64 – 4962.04	4922.54 – 4962.89	4939.48 – 4982.38	4937.23 – 4980.18	4891.30 – 4938.94	4902.49 – 4950.48	4933.60 – 4990.50	4924.28 – 4981.58	4937.82 – 4998.67	4905.15 – 4965.42
A_{Mean}	5172.37	5172.76	5220.35	5219.17	5240.6	5256.59	5428.71	5428.23	5503.81	5464.08
SD	1596.59	1595.84	1704.24	1709.46	1937.55	1958.56	2412.07	2442.9	2615.96	2590.17
SE	11.02	11.01	11.76	11.8	13.37	13.52	16.64	16.86	18.05	17.87
CV%	30.9	30.9	32.6	32.8	37	37.3	44.4	45	47.5	47.4
Variance	2549099.29	2546698.93	2904439.93	2922249.6	3754082.15	3835964.9	5818095.45	5967772.18	6843223.15	6708975.32
A_{mean} 95% CI	5150.77 – 5193.96	5151.17 – 5194.34	5197.30 – 5243.40	5196.05 – 5242.29	5214.39 – 5266.80	5230.09 – 5283.08	5396.09 – 5461.34	5395.19 – 5461.27	5468.42 – 5539.19	5429.05 – 5499.12
Minimum	1465.52	1466.27	1388.32	1161.27	1251.35	1114.54	1015.02	919.74	744.89	726.78
Q1	4037.21	4033.31	3995.27	3996.38	3849.27	3862.07	3740.45	3709.39	3659.69	3643.75
Median	4952	4953.04	4963.63	4959.14	4923.76	4936.2	4977.4	4948.06	4952.29	4928.41
Q3	6069.38	6042.51	6173.16	6154.16	6256.49	6281.42	6570.91	6587.53	6755.41	6687.64
Maximum	16615.89	16977.92	16205.64	18499.28	22144.15	18533.65	27547.43	41583.23	28818.18	27903.05

n: Number of observations, G_{mean}: Geometric mean, GSD: Geometric standard deviation, GCV%: Geometric coefficient of variation, G_{mean} 95% CI: 95% confidence interval of the geometric mean, A_{mean}: Arithmetic mean, SD: Standard deviation, SD: Standard error, CV%: Coefficient of variation, A_{mean} 95% CI: 95% confidence interval of the arithmetic mean, Q1: 1st (25%) quartile, Q3: 3rd (75%) quartile

Table S.19. Descriptive Statistics of AUC_{0-t} (h) Derived from Simulated True Bioinequivalent Studies, with Variability in V

	**30% IIV 0% IOV**		**30% IIV 10% IOV**		**30% IIV 20% IOV**		**30% IIV 30% IOV**		**0% IIV 45% IOV**	
	**Reference**	**Test**	**Reference**	**Test**	**Reference**	**Test**	**Reference**	**Test**	**Reference**	**Test**
n	21000	21000	21000	21000	21000	21000	21000	21000	21000	21000
G_{Mean}	4941.8	4942.68	4960.88	4958.66	4915.06	4926.42	4961.97	4952.85	4968.16	4935.19
GSD	1.35	1.35	1.38	1.38	1.43	1.43	1.53	1.53	1.57	1.57
GCV%	30.9	30.9	32.8	32.9	37.0	37.2	44.4	44.8	47.7	47.6
G_{mean} 95% CI	4921.64 – 4962.04	4922.54 – 4962.89	4939.48 – 4982.38	4937.23 – 4980.18	4891.30 – 4938.94	4902.49 – 4950.48	4933.60 – 4990.50	4924.28 – 4981.58	4937.82 – 4998.67	4905.15 – 4965.42
A_{Mean}	5172.37	5172.76	5220.35	5219.17	5240.6	5256.59	5428.71	5428.23	5503.81	5464.08
SD	1596.59	1595.84	1704.24	1709.46	1937.55	1958.56	2412.07	2442.9	2615.96	2590.17
SE	11.02	11.01	11.76	11.8	13.37	13.52	16.64	16.86	18.05	17.87
CV%	30.9	30.9	32.6	32.8	37.0	37.3	44.4	45.0	47.5	47.4
Variance	2549099.29	2546698.93	2904439.93	2922249.6	3754082.15	3835964.9	5818095.45	5967772.18	6843223.15	6708975.32
A_{mean} 95% CI	5150.77 – 5193.96	5151.17 – 5194.34	5197.30 – 5243.40	5196.05 – 5242.29	5214.39 – 5266.80	5230.09 – 5283.08	5396.09 – 5461.34	5395.19 – 5461.27	5468.42 – 5539.19	5429.05 – 5499.12
Minimum	1465.52	1466.27	1388.32	1161.27	1251.35	1114.54	1015.02	919.74	744.89	726.78
Q1	4037.21	4033.31	3995.27	3996.38	3849.27	3862.07	3740.45	3709.39	3659.69	3643.75
Median	4952	4953.04	4963.63	4959.14	4923.76	4936.2	4977.4	4948.06	4952.29	4928.41
Q3	6069.38	6042.51	6173.16	6154.16	6256.49	6281.42	6570.91	6587.53	6755.41	6687.64
Maximum	16615.89	16977.92	16205.64	18499.28	22144.15	18533.65	27547.43	41583.23	28818.18	27903.05

n: Number of observations, G_{mean}: Geometric mean, GSD: Geometric standard deviation, GCV%: Geometric coefficient of variation, G_{mean} 95% CI: 95% confidence interval of the geometric mean, A_{mean}: Arithmetic mean, SD: Standard deviation, SD: Standard error, CV%: Coefficient of variation, A_{mean} 95% CI: 95% confidence interval of the arithmetic mean, Q1: 1st (25%) quartile, Q3: 3rd (75%) quartile

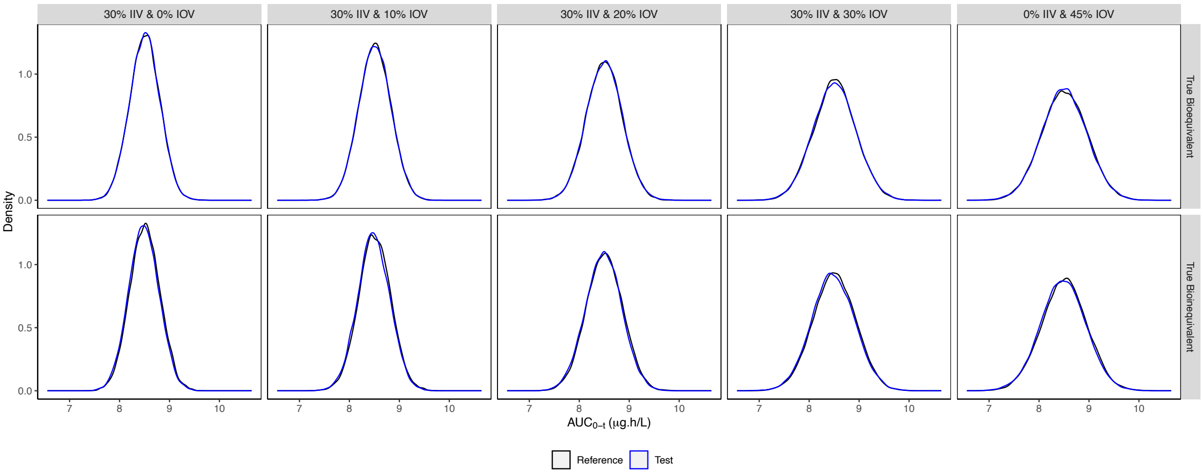

Figure S.64. Distribution of *Ln*-Transformed $AUC_{0\text{-}t}$, Derived from Simulations for Studies with Variability in V

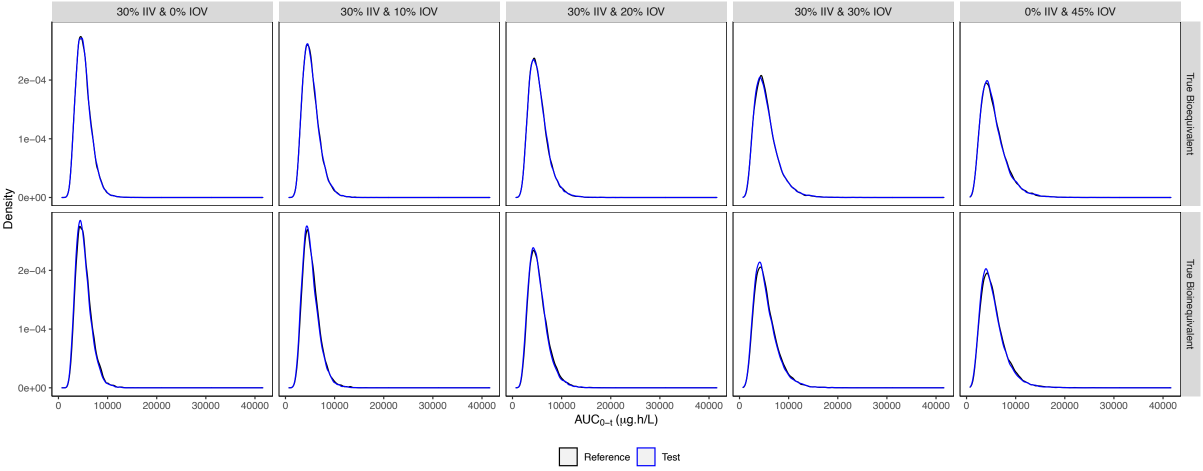

Figure S.65. Distribution of Untransformed $AUC_{0\text{-}t}$, Derived from Simulations for Studies with Variability in V

Appendix SA.3.4. Simulated Pilot Studies Results

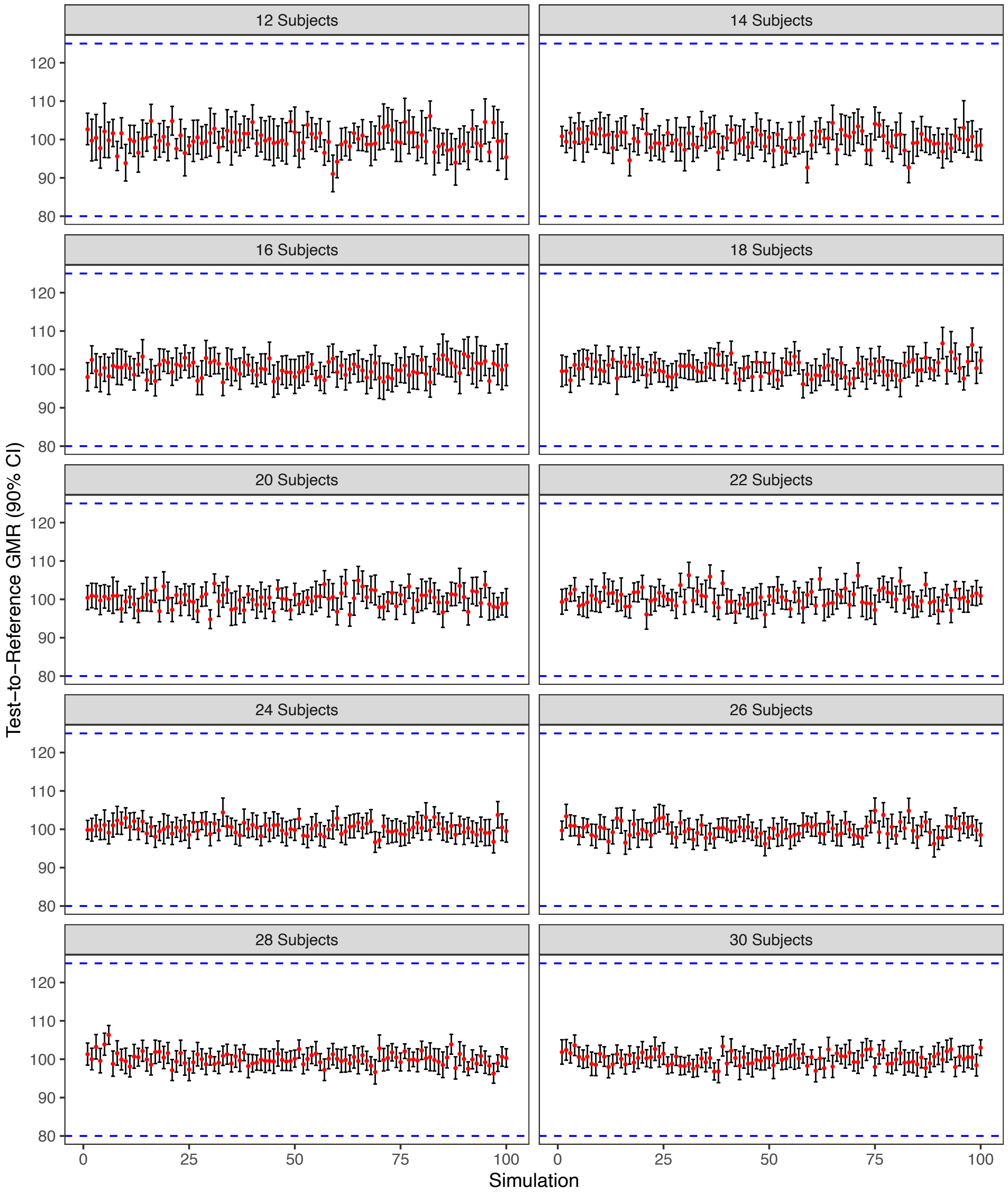

Figure S.66. Average Bioequivalence Analysis Results (Test-to-Reference GMR and Corresponding 90% CI) for C_{max} Derived from True Bioequivalent Simulations with 30% IIV & 0% IOV in V

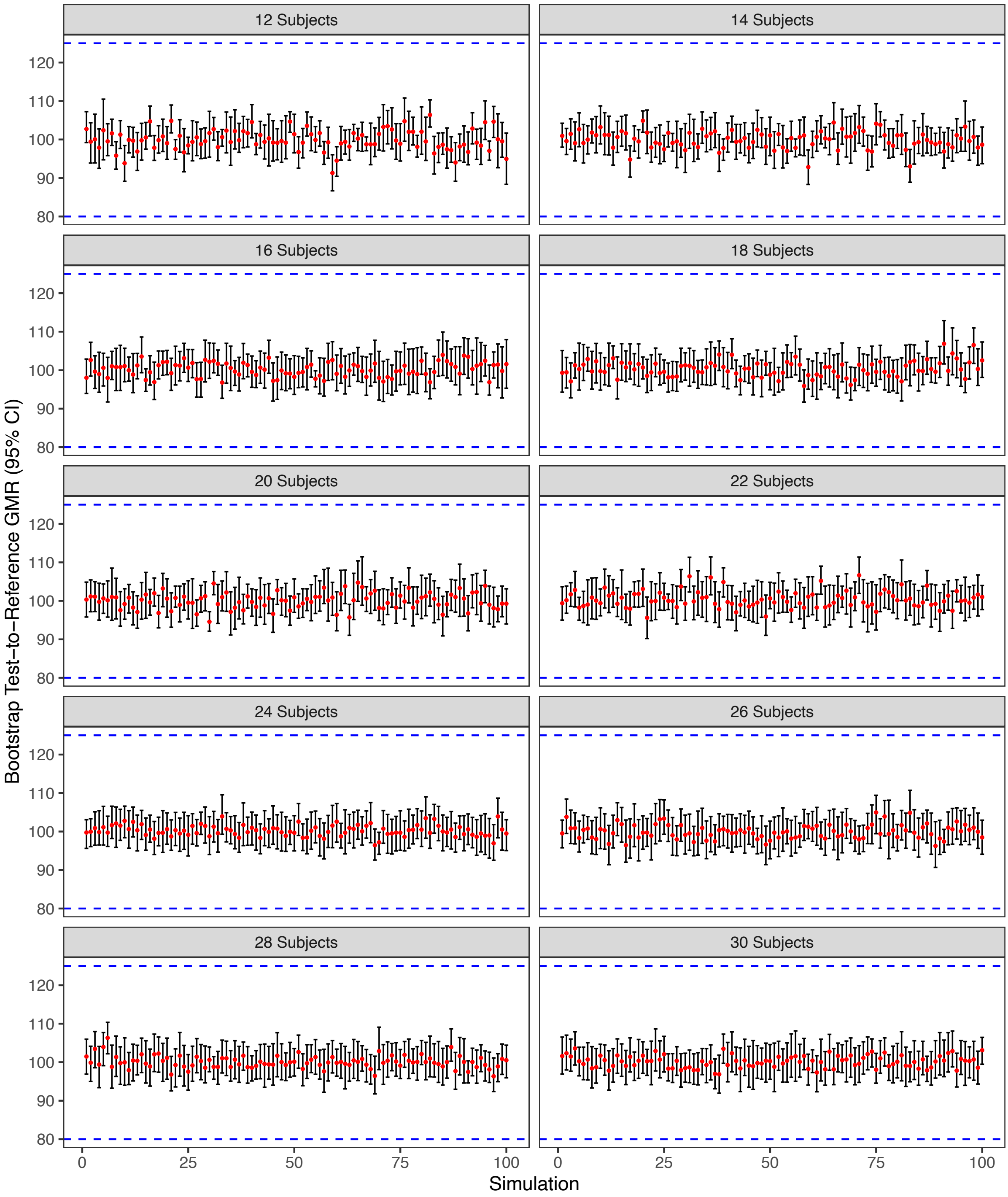

Figure S.67. Bootstrap Bioequivalence Analysis Results (Test-to-Reference GMR and Corresponding 95% CI) for C_{max} Derived from True Bioequivalent Simulations with 30% IIV & 0% IOV in V

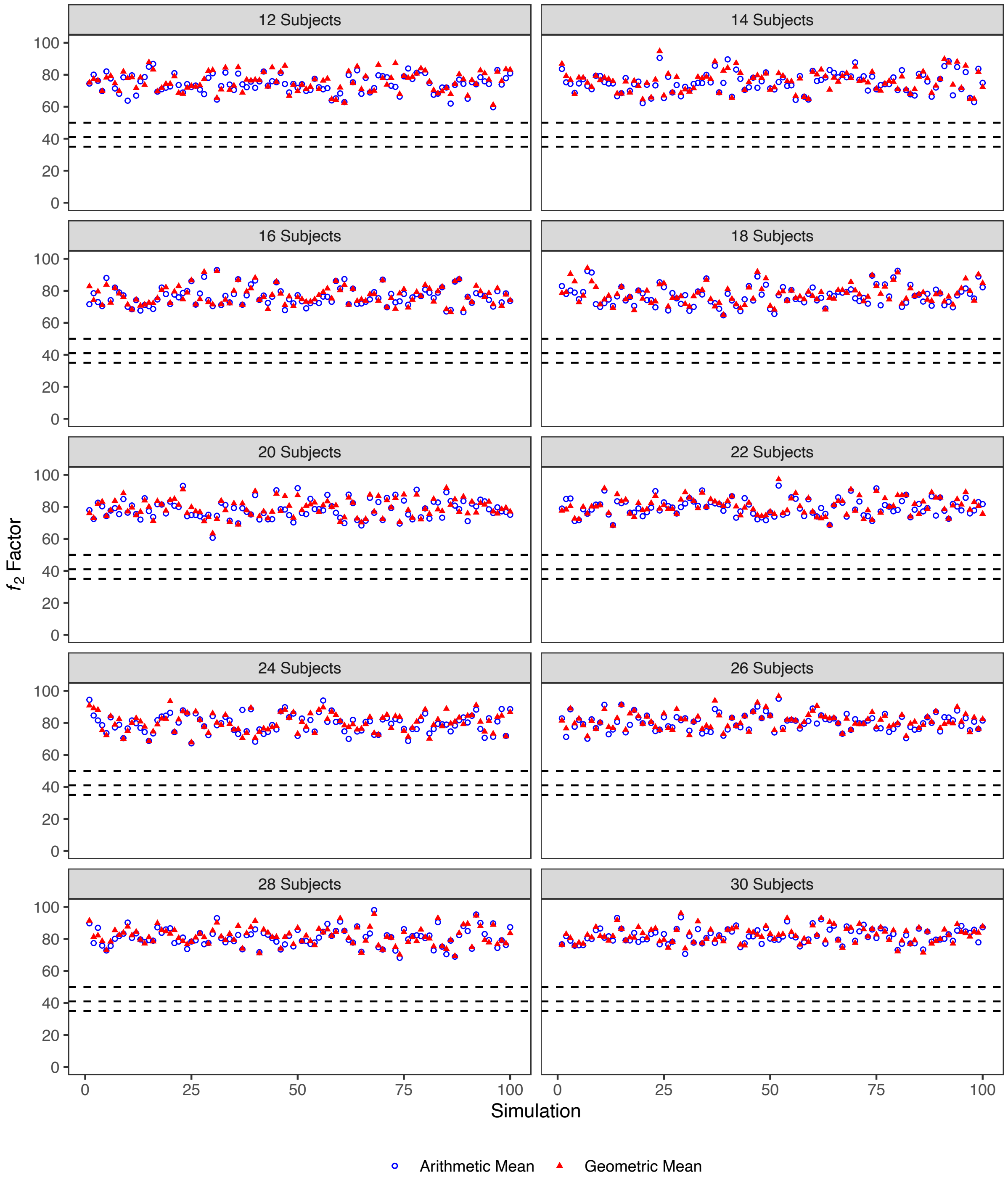

Figure S.68. Similarity f_2 Factor Results, Derived from Arithmetic and Geometric Mean Concentration-Time Profiles Until the Reference C_{max}, Derived from True Bioequivalent Simulations with 30% IIV & 0% IOV in V

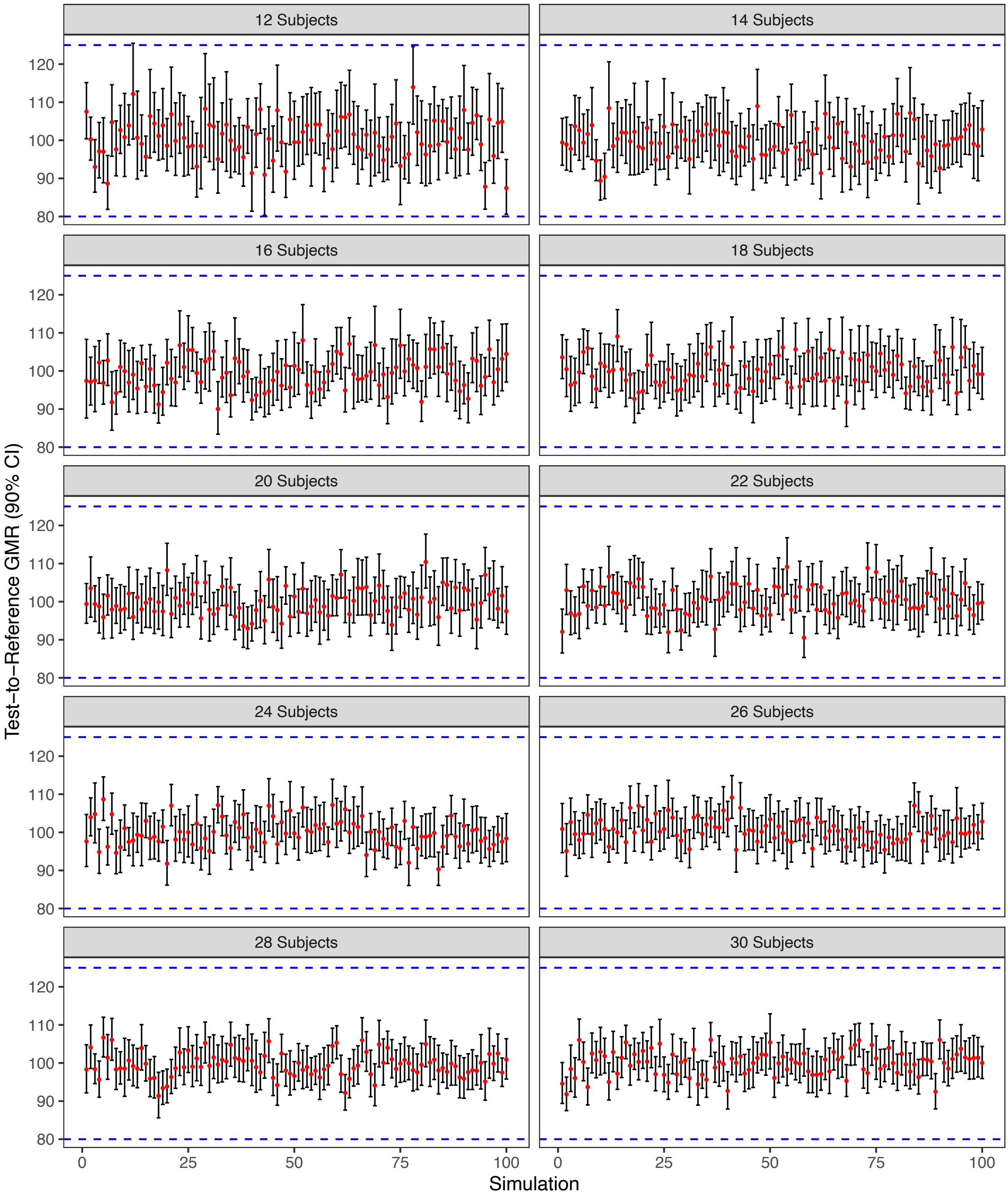

Figure S.69. Average Bioequivalence Analysis Results (Test-to-Reference GMR and Corresponding 90% CI) for C_{max} Derived from True Bioequivalent Simulations with 30% IIV & 10% IOV in V

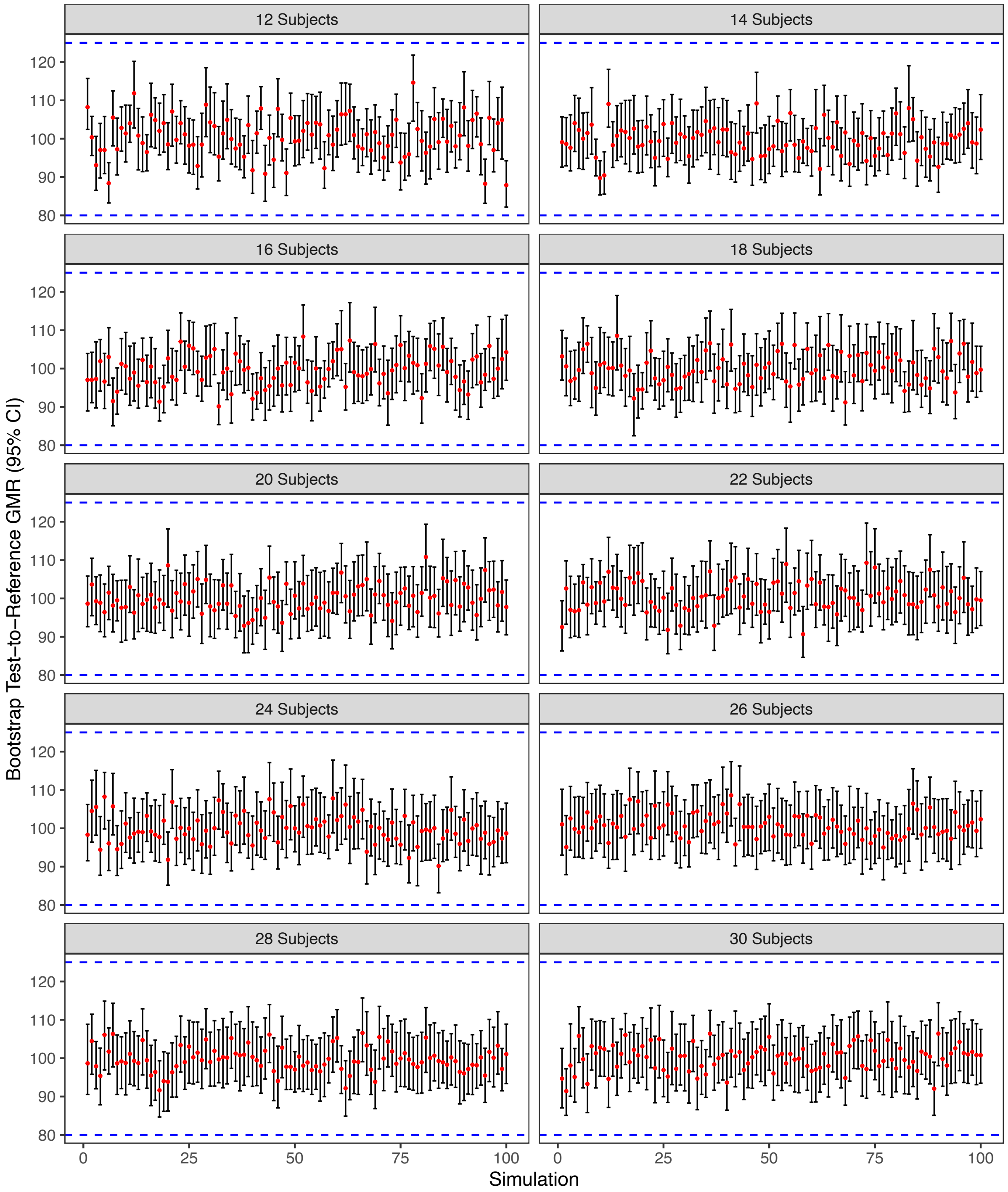

Figure S.70. Bootstrap Bioequivalence Analysis Results (Test-to-Reference GMR and Corresponding 95% CI) for C_{max} Derived from True Bioequivalent Simulations with 30% IIV & 10% IOV in V

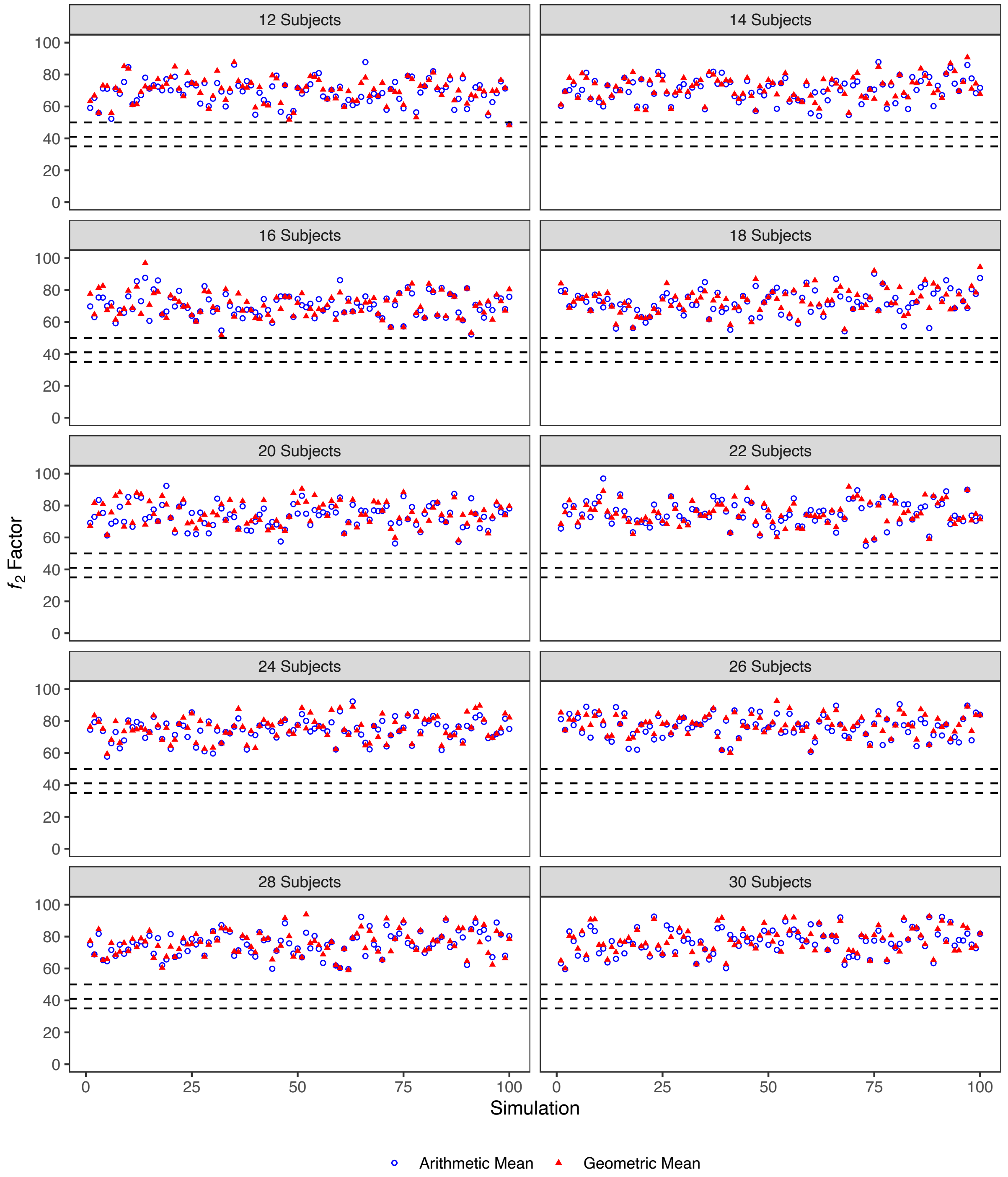

Figure S.71. Similarity f_2 Factor Results, Derived from Arithmetic and Geometric Mean Concentration-Time Profiles Until the Reference C_{max}, Derived from True Bioequivalent Simulations with 30% IIV & 10% IOV in V

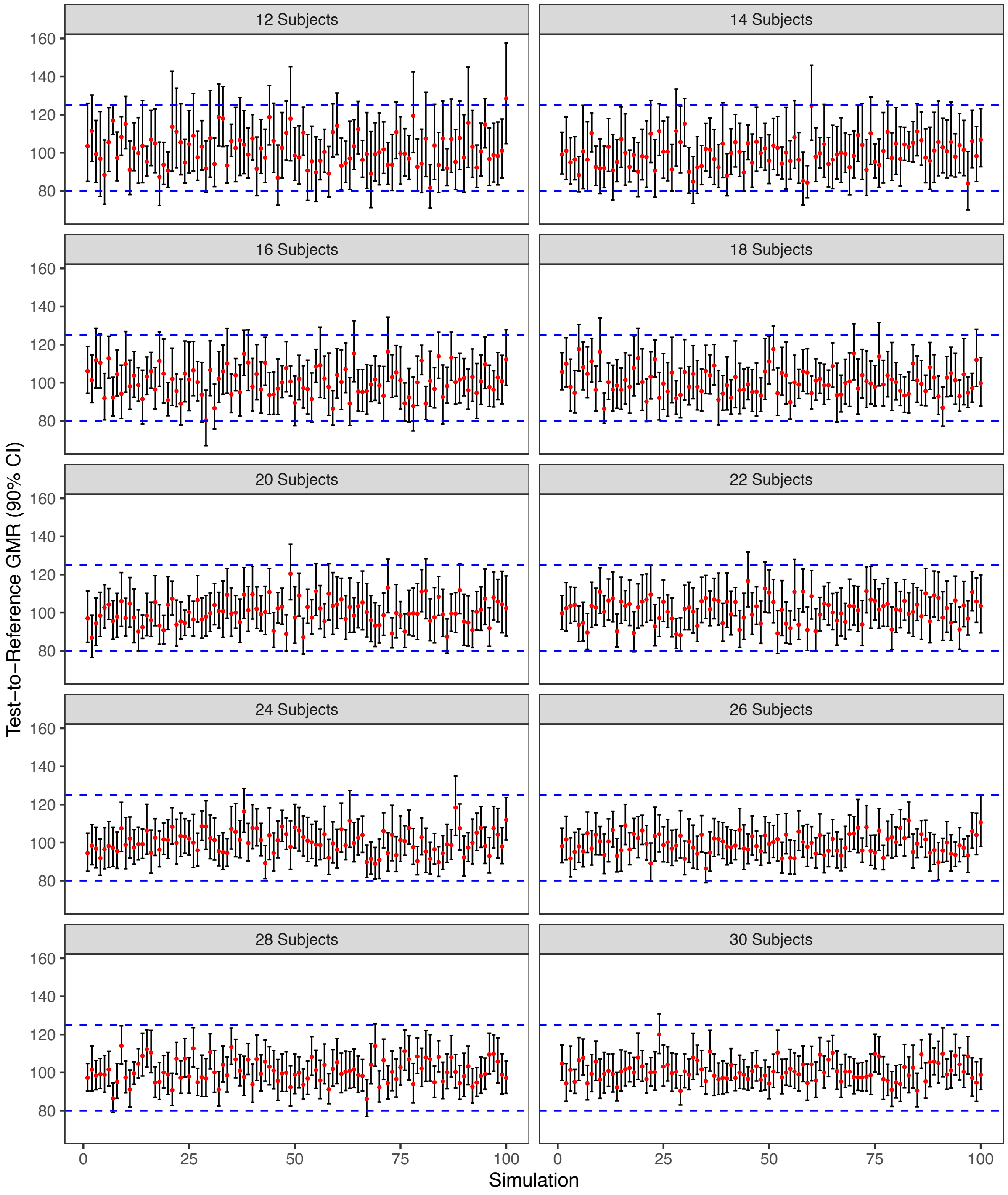

Figure S.72. Average Bioequivalence Analysis Results (Test-to-Reference GMR and Corresponding 90% CI) for C_{max} Derived from True Bioequivalent Simulations with 30% IIV & 20% IOV in V

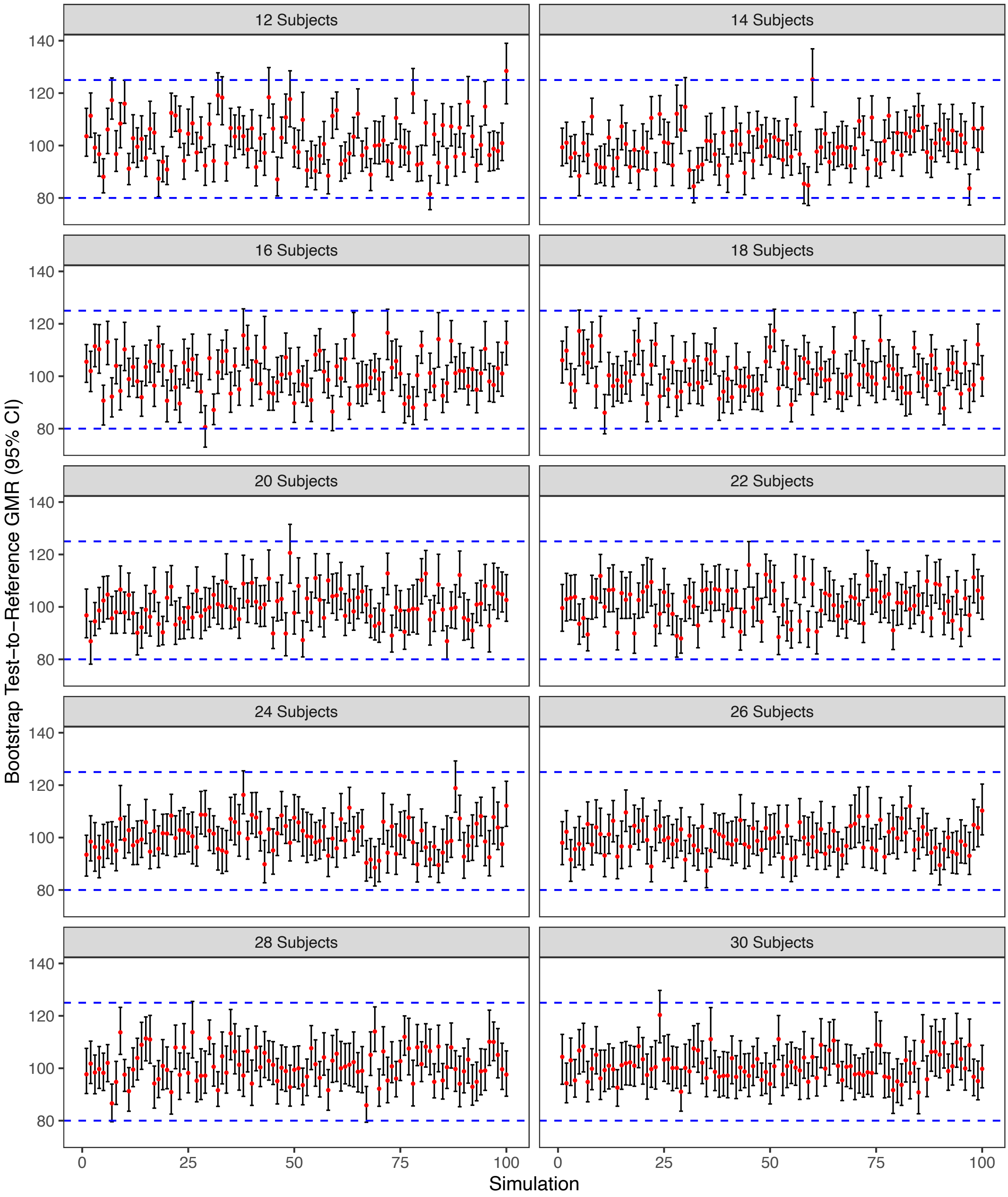

Figure S.73. Bootstrap Bioequivalence Analysis Results (Test-to-Reference GMR and Corresponding 95% CI) for C_{max} Derived from True Bioequivalent Simulations with 30% IIV & 20% IOV in V

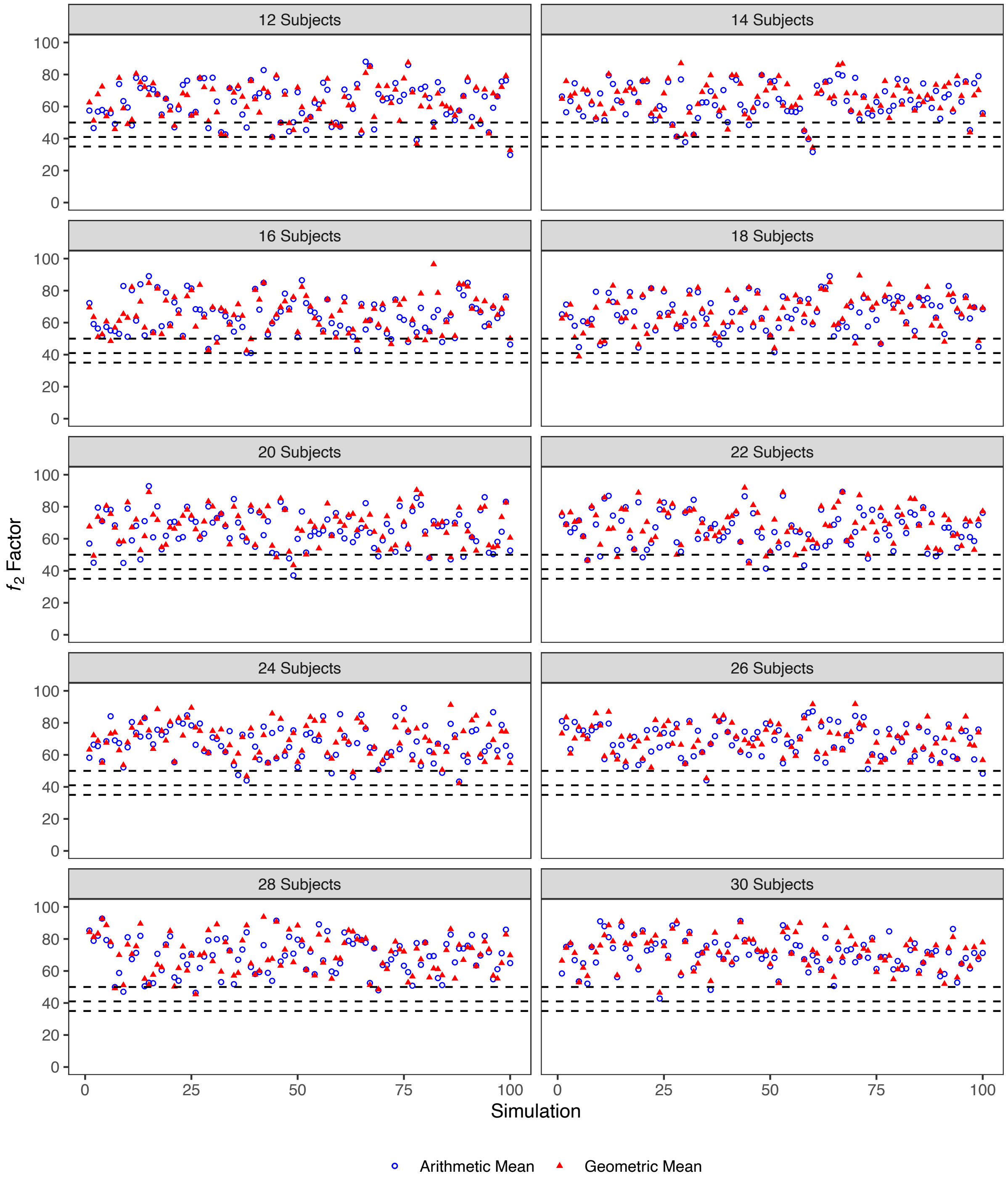

Figure S.74. Similarity f_2 Factor Results, Derived from Arithmetic and Geometric Mean Concentration-Time Profiles Until the Reference C_{max}, Derived from True Bioequivalent Simulations with 30% IIV & 20% IOV in V

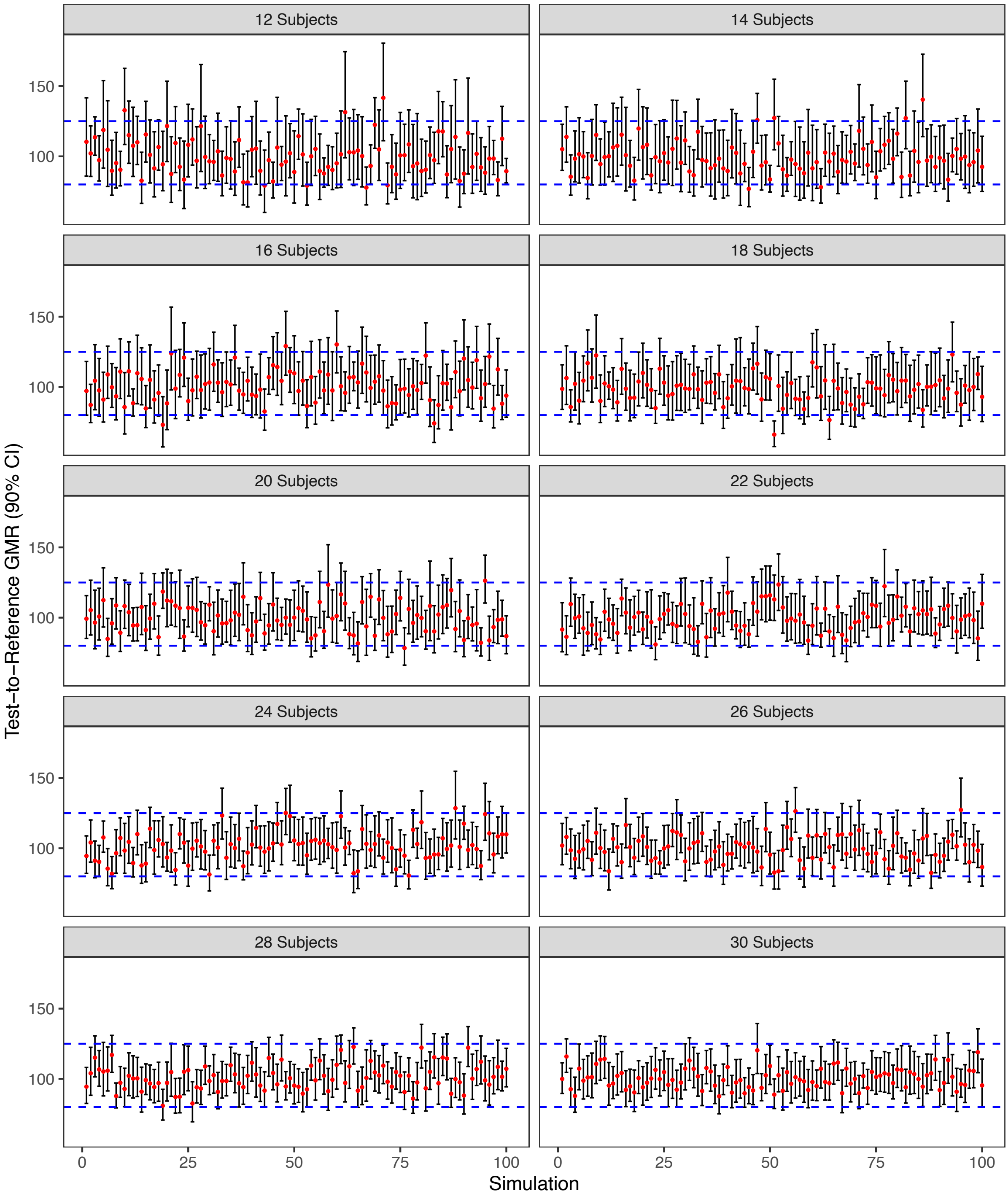

Figure S.75. Average Bioequivalence Analysis Results (Test-to-Reference GMR and Corresponding 90% CI) for C_{max} Derived from True Bioequivalent Simulations with 30% IIV & 30% IOV in V

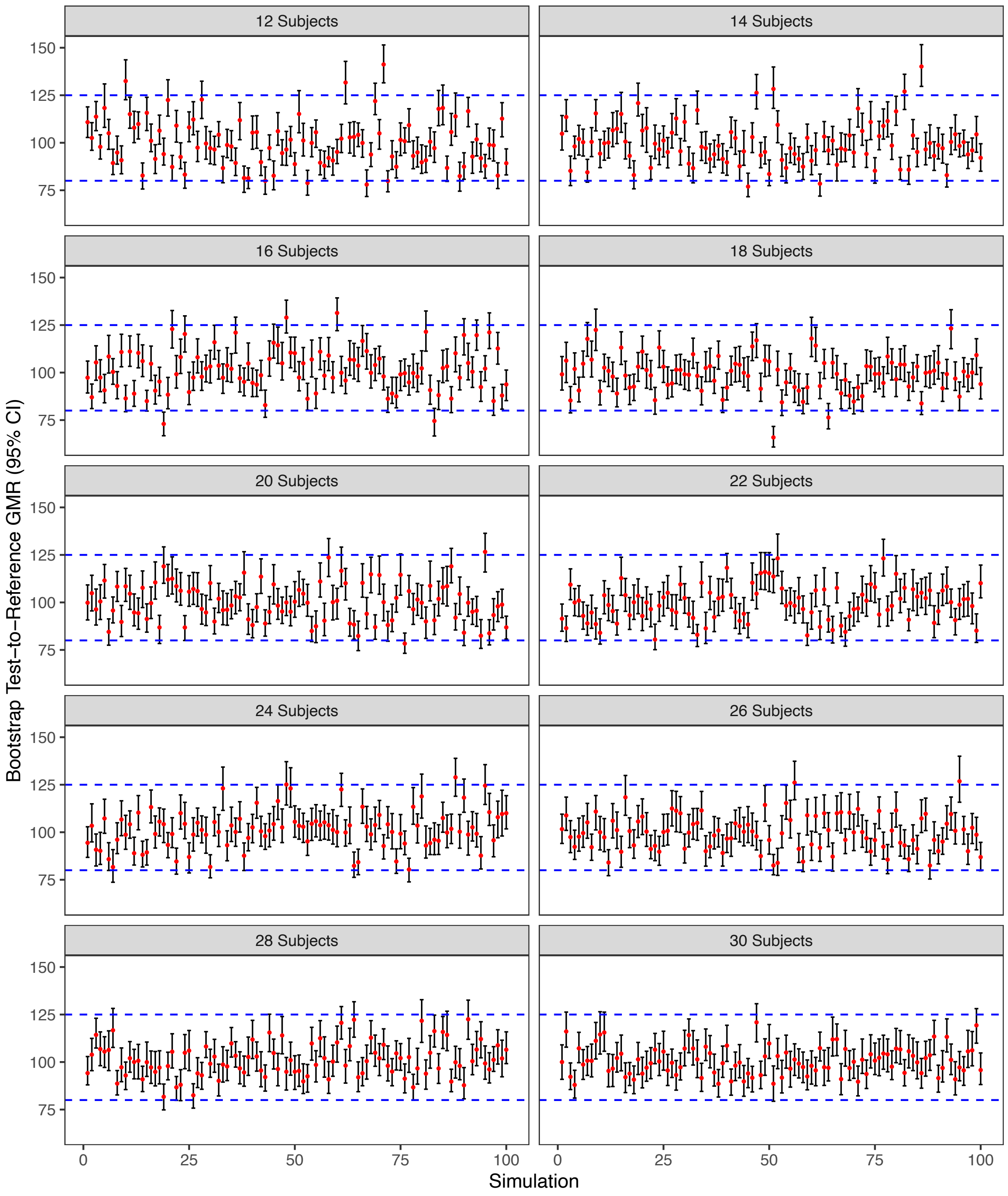

Figure S.76. Bootstrap Bioequivalence Analysis Results (Test-to-Reference GMR and Corresponding 95% CI) for C_{max} Derived from True Bioequivalent Simulations with 30% IIV & 30% IOV in V

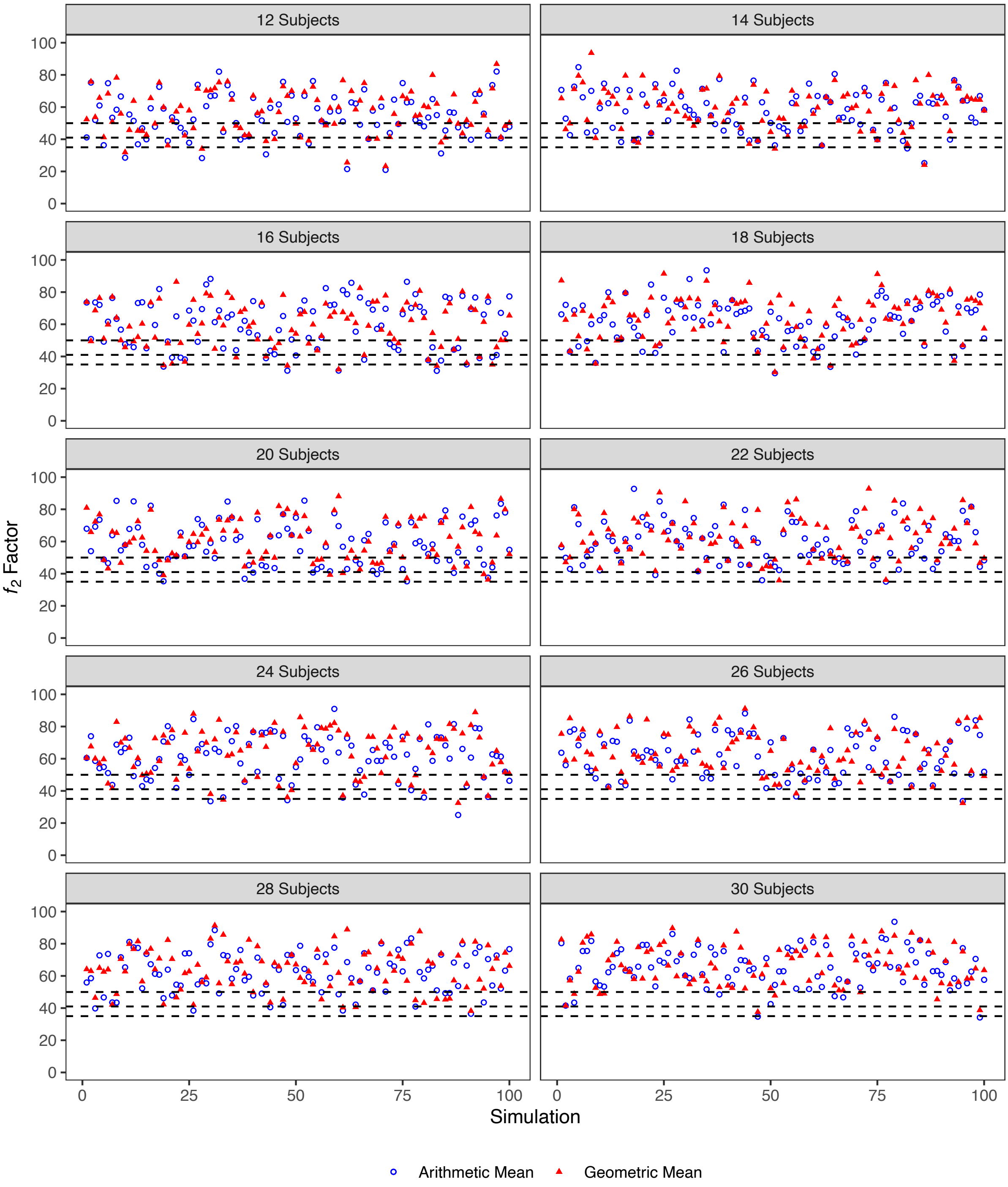

Figure S.77. Similarity f_2 Factor Results, Derived from Arithmetic and Geometric Mean Concentration-Time Profiles Until the Reference C_{max}, Derived from True Bioequivalent Simulations with 30% IIV & 30% IOV in V

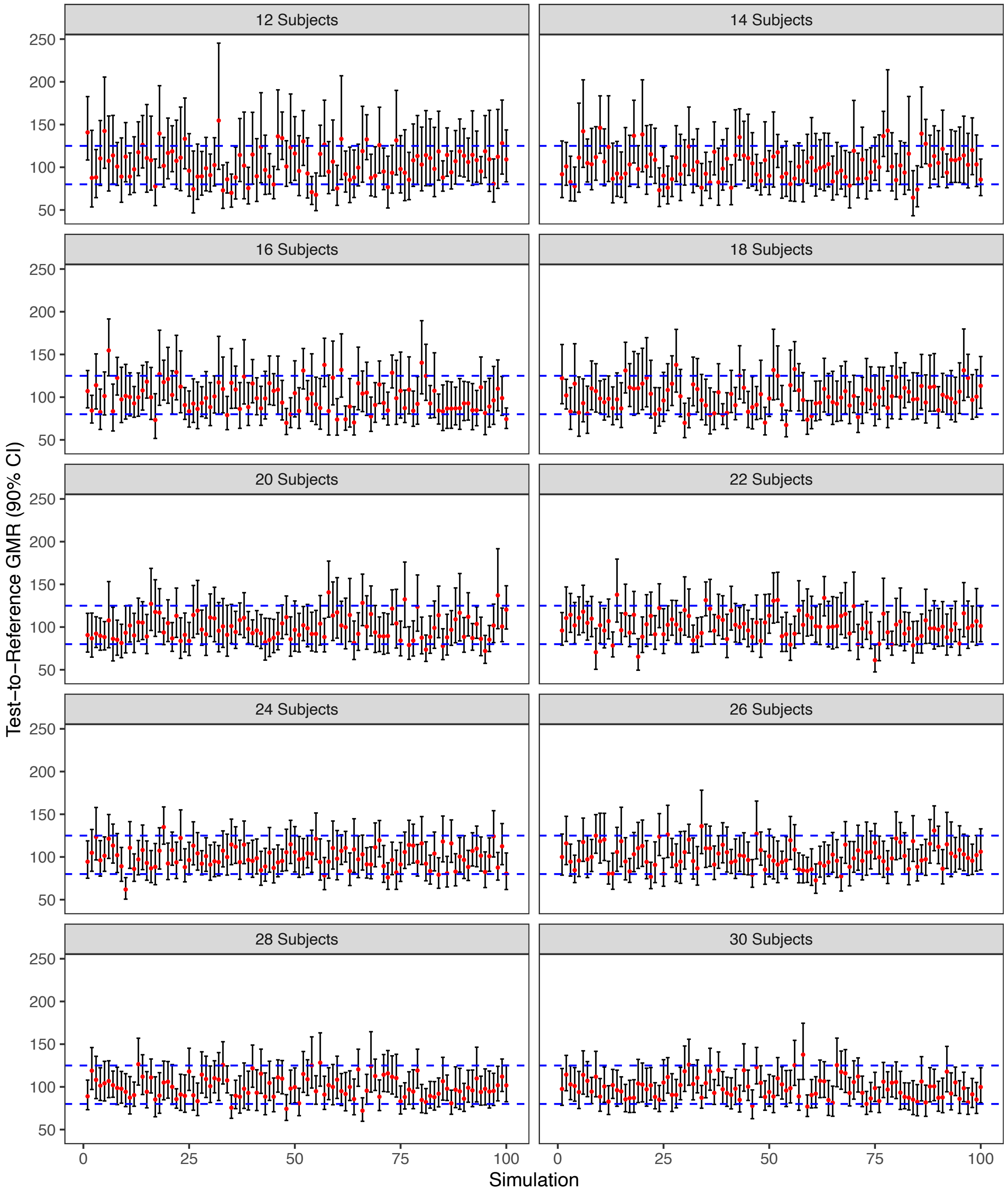

Figure S.78. Average Bioequivalence Analysis Results (Test-to-Reference GMR and Corresponding 90% CI) for C_{max} Derived from True Bioequivalent Simulations with 0% IIV & 45% IOV in V

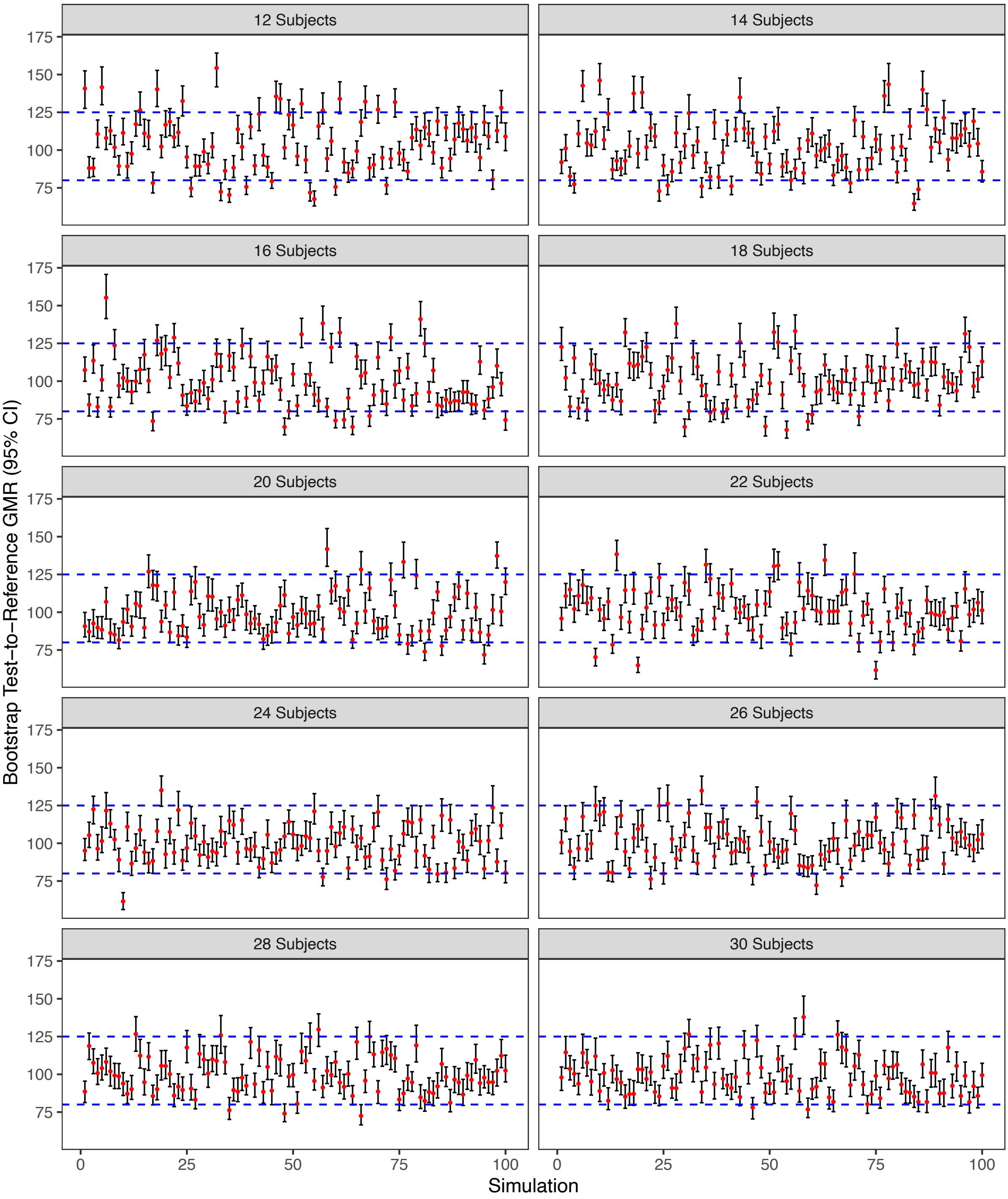

Figure S.79. Bootstrap Bioequivalence Analysis Results (Test-to-Reference GMR and Corresponding 95% CI) for C_{max} Derived from True Bioequivalent Simulations with 0% IIV & 45% IOV in V

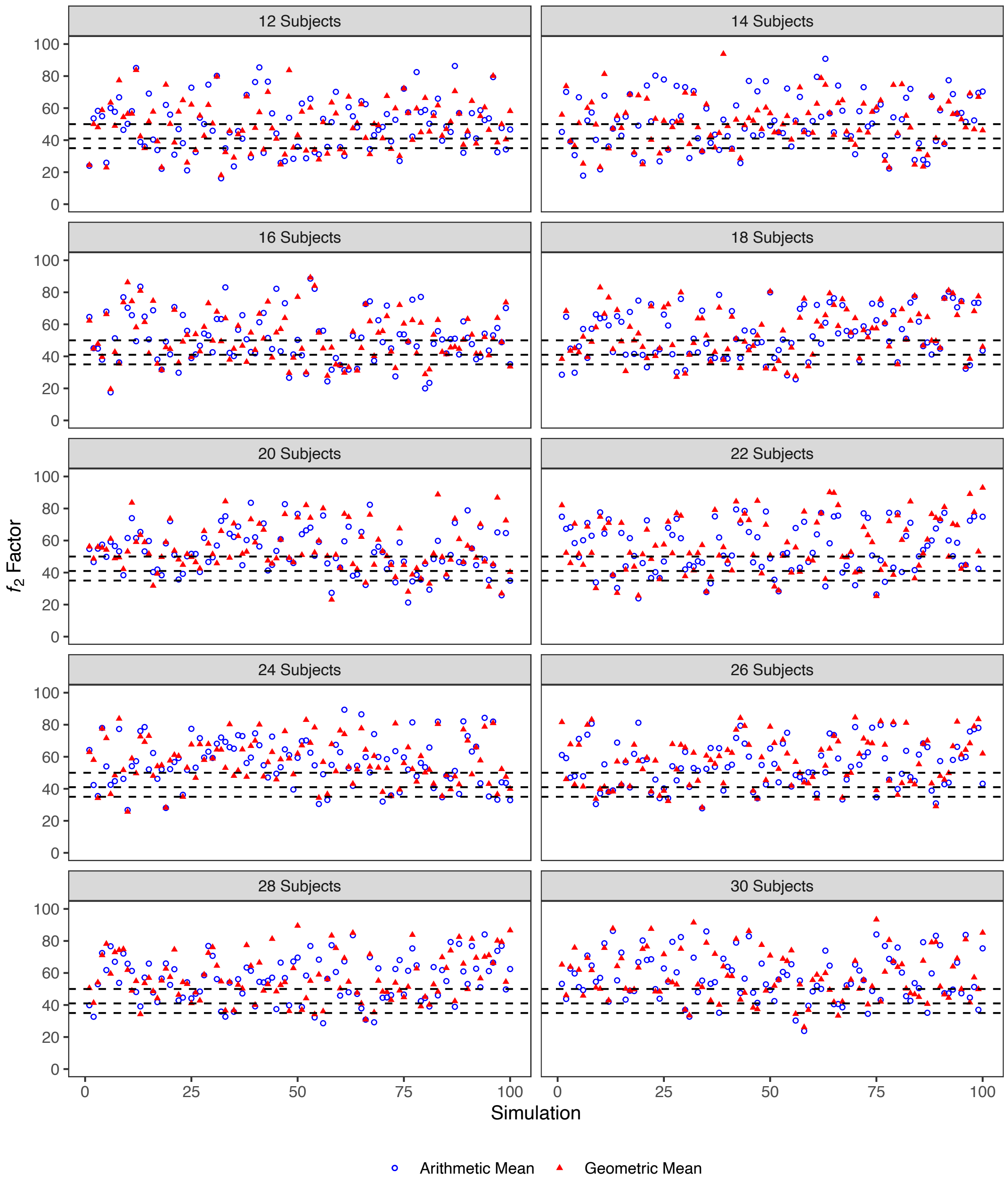

Figure S.80. Similarity f_2 Factor Results, Derived from Arithmetic and Geometric Mean Concentration-Time Profiles Until the Reference C_{max}, Derived from True Bioequivalent Simulations with 0% IIV & 45% IOV in V

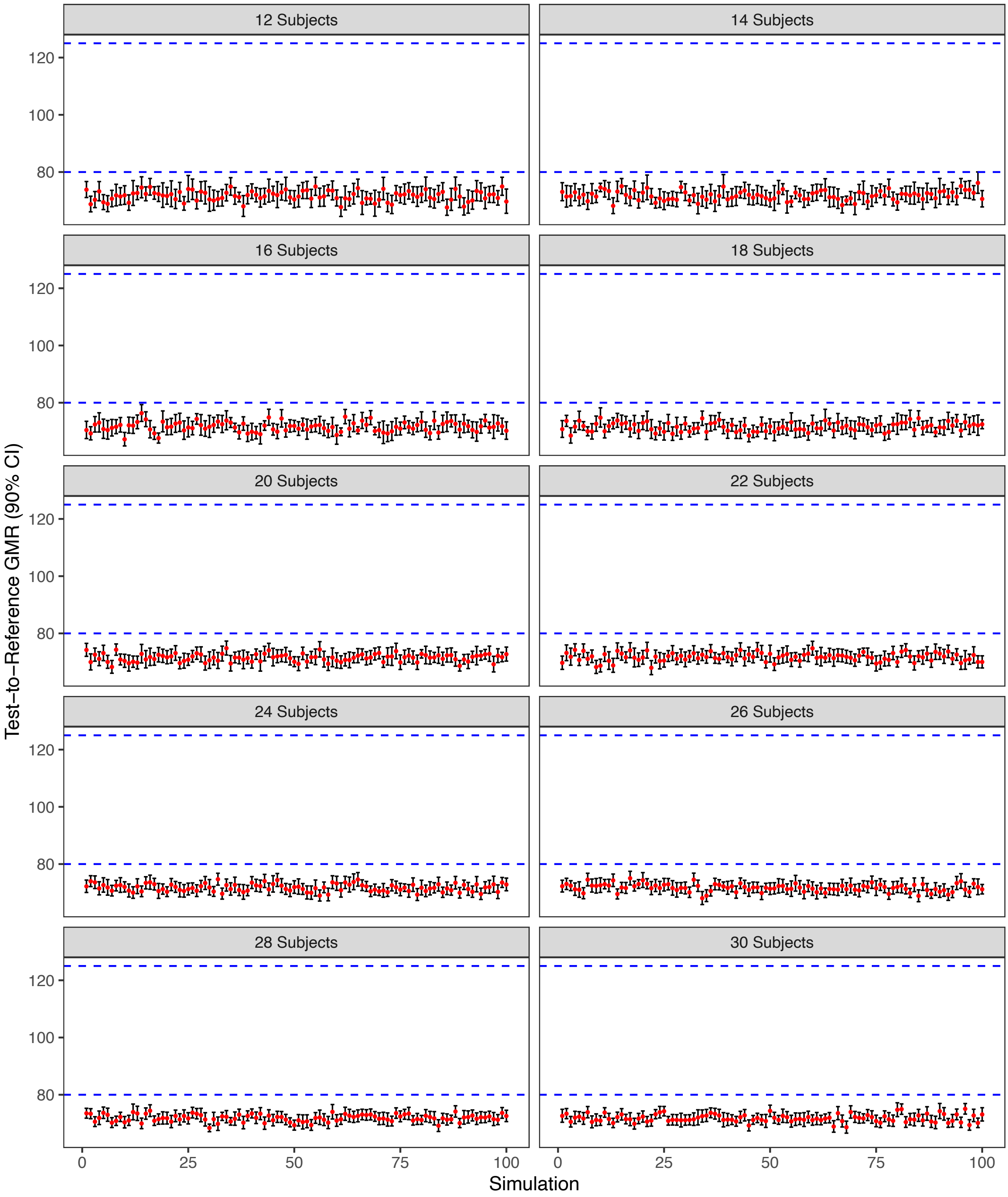

Figure S.81. Average Bioequivalence Analysis Results (Test-to-Reference GMR and Corresponding 90% CI) for C_{max} Derived from True Bioinequivalent Simulations with 30% IIV & 0% IOV in V

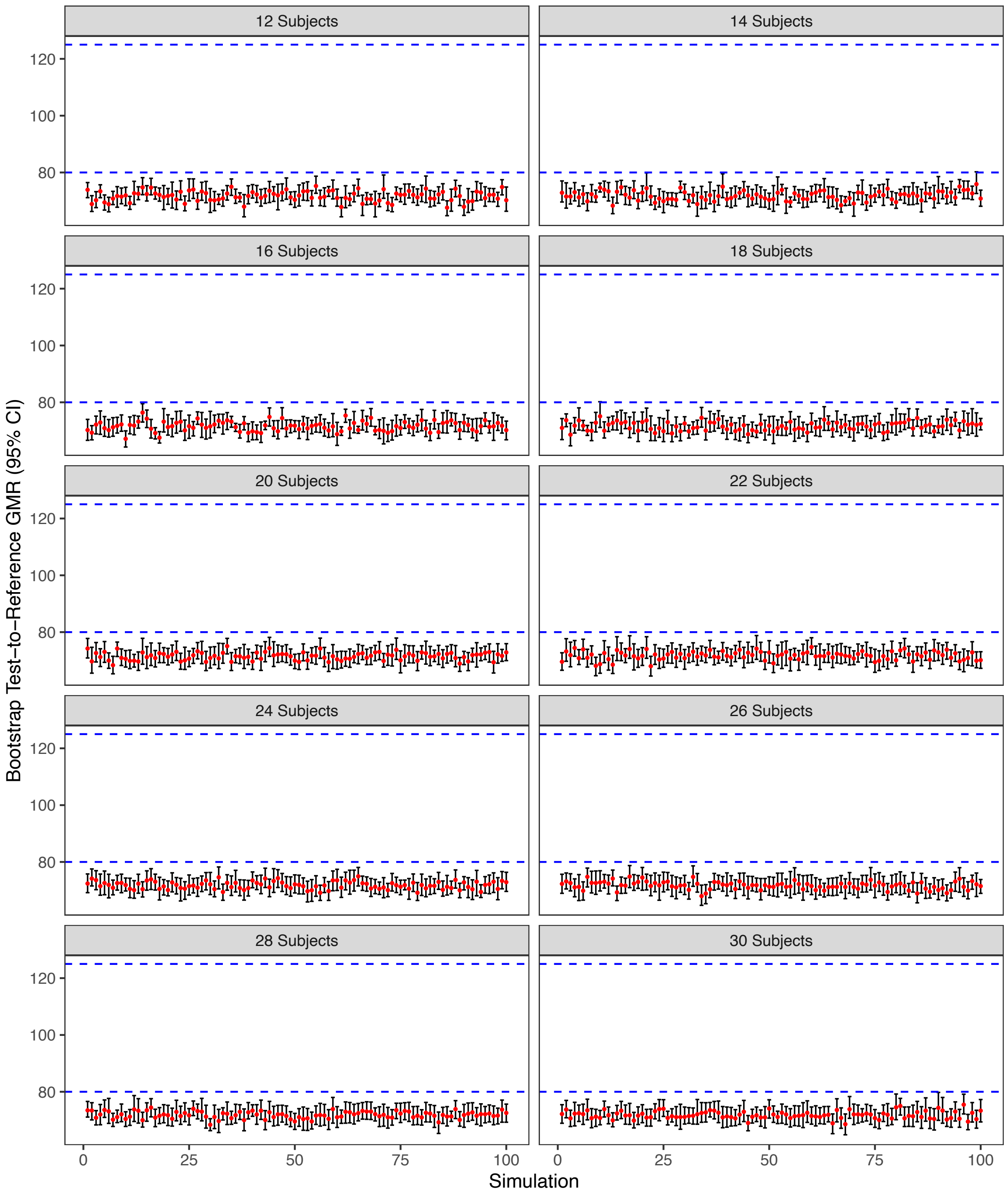

Figure S.82. Bootstrap Bioequivalence Analysis Results (Test-to-Reference GMR and Corresponding 95% CI) for C_{max} Derived from True Bioinequivalent Simulations with 30% IIV & 0% IOV in V

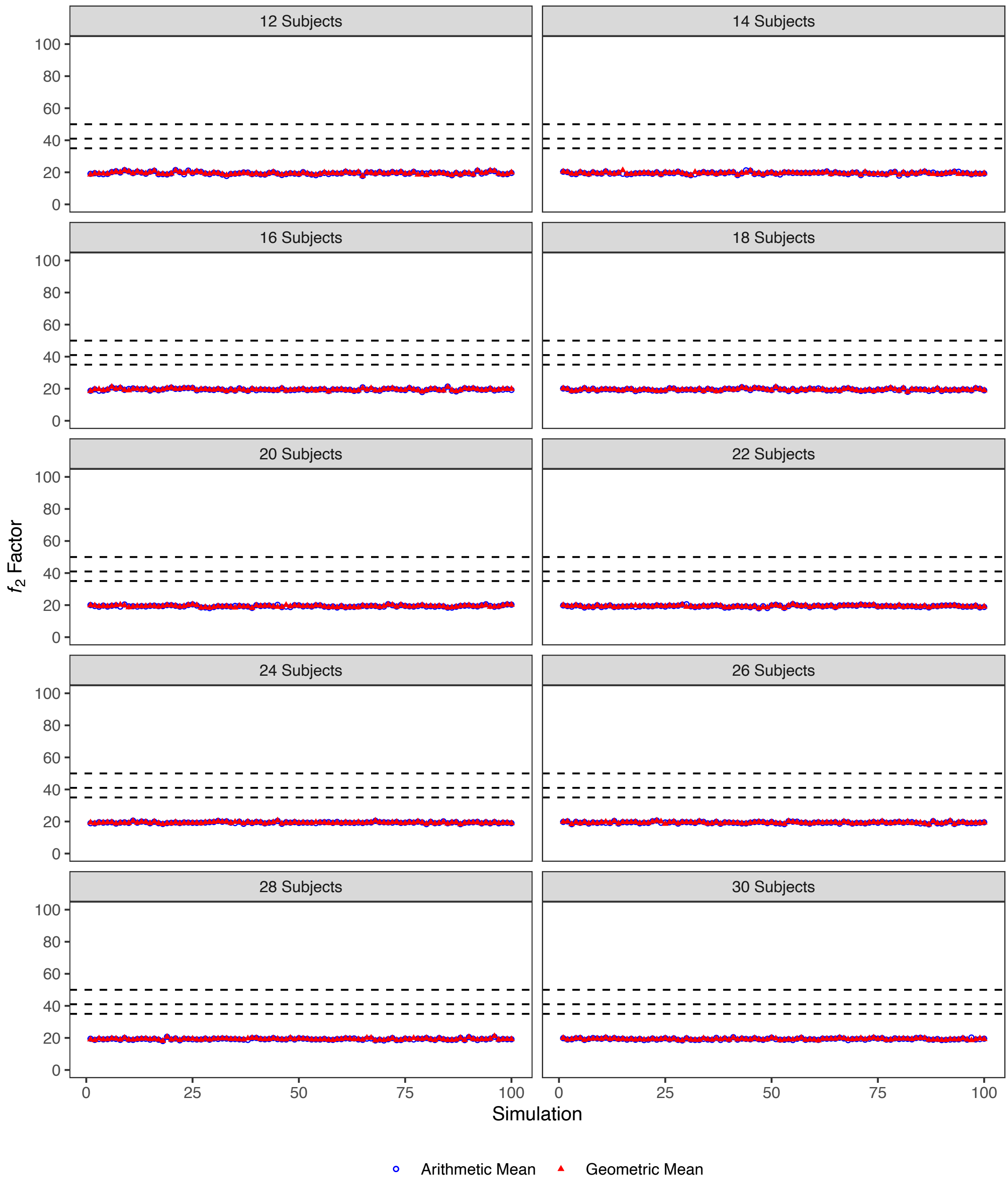

Figure S.83. Similarity f_2 Factor Results, Derived from Arithmetic and Geometric Mean Concentration-Time Profiles Until the Reference C_{max}, Derived from True Bioinequivalent Simulations with 30% IIV & 0% IOV in V

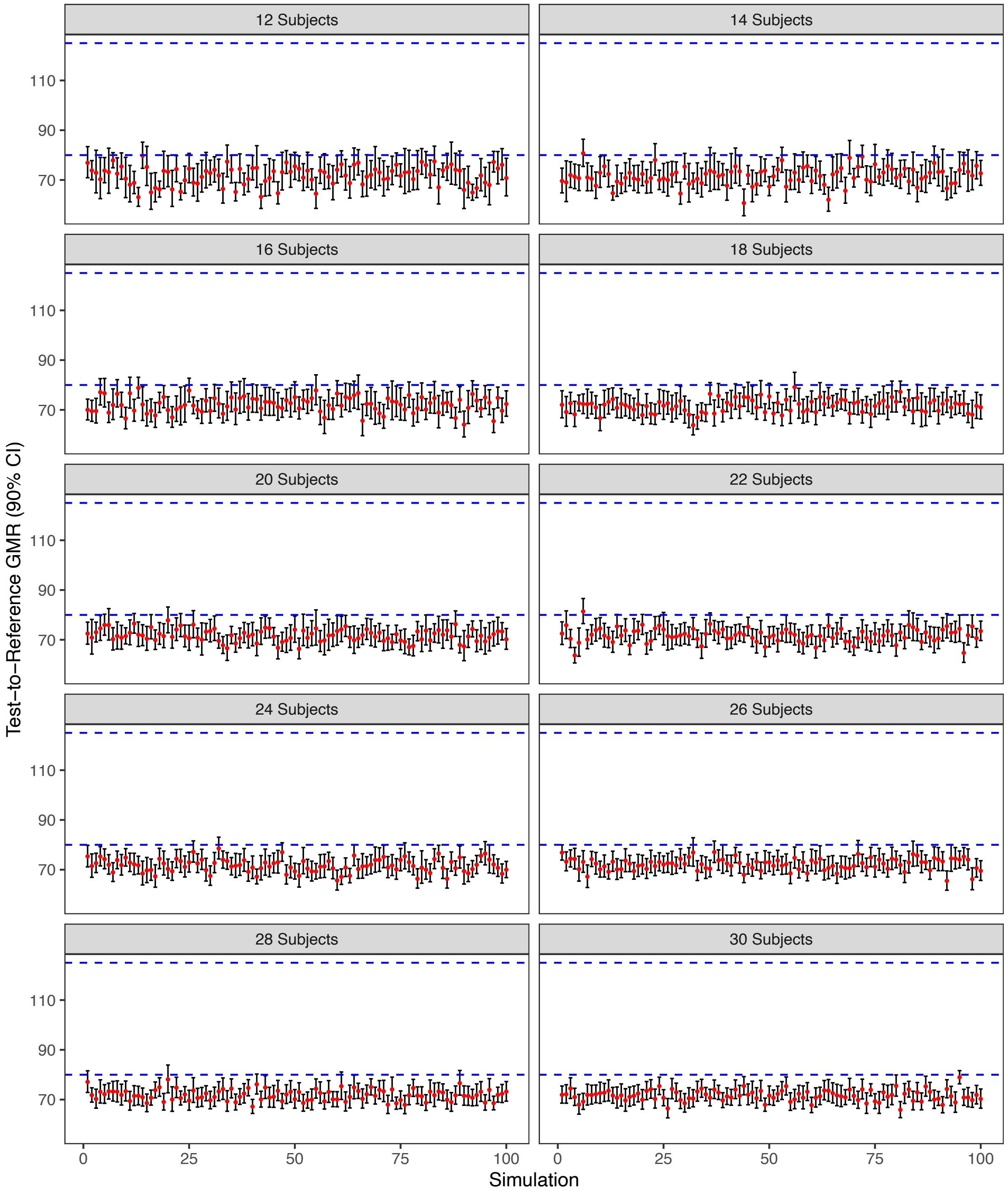

Figure S.84. Average Bioequivalence Analysis Results (Test-to-Reference GMR and Corresponding 90% CI) for C_{max} Derived from True Bioinequivalent Simulations with 30% IIV & 10% IOV in V

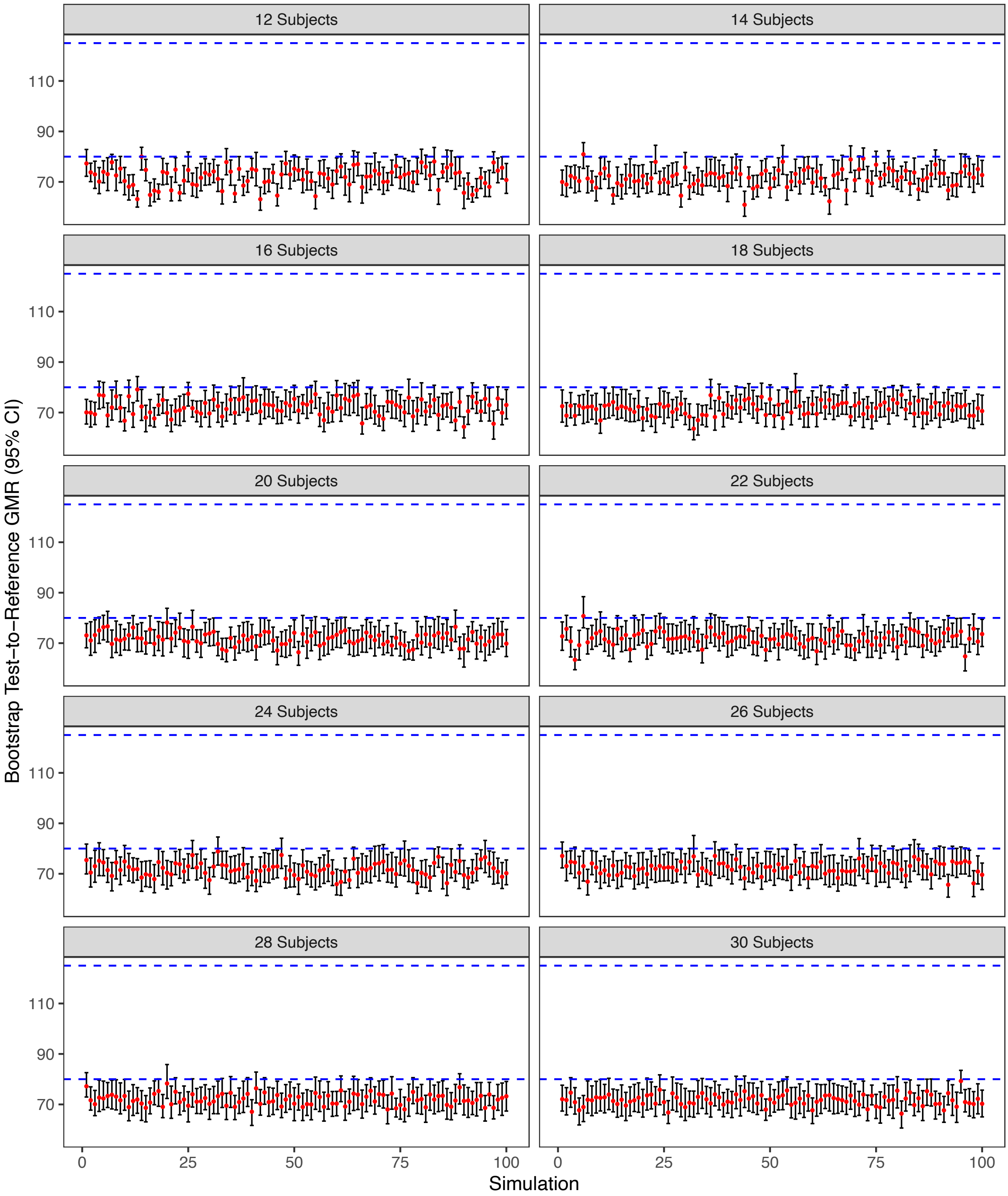

Figure S.85. Bootstrap Bioequivalence Analysis Results (Test-to-Reference GMR and Corresponding 95% CI) for C_{max} Derived from True Bioinequivalent Simulations with 30% IIV & 10% IOV in V

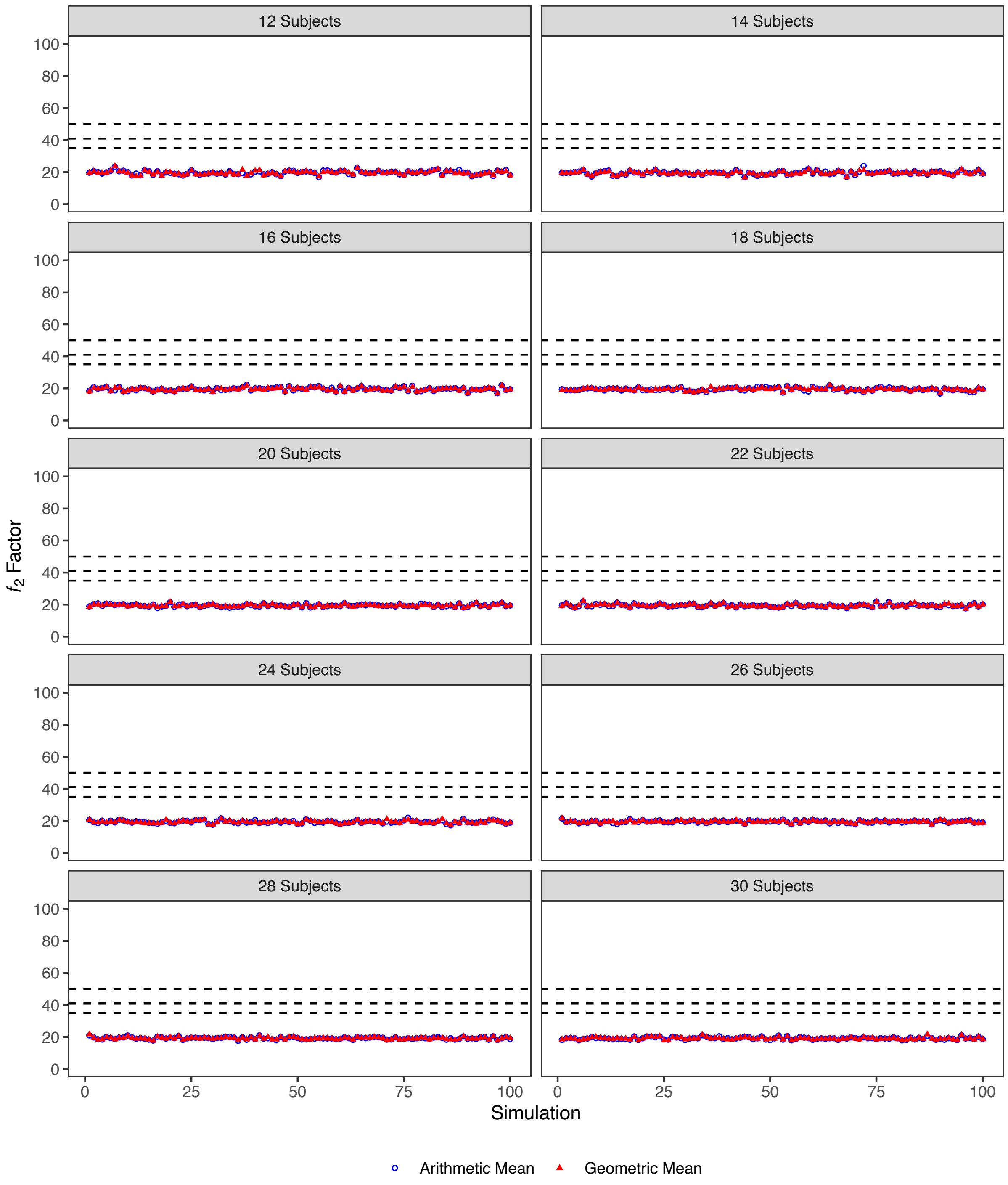

Figure S.86. Similarity f_2 Factor Results, Derived from Arithmetic and Geometric Mean Concentration-Time Profiles Until the Reference C_{max}, Derived from True Bioinequivalent Simulations with 30% IIV & 10% IOV in V

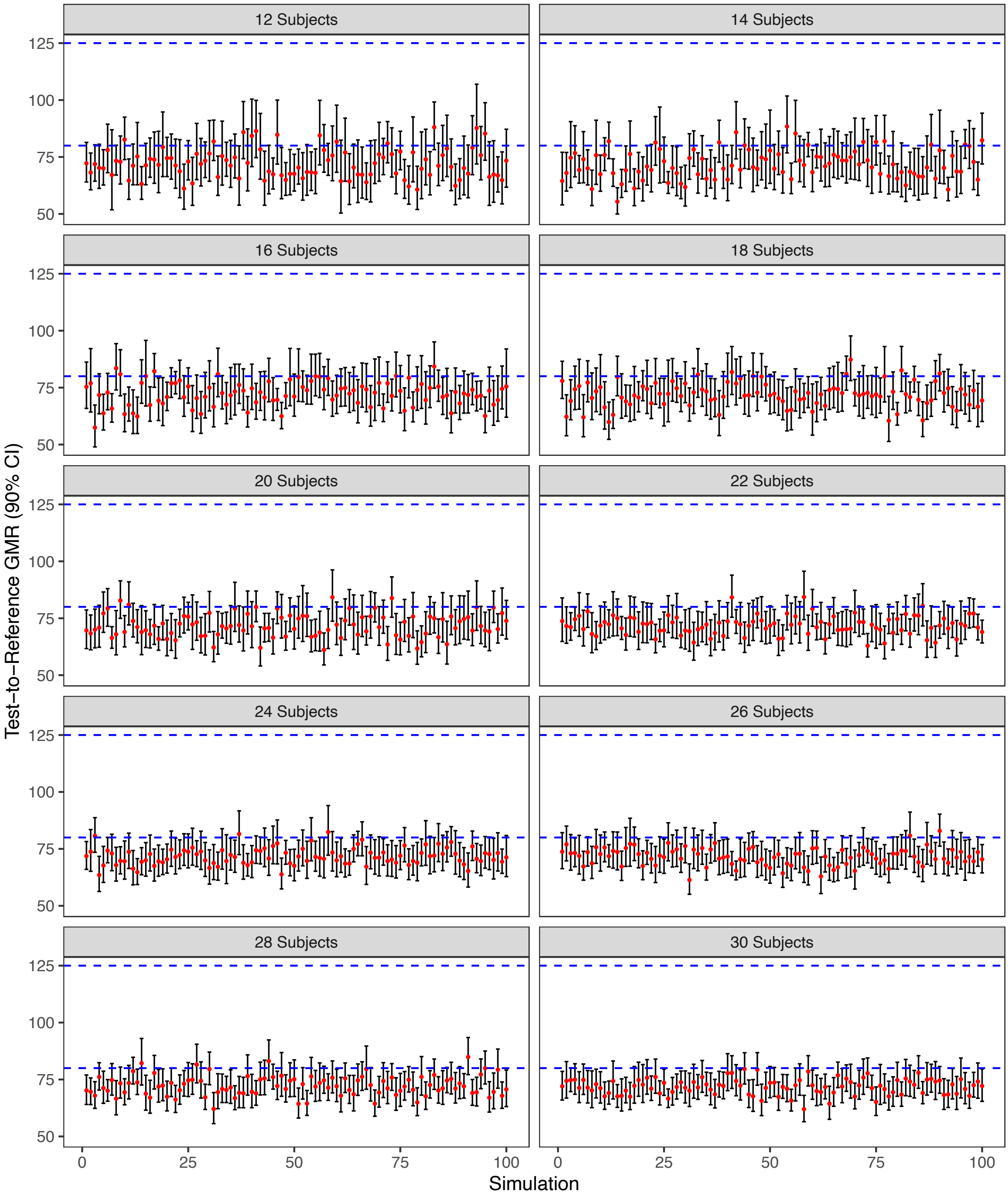

Figure S.87. Average Bioequivalence Analysis Results (Test-to-Reference GMR and Corresponding 90% CI) for C_{max} Derived from True Bioinequivalent Simulations with 30% IIV & 20% IOV in V

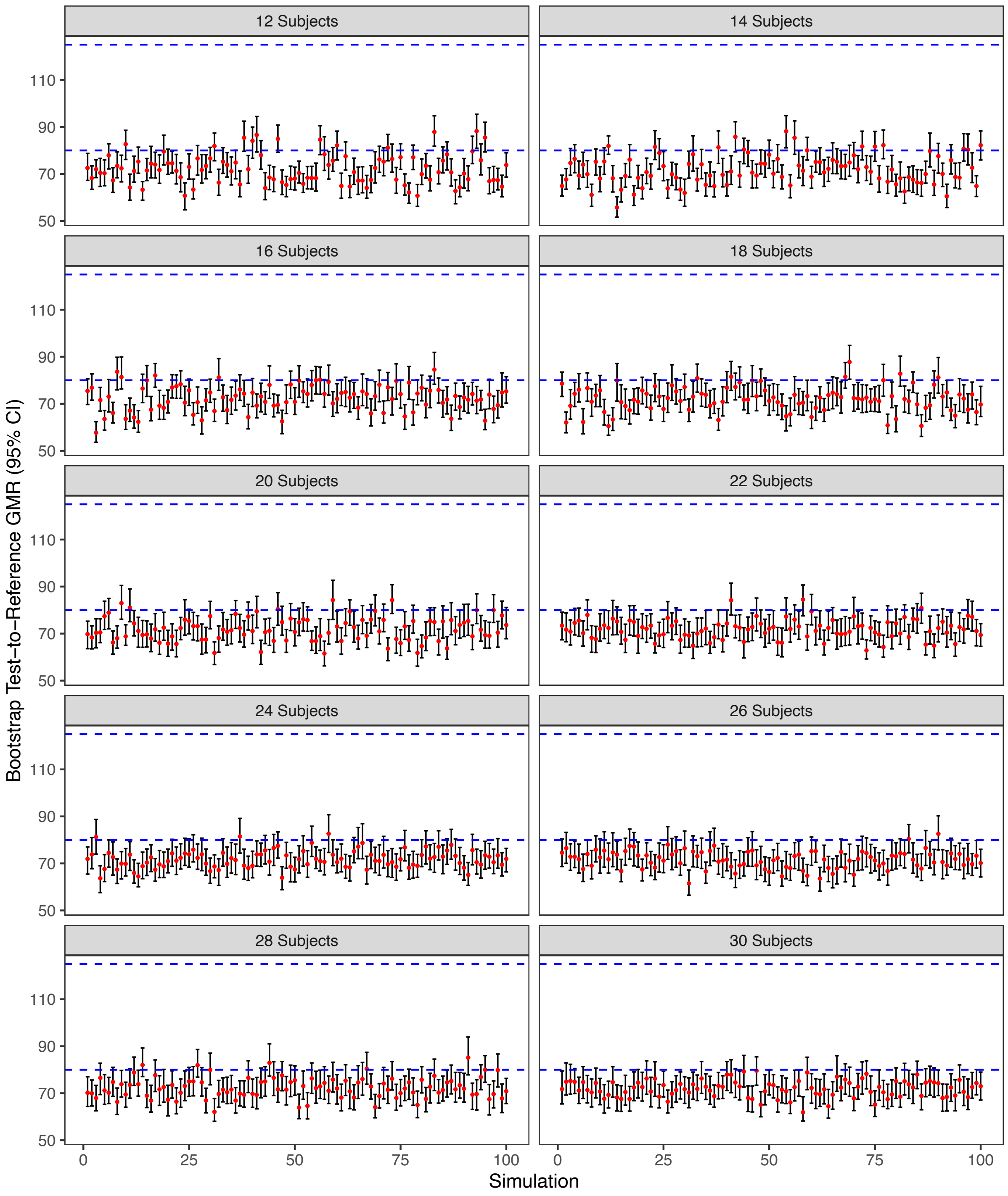

Figure S.88. Bootstrap Bioequivalence Analysis Results (Test-to-Reference GMR and Corresponding 95% CI) for C_{max} Derived from True Bioinequivalent Simulations with 30% IIV & 20% IOV in V

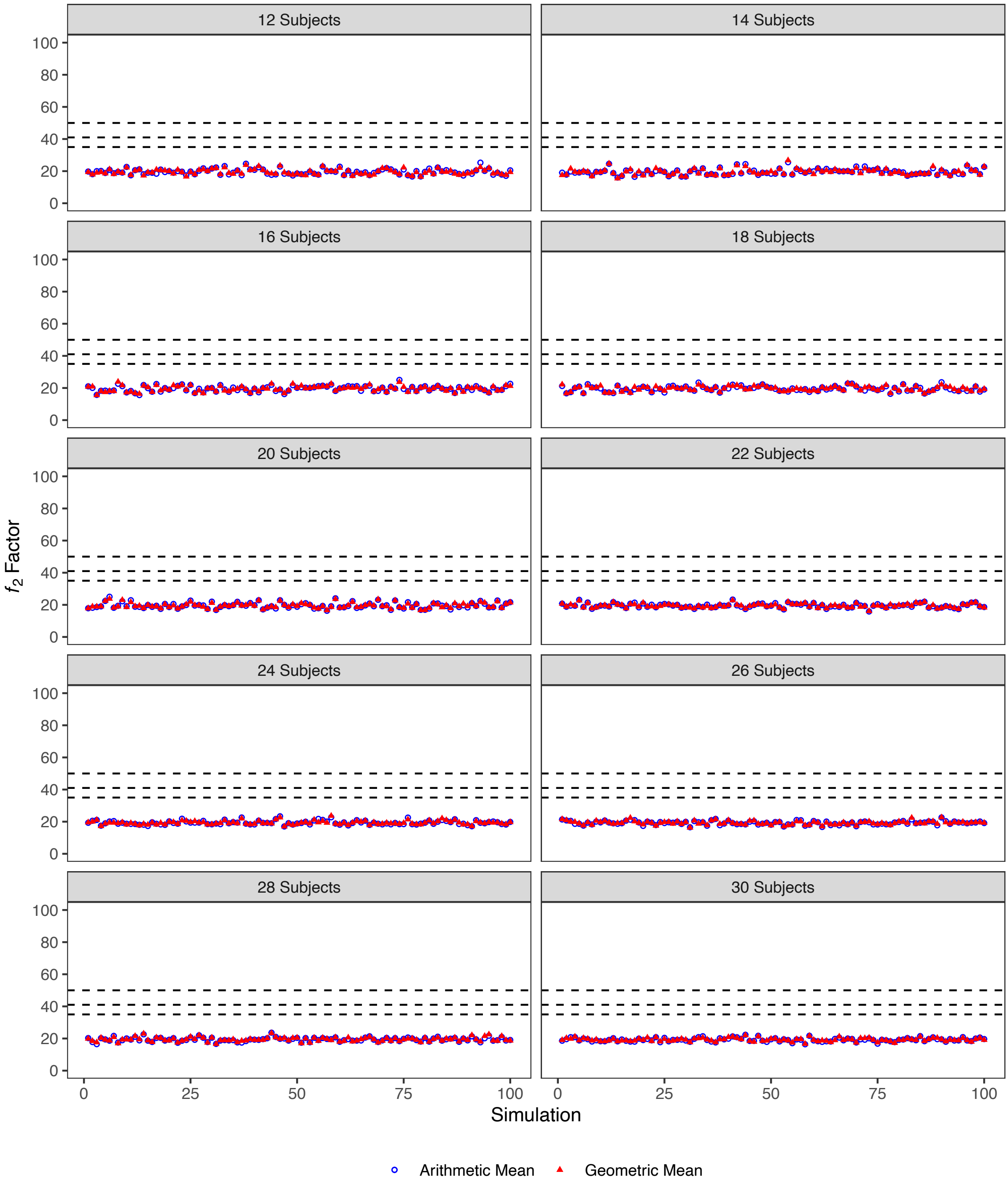

Figure S.89. Similarity f_2 Factor Results, Derived from Arithmetic and Geometric Mean Concentration-Time Profiles Until the Reference C_{max}, Derived from True Bioinequivalent Simulations with 30% IIV & 20% IOV in V

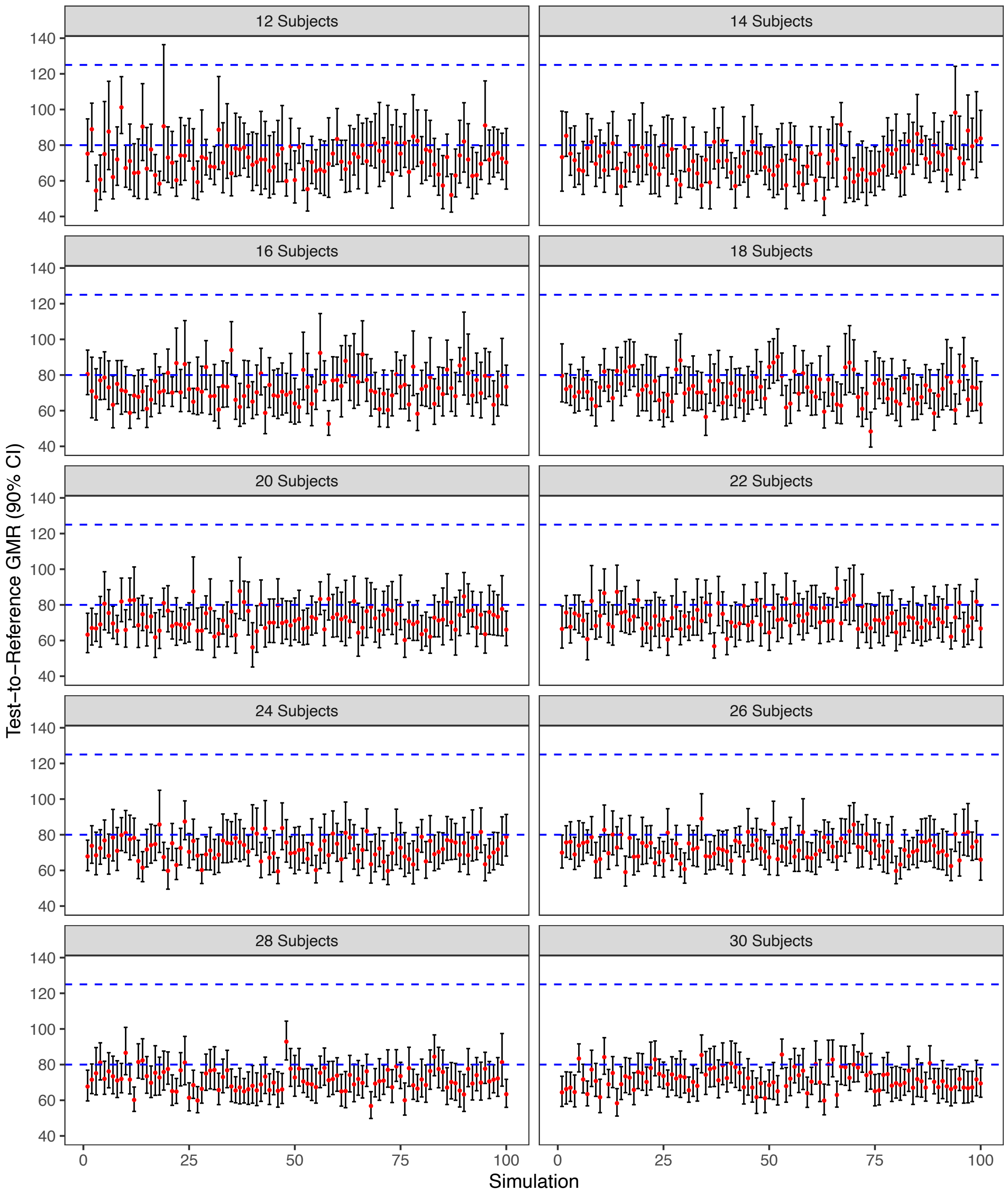

Figure S.90. Average Bioequivalence Analysis Results (Test-to-Reference GMR and Corresponding 90% CI) for C_{max} Derived from True Bioinequivalent Simulations with 30% IIV & 30% IOV in V

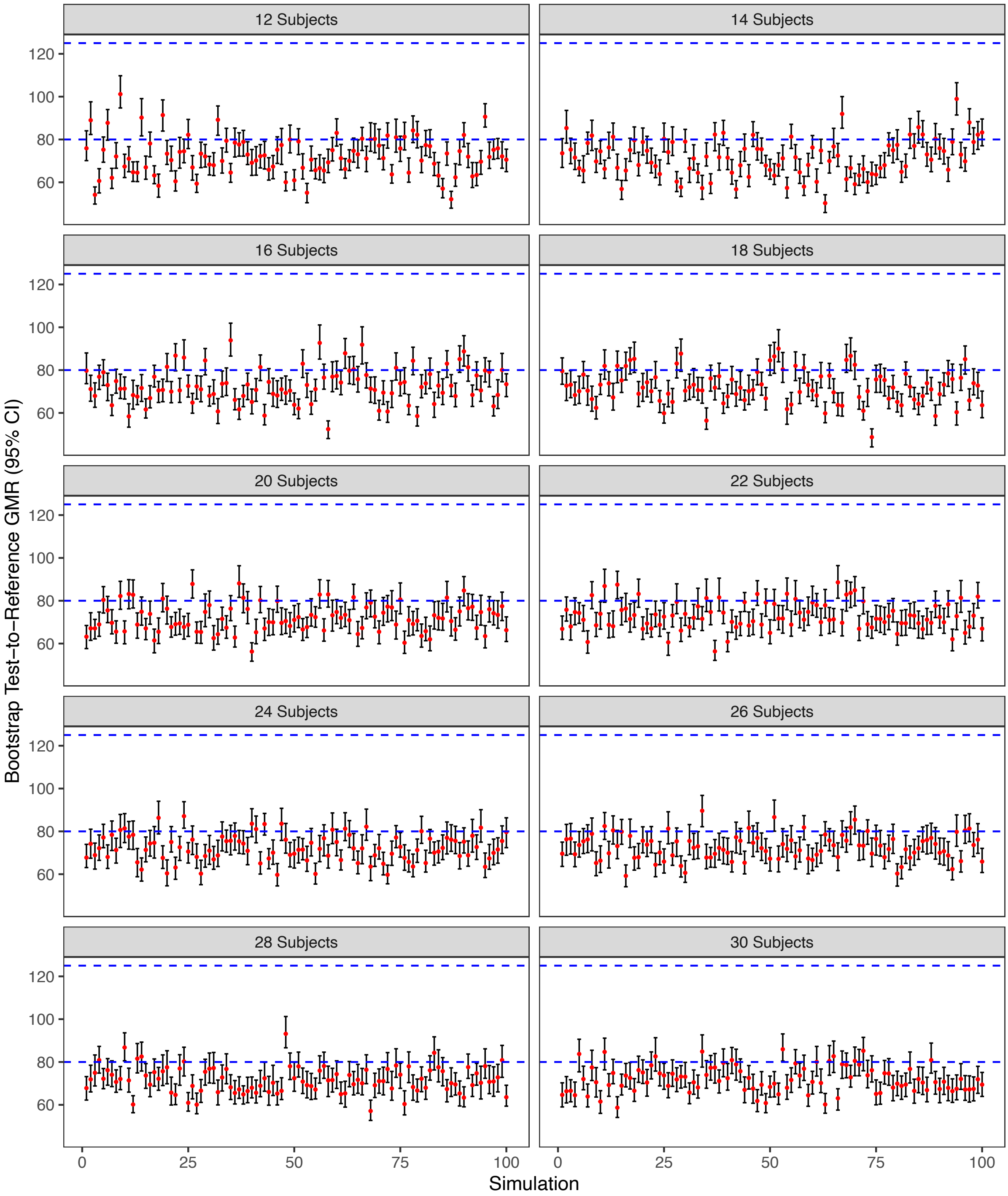

Figure S.91. Bootstrap Bioequivalence Analysis Results (Test-to-Reference GMR and Corresponding 95% CI) for C_{max} Derived from True Bioinequivalent Simulations with 30% IIV & 30% IOV in V

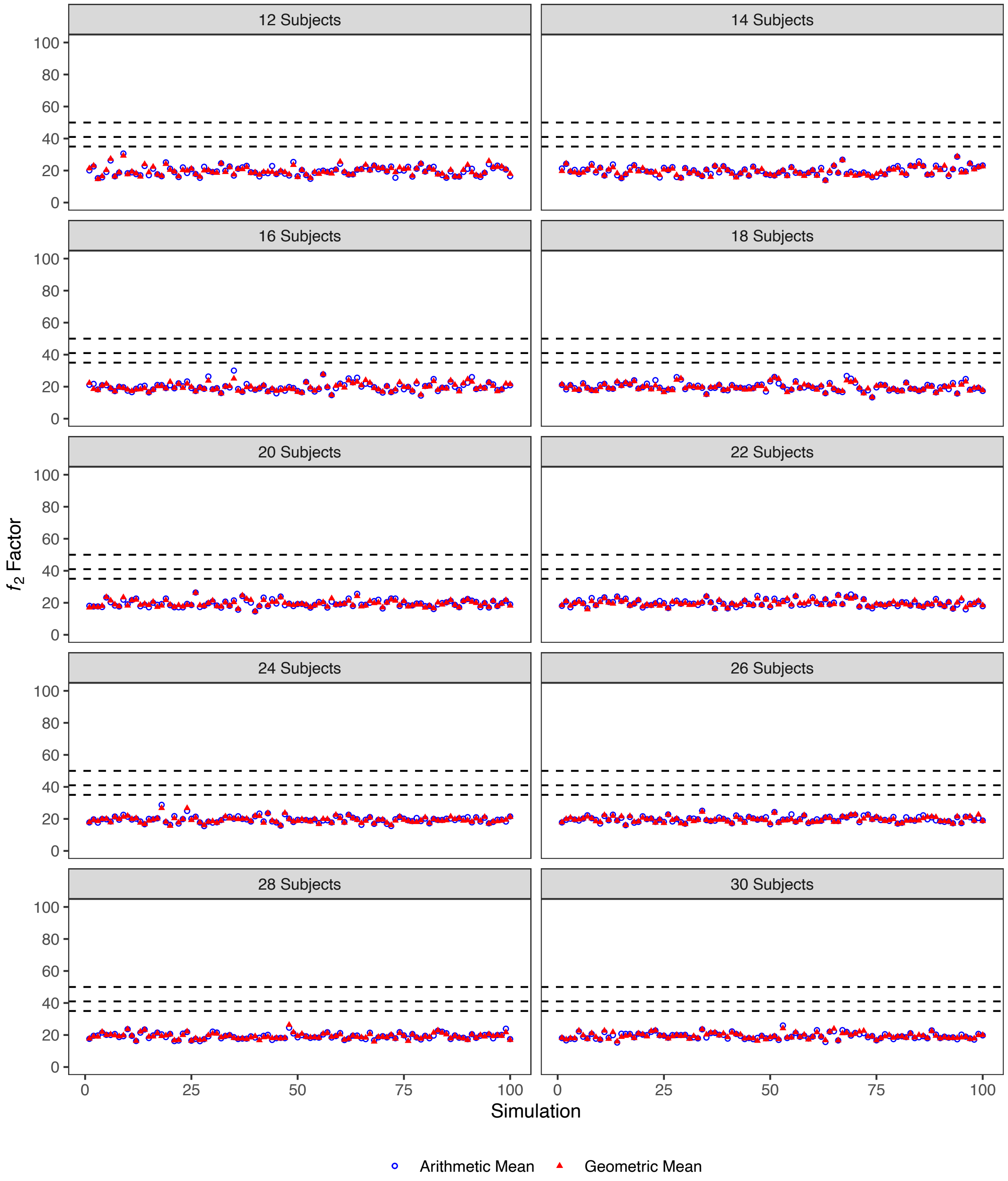

Figure S.92. Similarity f_2 Factor Results, Derived from Arithmetic and Geometric Mean Concentration-Time Profiles Until the Reference C_{max}, Derived from True Bioinequivalent Simulations with 30% IIV & 30% IOV in V

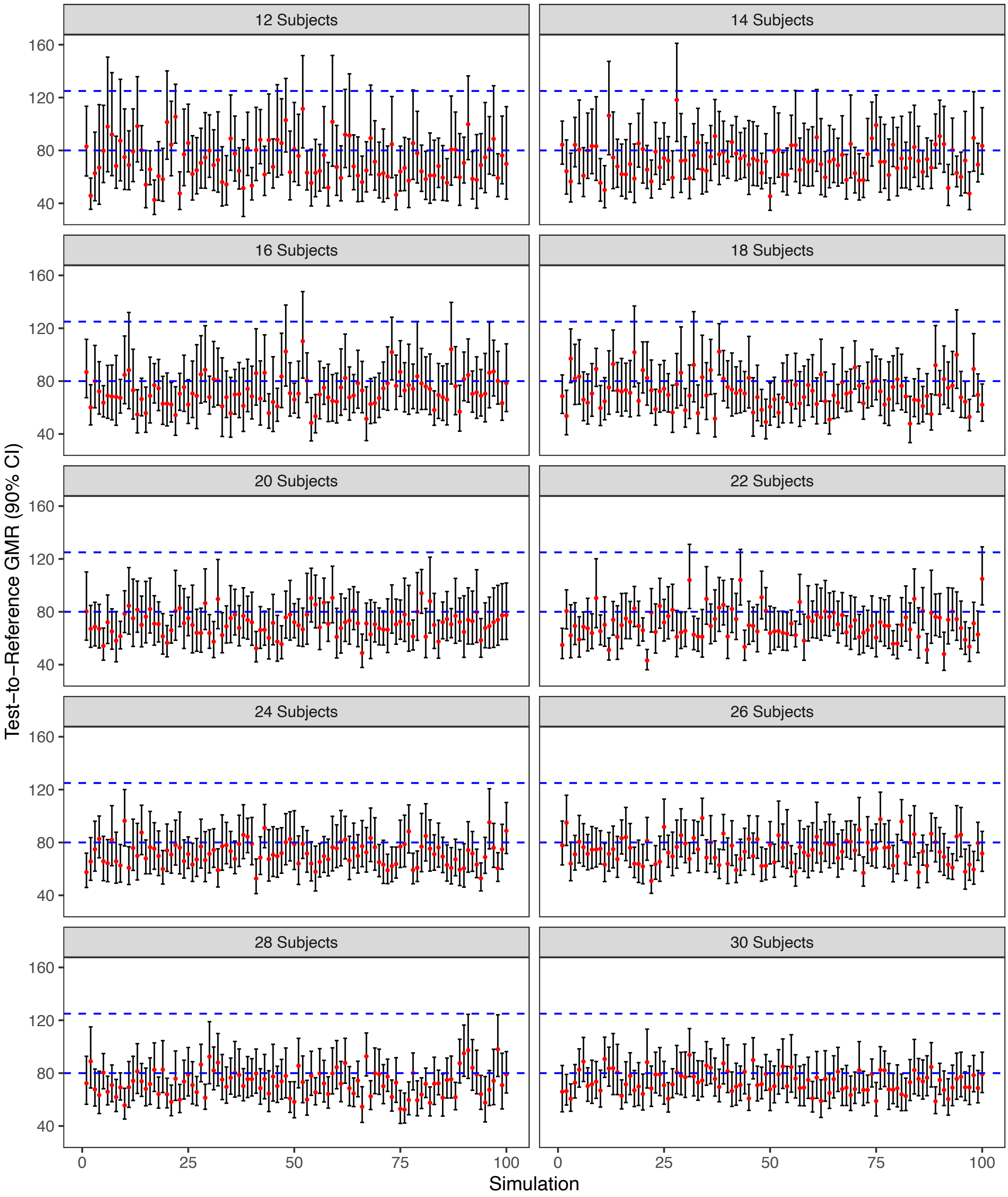

Figure S.93. Average Bioequivalence Analysis Results (Test-to-Reference GMR and Corresponding 90% CI) for C_{max} Derived from True Bioinequivalent Simulations with 0% IIV & 45% IOV in V

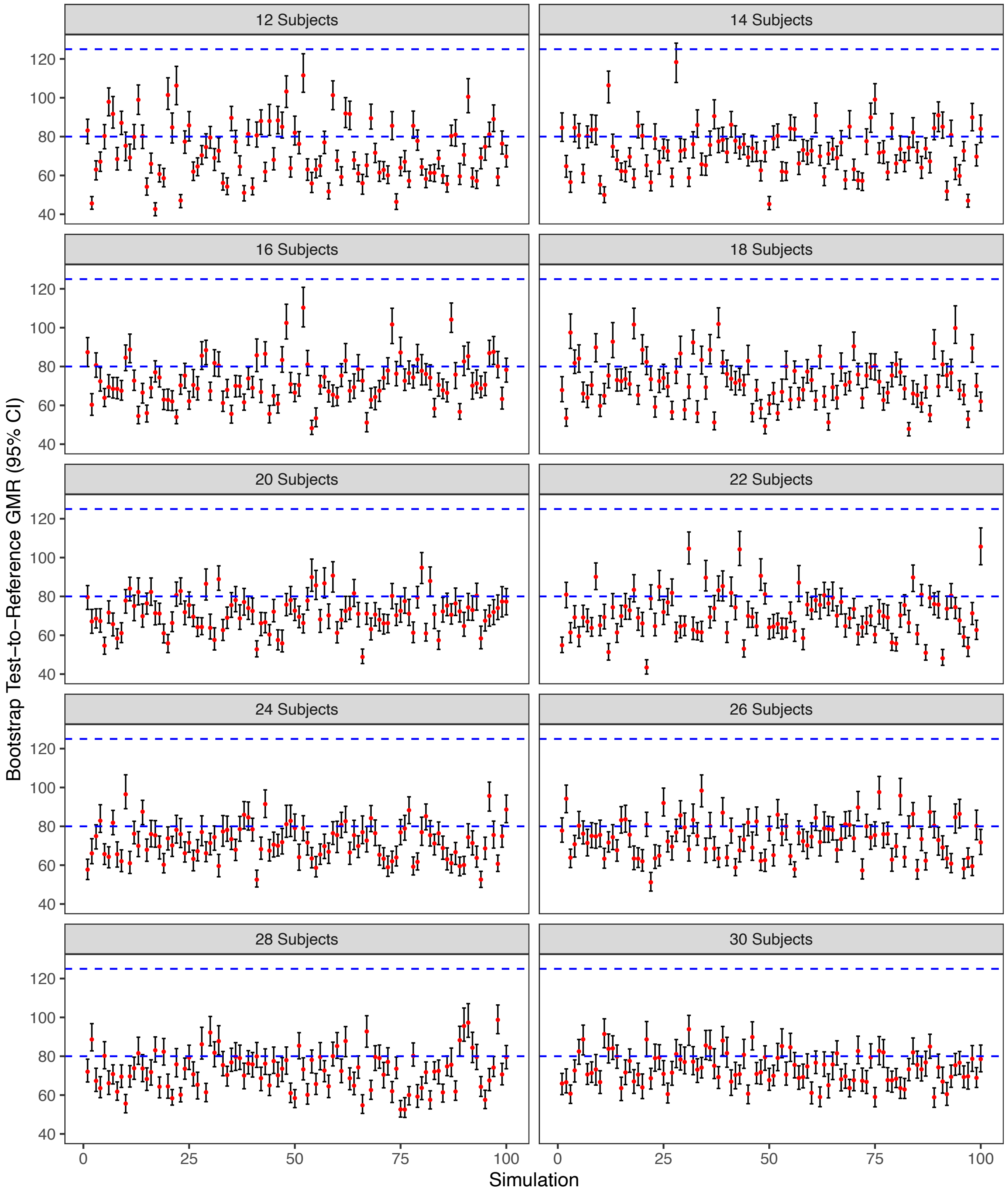

Figure S.94. Bootstrap Bioequivalence Analysis Results (Test-to-Reference GMR and Corresponding 95% CI) for C_{max} Derived from True Bioinequivalent Simulations with 0% IIV & 45% IOV in V

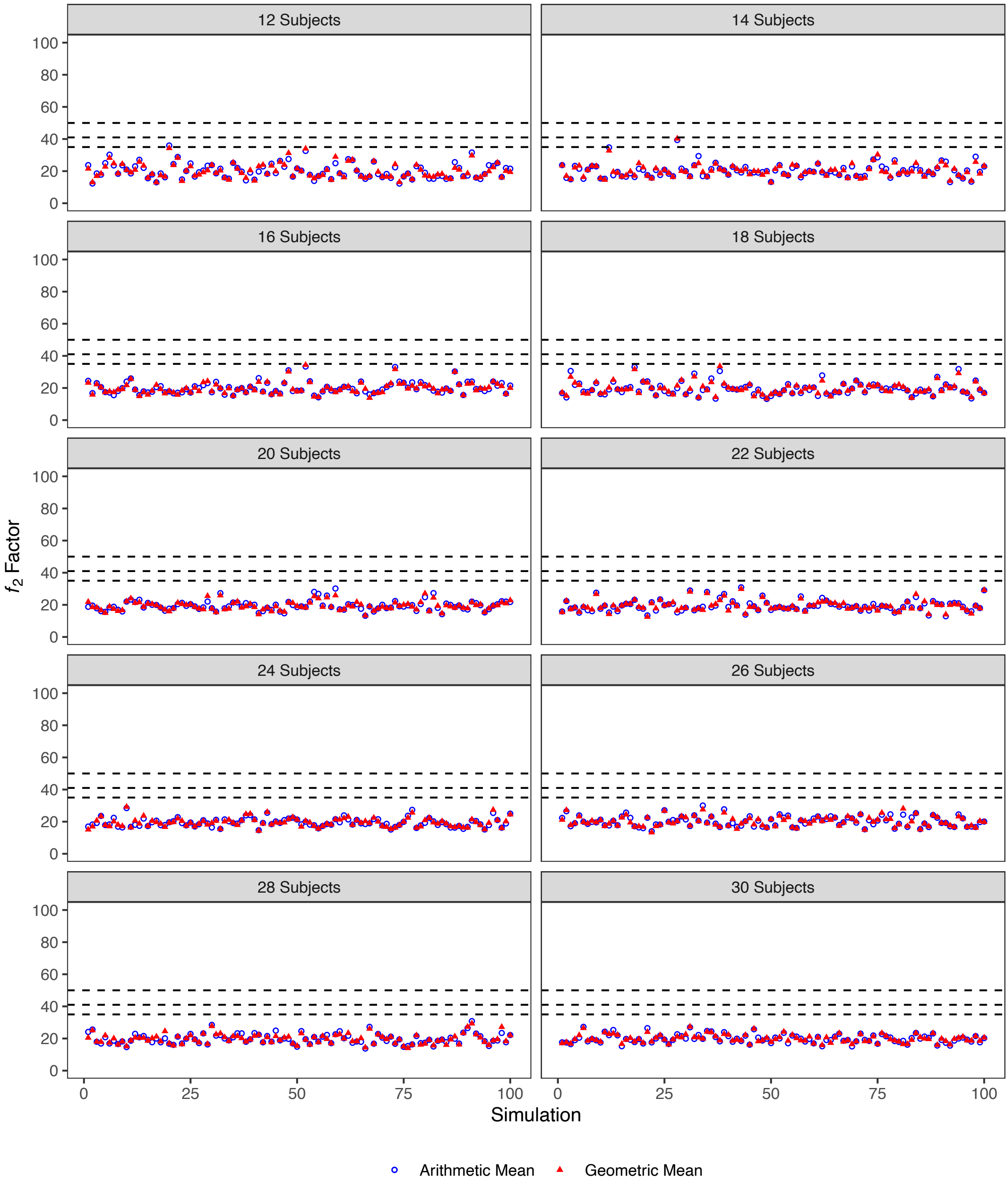

Figure S.95. Similarity f_2 Factor Results, Derived from Arithmetic and Geometric Mean Concentration-Time Profiles Until the Reference C_{max}, Derived from True Bioinequivalent Simulations with 0% IIV & 45% IOV in V

Appendix SA.4. Simulated Studies with Variability in k_e

Appendix SA.4.1. Simulated Pharmacokinetic Parameters

Table S.20. Descriptive Statistics of Simulated k_e for True Bioequivalent Studies

	30% IIV 0% IOV		30% IIV 10% IOV		30% IIV 20% IOV		30% IIV 30% IOV		0% IIV 45% IOV	
	Reference	Test	Reference	Test	Reference	Test	Reference	Test	Reference	Test
n	21000	21000	21000	21000	21000	21000	21000	21000	21000	21000
G_{Mean}	0.151	0.151	0.150	0.150	0.150	0.150	0.150	0.150	0.150	0.150
GSD	1.350	1.350	1.371	1.371	1.436	1.435	1.529	1.533	1.569	1.567
GCV%	30.7	30.7	32.4	32.4	37.4	37.3	44.4	44.8	47.4	47.3
G_{mean} 95% CI	0.150 – 0.151	0.150 – 0.151	0.149 – 0.151	0.149 – 0.151	0.149 – 0.150	0.149 – 0.151	0.149 – 0.151	0.149 – 0.151	0.149 – 0.151	0.149 – 0.151
A_{Mean}	0.157	0.157	0.158	0.158	0.160	0.160	0.164	0.164	0.166	0.166
SD	0.048	0.048	0.051	0.051	0.060	0.060	0.074	0.073	0.078	0.079
SE	0.000	0.000	0.000	0.000	0.000	0.000	0.001	0.001	0.001	0.001
CV%	30.7	30.7	32.6	32.4	37.4	37.4	44.9	44.6	47.3	47.5
Variance	0.002	0.002	0.003	0.003	0.004	0.004	0.005	0.005	0.006	0.006
A_{mean} 95% CI	0.157 – 0.158	0.157 – 0.158	0.157 – 0.158	0.157 – 0.158	0.159 – 0.161	0.159 – 0.161	0.163 – 0.165	0.163 – 0.165	0.165 – 0.167	0.165 – 0.167
Minimum	0.049	0.049	0.044	0.040	0.030	0.039	0.032	0.028	0.028	0.027
Q1	0.123	0.123	0.121	0.121	0.117	0.117	0.113	0.112	0.111	0.111
Median	0.150	0.150	0.150	0.150	0.150	0.150	0.150	0.150	0.150	0.150
Q3	0.185	0.185	0.186	0.186	0.191	0.191	0.199	0.200	0.203	0.203
Maximum	0.431	0.431	0.539	0.535	0.596	0.602	1.052	0.787	0.983	0.916

n: Number of observations, G_{mean}: Geometric mean, GSD: Geometric standard deviation, GCV%: Geometric coefficient of variation, G_{mean} 90% CI: 90% confidence interval of the geometric mean, A_{mean}: Arithmetic mean, SD: Standard deviation, SD: Standard error, CV%: Coefficient of variation, A_{mean} 90% CI: 90% confidence interval of the arithmetic mean, Q1: 1st (25%) quartile, Q3: 3rd (75%) quartile

Table S.21. Descriptive Statistics of Simulated k_e for True Bioinequivalent Studies

	30% IIV 0% IOV		30% IIV 10% IOV		30% IIV 20% IOV		30% IIV 30% IOV		0% IIV 45% IOV	
	Reference	Test	Reference	Test	Reference	Test	Reference	Test	Reference	Test
n	21000	21000	21000	21000	21000	21000	21000	21000	21000	21000
G_{Mean}	0.150	0.150	0.150	0.150	0.150	0.150	0.150	0.151	0.149	0.150
GSD	1.351	1.351	1.371	1.369	1.438	1.439	1.528	1.526	1.569	1.560
GCV%	30.8	30.8	32.3	32.2	37.5	37.6	44.4	44.2	47.4	46.8
G_{mean} 95% CI	0.150 – 0.151	0.150 – 0.151	0.149 – 0.151	0.150 – 0.151	0.149 – 0.151	0.149 – 0.151	0.149 – 0.151	0.150 – 0.152	0.148 – 0.150	0.149 – 0.151
A_{Mean}	0.157	0.157	0.158	0.158	0.160	0.160	0.164	0.165	0.165	0.165
SD	0.048	0.048	0.051	0.051	0.060	0.060	0.073	0.072	0.078	0.077
SE	0.000	0.000	0.000	0.000	0.000	0.000	0.001	0.000	0.001	0.001
CV%	30.7	30.7	32.2	32.2	37.4	37.6	44.5	43.9	47.5	46.5
Variance	0.002	0.002	0.003	0.003	0.004	0.004	0.005	0.005	0.006	0.006
A_{mean} 95% CI	0.156 – 0.158	0.156 – 0.158	0.157 – 0.158	0.157 – 0.158	0.159 – 0.161	0.160 – 0.161	0.163 – 0.165	0.164 – 0.166	0.164 – 0.166	0.164 – 0.166
Minimum	0.048	0.048	0.046	0.045	0.036	0.031	0.032	0.030	0.030	0.014
Q1	0.123	0.123	0.121	0.122	0.117	0.118	0.113	0.113	0.110	0.111
Median	0.150	0.150	0.150	0.150	0.150	0.150	0.150	0.151	0.149	0.150
Q3	0.184	0.184	0.185	0.185	0.192	0.192	0.200	0.201	0.203	0.202
Maximum	0.448	0.448	0.527	0.495	0.548	0.575	0.884	0.793	0.878	1.044

n: Number of observations, G_{mean}: Geometric mean, GSD: Geometric standard deviation, GCV%: Geometric coefficient of variation, G_{mean} 90% CI: 90% confidence interval of the geometric mean, A_{mean}: Arithmetic mean, SD: Standard deviation, SD: Standard error, CV%: Coefficient of variation, A_{mean} 90% CI: 90% confidence interval of the arithmetic mean, Q1: 1st (25%) quartile, Q3: 3rd (75%) quartile

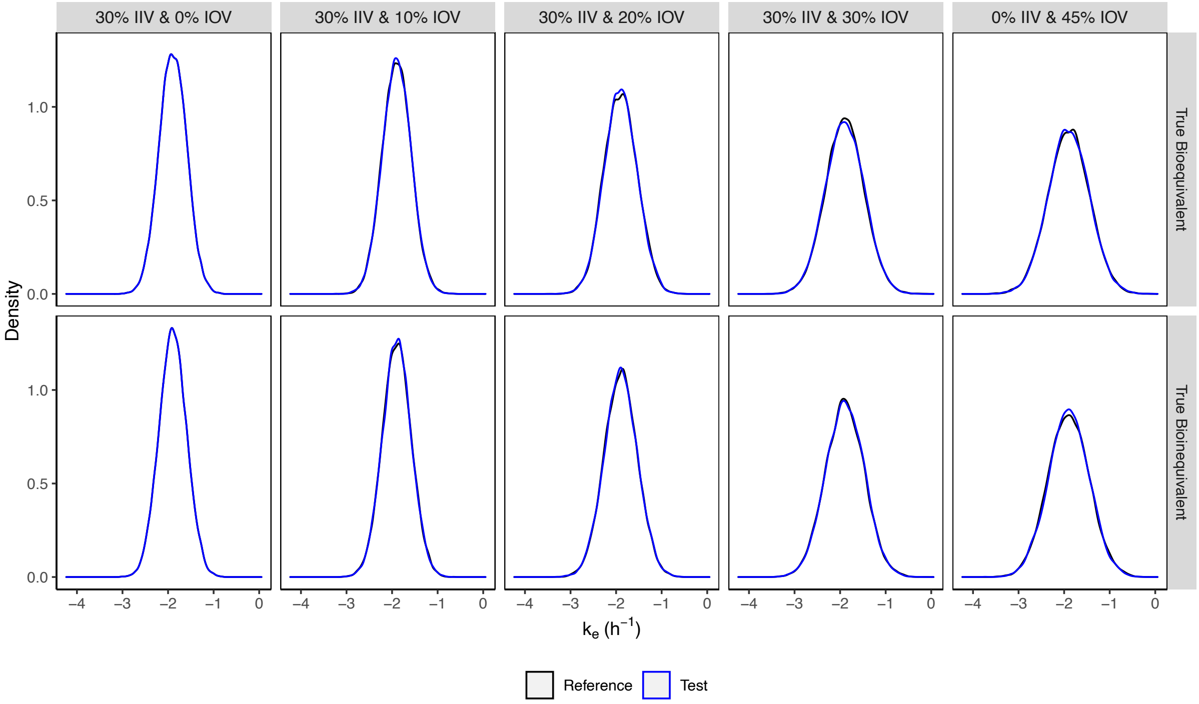

Figure S.96. Distribution of Simulated *Ln*-Transformed k_e

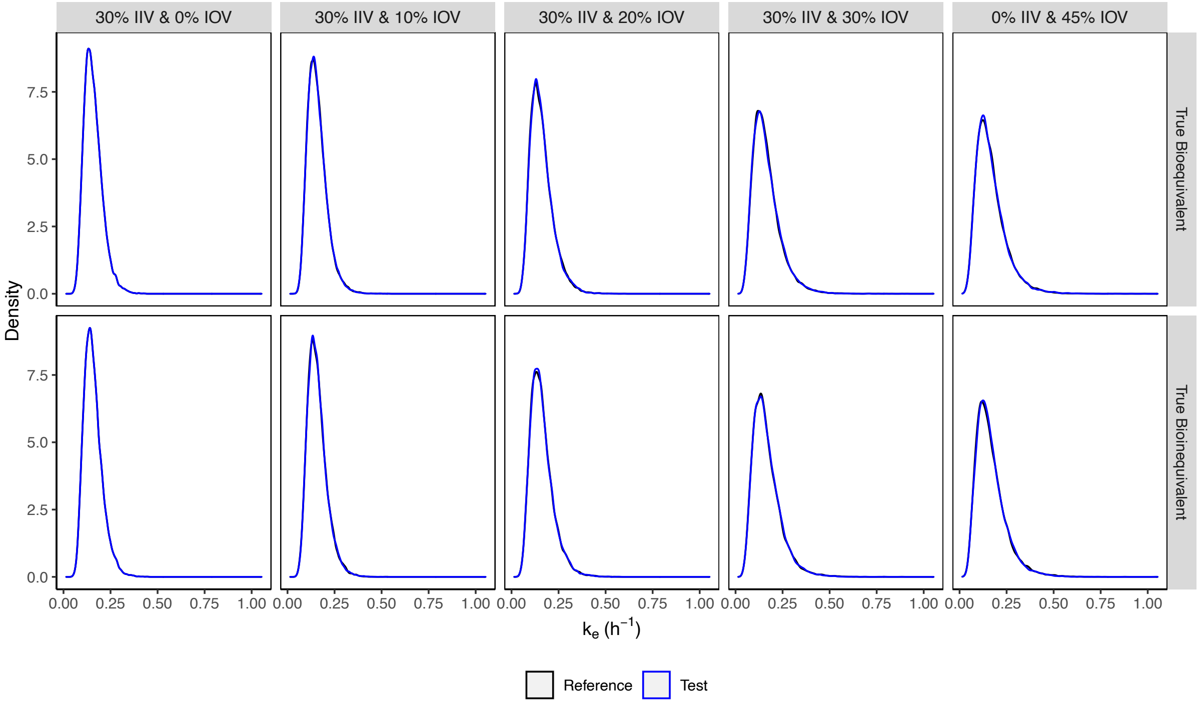

Figure S.97. Distribution of Simulated Untransformed k_e

Appendix SA.4.2. Simulated Concentrations

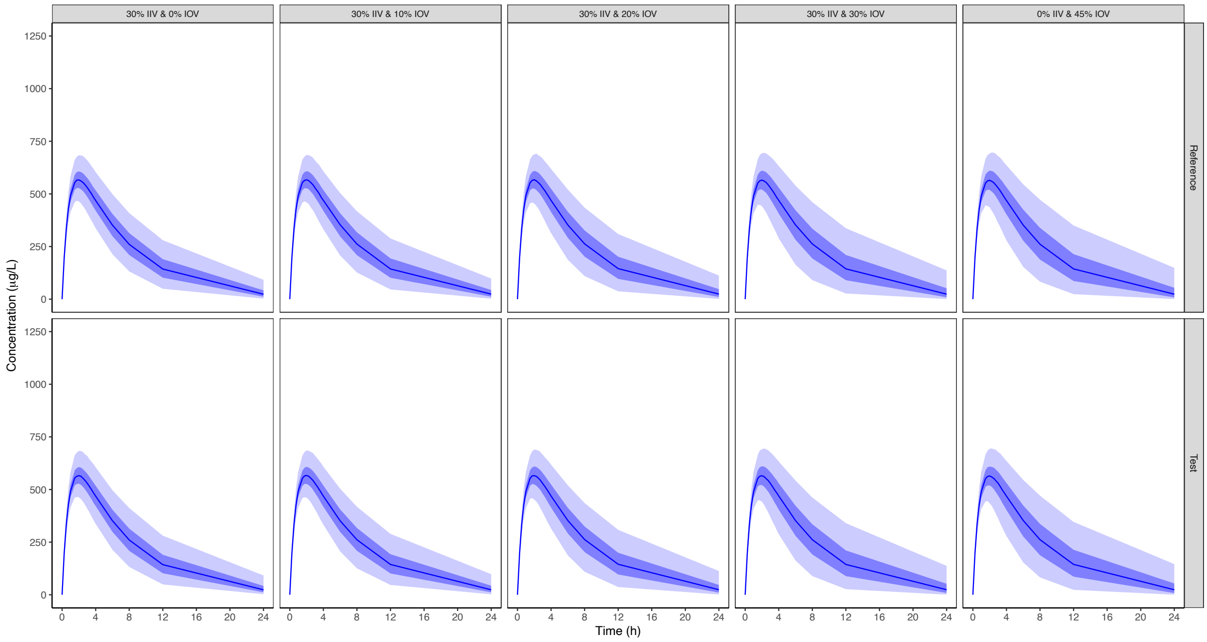

Figure S.98. 95% Confidence Interval of Simulated Concentration-Time Data, for True Bioequivalent Studies, with Variability in k_e – Linear Scale

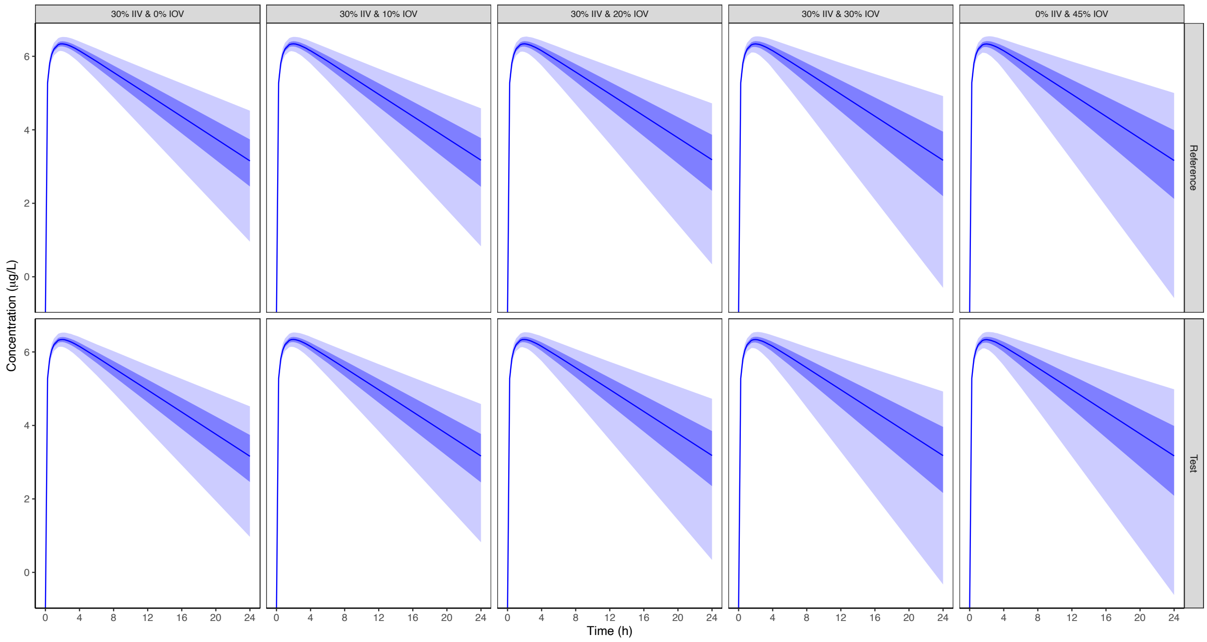

Figure S.99. 95% Confidence Interval of Simulated Concentration-Time Data, for True Bioequivalent Studies, with Variability in k_e – Semi-Logarithmic Scale

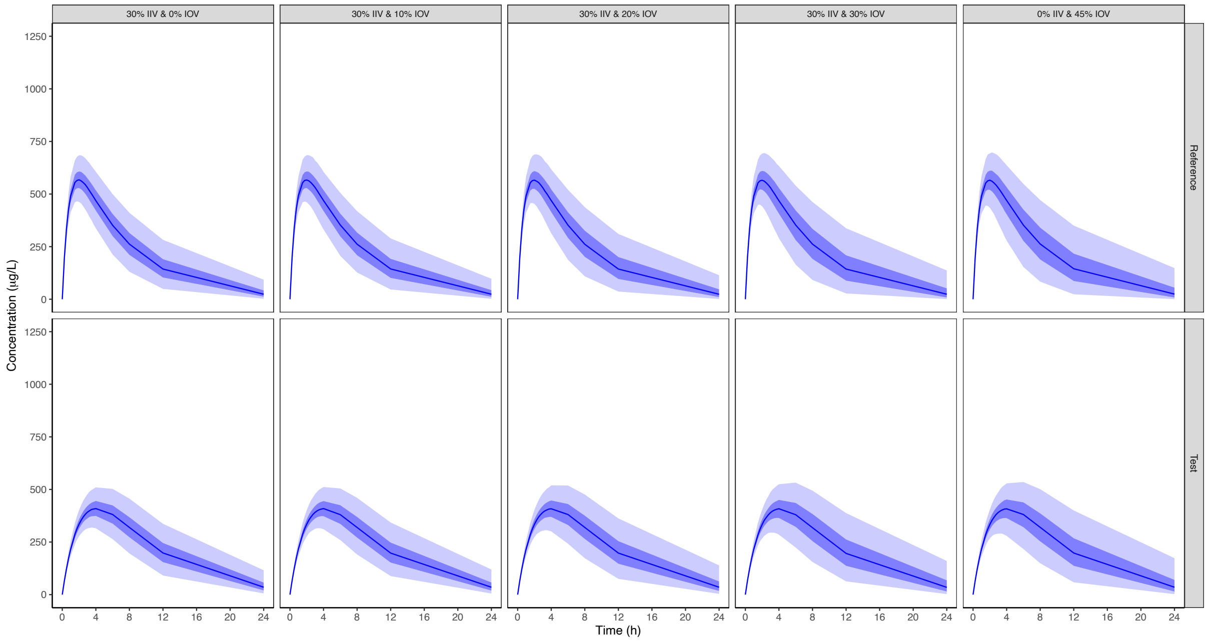

Figure S.100. 95% Confidence Interval of Simulated Concentration-Time Data, for True Bioinequivalent Studies, with Variability in k_e – Linear Scale

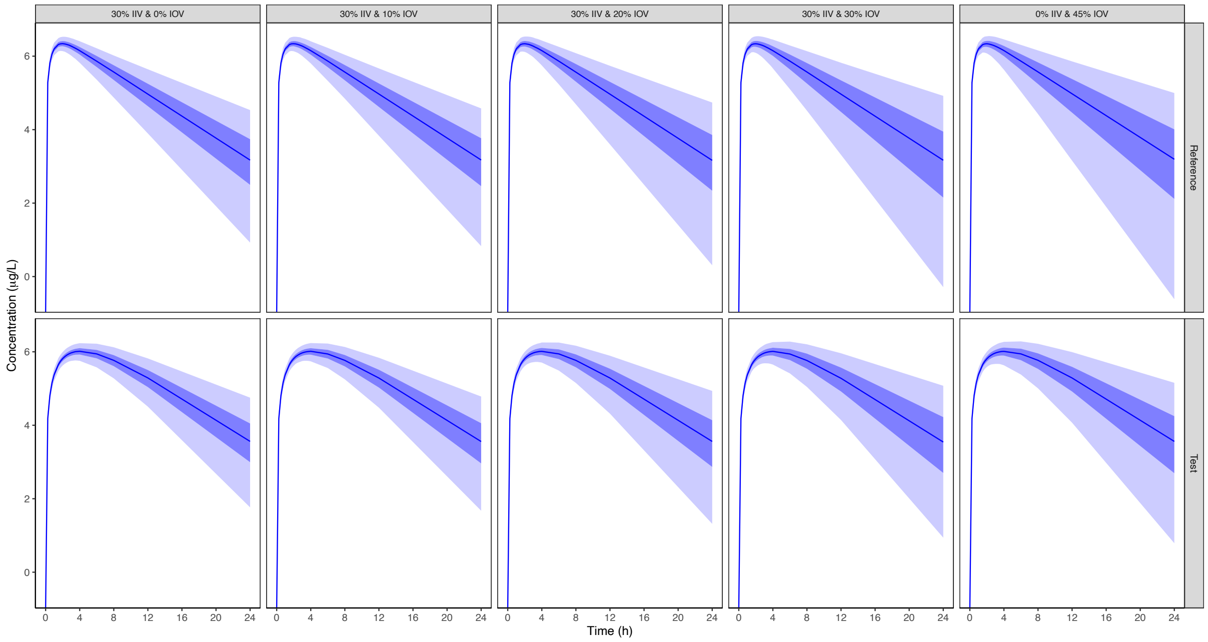

Figure S.101. 95% Confidence Interval of Simulated Concentration-Time Data, for True Bioinequivalent Studies, with Variability in k_e – Semi-Logarithmic Scale

Appendix SA.4.3. Simulated Non-Compartmental Analysis Parameters

Table S.22. Descriptive Statistics of C_{max} (µg/L) Derived from Simulated True Bioequivalent Studies, with Variability in k_e

	30% IIV 0% IOV		30% IIV 10% IOV		30% IIV 20% IOV		30% IIV 30% IOV		0% IIV 45% IOV	
	Reference	Test	Reference	Test	Reference	Test	Reference	Test	Reference	Test
n	21000	21000	21000	21000	21000	21000	21000	21000	21000	21000
G_{Mean}	638.17	637.87	637.81	637.71	637.27	637.41	635.08	635.12	633.31	633.07
GSD	1.10	1.10	1.10	1.10	1.11	1.11	1.13	1.13	1.13	1.13
GCV%	9.4	9.4	9.8	9.7	10.5	10.5	11.9	11.9	12.4	12.3
G_{mean} 95% CI	637.36 – 638.98	637.06 – 638.68	636.96 – 638.65	636.87 – 638.55	636.37 – 638.17	636.51 – 638.31	634.07 – 636.09	634.10 – 636.14	632.25 – 634.37	632.02 – 634.12
A_{Mean}	640.99	640.68	641.06	640.69	640.71	640.86	639.44	639.51	638.08	637.77
SD	60.48	60.02	86.2	61.62	66.11	66.23	73.79	74.18	77.22	76.45
SE	0.42	0.41	0.59	0.43	0.46	0.46	0.51	0.51	0.53	0.53
CV%	9.4	9.4	13.4	9.6	10.3	10.3	11.5	11.6	12.1	12.0
Variance	3657.64	3602.82	7430.36	3796.94	4370.03	4387.03	5444.77	5502.98	5962.22	5844.38
A_{mean} 95% CI	640.17 – 641.80	639.87 – 641.49	639.89 – 642.22	639.85 – 641.52	639.82 – 641.61	639.96 – 641.75	638.44 – 640.44	638.51 – 640.51	637.03 – 639.12	636.74 – 638.81
Minimum	418.03	398.37	419.82	350.28	408.36	372.68	301.39	362.15	285.46	297.57
Q1	600.61	600.13	600.45	599.18	596.52	597.59	590.8	589.86	586.99	587.5
Median	639.6	639.19	640.55	639.35	640.49	640.98	640.1	641.2	639.93	638.76
Q3	679.88	679.64	680	680.46	684.44	683.96	688.98	689.2	690.3	689.55
Maximum	2074.81	962.14	9483.74	921.95	944.66	1704.06	959.37	986.84	1843.76	948.25

n: Number of observations, G_{mean}: Geometric mean, GSD: Geometric standard deviation, GCV%: Geometric coefficient of variation, G_{mean} 95% CI: 95% confidence interval of the geometric mean, A_{mean}: Arithmetic mean, SD: Standard deviation, SD: Standard error, CV%: Coefficient of variation, A_{mean} 95% CI: 95% confidence interval of the arithmetic mean, Q1: 1st (25%) quartile, Q3: 3rd (75%) quartile

Table S.23. Descriptive Statistics of C_{max} (μg/L) Derived from Simulated True Bioinequivalent Studies, with Variability in k_e

	30% IIV 0% IOV		30% IIV 10% IOV		30% IIV 20% IOV		30% IIV 30% IOV		0% IIV 45% IOV	
	**Reference**	**Test**	**Reference**	**Test**	**Reference**	**Test**	**Reference**	**Test**	**Reference**	**Test**
n	21000	21000	21000	21000	21000	21000	21000	21000	21000	21000
G_{Mean}	638.27	456.69	638.58	456.04	637.23	454.97	634.84	452.9	634.59	452.7
GSD	1.1	1.12	1.1	1.13	1.11	1.14	1.13	1.16	1.13	1.17
GCV%	9.4	11.4	9.6	11.9	10.5	13.4	11.9	15.3	12.3	16.0
G_{mean} 95% CI	637.46 – 639.08	455.99 – 457.40	637.75 – 639.40	455.31 – 456.77	636.32 – 638.13	454.15 – 455.80	633.82 – 635.86	451.97 – 453.83	633.54 – 635.64	451.73 – 453.67
A_{Mean}	641.09	459.64	641.51	459.23	640.73	458.99	639.27	458.04	639.29	458.28
SD	60.73	51.95	61.83	54.1	66.8	60.32	76.4	67.72	76.49	70.43
SE	0.42	0.36	0.43	0.37	0.46	0.42	0.53	0.47	0.53	0.49
CV%	9.5	11.3	9.6	11.8	10.4	13.1	12.0	14.8	12.0	15.4
Variance	3687.8	2699.03	3822.52	2926.74	4461.79	3638.98	5837.14	4585.67	5851.47	4960.06
A_{mean} 95% CI	640.26 – 641.91	458.94 – 460.35	640.67 – 642.35	458.50 – 459.96	639.82 – 641.63	458.18 – 459.81	638.24 – 640.31	457.12 – 458.95	638.26 – 640.33	457.33 – 459.24
Minimum	324.62	241.18	406.49	269.12	392.67	239.44	337.24	174.46	346.63	157.66
Q1	601.41	424.36	599.89	423.17	596.21	419.27	590.17	412.69	589.01	411.06
Median	639.21	458.9	640.7	458.37	641.01	458.53	640.29	457.99	641.48	458.10
Q3	679.49	493.74	680.99	493.96	684.59	498.06	689.09	503.19	691.38	505.34
Maximum	2317.91	758.35	2189.38	1151.35	1754.11	1076.57	3476.08	863.24	939.68	756.12

n: Number of observations, G_{mean}: Geometric mean, GSD: Geometric standard deviation, GCV%: Geometric coefficient of variation, G_{mean} 95% CI: 95% confidence interval of the geometric mean, A_{mean}: Arithmetic mean, SD: Standard deviation, SD: Standard error, CV%: Coefficient of variation, A_{mean} 95% CI: 95% confidence interval of the arithmetic mean, Q1: 1st (25%) quartile, Q3: 3rd (75%) quartile

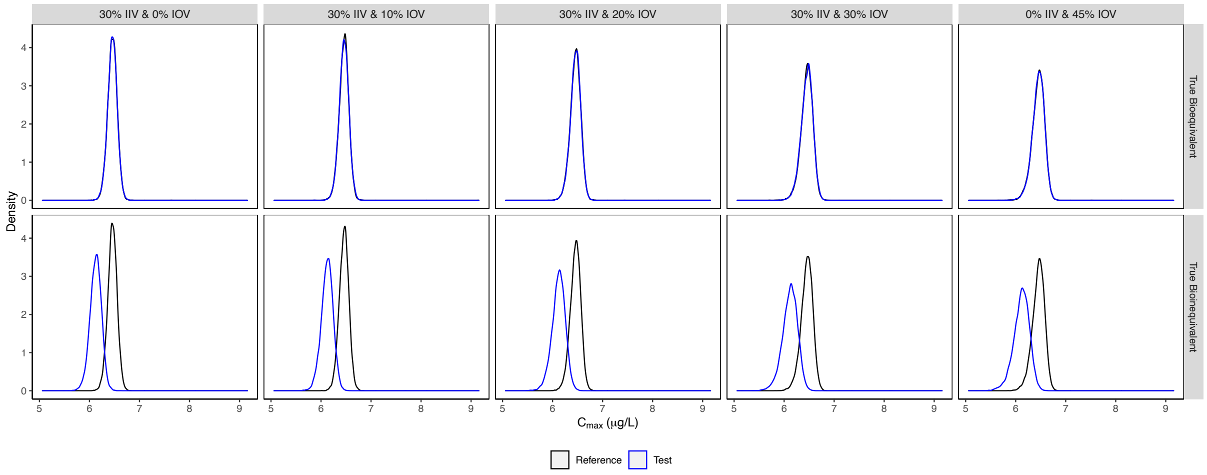

Figure S.102. Distribution of *Ln*-Transformed C_{max}, Derived from Simulations for Studies with Variability in k_e

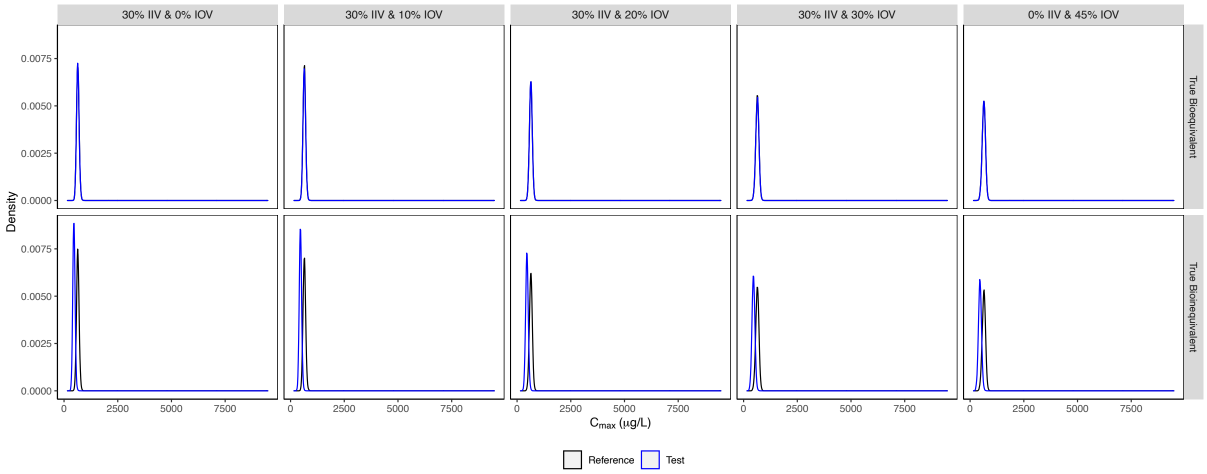

Figure S.103. Distribution of Untransformed C_{max}, Derived from Simulations for Studies with Variability in k_e

Table S.24. Descriptive Statistics of t_{max} (h) Derived from Simulated True Bioequivalent Studies, with Variability in k_e

	30% IIV 0% IOV		30% IIV 10% IOV		30% IIV 20% IOV		30% IIV 30% IOV		0% IIV 45% IOV	
	**Reference**	**Test**	**Reference**	**Test**	**Reference**	**Test**	**Reference**	**Test**	**Reference**	**Test**
n	21000	21000	21000	21000	21000	21000	21000	21000	21000	21000
Minimum	0.75	0.75	0.75	0.75	0.75	0.75	0.50	0.75	0.50	0.75
Q1	1.75	1.75	1.75	1.75	1.75	1.75	1.75	1.75	1.75	1.75
Median	2.25	2.25	2.00	2.25	2.00	2.00	2.00	2.00	2.00	2.00
Q3	2.50	2.50	2.50	2.50	2.50	2.50	2.50	2.50	2.50	2.50
Maximum	4.00	6.00	12.00	6.00	6.00	6.00	6.00	6.00	8.00	6.00

n: Number of observations, Q1: 1st (25%) quartile, Q3: 3rd (75%) quartile

Table S.25. Descriptive Statistics of t_{max} (h) Derived from Simulated True Bioinequivalent Studies, with Variability in k_e

	30% IIV 0% IOV		30% IIV 10% IOV		30% IIV 20% IOV		30% IIV 30% IOV		0% IIV 45% IOV	
	**Reference**	**Test**	**Reference**	**Test**	**Reference**	**Test**	**Reference**	**Test**	**Reference**	**Test**
n	21000	21000	21000	21000	21000	21000	21000	21000	21000	21000
Minimum	0.75	1.5	0.75	1.5	0.75	1.50	0.75	1.00	0.75	1.00
Q1	1.75	3.25	1.75	3.25	1.75	3.00	1.75	3.25	1.75	3.00
Median	2.25	3.50	2.25	3.50	2.00	3.5	2.00	3.50	2.00	3.50
Q3	2.50	4.00	2.50	4.00	2.50	4.00	2.50	4.00	2.50	4.00
Maximum	6.00	8.00	6.00	12.00	6.00	12.00	4.00	12.00	8.00	12.00

n: Number of observations, Q1: 1st (25%) quartile, Q3: 3rd (75%) quartile

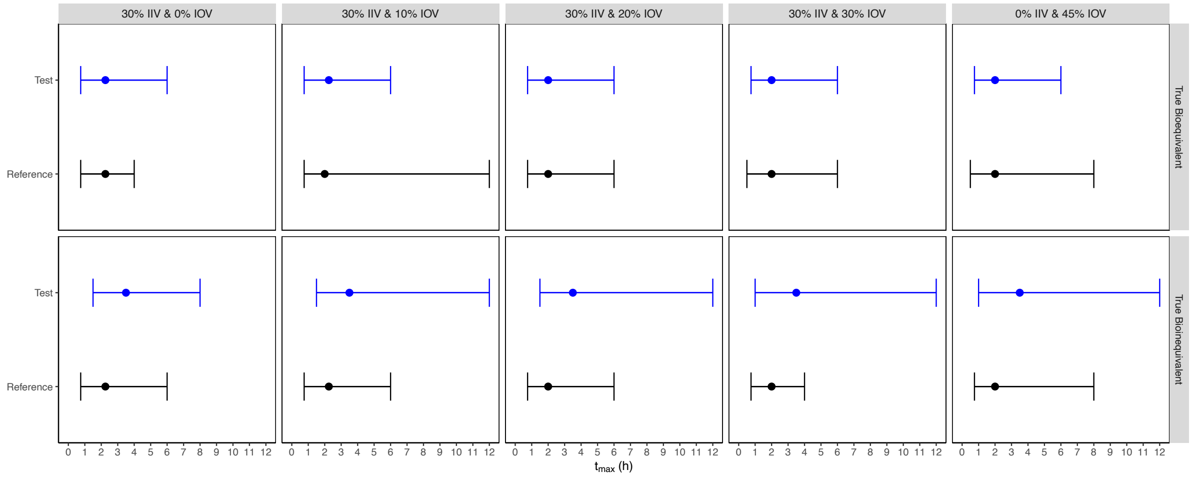

Figure S.104. Distribution of Untransformed t_{max}, Derived from Simulations for Studies with Variability in k_e

Table S.26. Descriptive Statistics of AUC_{0-t} (µg.h/L) Derived from Simulated True Bioequivalent Studies, with Variability in k_e

	30% IIV 0% IOV		30% IIV 10% IOV		30% IIV 20% IOV		30% IIV 30% IOV		0% IIV 45% IOV	
	**Reference**	**Test**	**Reference**	**Test**	**Reference**	**Test**	**Reference**	**Test**	**Reference**	**Test**
n	21000	21000	21000	21000	21000	21000	21000	21000	21000	21000
G_{Mean}	4872.59	4872.97	4883.33	4878.92	4870.85	4866.09	4835.58	4831.16	4814.2	4810.89
GSD	1.3	1.3	1.32	1.32	1.37	1.37	1.45	1.45	1.48	1.48
GCV%	27.0	27.0	28.5	28.4	32.5	32.4	38.2	38.4	40.5	40.4
G_{mean} 95% CI	4855.13 – 4890.11	4855.52 – 4890.49	4864.90 – 4901.83	4860.57 – 4897.34	4850.01 – 4891.78	4845.32 – 4886.94	4811.53 – 4859.76	4806.98 – 4855.47	4788.90 – 4839.63	4785.65 – 4836.27
A_{Mean}	5043.29	5043.58	5074.83	5066.72	5113.11	5106.6	5159.2	5159.84	5174.56	5169.19
SD	1313.6	1313.45	1507.99	1378.78	1569.57	1563.62	1821.93	1838.39	1927.83	1918.1
SE	9.06	9.06	10.41	9.51	10.83	10.79	12.57	12.69	13.3	13.24
CV%	26.0	26.0	29.7	27.2	30.7	30.6	35.3	35.6	37.3	37.1
Variance	1725557.08	1725163.5	2274030.2	1901046.31	2463558.63	2444914.56	3319444.63	3379664.63	3716540.76	3679117.58
A_{mean} 95% CI	5025.52 – 5061.05	5025.81 – 5061.34	5054.44 – 5095.23	5048.07 – 5085.37	5091.88 – 5134.34	5085.45 – 5127.75	5134.56 – 5183.84	5134.98 – 5184.71	5148.48 – 5200.63	5143.25 – 5195.14
Minimum	1776.47	1669.63	1465.75	879.2	1307.66	1207.47	709.2	985.4	677.63	840.65
Q1	4079.51	4076.01	4068.75	4066.06	3946.76	3958.95	3813.45	3783.17	3729.6	3723.16
Median	4931.06	4932.57	4943.16	4943.1	4967.49	4960.67	4954.92	4957.61	4936.67	4959.58
Q3	5881.37	5881.71	5955.85	5953.48	6126.15	6105.61	6305.69	6328.64	6381.03	6369.1
Maximum	11103.91	11430.32	93883.46	12544.22	13270.54	12332.6	13189.48	13454.17	14904.56	13558.98

n: Number of observations, G_{mean}: Geometric mean, GSD: Geometric standard deviation, GCV%: Geometric coefficient of variation, G_{mean} 95% CI: 95% confidence interval of the geometric mean, A_{mean}: Arithmetic mean, SD: Standard deviation, SD: Standard error, CV%: Coefficient of variation, A_{mean} 95% CI: 95% confidence interval of the arithmetic mean, Q1: 1st (25%) quartile, Q3: 3rd (75%) quartile

Table S.27. Descriptive Statistics of AUC_{0-t} (µg.h/L) Derived from Simulated True Bioinequivalent Studies, with Variability in k_e

	30% IIV 0% IOV		30% IIV 10% IOV		30% IIV 20% IOV		30% IIV 30% IOV		0% IIV 45% IOV	
	**Reference**	**Test**	**Reference**	**Test**	**Reference**	**Test**	**Reference**	**Test**	**Reference**	**Test**
n	21000	21000	21000	21000	21000	21000	21000	21000	21000	21000
G_{Mean}	4885.7	4771.83	4881.77	4762.64	4862.29	4739.78	4827.06	4697.43	4832.85	4706.71
GSD	1.30	1.29	1.32	1.31	1.37	1.36	1.45	1.43	1.48	1.45
GCV%	27.0	26.3	28.3	27.4	32.6	31.8	38.1	36.8	40.5	38.8
G_{mean} 95% CI	4868.17 – 4903.29	4755.19 – 4788.53	4863.48 – 4900.13	4745.32 – 4780.02	4841.44 – 4883.23	4719.92 – 4759.72	4803.06 – 4851.18	4674.82 – 4720.14	4807.44 – 4858.39	4682.94 – 4730.61
A_{Mean}	5056.95	4929.48	5068.64	4933.94	5105.5	4965.13	5150.46	4992.23	5193.87	5031.09
SD	1316.04	1243.32	1377.92	1299.34	1575.1	1490.47	1820.48	1709.81	1924.9	1796.24
SE	9.08	8.58	9.51	8.97	10.87	10.29	12.56	11.8	13.28	12.4
CV%	26.0	25.2	27.2	26.3	30.9	30	35.3	34.2	37.1	35.7
Variance	1731971.3	1545846.87	1898665.04	1688273.8	2480938.01	2221501.06	3314145.45	2923449.09	3705258.86	3226493.78
A_{mean} 95% CI	5039.15 – 5074.76	4912.67 – 4946.30	5050.00 – 5087.28	4916.36 – 4951.51	5084.19 – 5126.80	4944.97 – 4985.29	5125.83 – 5175.08	4969.10 – 5015.36	5167.84 – 5219.91	5006.79 – 5055.38
Minimum	1147.21	565.86	1421.28	1210.86	1374.72	469.55	891.83	991.71	880.61	616.86
Q1	4106.53	4035.64	4071.13	4001.62	3950.97	3877.73	3787.66	3717.03	3739.74	3689.06
Median	4948.57	4835.11	4945.24	4827.53	4942.25	4831.85	4946.62	4817.77	4975.63	4841.65
Q3	5900.15	5725.45	5935.78	5757.59	6108.85	5906.36	6305.37	6093.03	6442.52	6169.44
Maximum	11130.2	10650.86	12470.64	15224.74	12056.67	13001.76	12513.72	12091.63	14039.14	13820.1

n: Number of observations, G_{mean}: Geometric mean, GSD: Geometric standard deviation, GCV%: Geometric coefficient of variation, G_{mean} 95% CI: 95% confidence interval of the geometric mean, A_{mean}: Arithmetic mean, SD: Standard deviation, SD: Standard error, CV%: Coefficient of variation, A_{mean} 95% CI: 95% confidence interval of the arithmetic mean, Q1: 1st (25%) quartile, Q3: 3rd (75%) quartile

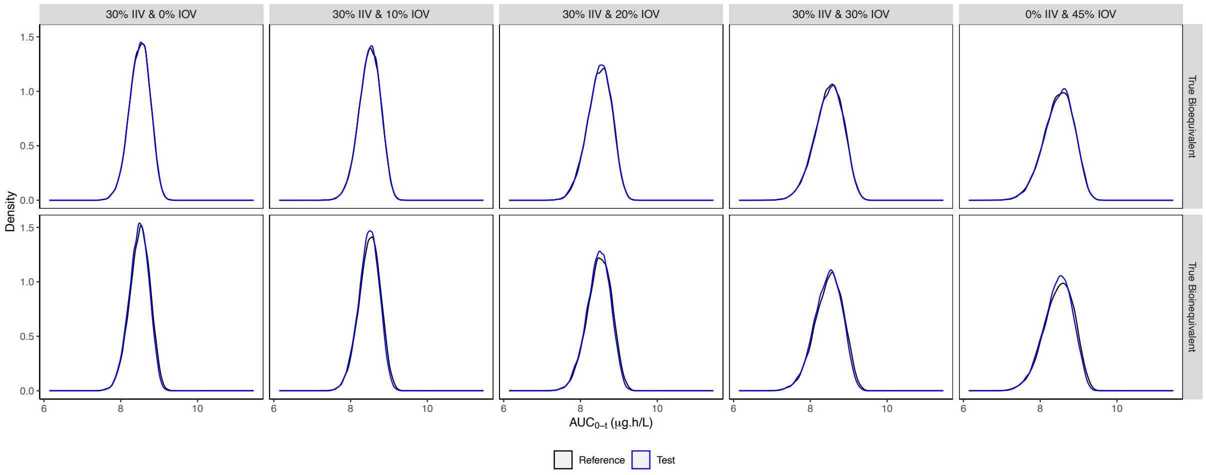

Figure S.105. Distribution of *Ln*-Transformed AUC$_{0\text{-}t}$, Derived from Simulations for Studies with Variability in k$_e$

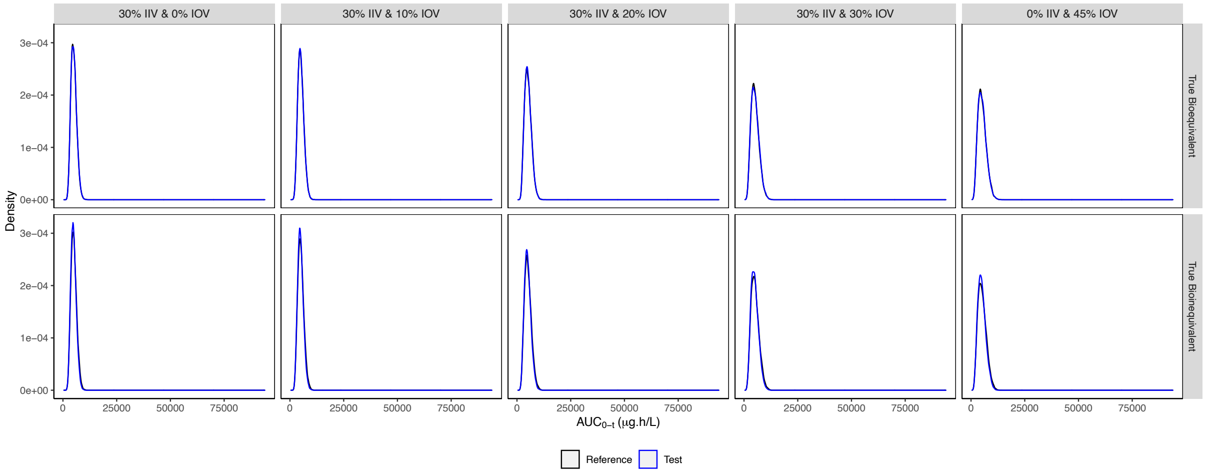

Figure S.106. Distribution of Untransformed AUC$_{0\text{-}t}$, Derived from Simulations for Studies with Variability in k$_e$

Appendix SA.4.4.   Simulated Pilot Studies Results

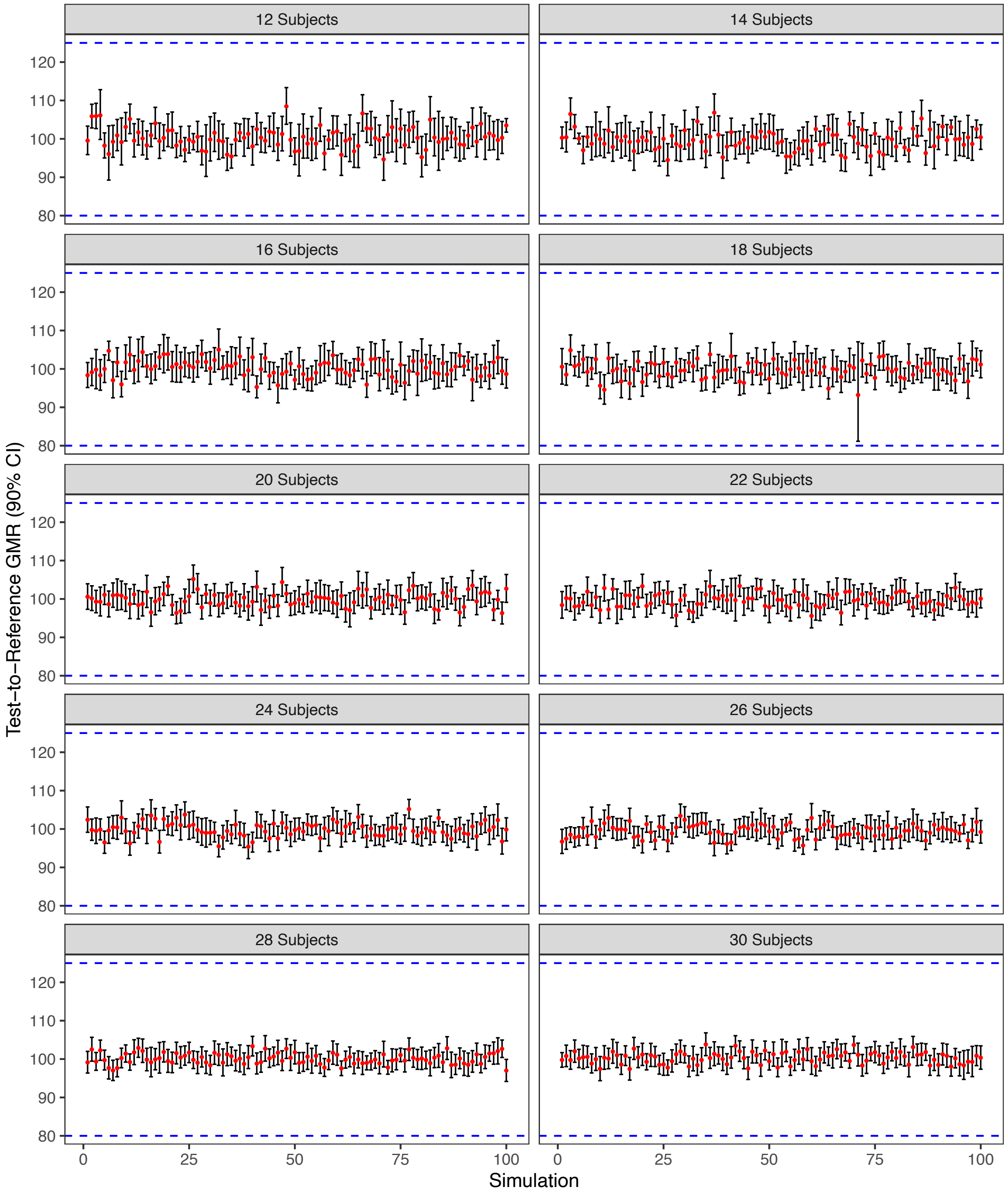

Figure S.107. Average Bioequivalence Analysis Results (Test-to-Reference GMR and Corresponding 90% CI) for C_{max} Derived from True Bioequivalent Simulations with 30% IIV & 0% IOV in k_e

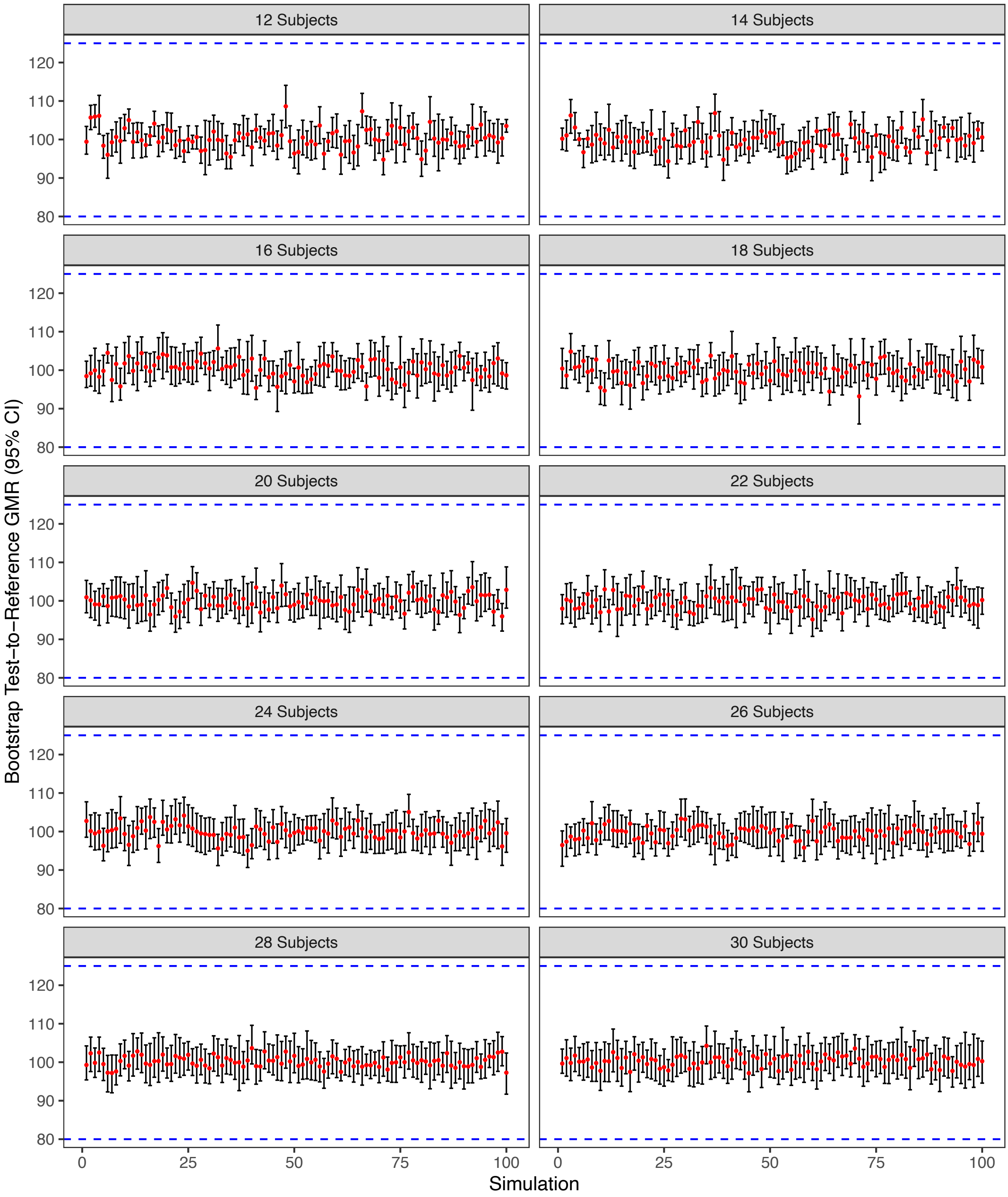

Figure S.108. Bootstrap Bioequivalence Analysis Results (Test-to-Reference GMR and Corresponding 95% CI) for C_{max} Derived from True Bioequivalent Simulations with 30% IIV & 0% IOV in k_e

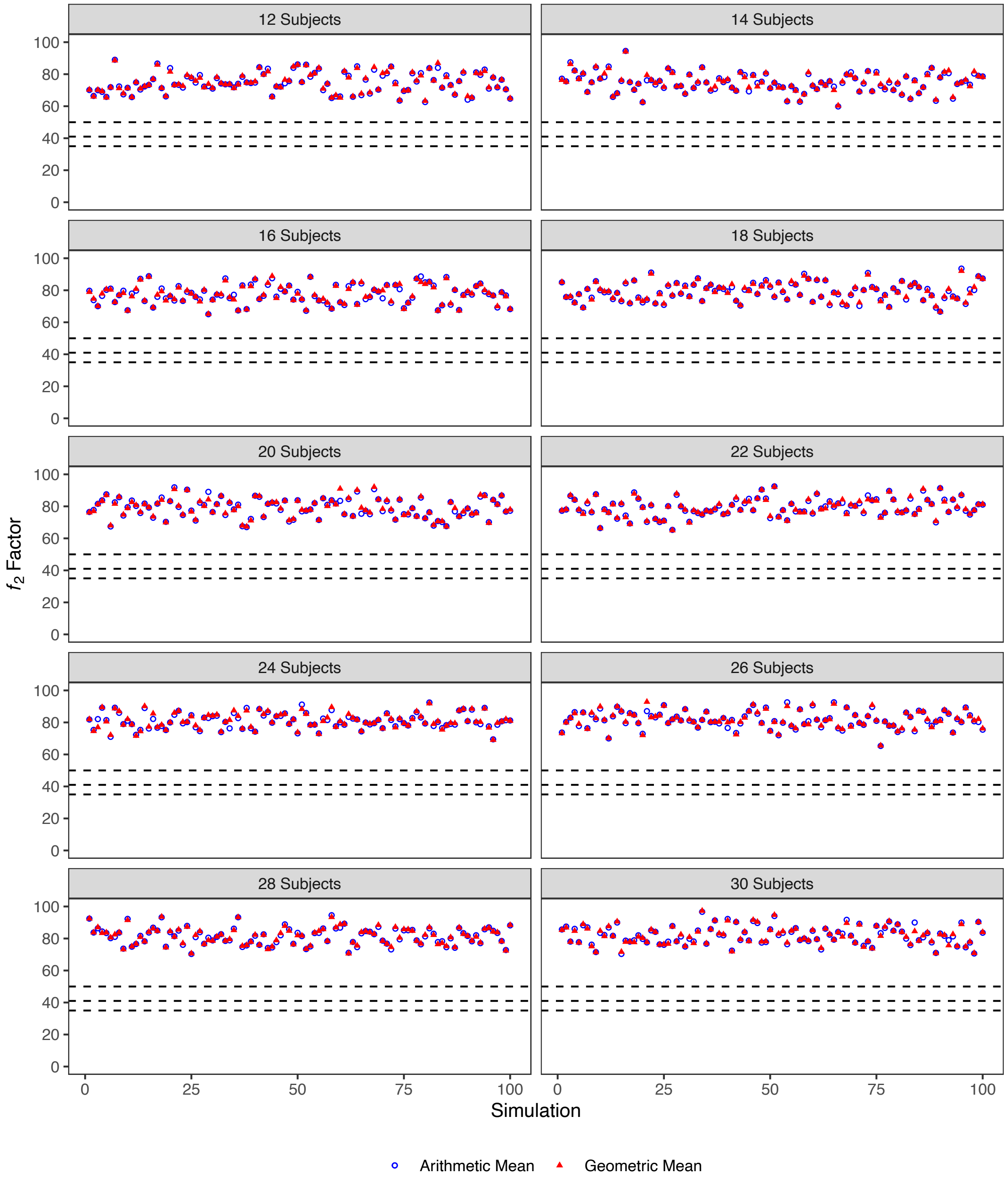

Figure S.109. Similarity f_2 Factor Results, Derived from Arithmetic and Geometric Mean Concentration-Time Profiles Until the Reference C_{max}, Derived from True Bioequivalent Simulations with 30% IIV & 0% IOV in k_e

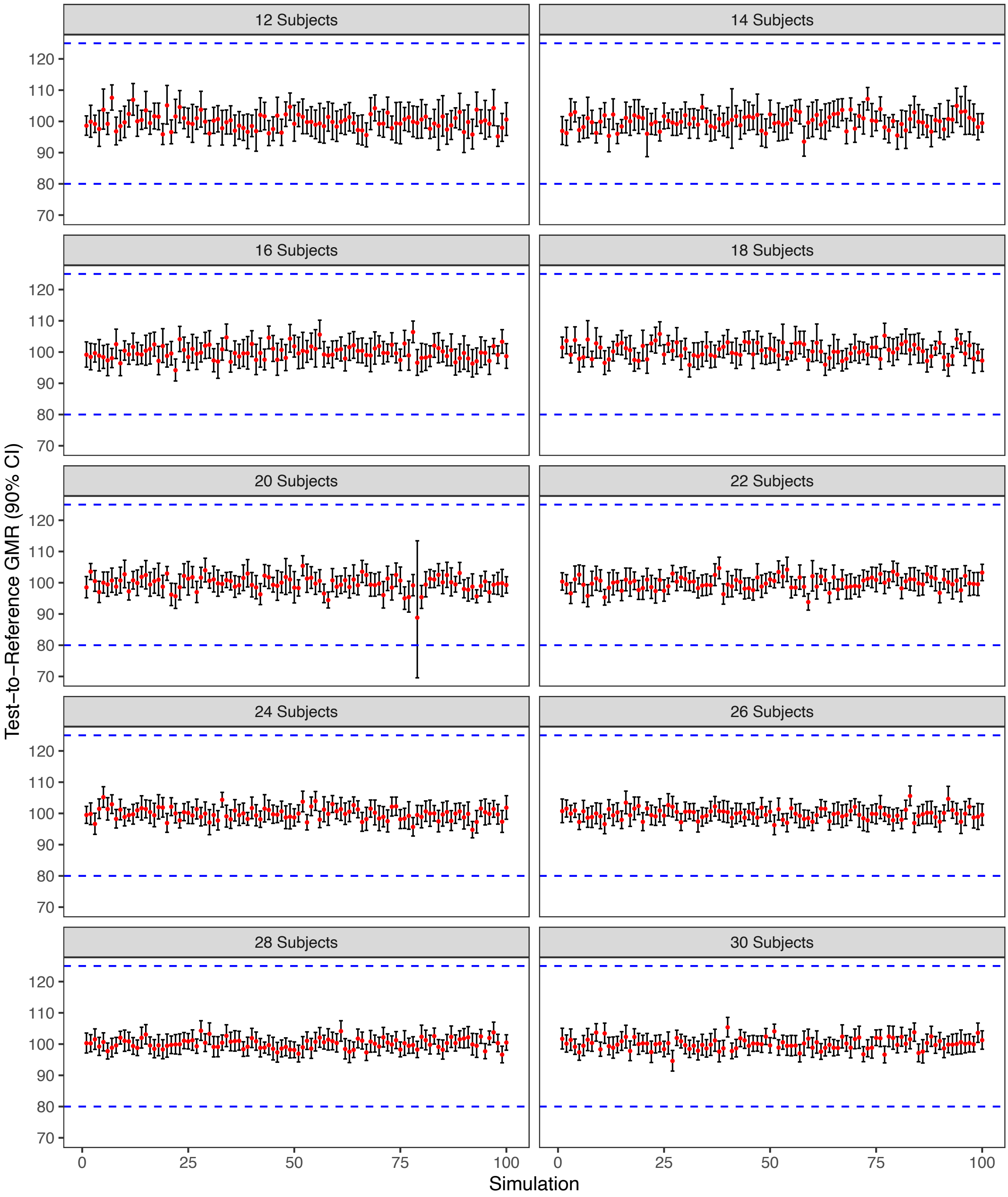

Figure S.110. Average Bioequivalence Analysis Results (Test-to-Reference GMR and Corresponding 90% CI) for C_{max} Derived from True Bioequivalent Simulations with 30% IIV & 10% IOV in k_e

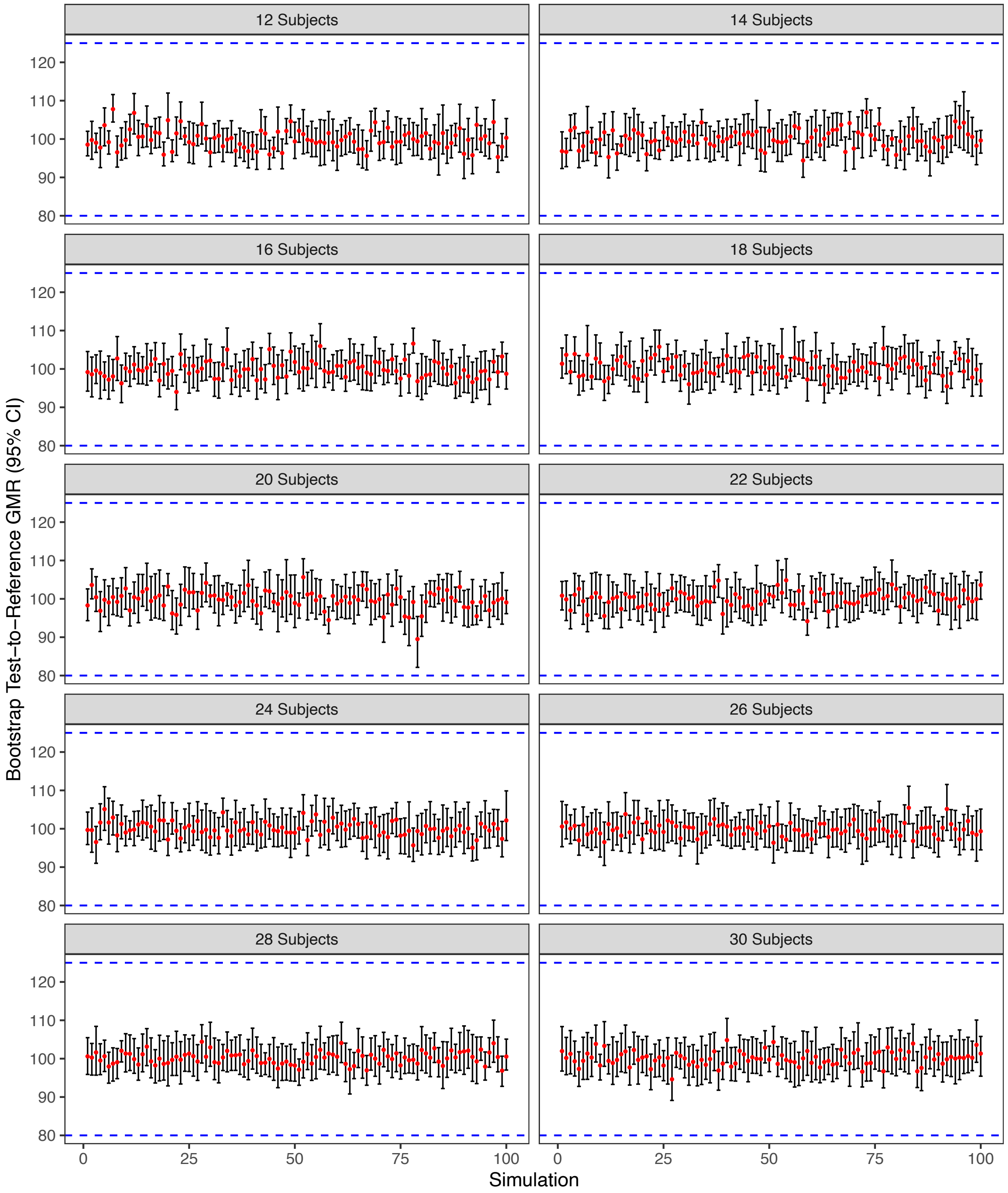

Figure S.111. Bootstrap Bioequivalence Analysis Results (Test-to-Reference GMR and Corresponding 95% CI) for C_{max} Derived from True Bioequivalent Simulations with 30% IIV & 10% IOV in k_e

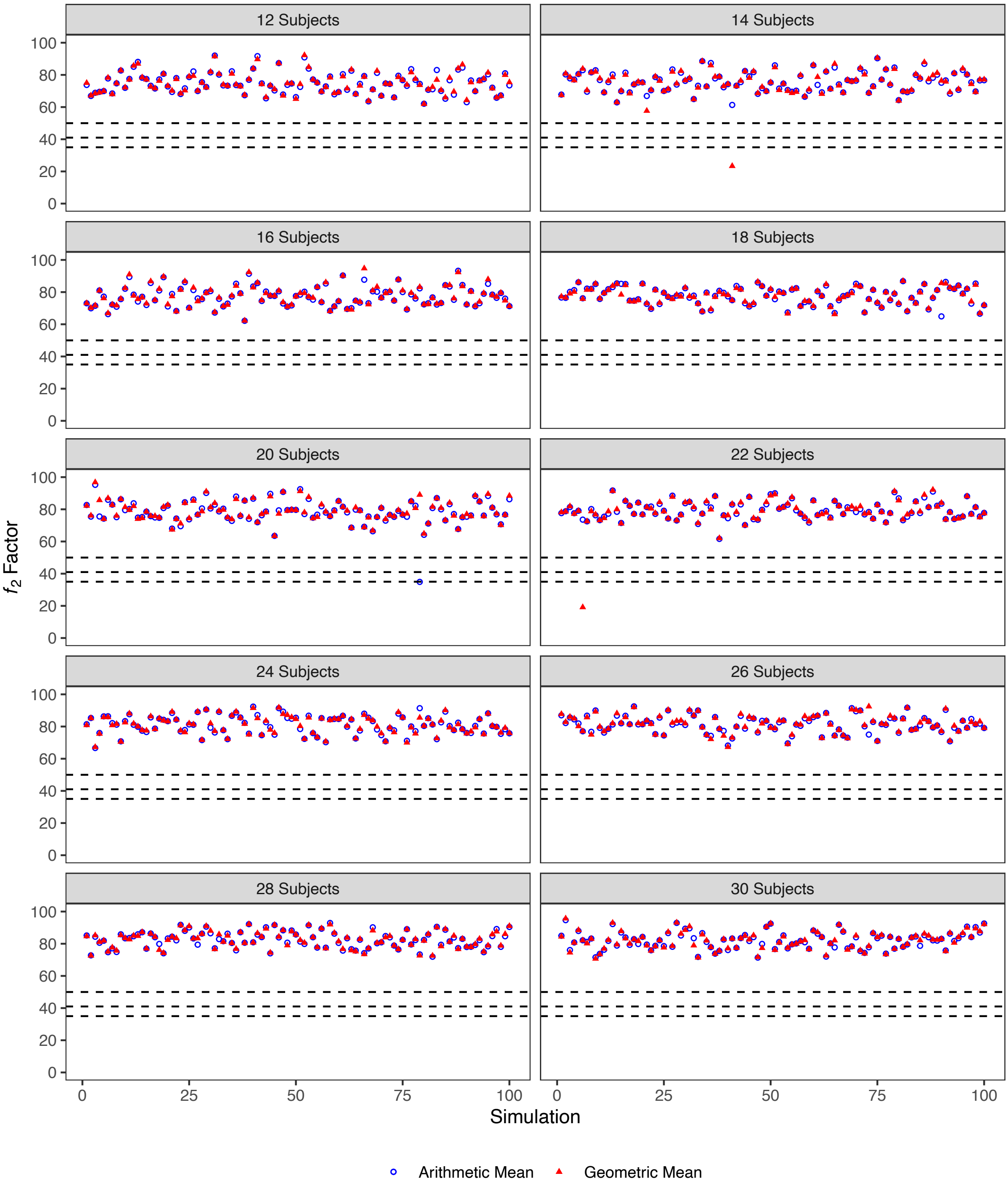

Figure S.112. Similarity f_2 Factor Results, Derived from Arithmetic and Geometric Mean Concentration-Time Profiles Until the Reference C_{max}, Derived from True Bioequivalent Simulations with 30% IIV & 10% IOV in k_e

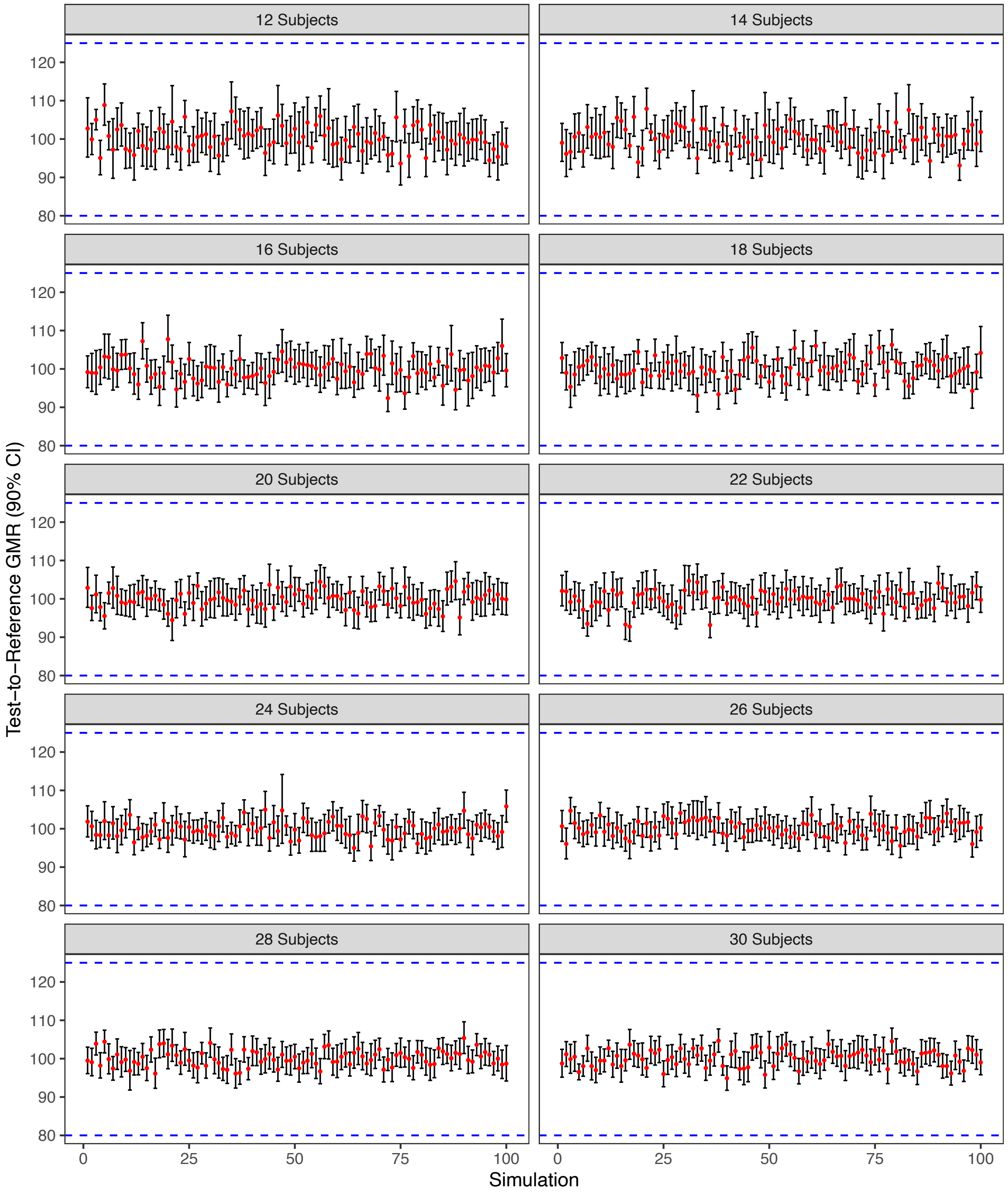

Figure S.113. Average Bioequivalence Analysis Results (Test-to-Reference GMR and Corresponding 90% CI) for C_{max} Derived from True Bioequivalent Simulations with 30% IIV & 20% IOV in k_e

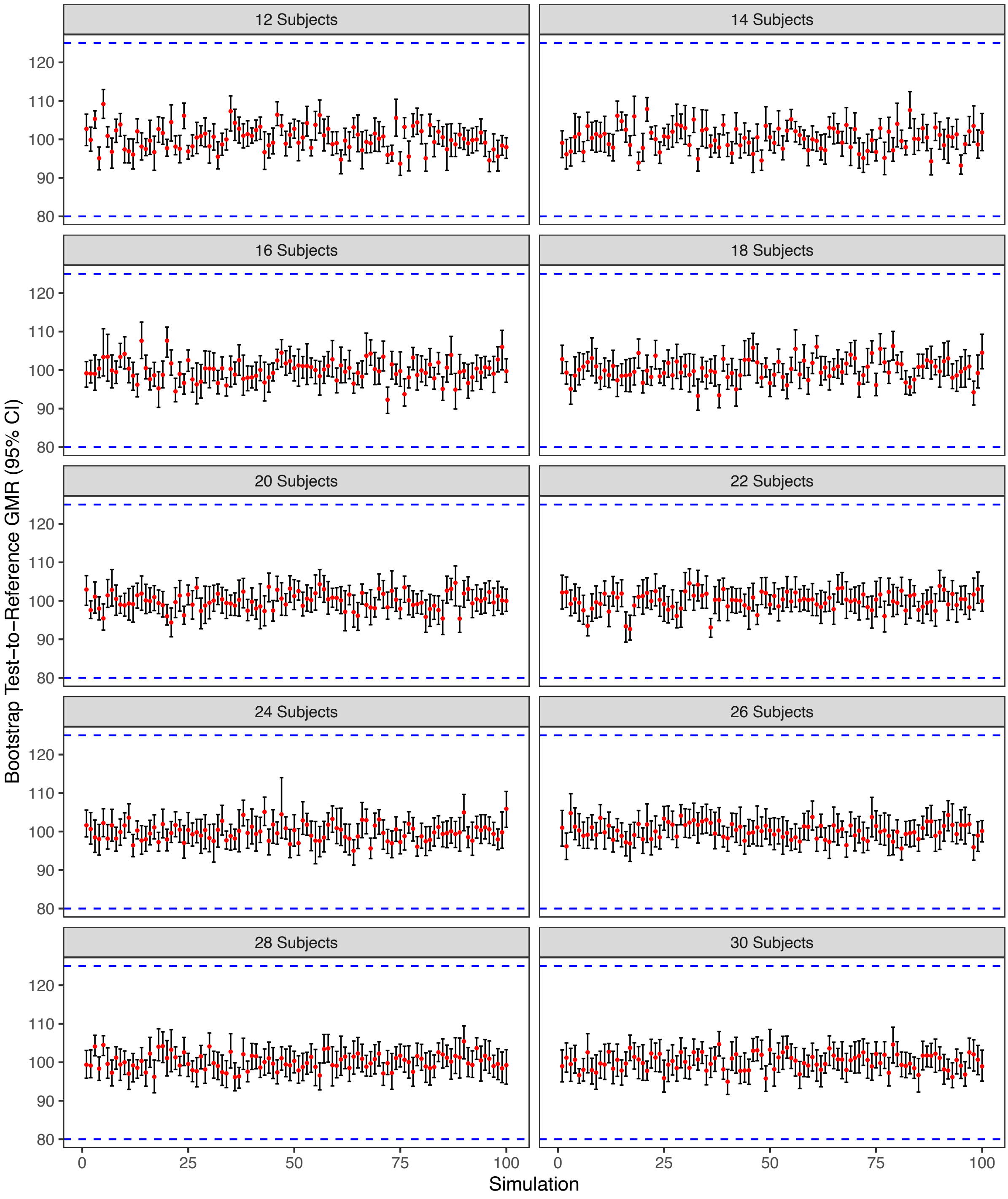

Figure S.114. Bootstrap Bioequivalence Analysis Results (Test-to-Reference GMR and Corresponding 95% CI) for C_{max} Derived from True Bioequivalent Simulations with 30% IIV & 20% IOV in k_e

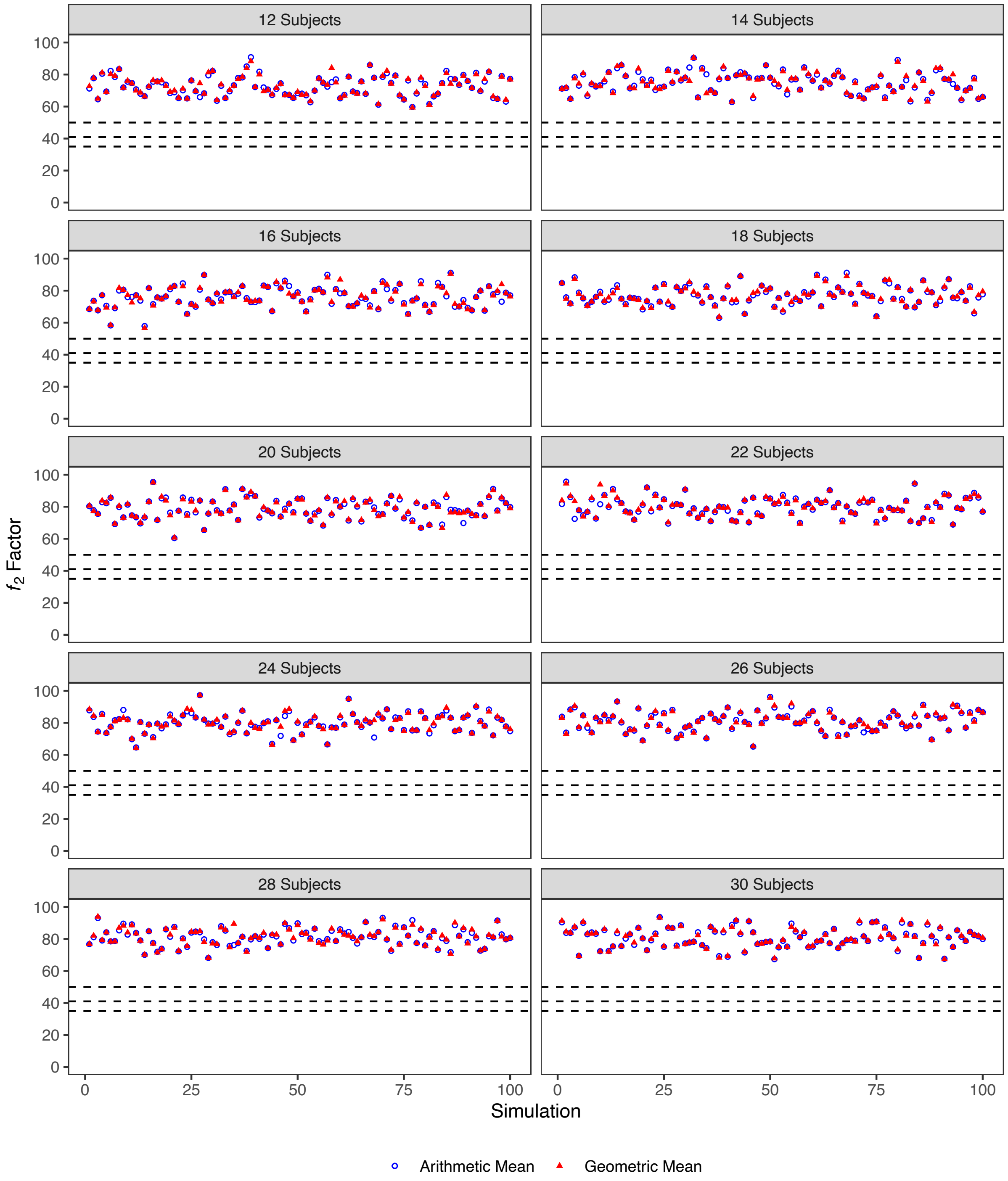

Figure S.115. Similarity f_2 Factor Results, Derived from Arithmetic and Geometric Mean Concentration-Time Profiles Until the Reference C_{max}, Derived from True Bioequivalent Simulations with 30% IIV & 20% IOV in k_e

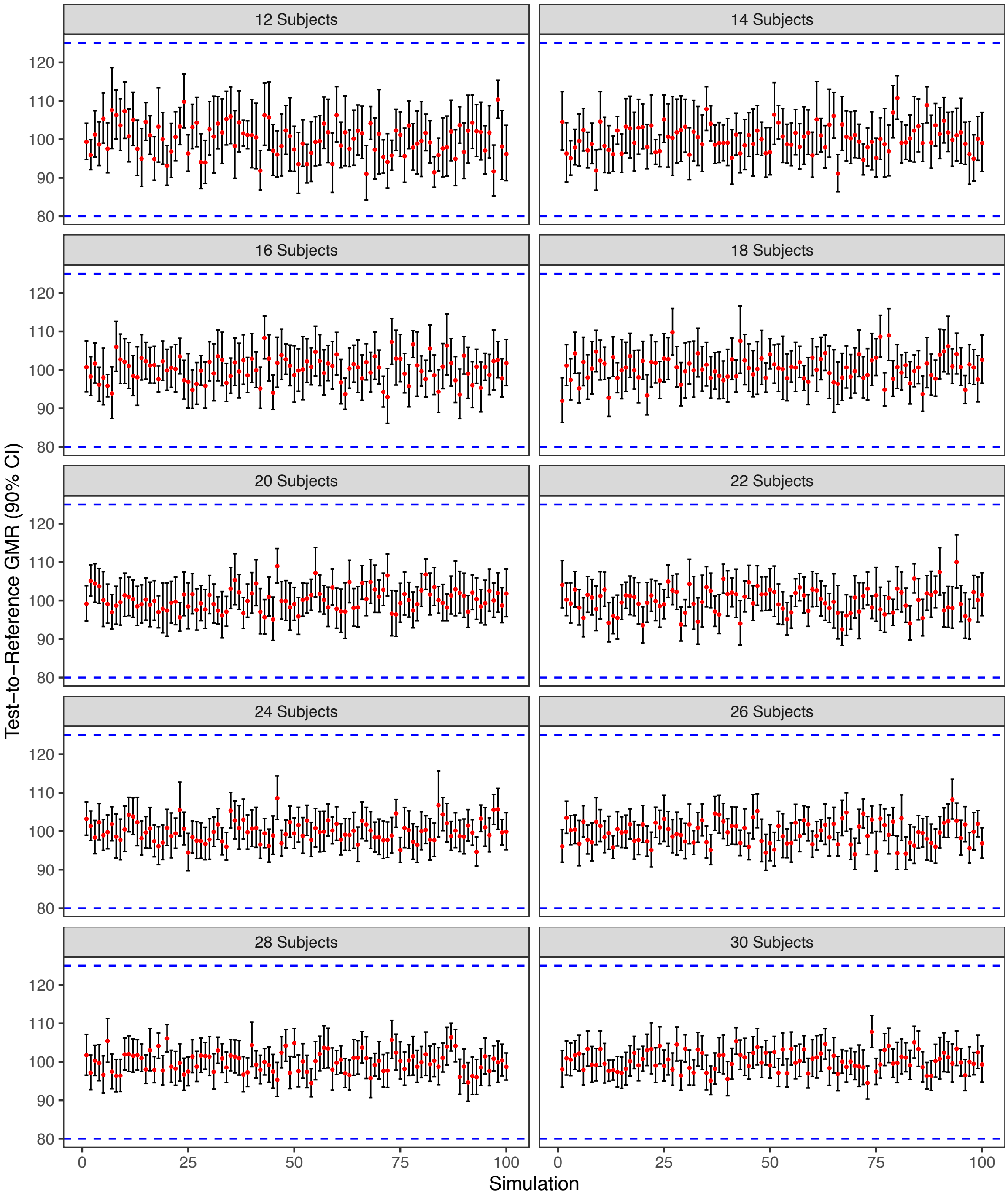

Figure S.116. Average Bioequivalence Analysis Results (Test-to-Reference GMR and Corresponding 90% CI) for C_{max} Derived from True Bioequivalent Simulations with 30% IIV & 30% IOV in k_e

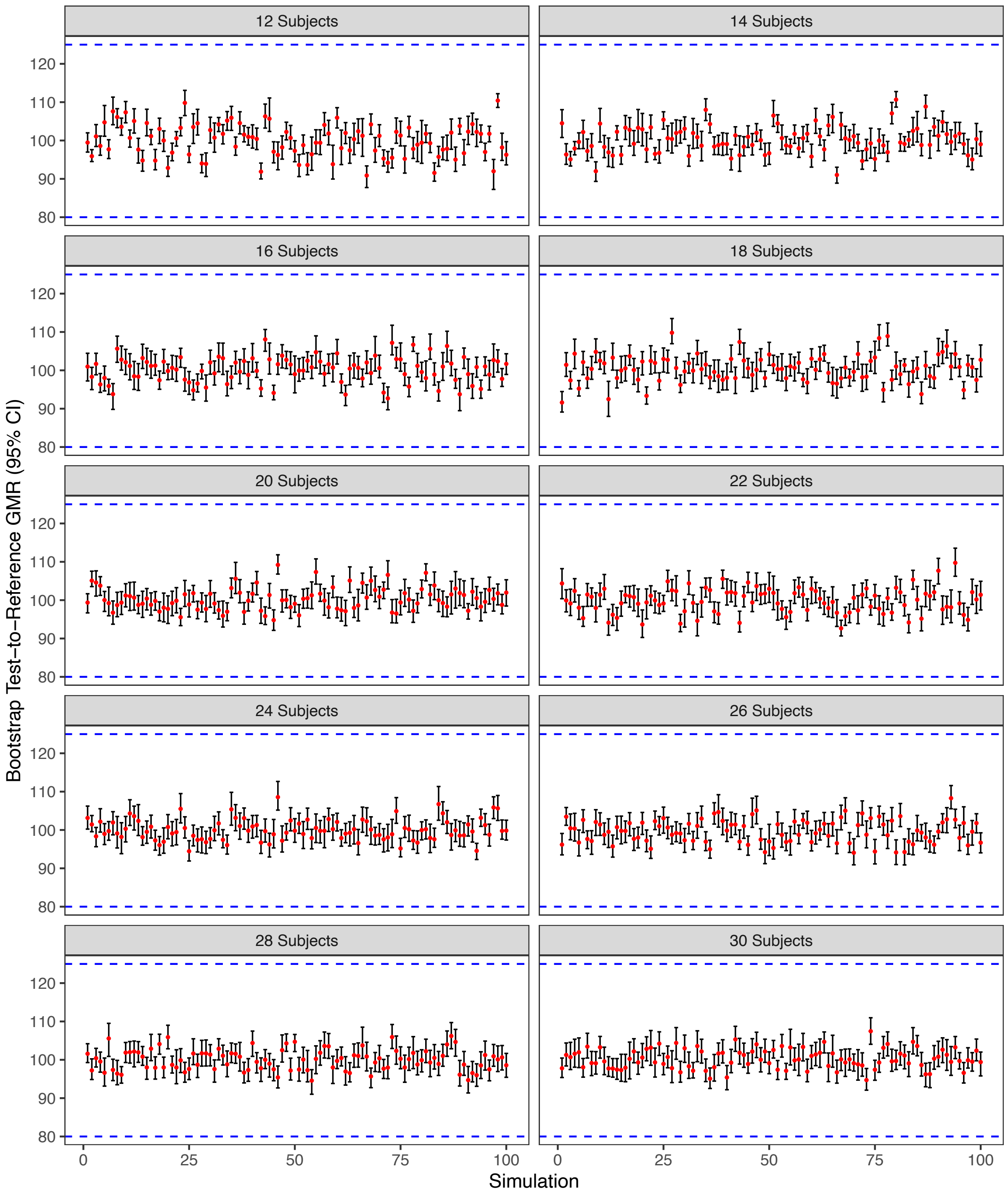

Figure S.117. Bootstrap Bioequivalence Analysis Results (Test-to-Reference GMR and Corresponding 95% CI) for C_{max} Derived from True Bioequivalent Simulations with 30% IIV & 30% IOV in k_e

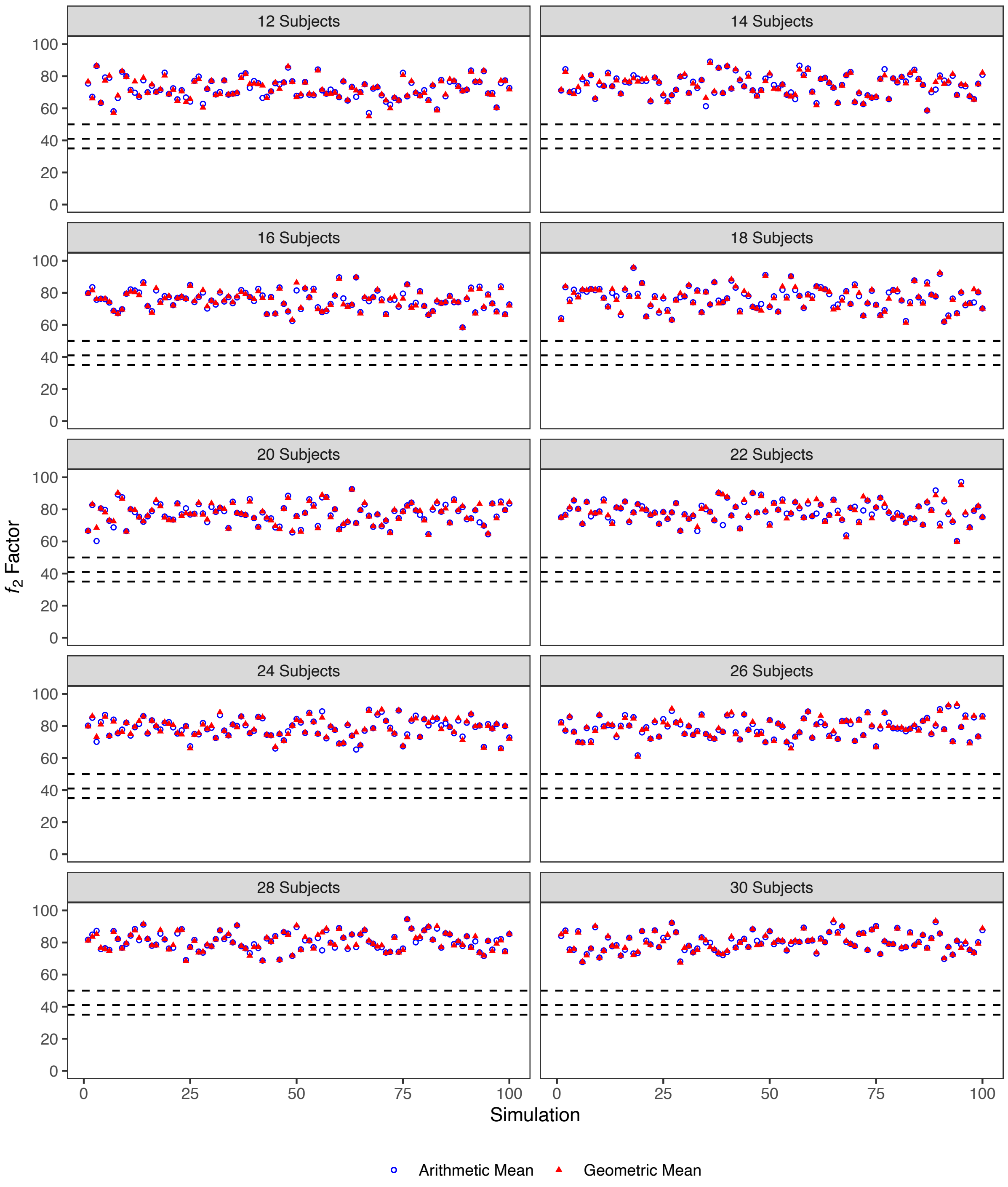

Figure S.118. Similarity f_2 Factor Results, Derived from Arithmetic and Geometric Mean Concentration-Time Profiles Until the Reference C_{max}, Derived from True Bioequivalent Simulations with 30% IIV & 30% IOV in k_e

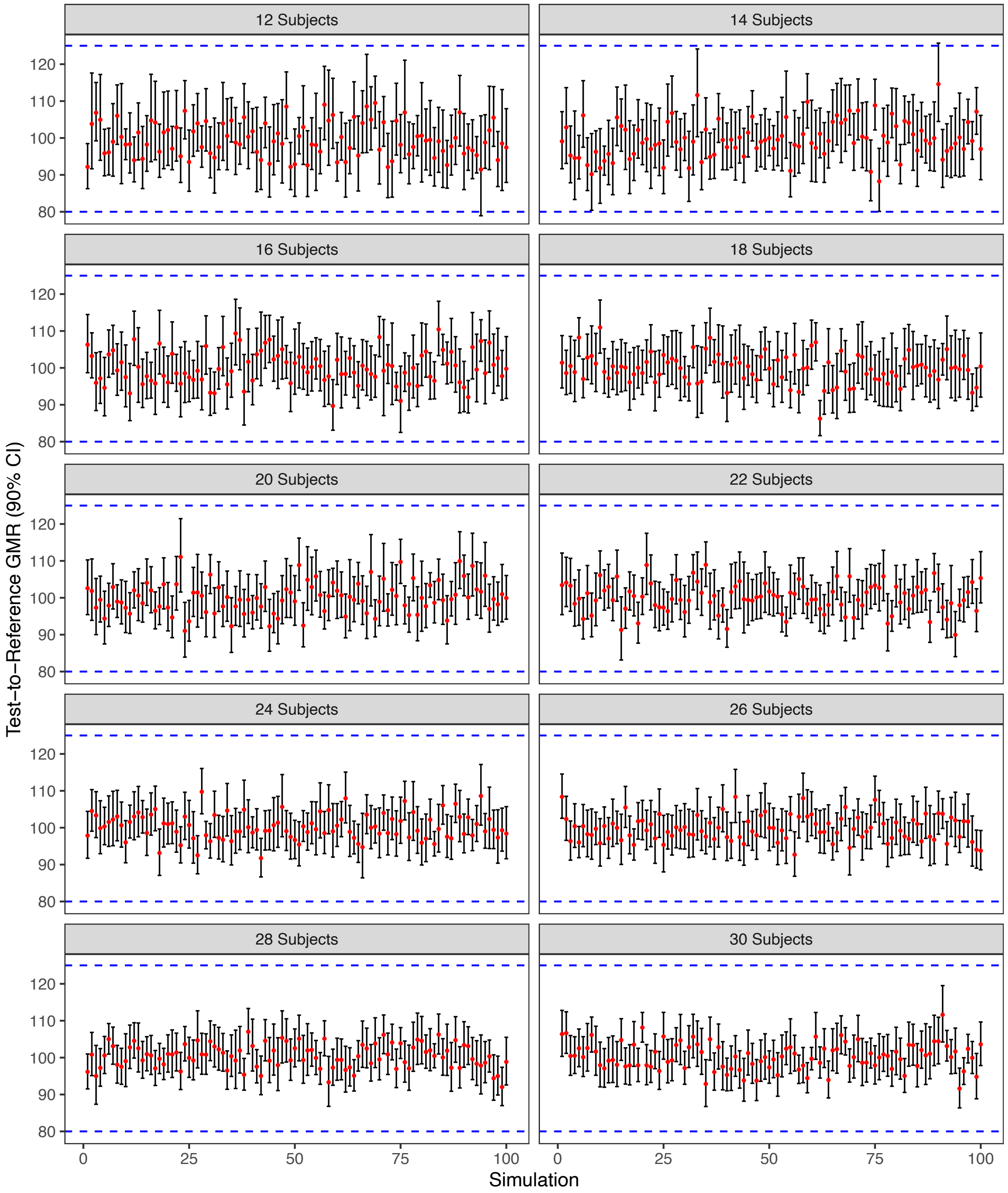

Figure S.119. Average Bioequivalence Analysis Results (Test-to-Reference GMR and Corresponding 90% CI) for C_{max} Derived from True Bioequivalent Simulations with 0% IIV & 45% IOV in k_e

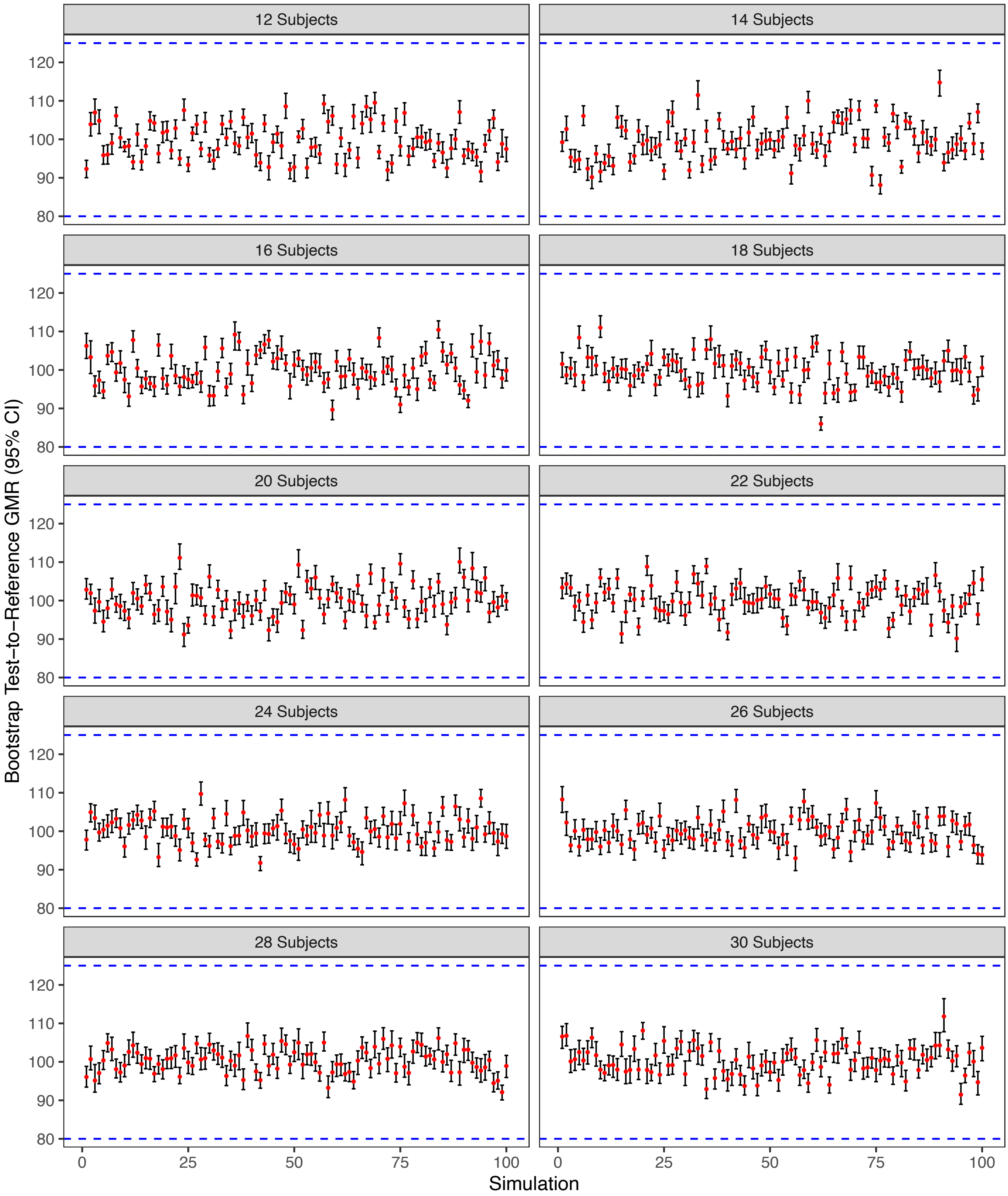

Figure S.120. Bootstrap Bioequivalence Analysis Results (Test-to-Reference GMR and Corresponding 95% CI) for C_{max} Derived from True Bioequivalent Simulations with 0% IIV & 45% IOV in k_e

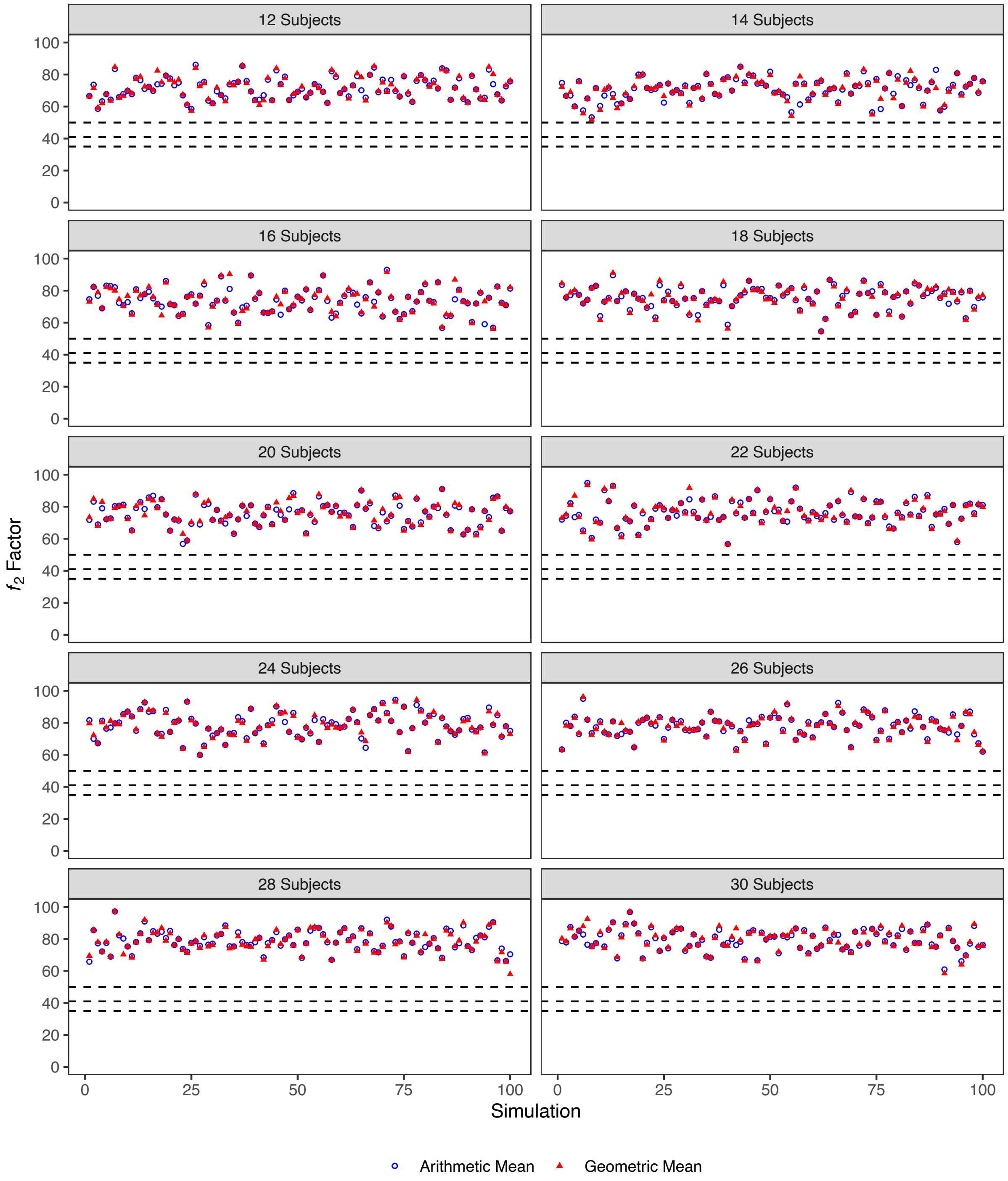

Figure S.121. Similarity f_2 Factor Results, Derived from Arithmetic and Geometric Mean Concentration-Time Profiles Until the Reference C_{max}, Derived from True Bioequivalent Simulations with 0% IIV & 45% IOV in k_e

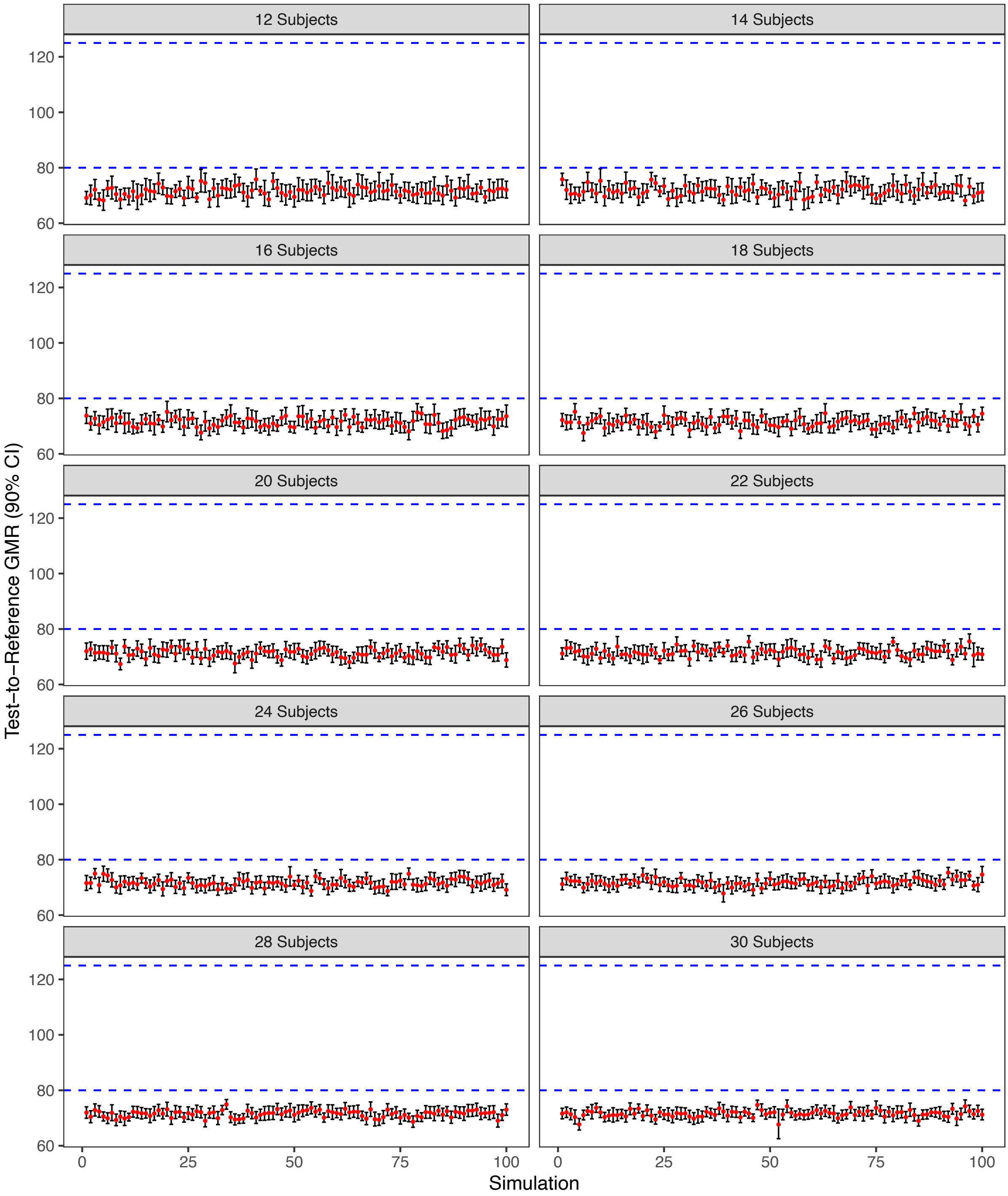

Figure S.122. Average Bioequivalence Analysis Results (Test-to-Reference GMR and Corresponding 90% CI) for C_{max} Derived from True Bioinequivalent Simulations with 30% IIV & 0% IOV in k_e

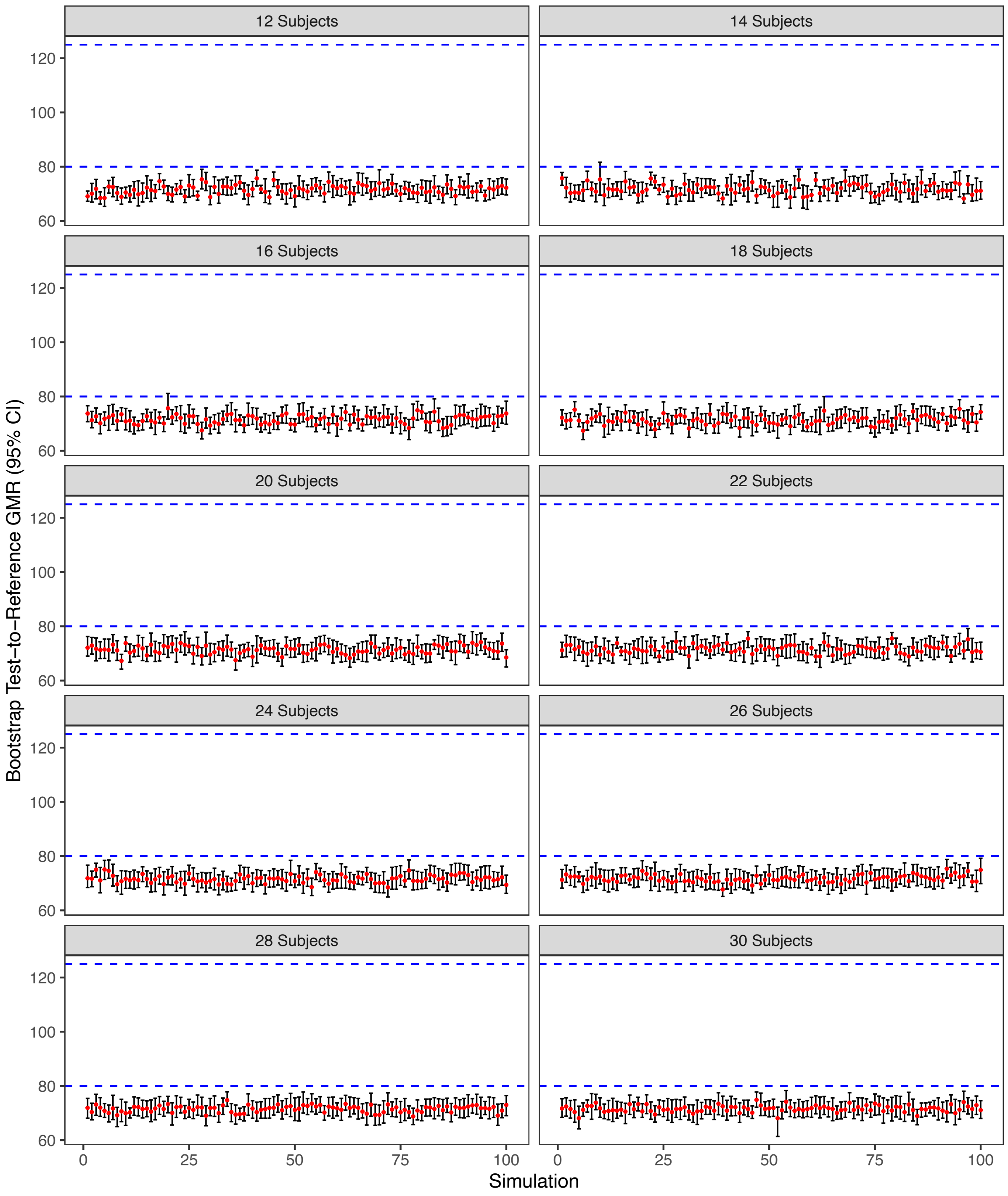

Figure S.123. Bootstrap Bioequivalence Analysis Results (Test-to-Reference GMR and Corresponding 95% CI) for C_{max} Derived from True Bioinequivalent Simulations with 30% IIV & 0% IOV in k_e

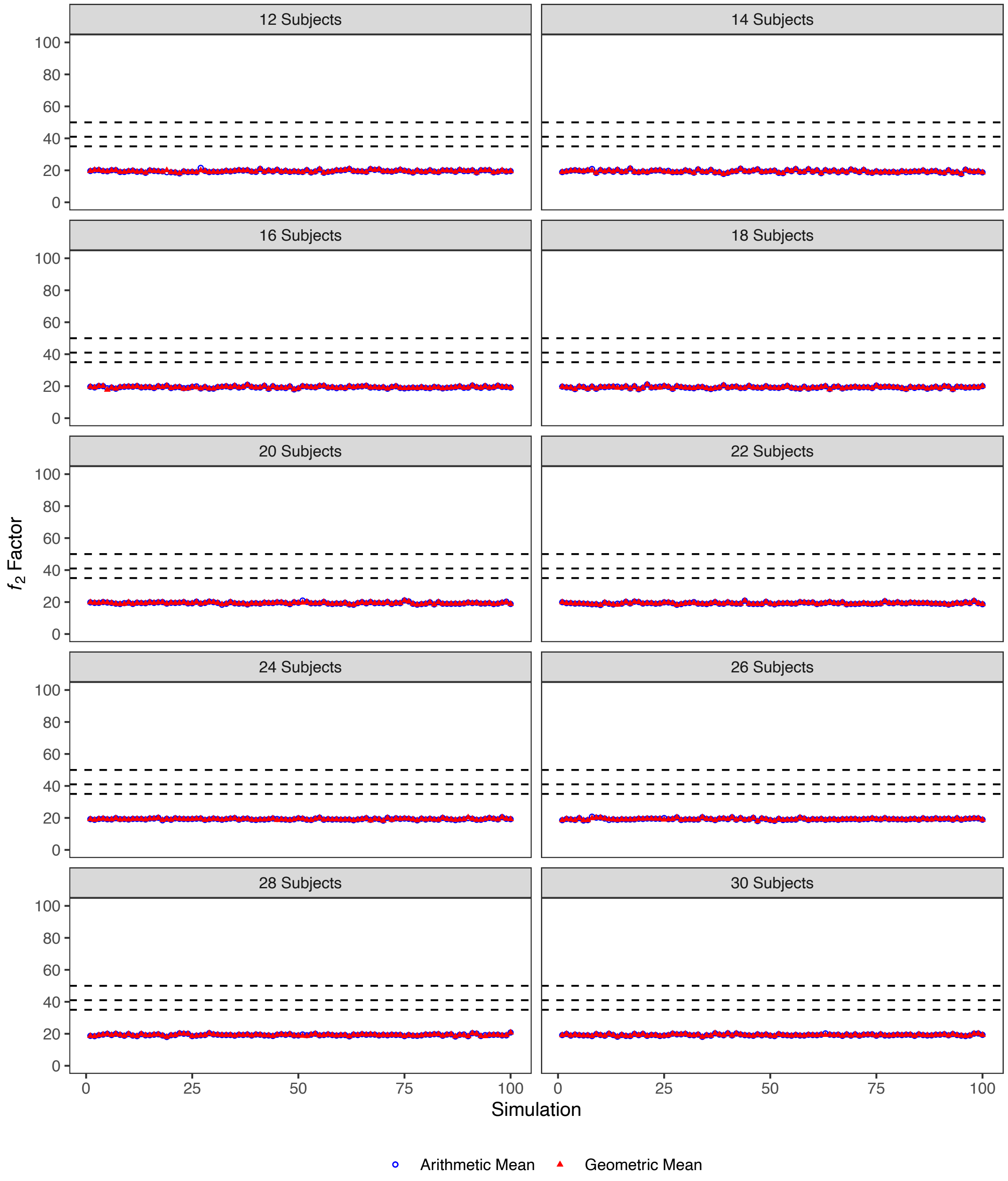

Figure S.124. Similarity f_2 Factor Results, Derived from Arithmetic and Geometric Mean Concentration-Time Profiles Until the Reference C_{max}, Derived from True Bioinequivalent Simulations with 30% IIV & 0% IOV in k_e

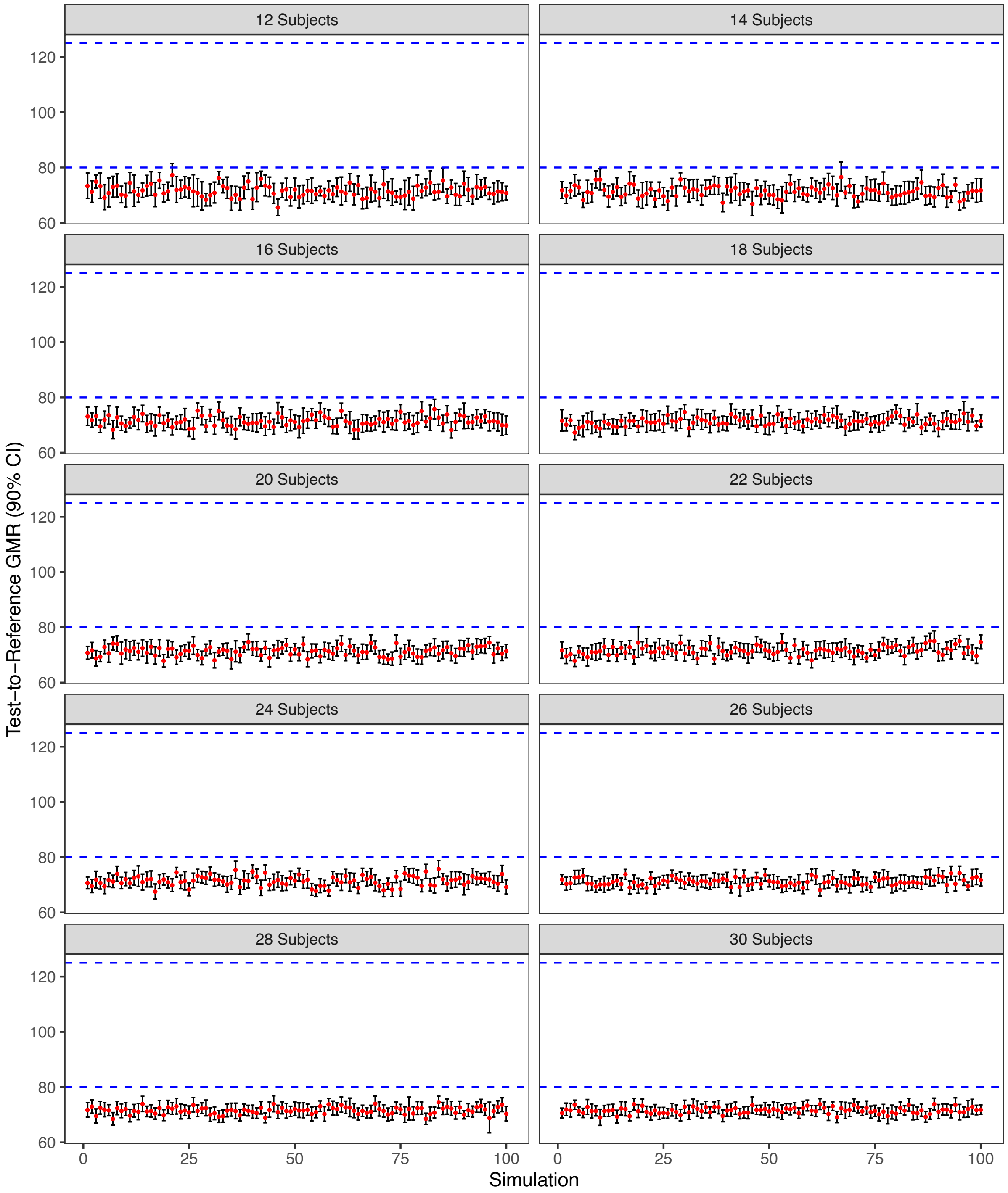

Figure S.125. Average Bioequivalence Analysis Results (Test-to-Reference GMR and Corresponding 90% CI) for C_{max} Derived from True Bioinequivalent Simulations with 30% IIV & 10% IOV in k_e

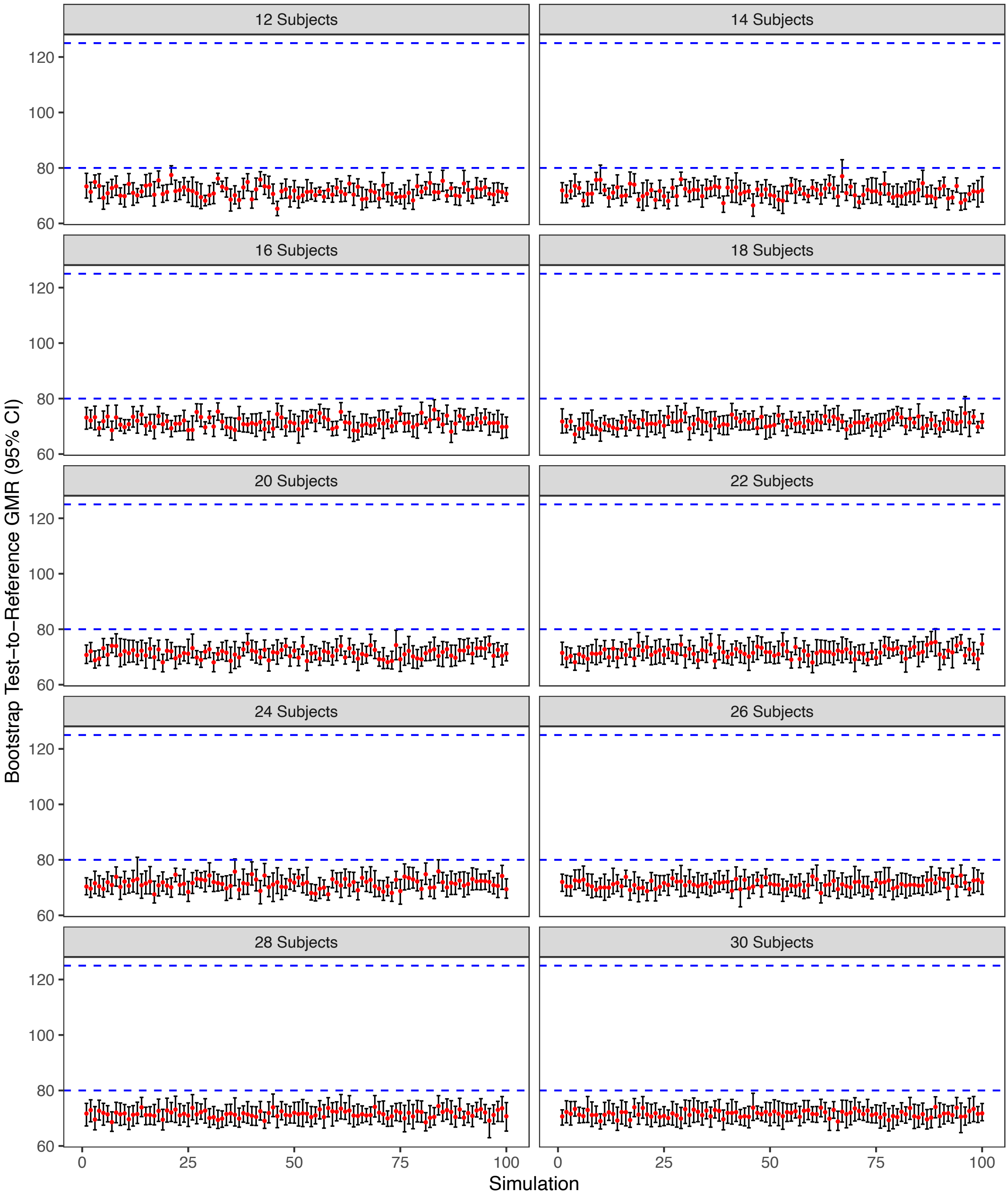

Figure S.126. Bootstrap Bioequivalence Analysis Results (Test-to-Reference GMR and Corresponding 95% CI) for C_{max} Derived from True Bioinequivalent Simulations with 30% IIV & 10% IOV in k_e

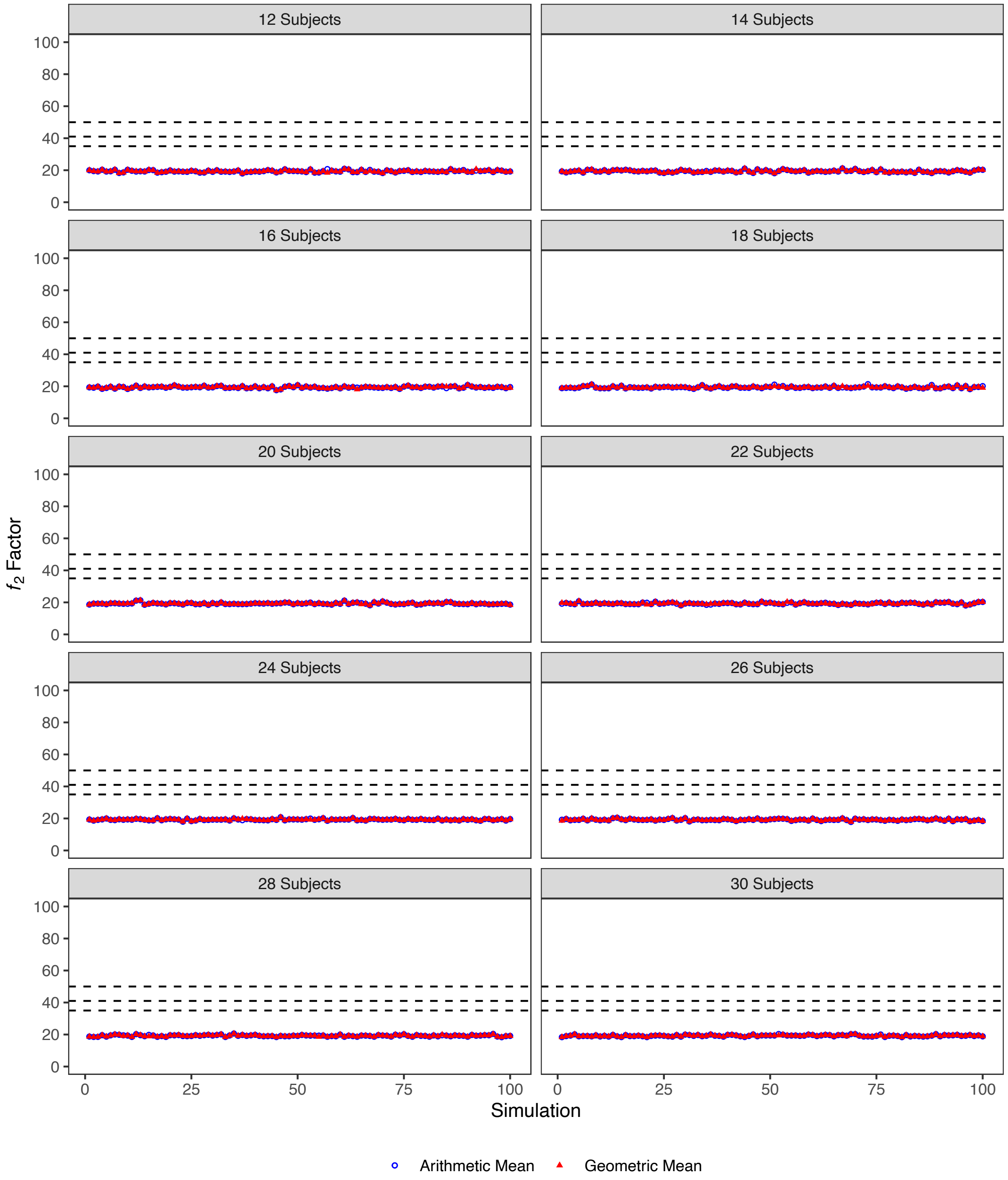

Figure S.127. Similarity f_2 Factor Results, Derived from Arithmetic and Geometric Mean Concentration-Time Profiles Until the Reference C_{max}, Derived from True Bioinequivalent Simulations with 30% IIV & 10% IOV in k_e

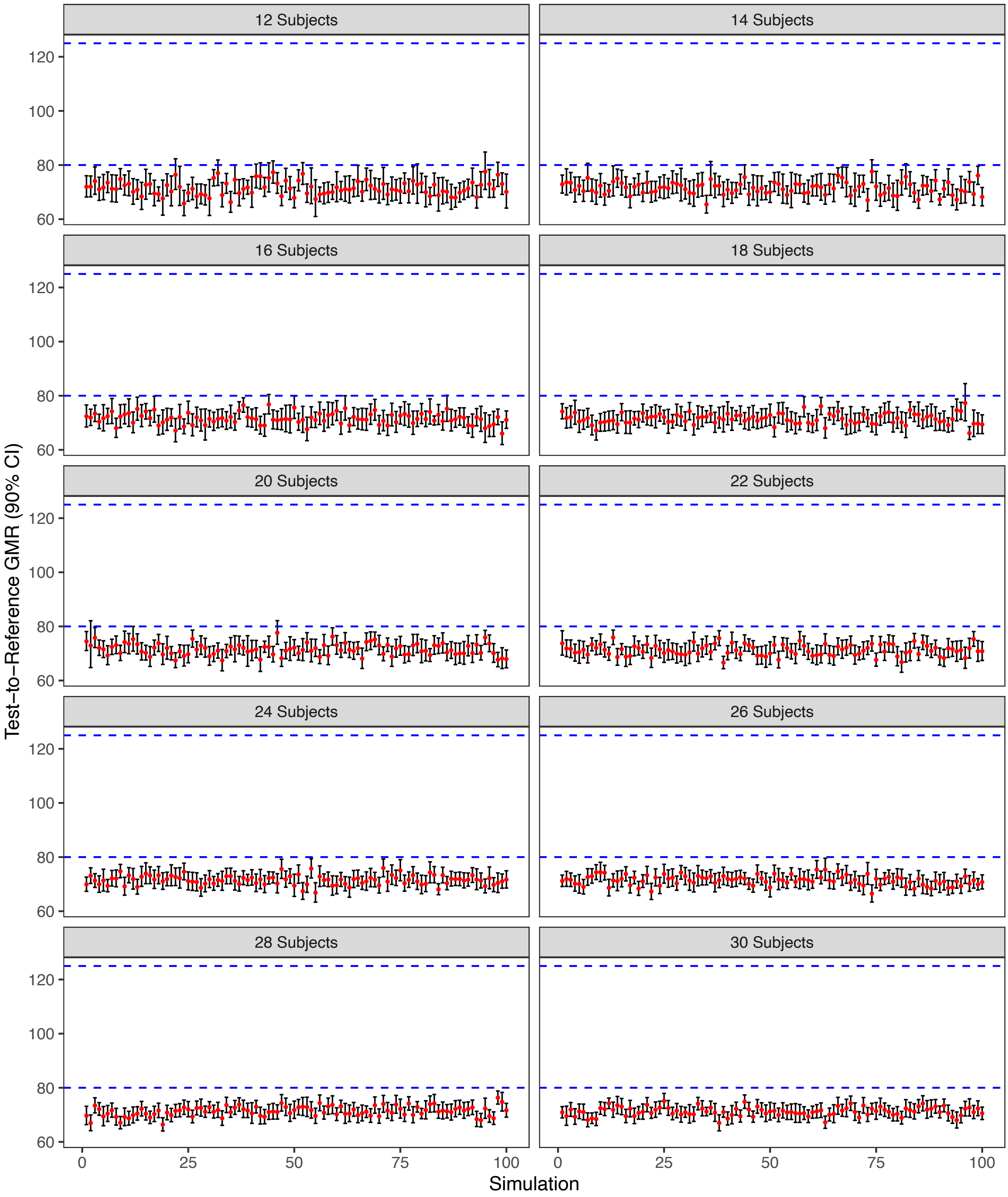

Figure S.128. Average Bioequivalence Analysis Results (Test-to-Reference GMR and Corresponding 90% CI) for C_{max} Derived from True Bioinequivalent Simulations with 30% IIV & 20% IOV in k_e

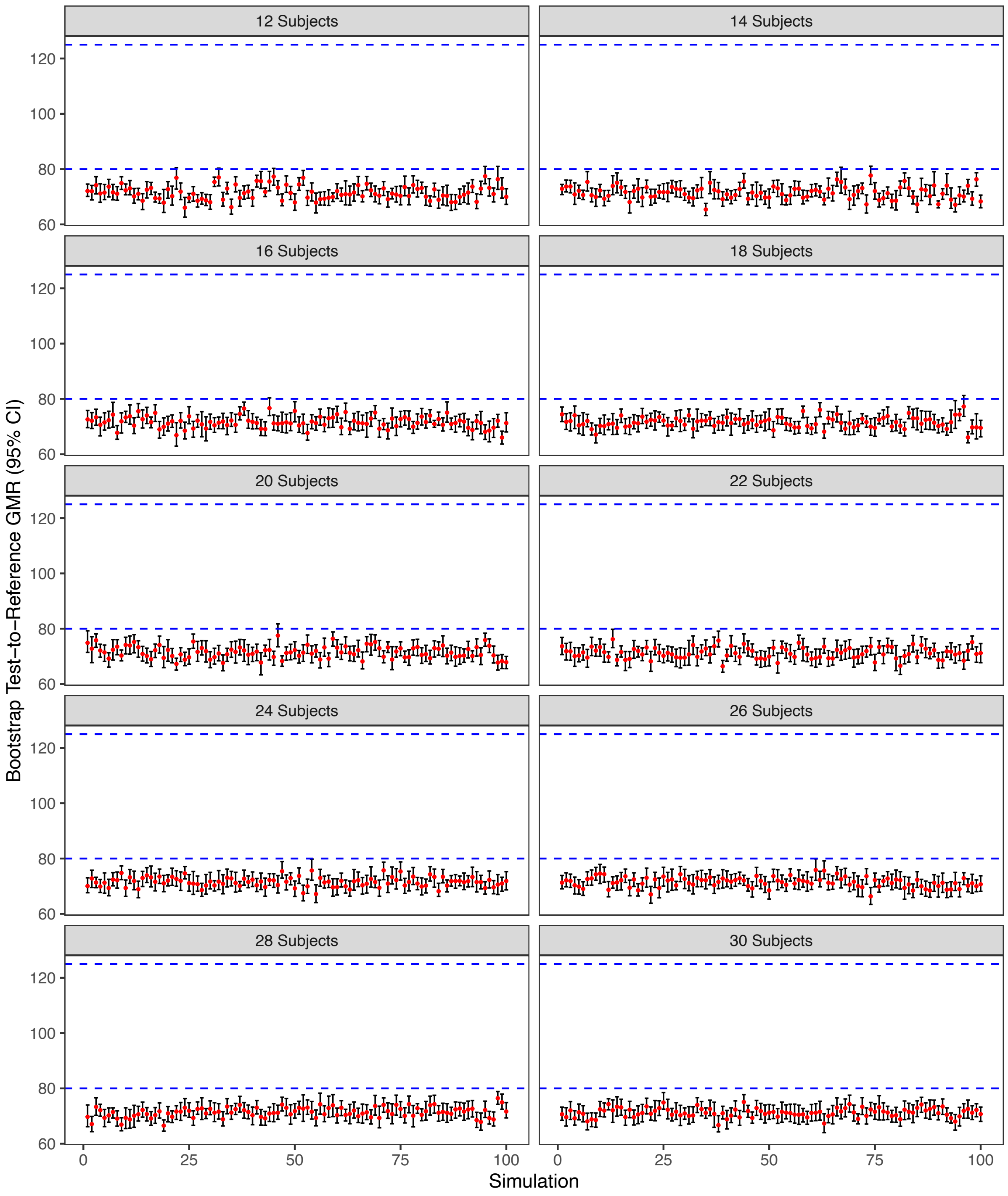

Figure S.129. Bootstrap Bioequivalence Analysis Results (Test-to-Reference GMR and Corresponding 95% CI) for C_{max} Derived from True Bioinequivalent Simulations with 30% IIV & 20% IOV in k_e

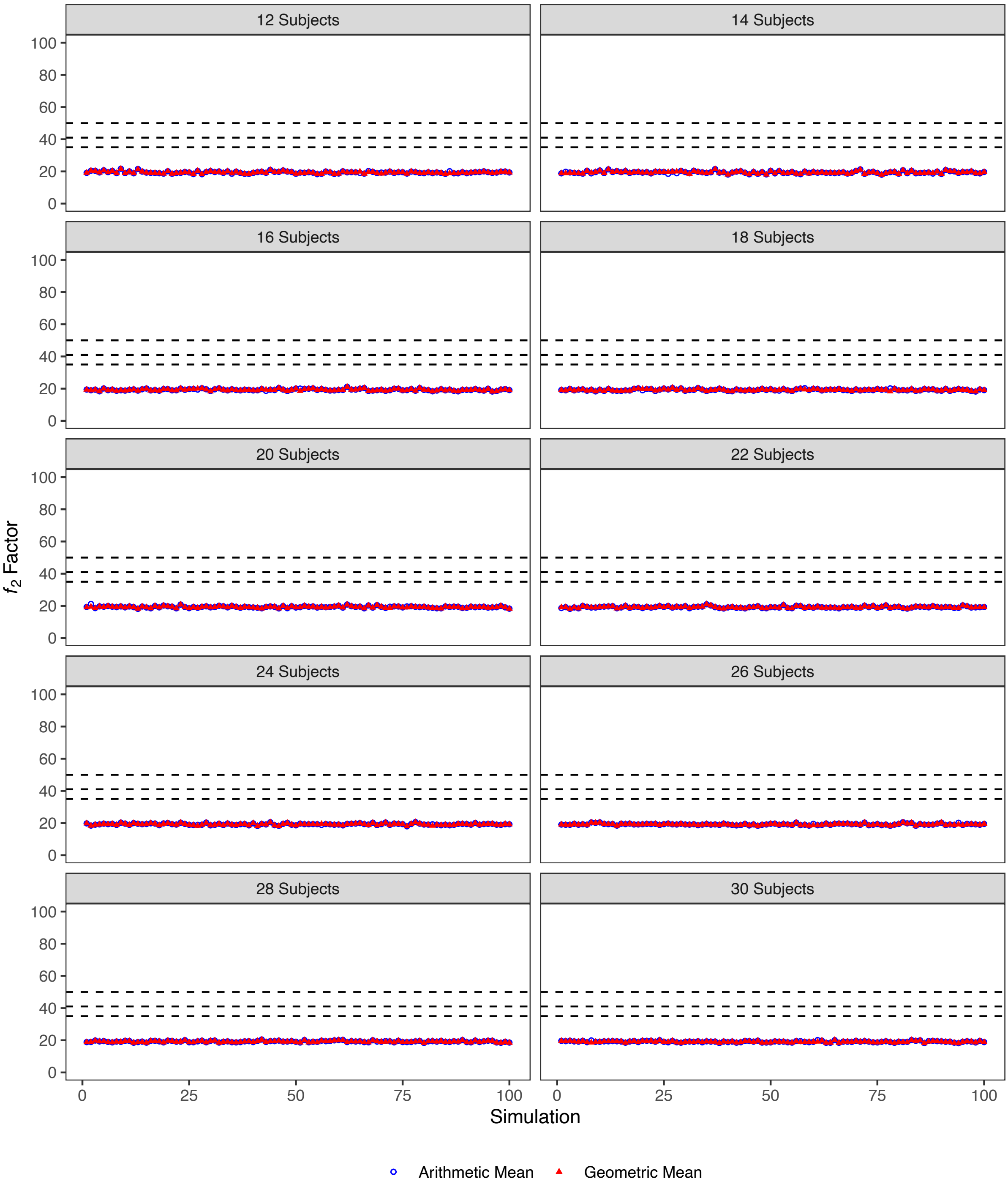

Figure S.130. Similarity f_2 Factor Results, Derived from Arithmetic and Geometric Mean Concentration-Time Profiles Until the Reference C_{max}, Derived from True Bioinequivalent Simulations with 30% IIV & 20% IOV in k_e

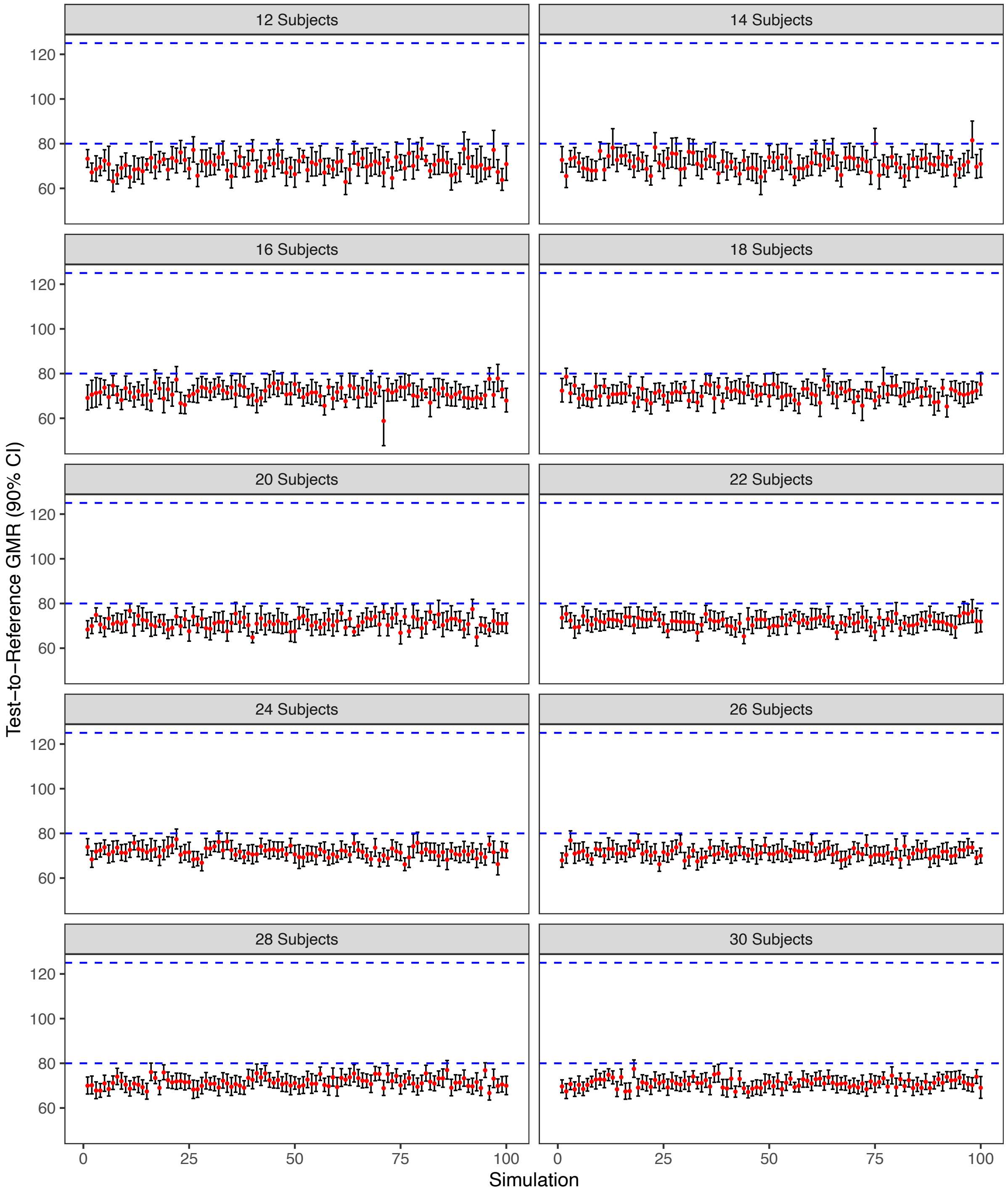

Figure S.131. Average Bioequivalence Analysis Results (Test-to-Reference GMR and Corresponding 90% CI) for C_{max} Derived from True Bioinequivalent Simulations with 30% IIV & 30% IOV in k_e

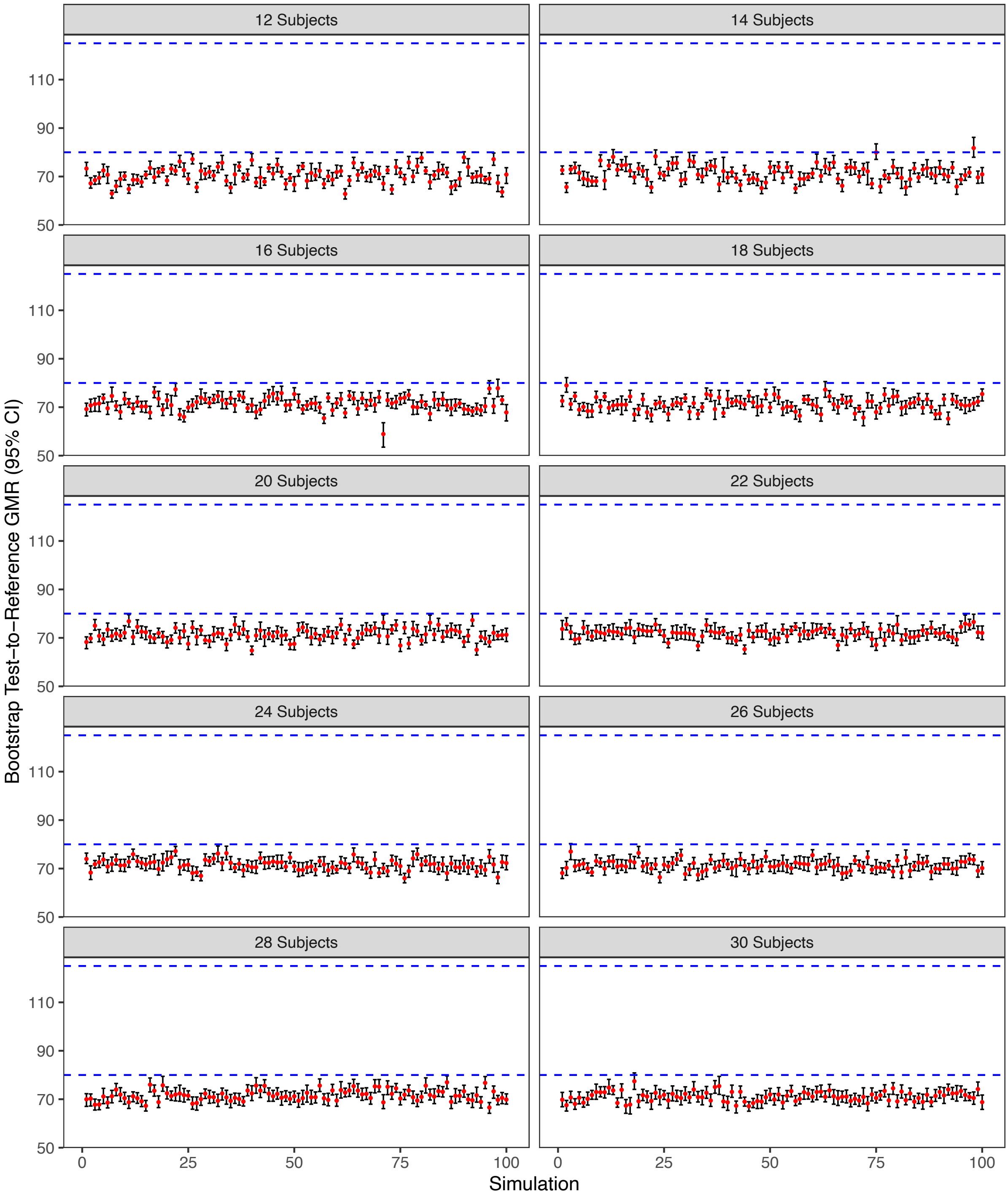

Figure S.132. Bootstrap Bioequivalence Analysis Results (Test-to-Reference GMR and Corresponding 95% CI) for C_{max} Derived from True Bioinequivalent Simulations with 30% IIV & 30% IOV in k_e

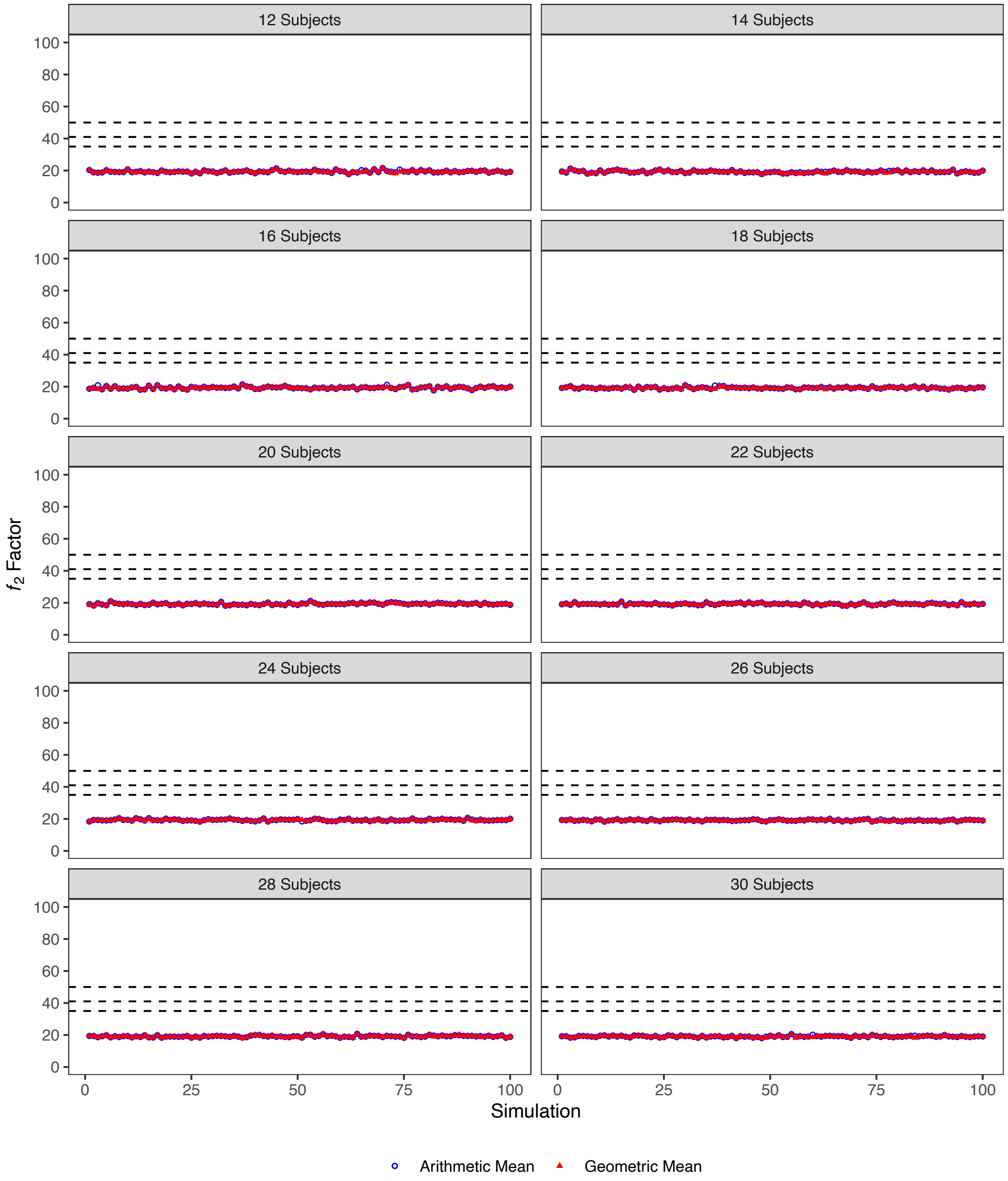

Figure S.133. Similarity f_2 Factor Results, Derived from Arithmetic and Geometric Mean Concentration-Time Profiles Until the Reference C_{max}, Derived from True Bioinequivalent Simulations with 30% IIV & 30% IOV in k_e

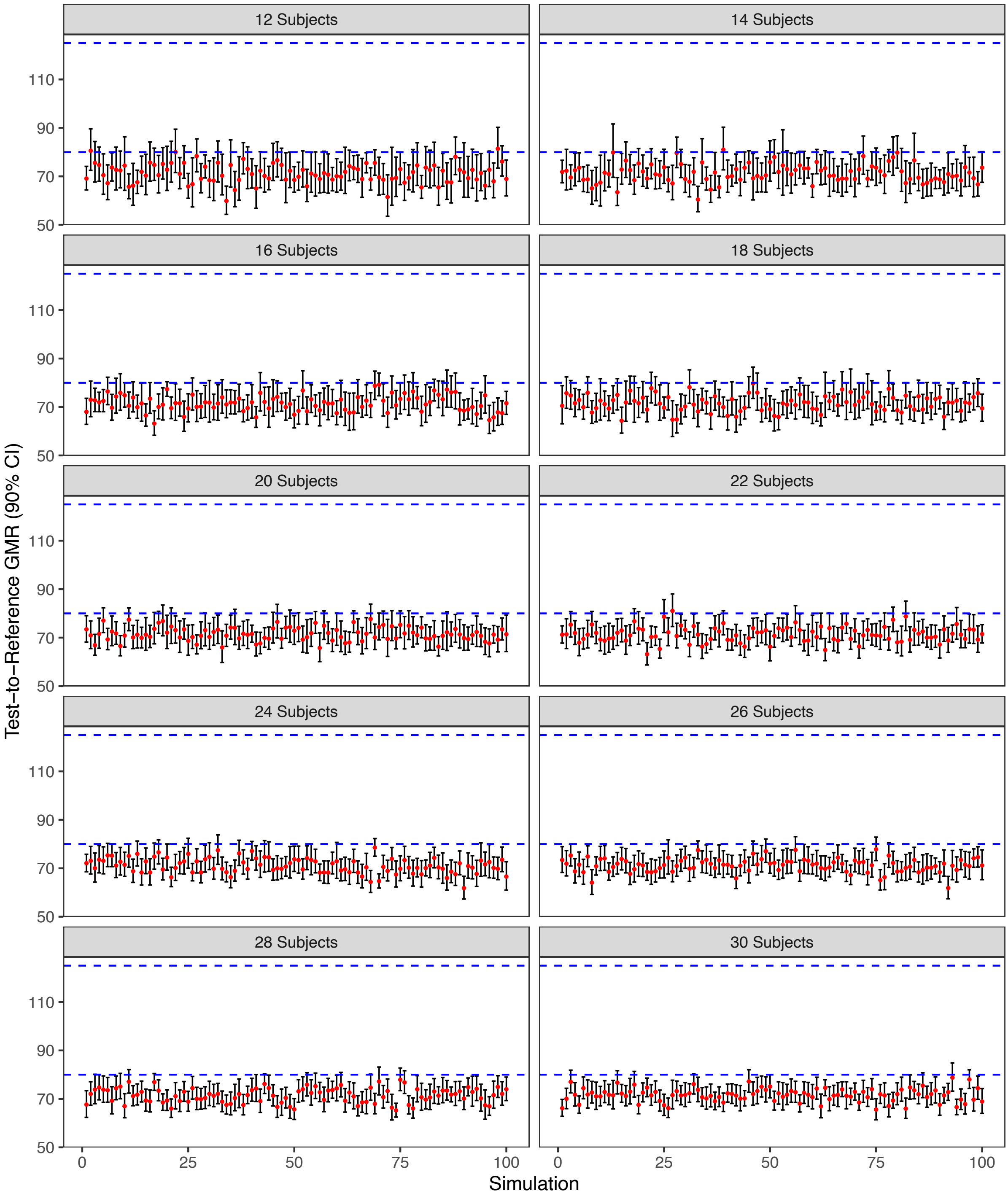

Figure S.134. Average Bioequivalence Analysis Results (Test-to-Reference GMR and Corresponding 90% CI) for C_{max} Derived from True Bioinequivalent Simulations with 0% IIV & 45% IOV in k_e

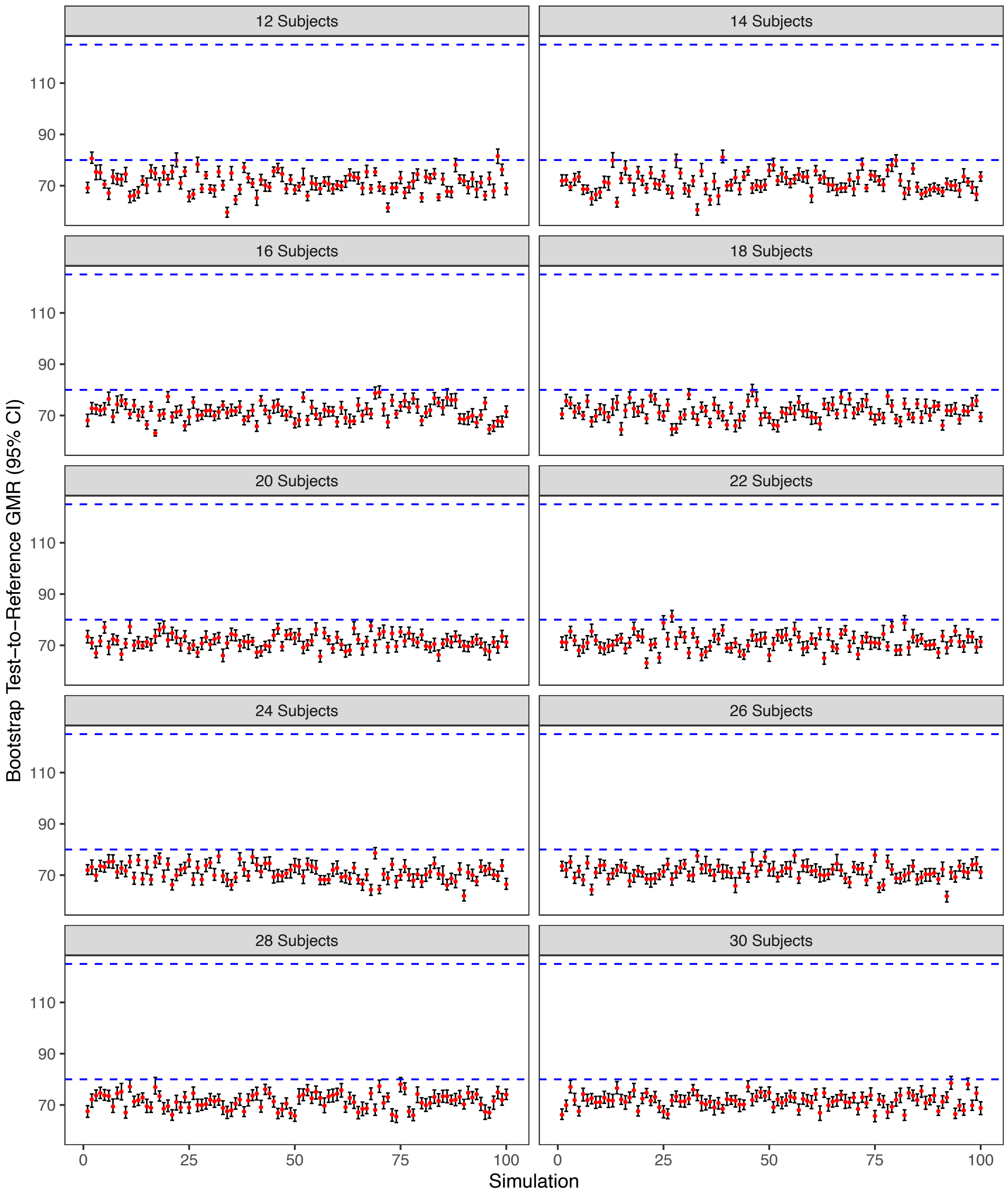

Figure S.135. Bootstrap Bioequivalence Analysis Results (Test-to-Reference GMR and Corresponding 95% CI) for C_{max} Derived from True Bioinequivalent Simulations with 0% IIV & 45% IOV in k_e

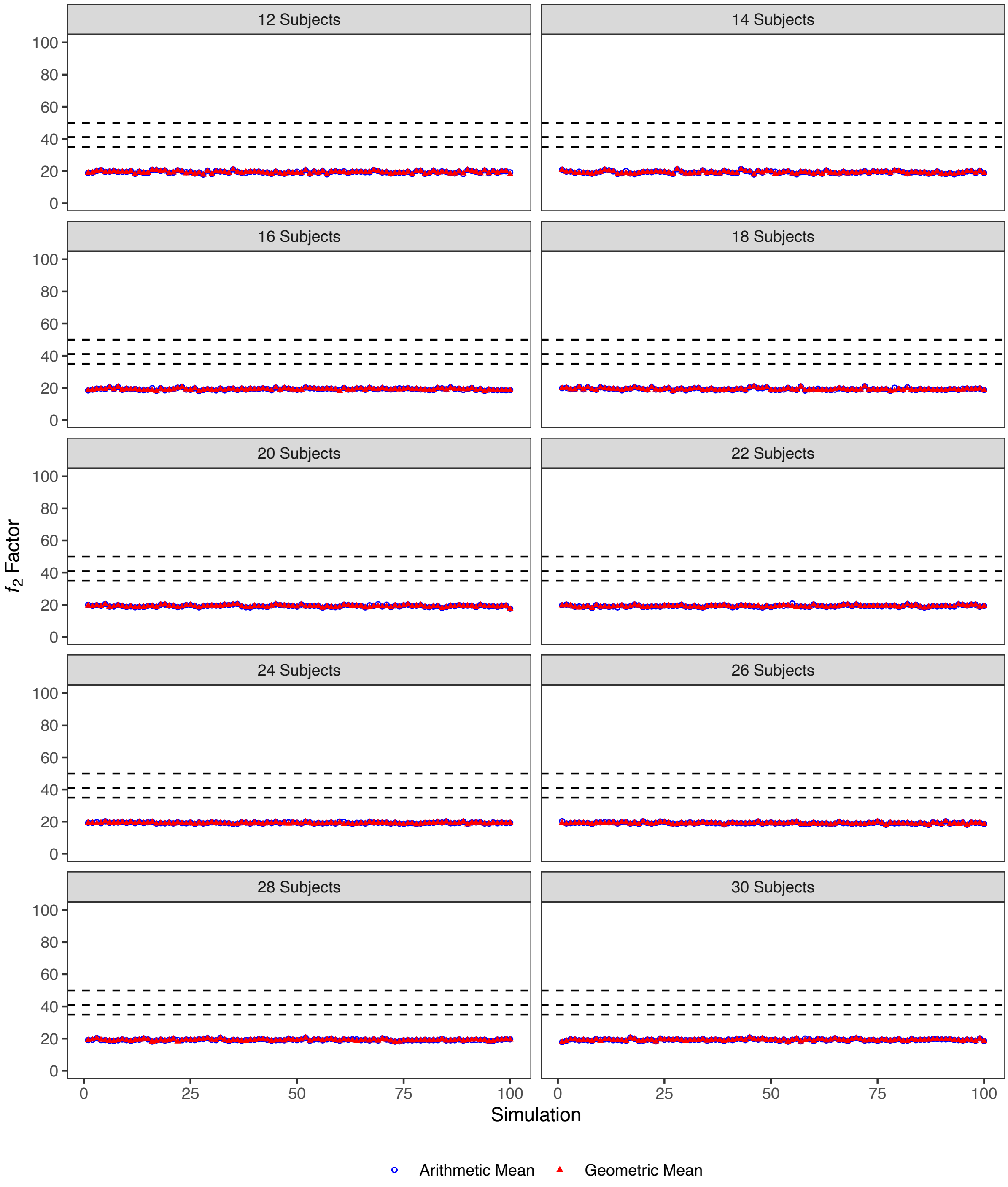

Figure S.136. Similarity f_2 Factor Results, Derived from Arithmetic and Geometric Mean Concentration-Time Profiles Until the Reference C_{max}, Derived from True Bioinequivalent Simulations with 0% IIV & 45% IOV in k_e
